# CYP2D6-Guided Opioid Management and Postoperative Pain Control: A Randomized Clinical Trial

**DOI:** 10.1001/jamanetworkopen.2025.58299

**Published:** 2026-02-20

**Authors:** Larisa H. Cavallari, Rachel A. Myers, Hrishikesh Chakraborty, Todd C. Skaar, Chancellor F. Gray, Jordan F. Baye, Simona Volpi, Renee Rider, Emily J. Cicali, Erica N. Elwood, Elizabeth C. Harris, Lindsay J. Hines, Noor A. Nahid, Khoa A. Nguyen, Aniwaa Owusu Obeng, J. Andrew Parr, Michelle A. Ramos, Lori A. Orlando, Hernan A. Prieto, Azita Sadeghpour, Rajbir Singh, Petr Starostik, Emma M. Tillman, Christina Wyatt, Carol R. Horowitz, Deepak Voora, Kathryn V. Blake, Hari K. Parvataneni, Roger B. Fillingim, Paul R. Dexter, Josh F. Peterson, Julie A. Johnson

**Affiliations:** 1 Department of Pharmacotherapy and Translational Research, College of Pharmacy, University of Florida, Gainesville; 2 Center for Pharmacogenomics and Precision Medicine, College of Pharmacy, University of Florida, Gainesville; 3 Clinical Research Unit, Department of Medicine, Duke University School of Medicine, Durham, North Carolina; 4 Department of Biostatistics and Bioinformatics, Duke University, Durham, North Carolina; 5 Department of Medicine, Indiana University School of Medicine, Indianapolis; 6 Florida Orthopaedic Institute, Gainesville; 7 Department of Pharmacy Practice, South Dakota State University, Brookings; 8 National Human Genome Research Institute, National Institutes of Health, Bethesda, Maryland; 9 Duke Clinical Research Institute, Duke University School of Medicine, Durham, North Carolina; 10 Department of Psychology, University of North Dakota, Grand Forks; 11 Brain and Spine Center, Sanford Health, Fargo, North Dakota; 12 Department of Biomedical Informatics, College of Medicine, The Ohio State University, Columbus; 13 Department of Medicine, Icahn School of Medicine at Mount Sinai, New York, New York; 14 The Charles Bronfman Institute for Personalized Medicine, Icahn School of Medicine at Mount Sinai, New York, New York; 15 Department of Orthopaedic Surgery, Indiana University School of Medicine, Indianapolis; 16 Department of Population Health Science and Policy, Institute for Health Equity Research, Icahn School of Medicine at Mount Sinai, New York, New York; 17 Duke Precision Medicine Program, Department of Medicine, Duke University School of Medicine, Durham, North Carolina; 18 Department of Orthopaedic Surgery, University of Florida School of Medicine, Gainesville; 19 Division of General Internal Medicine, Department of Medicine, Duke University School of Medicine, Durham, North Carolina; 20 Clinical and Translational Research Center, Meharry Medical College, Nashville, Tennessee; 21 Department of Pathology, Immunology and Laboratory Medicine, College of Medicine, University of Florida, Gainesville; 22 Division of Clinical Pharmacology, Indiana University School of Medicine, Indianapolis; 23 Division of Nephrology, Department of Medicine, Duke University School of Medicine, Durham, North Carolina; 24 Center for Pharmacogenomics and Translational Research, Nemours Health, Jacksonville, Florida; 25 Pain Research and Intervention Center of Excellence, University of Florida, Gainesville; 26 Regenstrief Institute, Indiana University, Indianapolis; 27 Department of Medicine, Vanderbilt University Medical Center, Nashville, Tennessee; 28 Clinical and Translational Science Institute, The Ohio State University, Columbus; 29 Department of Internal Medicine, College of Medicine, The Ohio State University, Columbus; 30 Department of Pharmaceutics and Pharmacology, College of Pharmacy, The Ohio State University, Columbus

## Abstract

**Importance:**

Individuals with genetic variation that results in absent (poor metabolizers) or reduced (intermediate metabolizers) cytochrome P450 2D6 (CYP2D6) enzyme activity have lower concentrations of highly potent active metabolites of tramadol, hydrocodone, and codeine and are thus at increased risk for inadequate pain control.

**Objective:**

To determine the effect of CYP2D6-guided opioid prescribing on postoperative pain and opioid use.

**Design, Setting, and Participants:**

This open-label randomized clinical trial enrolled participants from surgery clinics at 8 US health systems. Individuals undergoing a planned surgery anticipated to cause postoperative pain for at least 7 to 10 days were enrolled from March 2021 to September 2023, with follow-up concluded in March 2024.

**Intervention:**

Participants in the CYP2D6-guided arm underwent *CYP2D6* genotyping before surgery, with recommendations to avoid tramadol, hydrocodone, and codeine for postoperative pain in CYP2D6 poor and intermediate metabolizers, as defined by genotype and use of CYP2D6 inhibitors. Participants in the control arm had usual pain management.

**Main Outcomes and Measures:**

The primary outcome was the 10-day Silverman integrated analgesic assessment (SIA) score, a rank-based composite measure of average pain intensity on a 10-point scale, with higher scores indicating greater pain and opioid use (in morphine milligram equivalents [MMEs]). Secondary end points included individual components of the primary outcome and concordance between metabolizer phenotype and prescribed opioid 10 days after surgery. The primary analysis compared outcomes between poor and intermediate metabolizers in the CYP2D6-guided arm vs the control study arm. The analytical population comprised the subset of intent-to-treat participants with an actionable phenotype who completed surgery.

**Results:**

Of 1602 participants enrolled, 351 (mean [SD] age, 62 [13] years; 237 [68%] female) had a CYP2D6 poor or intermediate metabolizer phenotype, proceeded to surgery, and were randomized to the CYP2D6-guided (n = 176) or control (n = 175) study arm. The most common procedures in the actionable population were total knee (177 [50%]) and total hip (97 [28%]) arthroplasties. Concordance between postsurgical opioid treatment and CYP2D6 phenotype was 64% (n = 112) in the CYP2D6-guided arm and 27% (n = 47) in the control arm (difference, 37 [95% CI, 27-46] percentage points; *P* < .001). At 10 days, the mean (SD) SIA score was 1.4 (95.9) in the CYP2D6-guided arm and −1.4 (93.1) in the control arm (difference, 2.8 [95% CI, −18.3 to 23.8]; *P* = .80). Mean (SD) numeric pain intensity rating (5.2 [2.2] and 5.1 [2.3]), overall opioid use (13.7 [14.9] and 13.2 [14.7] MME/d), and other secondary end points did not differ between the CYP2D6-guided arm and the control arm.

**Conclusions and Relevance:**

In this randomized clinical trial of CYP2D6-guided postoperative opioid prescribing, there were significant prescribing changes in CYP2D6 poor and intermediate metabolizers but no differences in pain control compared with usual care. The data do not support a role for CYP2D6-guided opioid therapy in the contemporary postoperative setting of multimodal pain management.

**Trial Registration:**

ClinicalTrials.gov Identifier: NCT05966129

## Key Points

**Question:**

Does postoperative opioid therapy guided by cytochrome P450 2D6 (CYP2D6) genotype and use of CYP2D6 enzyme inhibitors alter opioid use or reduce pain intensity?

**Findings:**

This randomized clinical trial including 351 participants evaluated postoperative CYP2D6-guided opioid therapy, with recommendations to avoid hydrocodone, tramadol, and codeine in CYP2D6 poor and intermediate metabolizers, vs usual opioid prescribing without genotyping. CYP2D6-guided opioid prescribing did not affect the primary composite end point of pain intensity or overall opioid use.

**Meaning:**

Findings suggest that a CYP2D6-guided approach to opioid selection did not improve pain control in the setting of multimodal approaches to postoperative pain management.

## Teaser

This randomized clinical trial assesses whether postoperative opioid therapy guided by cytochrome P450 2D6 genotype alters opioid use or improves pain control among individuals undergoing a planned surgery anticipated to cause postoperative pain for at least 7 to 10 days.

## Introduction

Opioids remain a mainstay of postoperative pain management, with hydrocodone, oxycodone, and tramadol being the most commonly prescribed.^1^ These drugs, similar to codeine, undergo O-demethylation via the cytochrome P450 2D6 (CYP2D6) enzyme to more potent metabolites.^2^ Approximately 15% of individuals have genetic variation that results in reduced (intermediate metabolizers [IMs]) or absent (poor metabolizers [PMs]) CYP2D6 enzyme activity and lower concentrations of active opioid metabolites.^2,3^ Commonly prescribed medications (eg, fluoxetine, paroxetine, bupropion, and duloxetine) also reduce CYP2D6 activity and impair opioid metabolism through a process called phenoconversion. Reduced metabolism can result in ineffective analgesia with hydrocodone, tramadol, and codeine; the data are less clear for oxycodone.^2,4^ CYP2D6 activity is increased in approximately 3% of individuals (ultra-rapid metabolizers [UMs]), resulting in higher concentrations of more active opioid metabolites and an increased risk for toxicity.^2,3^

A single-center pilot trial showed a reduction in opioid use without a compromise in pain control when recommendations were provided to avoid tramadol, hydrocodone, and codeine in patients with the CYP2D6 IM or PM phenotype.^5^ Based on these pilot trial results, A Depression and Opioid Pragmatic Trial in Pharmacogenetics (ADOPT PGx) aimed to determine the effect of CYP2D6-guided opioid prescribing on pain control and opioid exposure as quantified by morphine milligram equivalents (MMEs) at 10 days after surgery, with the analytical population comprising patients with the IM or PM phenotype.

## Methods

### Study Design

ADOPT PGx was a set of 3 randomized clinical trials conducted in parallel,^6,7,8^ evaluating whether pharmacogenetic testing improved medication-related outcomes of depression, acute pain, and chronic pain. The ADOPT PGx Acute Pain trial is part of a Master Protocol Research Program platform trial^9^ that was registered June 22, 2020, prior to the first patient enrollment. In July 2023, each of the 3 trials under the program was registered under a separate National Clinical Trial identifier number,^6,7,8^ and the Master Protocol Research Program was converted to a screening record per recent guidance. The current analysis is for the ADOPT PGx Acute Pain trial, registered under NCT05966129, and the trial rationale and design have been previously published.^6^ Briefly, this was a prospective, multicenter, pragmatic, open-label randomized clinical trial designed to demonstrate the superiority of CYP2D6-guided postoperative pain management vs usual care. Pragmatic elements of the trial included broad inclusion criteria, heterogeneity in delivery of prescribing recommendations, prescriber autonomy in opioid-prescribing and multimodal pain management decisions, and an outcome (ie, pain control) highly relevant to participants.^10,11,12,13^ Participants were recruited between March 2021 and September 2023 from surgery clinics at 8 US health systems (eAppendix 5 in Supplement 1). Follow-up concluded in March 2024. The protocol was approved by the Duke University institutional review board, serving as the single approval board, and all participants provided written informed consent. The trial protocol, protocol changes, and statistical analysis plan are included in Supplement 2. ADOPT PGx clinical groups and study team members are listed in eAppendixes 1-4 in Supplement 1 and that the trial monitoring plan is provided in eAppendix 10 in Supplement 1. This report followed the Consolidated Standards of Reporting Trials (CONSORT) guideline.^14^ A patient advisory board was involved in the trial design.

### Participants

Eligible participants were 8 years of age or older with a scheduled surgery. The goal was to recruit participants from practices in which hydrocodone, tramadol, or codeine was usually prescribed postoperatively. Individuals undergoing procedures for which there was a reasonable expectation of pain 7 to 10 days postoperatively were prioritized for enrollment. Patients with chronic opioid use, defined as use on most days of the week during the past 3 months, were excluded. Full eligibility criteria and recruitment procedures are described in eAppendix 6 and eAppendix 7, respectively, in Supplement 1 and in the trial protocol (Supplement 2).

### Randomization and Masking

Eligible participants were randomized 1:1 to immediate pharmacogenetic testing and CYP2D6-guided postoperative opioid therapy or usual pain management (control) with pharmacogenetic testing after the participant completed study procedures. Randomization was stratified by clinic site and pediatric or adult patient, using a random block size within each site (eAppendix 11 in Supplement 1). Assignment was generated in real time using REDCap at the coordinating center and was not blinded to participants or health care professionals but masked to personnel administering follow-up surveys.

### Procedures

Genotyping details and phenotype assignment, based on genotype and use of CYP2D6 enzyme inhibitors, are described in eAppendix 8 in Supplement 1 and the published article describing the design.^6^ The CYP2D6 PM phenotype was defined as an activity score of 0; the IM phenotype was defined as an activity score higher than 0 and less than or equal to 0.75. Recommendations, delivered to most sites via automated alerts and consult notes (eAppendix 8 in Supplement 1), were to avoid hydrocodone, tramadol, and codeine in CYP2D6 PMs, IMs, and UMs in the CYP2D6-guided arm, with the ultimate prescribing decision left to the prescriber.^6^ Oxycodone avoidance was also recommended in CYP2D6 UMs given the toxicity risk, but oxycodone was considered acceptable for CYP2D6 PMs and IMs.^2^ Investigators did not actively share genotype results with participants but also did not prevent patients from learning their results through other channels (eg, patient portal).

At 10 (±3) days and at 1 (±7 days), 3 (±14 days), and 6 (±14 days) months after surgery, adult participants were asked to complete surveys to rate their average pain on a 10-point scale, per the Patient-Reported Outcomes Measurement Information System (PROMIS)^15^ Numeric Rating Scale; this result was referred to as numeric pain intensity. A second measure, composite pain intensity, combined the patients’ current pain and their worst and average pain in the past 7 days, each on a 5-point Likert scale, according to the PROMIS Pain Intensity Scale.^16,17^ Data for pediatric participants (age, <14 years) were collected from a parent. At 10 days and 1 month, participants completed 8-item surveys, developed for an earlier pilot trial, to assess opioid use (eAppendix 13 in Supplement 1).^5^ Additional assessments included mobility (per the PROMIS Mobility survey) at each follow-up time point, overall well-being (per the PROMIS-43 or Pediatric-37 instrument) at 1, 3, and 6 months, and participant awareness of their results during the trial.^18,19^ Participants were called at the 10-day time point for a full medication reconciliation. The primary outcome was at 10 days, but data were collected up to 6 months to assess time trends in pain intensity and other outcomes (eg, opioid adverse effects, opioid misuse and persistence) to be reported elsewhere.^6^

### Outcomes

The primary outcome was the Silverman integrated analgesic assessment (SIA) score, a rank-based composite of numeric pain intensity and prescribed opioid use, at 10 days.^20^ Opioid use was calculated as the difference between tablets prescribed vs remaining at 10 days after surgery, adjusted for the number of days between discharge and survey completion, and expressed as MMEs using standard conversion factors.^21^ The score was derived in the actionable population (defined below) and separately in the total population who completed surgery. Negative values indicated low pain with minimal opioid use, whereas positive values corresponded to high pain levels with or without higher opioid consumption. Secondary end points included the individual components of the primary outcome (ie, numeric pain intensity and prescribed opioid use), composite pain intensity, SIA score based on composite instead of numeric pain intensity, and concordance between CYP2D6 phenotype and prescribed medication, all at 10 days, and mobility at 30 days. Phenotype-concordant prescribing was defined as the absence of a conflict between the CYP2D6 phenotype and any opioid medication. Prescriptions of an opioid other than tramadol, hydrocodone, or codeine for a participant with the PM or IM phenotype were considered concordant. When more than 1 opioid was prescribed, prescriptions were only considered concordant if codeine, tramadol, and hydrocodone were not among the opioids prescribed. Overall well-being, its subdomains, composite pain intensity and opioid use at 1 month, and time trends in composite pain intensity from 10 days to 6 months are also reported. Early in the trial, it was determined that it was not feasible to define dose consumed for a liquid opioid. Thus, opioid use and SIA score were not captured for liquid opioids. Additional details for opioid use and SIA score derivations are available in eAppendix 12 in Supplement 1.

### Sample Size Calculation

Power calculations were based on pilot trial data suggesting that health care professional adherence to CYP2D6-guided recommendations in the guided arm would be higher than 70% and have a Cohen *d* of 0.375.^6^ A sample size of 304 participants (152 in each arm) with an IM or PM phenotype was estimated to provide 90% power to detect a standardized effect size (Cohen *d* [mean difference]/[SD]) of 0.375 for the SIA score between arms, with a 2-sided type 1 error rate of .05.^22^ Assuming that 10% would drop out or be unavailable for follow-up, an additional 7% would not proceed to surgery, and 21% to 24% would have the IM or PM phenotype, we estimated that 1518 to 1732 total participants needed to be enrolled.^5^

### Statistical Analysis

The primary analysis population consisted of randomized participants who completed surgery and had the CYP2D6 IM or PM phenotype, hereafter referred to as the actionable population. The mean SIA score and secondary outcomes, with the exception of concordance, were compared between the CYP2D6-guided and control arms of the actionable population using a 2-sample *t* test. Concordance and use of pain modalities were compared between arms using a χ^2^ or Fisher exact test. Opioid use at 10 days and at 1 month were compared using the Wilcoxon rank sum test. As a sensitivity analysis, covariate-adjusted linear regression was conducted to account for differences in baseline characteristics between arms. Age, self-reported gender (male, female, other, or prefer not to answer), self-reported race and ethnicity (Black or African American, Hispanic or Latino, White or European American, and other, which included Asian, American Indian, Middle Eastern or North African or Mediterranean, Native American or Alaska Native, Native Hawaiian or other Pacific Islander, or more than 1 race), and surgery type were included as covariates in the adjusted analyses. Race and ethnicity were assessed to more fully characterize the cohort, and category selections were guided by the National Academies of Sciences, Engineering, and Medicine.^23^ Longitudinal patterns in composite pain score from 10 days to 6 months were compared using covariate-adjusted linear mixed effect regression, with age, gender, race, and surgery type included as covariates.

Complementary analyses of the primary end point were conducted in the per-protocol (ie, therapy concordant with recommendations for the CYP2D6-guided arm) and nonoxycodone (ie, per-protocol population excluding participants prescribed oxycodone in the CYP2D6-guided arm) subgroups (eAppendix 9 in Supplement 1). The SIA score was also assessed in the nonactionable population (ie, participants without an IM or PM phenotype), participants who underwent total knee or total hip arthroplasty, and participants in the CYP2D6-guided arm who became aware of their genotype during the trial vs those who did not. A post hoc analysis assessed the effect of oxycodone use on intervention effect. All tests were 2-sided and assessed at a significance threshold of .05 without adjustment for multiple testing. SIA scores were missing in higher than 10% of the actionable population. All other end points were analyzed using the data as reported, without imputation. Imputation is described in eAppendix 12 in Supplement 1. All analyses were conducted with SAS, version 9.45 (SAS Institute Inc).

## Results

### Trial Participants

**Figure 1.**
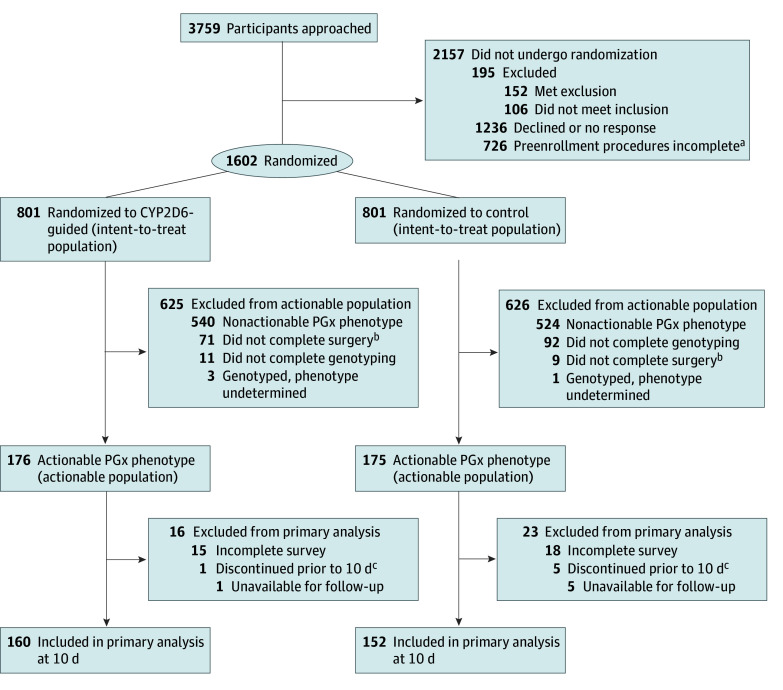
Flow Chart of Screening, Randomization, and Population for Primary Analyses The intent-to-treat population included all randomized participants. The analytical population comprised the subset of intent-to-treat participants with an actionable phenotype (eTable 1 in Supplement 1). CYP2D6 indicates cytochrome P450 2D6; PGx, pharmacogenetic. ^a^Participants did not attend the baseline visit, had an incomplete baseline visit, or could not be reached for scheduling. ^b^Of the participants who were genotyped. ^c^Participants who discontinued prior to 10 days, were unavailable for follow-up, and provided no analyzable data at 10 days after surgery. These participants were included in imputation analyses and had pain and opioid use imputed.

Of 1602 participants enrolled, 351 (mean [SD] age, 62 [13] years; 237 [68%] self-reported female, 112 [32%] male, and 2 (<1%) other gender or preferred not to answer; 51 [14%] self-reported Black or African American, 273 [78%] White or European American, and 27 [8%] other or unknown race or preferred not to answer) proceeded to surgery, had the IM or PM phenotype, and were randomized to the CYP2D6-guided (n = 176) or control (n = 175) arm (Figure 1). These patients constituted the actionable population (eTable 1 in Supplement 1). The percentage of participants with an actionable phenotype increased from 14% to 24% after phenoconversion was considered (eTable 2 in Supplement 1). Baseline characteristics were well-balanced between arms, excluding psychiatric disorders for all randomized participants and surgery type for the actionable population (Table 1). The most common procedures were total knee (177 or 50% of the actionable population) and total hip (97 or 28% of the actionable population) arthroplasties. Genotype results were available before surgery for 165 of 176 participants (94%) in the CYP2D6-guided arm, whereas 100% of participants in the control arm had their results reported after their surgery.

**Table 1.** Baseline Patient Characteristics^a^

Characteristic	CYP2D6 intermediate and poor metabolizers		All randomized patients	
	CYP2D6-guided arm (n = 176)	Control arm (n = 175)	CYP2D6-guided arm (n = 801)	Control arm (n = 801)
Age, mean (SD), y	62.8 (12.1)	61.8 (13.1)	62.4 (14.2)	62.8 (13.7)
Gender, No./total No. (%)				
Female	116/176 (66)	121/175 (69)	493/801 (62)	487/801 (61)
Male	59/176 (34)	53/175 (30)	304/801 (38)	313/801 (39)
Other or prefer not to answer	1/176 (<1)	1/176 (<1)	4/801 (<1)	1/801 (<1)
Race, No./total No. (%)				
Black or African American	22/176 (13)	29/175 (17)	122/801 (15)	104/801 (13)
White or European American	139/176 (79)	134/175 (77)	602/801 (75)	621/801 (78)
Other race, unknown, or preferred not to answer^b^	15/176 (9)	12/175 (7)	77/801 (106)	76/801 (10)
Hispanic or Latino ethnicity, No./total No. (%)^c^	5/176 (3)	6/175 (3)	43/801 (5)	54/801 (7)
BMI, mean (SD)	32.1 (7.0)	32.6 (7.7)	31.6 (7.2)	31.3 (6.9)
Medical history, No./total No. (%)				
Diabetes	33/176 (19)	33/175 (19)	137/801 (17)	146/800 (18)
Hypertension	88/174 (51)	100/175 (57)	387/799 (48)	400/800 (50)
Depression	69/176 (39)	72/175 (41)	191/800 (24)	195/800 (24)
Anxiety	68/176 (39)	67/174 (38.5)	202/801 (25)	211/799 (26)
Psychiatric disorder^d^	14/176 (8)	8/175 (5)	43/801 (5)	25/799 (3)
Cancer	38/175 (22)	48/175 (27)	178/799 (22)	218/800 (27)
Pain numeric rating, mean (SD)^e^	5.9 (2.4)	6.1 (2.6)	5.7 (2.5)	5.7 (2.5)
Nonopioid analgesic prior to surgery, No./total No. (%)				
Acetaminophen	55/172 (32)	58/172 (34)	226/756 (30)	222/744 (30)
NSAID	66/172 (38)	79/172 (46)	279/756 (37)	291/744 (39)
Gabapentinoid	46/172 (27)	39/172 (23)	142/756 (19)	117/744 (16)
Surgery type, No./total No. (%)^f^^,^^g^				
Total knee arthroplasty	87/176 (49)	90/175 (51)	310/719 (43)	307/700 (44)
Total hip arthroplasty	50/176 (28)	47/175 (27)	207/719 (29)	209/700 (30)
Other joint arthroplasty	8/176 (4)	12/175 (7)	37/719 (5)	36/700 (5)
Spinal	11/176 (6)	3/175 (27)	46/719 (6)	34/700 (5)
Breast	6/176 (3)	3/175 (2)	39/719 (5)	36/700 (5)
Arthroscopic	8/176 (4)	3/175 (2)	34/719 (5)	25/700 (4)
Other^h^	6/176 (3)	17/175 (10)	46/719 (6)	53/700 (8)
Surgery admission length of stay, mean (SD), d^f^	1.1 (1.7)	1.1 (1.8)	1.1 (2.0)	1.1 (1.8)
Discharge status, No./total No. (%)^f^				
Home	163/176 (93)	162/175 (93)	678/719 (94)	666/700 (95)
Skilled nursing facility	5/176 (3)	9/175 (5)	23/719 (3)	23/700 (3)
Inpatient rehabilitation facility	7/176 (4)	4/175 (2)	16/719 (2)	9/700 (1)
Hospice	1/176 (1)	0/175 (0)	2/719 (<1)	0/722 (0)
Acute care facility	0/176 (0)	0/175 (0)	0/719 (0)	2/700 (<1)

Abbreviations: BMI, body mass index (calculated as weight in kilograms divided by height in meters squared); CYP2D6, cytochrome P450 2D6; NSAID, nonsteroidal anti-inflammatory drug.

^a^ Percentages are based on the total number of patients with nonmissing responses per question.

^b^ Because this includes numbers less than 5, we did not separate these groups, but other race includes individuals reporting as Asian, American Indian, Middle Eastern or North African or Mediterranean, Native American or Alaska Native, Native Hawaiian or other Pacific Islander, or more than 1 race.

^c^ Denominator includes 1 participant in the actionable population and 8 participants among all randomized participants who preferred not to answer.

^d^ The *P* value for psychiatric disorders between the CYP2D6-guided arm and the control arm for all randomized patients was .03.

^e^ Numeric pain rating intensity on a scale of 0 to 10, with higher values indicating greater pain.

^f^ Data provided for 351 intermediate and poor metabolizers and 1419 total randomized patients who proceeded to surgery and were genotyped.

^g^ The *P* value for surgery type between the CYP2D6-guided arm and the control arm of the actionable population was .02.

^h^ Other surgery types that did not fit into other classifications (eg, ostomy, fracture repair, and open abdominal).

Within the actionable population, 172 of 176 participants in the CYP2D6-guided and 171 of 175 in the control arm, 98% in each arm, were prescribed an opioid, with only 1 participant being prescribed a liquid opioid (Table 2). The most often prescribed opioids were hydromorphone (69 of 176 [39%]) and oxycodone (59 of 176 [34%]) in the CYP2D6-guided arm and hydrocodone (101 of 175 [58%]) and oxycodone (68 of 175 [39%]) in the control arm. Significantly fewer participants in the CYP2D6-guided arm were prescribed hydrocodone (−33.3% [95% CI, −43.0% to 23.6%]; *P* < .001). No participant was prescribed codeine. Among the participants who were prescribed an opioid, 25 (16%) in the CYP2D6-guided arm and 16 (11%) in the control arm reported not taking any of their prescribed opioid at 10 days.

**Table 2.** Postsurgical Pain Medication, Nerve Block Use, and Concordance Between Opioid Medication and CYP2D6 Phenotype

	All participants with an actionable phenotype, No./total No. (%) (n = 351)	Participants in the CYP2D6-guided arm, No./total No. (%) (n = 176)	Participants in the Control arm, No./total No. (%) (n = 175)	*P* value^a^
Opioid^b^				
Tramadol	60/351 (17)	25/176 (14)	35/175 (20)	.15
Hydrocodone	144/351 (41)	43/176 (24)	101/175 (58)	<.001
Oxycodone	127/351 (36)	59/176 (34)	68/175 (39)	.30
Hydromorphone	75/351 (21)	69/176 (39)	6/175 (3)	<.001
Meperidine	1/351 (<1)	1/176 (<1)	0/175 (0)	>.99
No opioid prescribed	8/351 (2)	4/176 (2)	4/175 (2)	>.99
Concordance between prescribed postsurgical opioid medication and CYP2D6 phenotype	159/351 (45)	112/176 (64)	47/175 (27)	<.001
Nerve block use	193/351 (55)	98/176 (56)	95/175 (54)	.61
Nerve block removed after discharge	73/110 (66)	37/58 (64)	36/52 (69)	.55
Nonopioid analgesic^c^				
Acetaminophen	229/351 (65)	114/176 (65)	115/175 (66)	.94
NSAID	149/322 (46)	72/165 (44)	77/157 (49)	.33
Gabapentinoid	108/322 (34)	61/165 (37)	47/157 (30)	.18

Abbreviations: CYP2D6, cytochrome P450 2D6; NSAID, nonsteroidal anti-inflammatory drug.

^a^ *P* values are for comparisons between the CYP2D6-guided arm and the control arm based on a χ^2^ test or Fisher exact test (for cell counts <5).

^b^ Data reflect opioids prescribed; patients may have been prescribed more than 1 opioid medication.

^c^ Nonopioid analgesic use is based on participant report at the 10-day medication inventory. Analgesics included in cold medications were excluded. Acetaminophen included acetaminophen in combination with opioids (for patients who reported taking their prescribed opioid) plus additional acetaminophen use alone reported by the patient.

In the actionable population, 193 of 351 (55%) received a nerve block, and 73 of 110 (66%) of those responding reported removal of the nerve block after hospital discharge, with no difference between study arms (Table 2). Use of nonopioid analgesic medications was also common (eg, 229 of 351 [65%] reported taking acetaminophen, 149 of 322 [46%] nonsteroidal anti-inflammatory drugs, and 108 of 322 [34%] gabapentinoids) and did not differ between arms (Table 2). Concordance between prescribed opioids and CYP2D6 phenotype was higher in the CYP2D6-guided arm (112 [64%]) compared with the control arm (47 [27%]) (difference, 37 [95% CI, 27-46] percentage points; *P* < .001).

### Primary and Secondary End Points

**Figure 2.**
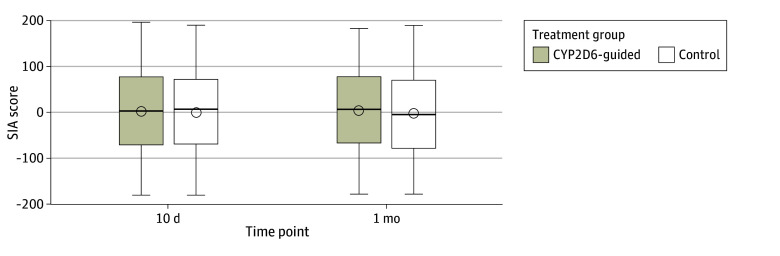
Box Plots Comparing Silverman Integrated Analgesic Assessment (SIA) Scores in Cytochrome P450 2D6 (CYP2D6)-Guided and Control Groups in the Actionable Phenotype Population SIA scores are shown for 160 participants at 10 d and 163 participants at 1 mo for the treatment group and 152 participants at 10 d and 159 participants at 1 mo for the control group. Negative values indicate low pain with minimal opioid use; positive values, high pain levels or higher opioid use. The horizontal line within each box represents the median; top and bottom of each box, upper and lower limits of the interquartile range; whiskers, 1.5 times the interquartile range; circles within boxes, mean values.

Data were available to calculate the SIA score for 160 participants (91%) in the CYP2D6-guided arm and 152 participants (87%) in the control arm of the actionable population. No differences were observed in the mean (SD) SIA scores between the CYP2D6-guided arm and the control arm at 10 days (1.4 [95.9] vs −1.4 [93.1]; difference, 2.8 [95% CI, −18.3 to 23.8]; *P* = .80) or at 1 month (2.6 [91.8] vs −2.7 [98.8]; difference, 5.2 [95% CI, −15.7 to 26.2]; *P* = .62) (Figure 2). The results did not differ after imputation of missing data (difference, 1.7 [95% CI, 18.4-23.8]). Additionally, no differences were found in any of the secondary end points between study arms (eg, mean [SD], 5.2 [2.2] and 5.1 [2.3] for numeric pain rating and 13.7 [14.9] and 13.2 [14.7] MME/d for overall opioid use) (Table 3; eTable 3 in Supplement 1). After adjusting for age, gender, race and ethnicity, and surgery type, there remained no differences in the 10-day SIA scores between the CYP2D6-guided arm (least squares mean [SE], −27.7 [32.8]) and control arm (least squares mean [SE], −31.6 [32.6]) (least squares mean difference, 3.9 [95% CI, −16.8 to 24.6]; *P* = .71). No differences were observed in the time patterns for the composite pain score from 10 days to 6 months between study arms (eTable 4 in Supplement 1).

**Table 3.** Secondary Outcomes in CYP2D6 Intermediate and Poor Metabolizers

Outcome	CYP2D6-guided arm (n = 176)		Control arm (n = 175)		*P* value
	No.	Mean (SD)	No.	Mean (SD)	
Numeric pain intensity at 10 d^a^	168	5.2 (2.2)	160	5.1 (2.3)	.83
Composite pain intensity at 10 d^b^	168	9.0 (2.2)	160	9.1 (2.4)	.64
Opioid use at 10 d, MME/d^c^	160	13.7 (14.9)	152	13.2 (14.7)	.91
Mobility at 30 d^d^	164	40.4 (16.6)	161	39.4 (17.2)	.60
Overall well-being at 1 mo^e^	165	48.3 (6.0)	162	47.7 (6.4)	.51

Abbreviations: CYP2D6, cytochrome P450 2D6; MME, morphine milligram equivalent; PROMIS, Patient-Reported Outcomes Measurement Information System.

^a^ Numeric pain intensity on a scale of 0 to 10, with higher values indicating worse pain.

^b^ Composite of worst and average pain during the past 7 days and current pain, each on a 5-point scale, with the total ranging from 3 to 15 and higher values indicating worse pain.

^c^ Nonparametric test used to assess for differences; difference and 95% CI not calculated.

^d^ Per the PROMIS Mobility Item Bank or Pediatric Mobility-Short Form 8a. Scales ranged from 15.0 to 75.0, with higher values indicating higher levels of mobility.

^e^ Per the PROMIS-43 instrument; the scale ranged from 20 to 80, with higher values indicating lower well-being.

### Subgroup Analyses

For the per-protocol population, the mean (SD) 10-day SIA score was 0.2 (101.1) for the CYP2D6-guided arm (n = 98) and −1.4 (93.1) for the control arm (n = 152) (difference, 1.6 [95% CI, −23.0 to 26.2]; *P* = .90). Neither the SIA score nor secondary end points, with the exception of physical function, differed between study arms when limiting the analysis to participants undergoing knee or hip arthroplasty (eTable 5 in Supplement 1). After excluding 33 participants from the CYP2D6-guided arm of the per-protocol population who received oxycodone (ie, nonoxycodone subgroup), the mean (SD) SIA score was −14.4 (92.9) in the guided arm (n = 65) and −1.4 (93.1) in the control arm (difference, −14.4 [95% CI, −37.1 to 8.4]; *P* = .35). After adjusting for age, gender, race and ethnicity, and surgery type, the least squares mean (SE) SIA score at 10 days was −56.7 (35.4) in the guided arm and −39.4 (33.6) in the control arm of the nonoxycodone subgroup (least squares mean difference, −17.2 [95% CI, −44.0 to 9.5]; *P* = .21). Using the SIA score estimated in the total population, no differences were found in the mean (SD) 10-day SIA scores in the guided arm (2.6 [90.9]; n = 597) vs the control arm (−3.9 [92.9]; n = 531; difference, 6.5 [95% CI, −5.2 to 18.3]; *P* = .28) for the nonactionable population. Additionally, no difference was observed in the 10-day mean (SD) SIA scores among participants in the actionable population who became aware of their genotype results (16.3 [96.8]) vs those who did not (−1.3 [98.6]; difference, 17.6 [95% CI, −16.4 to 51.5]; *P* = .31).

### Post Hoc Analysis of the Primary Outcome

Considering the control arm only, 68 of 175 (39%) of the actionable population (Table 2) and 255 of 722 (35%) of all control arm participants were prescribed oxycodone. Mean (SD) SIA scores were higher among participants prescribed vs not prescribed oxycodone for both the CYP2D6-guided arm (32.9 [107.3] vs −15.2 [85.4]; difference, 48.0 [95% CI, 18.0-78.1]; *P* = .002) and control arm (30.8 [99.7] vs −22.5 [82.4]; difference, 53.3 [95% CI, 23.4-83.2]; *P* < .001). However, there was no interaction between oxycodone use and the effect of the intervention on the SIA score (*P* = .81).

### Safety

No study-related adverse events were reported. Among participants who proceeded to surgery, 93 randomized to the CYP2D6-guided arm and 94 randomized to the control arm did not complete study participation. There were 3 nonstudy-related deaths, all in the nonactionable population.

## Discussion

This pragmatic randomized clinical trial found that genotyping participants for *CYP2D6* and providing recommendations to avoid tramadol, hydrocodone, and codeine in CYP2D6 IMs and PMs influenced postoperative opioid prescribing, with lower hydrocodone and higher hydromorphone prescribing rates in the CYP2D6-guided arm vs control arm. However, the intervention had no effect on pain control, with no difference in SIA scores or secondary outcomes between study arms.

The association between *CYP2D6* genotype and response to codeine and tramadol is well-defined.^2^ Evidence of worse pain control and more emergency department visits with concomitant use of hydrocodone and a CYP2D6 inhibitor supports reduced hydrocodone effectiveness in patients with impaired CYP2D6 activity.^24,25^ The data for oxycodone are less clear.^2^ In a study of CYP2D6-guided chronic pain management, with recommendations to avoid tramadol, hydrocodone, oxycodone, or codeine in PMs and IMs, guided therapy reduced pain in patients treated with tramadol, hydrocodone, or codeine at baseline but not in those treated with oxycodone.^26^ These data informed our approach to target enrollment of participants from sites in which tramadol, hydrocodone, or codeine (but not oxycodone) was predominately prescribed. While we considered oxycodone as an acceptable alternative in the CYP2D6-guided (but not control) arm, 35% of control arm participants were prescribed oxycodone. During the course of the trial, data emerged showing that, similar to hydrocodone, concomitant use of oxycodone and CYP2D6-inhibiting medications was associated with more frequent emergency department visits.^25^ This led us to question whether defining oxycodone as an acceptable opioid was appropriate and whether the high use of oxycodone in the control arm influenced trial outcomes. However, on post hoc analysis, no significant interaction between oxycodone use and outcomes by study arm was observed.

A multimodal approach is recommended to optimize postsurgical pain management and to minimize opioid use.^27^ Use of nerve blocks, acetaminophen, nonsteroidal anti-inflammatory drugs, and gabapentinoids, which have been associated with reduced postoperative pain or opioid use, was common in our population.^27,28^ In some cases in which sufficient data were provided, the block was not removed until after hospital discharge, thus overlapping with the 10-day period for assessing primary trial outcomes. Among trial participants prescribed an opioid, 41 (13%) reported not taking their prescribed opioid, possibly because of adequate pain control with the other modalities or concerns about the dangers of opioid use.

These findings contrast with those from an earlier pilot trial showing similar pain intensity but lower opioid use with CYP2D6-guided opioid prescribing.^5^ Oxycodone use was low in the pilot study (6% of participants), and while nearly all pilot trial participants received a nerve block, it was removed prior to discharge in most cases. Whether more frequent use of sustained nerve blocks in the current trial contributed to the inconsistent results between studies is unclear.

### Limitations

This study has limitations. Several study designs could address the impact of CYP2D6-guided opioid therapy. A traditional randomized clinical trial would have answered with the highest level of certainty the role of CYP2D6 phenotype on pain control in the postsurgical setting. A hybrid implementation-effectiveness trial could have provided insight into the effectiveness of both the implementation approaches and intervention.^29,30^ We chose a randomized pragmatic design, which is more likely than a traditional randomized clinical trial to provide insight into the clinical effectiveness of the intervention. However, this study design is likely to underestimate the effect of the CYP2D6-guided approach in an ideal setting in which health care professionals would have been required to prescribe specific opioids in IM and PM participants (thus resulting in near 100% concordance) and to follow similar multimodal pain management approaches and in which genotype information would have been blinded to both clinicians and participants.

Another limitation of this study is that we assumed a 50% reduction in enzyme activity with the use of moderate CYP2D6 inhibitors, which may have underestimated the impact of some inhibitors. In terms of our primary end point, this was the first large trial to use the SIA score, and the measure of variability (eg, SD) was large, making it difficult to observe statistical differences. Additionally, the SIA score was missing for 11% of the actionable population. However, evaluation of the components of the primary end point supports that a CYP2D6-guided approach in the postsurgical setting did not change pain control. Our power calculations also did not consider concordance, which was lower than anticipated in the CYP2D6-guided arm, or the potential impact of multimodal pain management. As reliance on opioids for postsurgical pain management declines in favor of nonopioid approaches, CYP2D6-guided opioid therapy may be less relevant.

## Conclusions

In summary, this randomized clinical trial found that a CYP2D6-guided approach led to significant changes in postsurgical opioid prescribing but had no effect on pain control or opioid use. The data do not support a role for CYP2D6-guided opioid therapy in the contemporary postoperative setting of multimodal pain management.

## Notes

Supplement 1. **eAppendix 1.** ADOPT PGx Clinical Groups **eAppendix 2.** ADOPT PGx Protocol Implementation Teams **eAppendix 3.** ADOPT PGx Site PIs and Site Study Staff **eAppendix 4.** Additional Collaborators **eAppendix 5.** Participating Health Systems **eAppendix 6.** Inclusion and Exclusion Criteria **eAppendix 7.** Recruitment **eAppendix 8**. Study Procedures **eAppendix 9.** Subgroups Analyzed **eAppendix 10.** Trial Monitoring Plan **eAppendix 11.** Randomization Procedure **eAppendix 12.** Supplemental Statistical Methods **eAppendix 13.** Opioid Consumption Survey Part 1: Prescribed Opioids **eTable 1.** CYP2D6 Phenotype Based on CYP2D6 Genotype Alone Versus CYP2D6 Genotype Plus CYP2D6 Inhibitor Use for All Randomized Participants **eTable 2.** Medications at Baseline Causing Phenoconversion **eTable 3.** Other Secondary and Exploratory Outcomes in CYP2D6 Intermediate and Poor Metabolizers **eTable 4.** Mixed-Model Analysis of Composite Pain Intensity Score Trends From 10 Days to 6 Months in the Actionable Population **eTable 5.** SIA Score and PROMIS-43 Subscales at 10 Days by Surgery Type in the Actionable Population

Supplement 2.
Trial Protocol and Statistical Analysis Plan

Supplement 3.
Group Information

Supplement 4.
Data Sharing Statement
