# Supplementary material for: CYP2D6-Guided Opioid Management and Postoperative Pain Control: A Randomized Clinical Trial
Source: JAMA Netw Open. 2026 Feb 20;9(2):e2558299. doi: 10.1001/jamanetworkopen.2025.58299 (PMC12924106; doi:10.1001/jamanetworkopen.2025.58299)
Supplement: Supplement 1. — eAppendix 1. ADOPT PGx Clinical Groups eAppendix 2. ADOPT PGx Protocol Implementation Teams eAppendix 3. ADOPT PGx Site PIs and Site Study Staff eAppendix 4. Additional Collaborators eAppendix 5. Participating Health Systems eAppendix 6. Inclusion and Exclusion Criteria eAppendix 7. Recruitment eAppendix 8. Study Procedures eAppendix 9. Subgroups Analyzed eAppendix 10. Trial Monitoring Plan eAppendix 11. Randomization Procedure eAppendix 12. Supplemental Statistical Methods eAppendix 13. Opioid Consumption Survey Part 1: Prescribed Opioids eTable 1. CYP2D6 Phenotype Based on CYP2D6 Genotype Alone Versus CYP2D6 Genotype Plus CYP2D6 Inhibitor Use for All Randomized Participants eTable 2. Medications at Baseline Causing Phenoconversion eTable 3. Other Secondary and Exploratory Outcomes in CYP2D6 Intermediate and Poor Metabolizers eTable 4. Mixed-Model Analysis of Composite Pain Intensity Score Trends From 10 Days to 6 Months in the Actionable Population eTable 5. SIA Score and PROMIS-43 Subscales at 10 Days by Surgery Type in the Actionable Population [file jamanetwopen-e2558299-s001.pdf]

Supplementary Online Content

Cavallari LH, Myers RA, Chakraborty H, et al. CYP2D6-guided opioid management and postoperative pain control: a randomized clinical trial. *JAMA Netw Open*. 2026;9(2):e2558299. doi:10.1001/jamanetworkopen.2025.58299

eAppendix 1. ADOPT PGx Clinical Groups

eAppendix 2. ADOPT PGx Protocol Implementation Teams

eAppendix 3. ADOPT PGx Site PIs and Site Study Staff

eAppendix 4. Additional Collaborators

eAppendix 5. Participating Health Systems

eAppendix 6. Inclusion and Exclusion Criteria

eAppendix 7. Recruitment

eAppendix 8. Study Procedures

eAppendix 9. Subgroups Analyzed

eAppendix 10. Trial Monitoring Plan

eAppendix 11. Randomization Procedure

eAppendix 12. Supplemental Statistical Methods

eAppendix 13. Opioid Consumption Survey Part 1: Prescribed Opioids

eTable 1. CYP2D6 Phenotype Based on CYP2D6 Genotype Alone Versus CYP2D6 Genotype Plus CYP2D6 Inhibitor Use for All Randomized Participants

eTable 2. Medications at Baseline Causing Phenoconversion

eTable 3. Other Secondary and Exploratory Outcomes in CYP2D6 Intermediate and Poor Metabolizers

eTable 4. Mixed-Model Analysis of Composite Pain Intensity Score Trends From 10 Days to 6 Months in the Actionable Population

eTable 5. SIA Score and PROMIS-43 Subscales at 10 Days by Surgery Type in the Actionable Population

eReferences.

© 2026 Cavallari LH et al. *JAMA Network Open*.

This supplementary material has been provided by the authors to give readers additional information about their work.

© 2026 Cavallari LH et al. *JAMA Network Open*.

eAppendix 1. ADOPT PGx Clinical Groups

Clinical Group Award PI: Duke University Medical Center: Durham, NC: Orlando, L
- Duke University Medical Center, Durham, NC: Voora, D

Clinical Group Award PI: Icahn School of Medicine at Mount Sinai, New York, NY: Horowitz, C
- Icahn School of Medicine at Mount Sinai, New York, NY: Horowitz, C
- The Institute for Family Health, New York, NY: Calman, N

Clinical Group Award PI: Indiana University Health System, Indianapolis, IN: Skaar, T
- Indiana University Health System, Indianapolis, IN: Skaar, T, Dexter, P
- Eskenazi Health, Indianapolis, IN: Skaar, T, Dexter, P

Clinical Group Award PI: University of Florida, Gainesville, FL: Johnson, J, Cavallari, L
- University of Florida, Gainesville, FL: Johnson, J, Cavallari, L
- University of Florida, Jacksonville, FL: Johnson, J, Cavallari, L
- Nemour's Children's Health System, Jacksonville, FL: Blake, K
- Nemour's Children's Health System, Orlando, FL: Blake, K
- Nemour's Children's Health System, Wilmington, DE: Blake, K

Clinical Group Award PI: Vanderbilt University Medical Center, Nashville, TN: Peterson, J
- Vanderbilt University Medical Center, Nashville, TN: Peterson, J, Cavanaugh, K
- Meharry Medical College, Nashville, TN: Singh, R
- Nashville General, Nashville, TN: Singh, R
- Sanford Health, Fargo, ND: Hines, L

IGNITE Coordinating Center: Duke Clinical Research Institute, Durham, NC: Wyatt, C, Chakraborty, H

© 2026 Cavallari LH et al. *JAMA Network Open*.

eAppendix 2. ADOPT PGx Protocol Implementation Teams

IGNITE Coordinating Center: Hrishikesh Chakraborty (PI), Christina Wyatt (PI), Geoff Ginsburg (*f*-PI), Kady-Ann Steen-Burrell, Bhargav Adagarla, Sarah George, Beth Harris, Jaclyn Holland, Yashika Johnson, Phyllis Kennel, Kristen Linney, Rania Metry, Rachel Myers, Wanda Parker, Gayle Passmore, Carol Pereira, and Teji Rakhra-Burris.

Duke University Medical Center: Deepak Voora (PI), Ryanne Wu, Teji Rakhra-Burris, Azita Sadeghpour (PM), Ruth Lehan, and Tyffany Locklear.

Icahn School of Medicine at Mount Sinai: Carol Horowitz (PI), Michelle Ramos (PM), Sabrina Clermont (PM), Bart Ferket, Kenneth Fifer, Diane Hauser, Joseph Kannry, James Murrough, Aniwaa Owusu Obeng, Janet Seo, Tatiana Sabin, Nandini Shroff, and Saskia Shuman.

Indiana University Health System: Todd Skaar (PI), Paul Dexter (PI), Abi Colwell (PM), Amy M. Breman, Zeru Desta, Cathy Fulton, Jennelle C. Hodge, Sheryl Lynch, Jonathan Oliver, Victoria M. Pratt, Ross Robinson, Elizabeth Rowe, Jennifer Stuart, Emma Tillman, and Ashely Vetor.

University of Florida: Julie Johnson (PI), Larisa Cavallari (PI), Alexander Parker (PI), Erica Elwood (PM), Kathryn Blake, Emily Cicali, Kelsey Cook, Karam Diaby, Julio Duarte, Ben Duong, Karla Giron, Elizabeth Eddy, Julia Krutov, Carol Mathews, Haesuk Park, Robyn Nelson, Ryan Rhoden, Caroline Schlierle, Taylor Sullivan, Almut Winterstein, and Kristen Wiisanen.

Vanderbilt University Medical Center: Josh Peterson (PI), Kerri Cavanaugh (PI), Sara Van Driest (*f*-PI), Sara Block (PM), Jordan Baye, Michelle Benck, Chantel Bender, Sylvia Eluhu, Colette Free, Shane Gonnelly, Sarah Hedeen, Lindsay Hines, Steve Houtschilt, Michelle Liu, Salisha Marryshow, Nicole Neville, Natasha Petry, Sidd Pratap, Vernon Sherden, Rajbir Singh, Kimberly Snell, Ana Tomescu, Megan Trietsch, James Walker, Deanna Web, Erin Whiting, and Russell A. Wilke.

National Human Genome Research Institute: Simona Volpi, Sarah Hutchison, Natalie Kucher, Ebony Madden, Jessica Reinach, Renee Rider, Ismail Safi, and Ella Samer.

© 2026 Cavallari LH et al. *JAMA Network Open*.

eAppendix 3. ADOPT PGx Site PIs and Site Study Staff

Duke University Medical Center: Lori Orlando (PI), Deepak Voora (PI), Azita Sadeghpour, Lani Banez, Lisa Bendz, Jackie Jordan, Ruth Lehan, Tyffany Locklear, Ann McGee, Christina Nix, Lorraine Vergara, and Ryanne Wu.

Icahn School of Medicine at Mount Sinai: Carol Horowitz (PI), Michelle Ramos (PM), Sabrina Clermont (PM), Tyler Colon, Aaishah Francis, Samantha Guagliardo, Najiba Khan, Camila Tan Lam, Michelle Sciarrino, Emma Maiman-Stadtmauer, Sofia Medina-Pardo, Tatiana Sabin, Janet Seo, and Ololade (Lola) Williams.

IGNITE Coordinating Center: Hrishikesh Chakraborty (PI), Christina Wyatt (PI), Geoff Ginsburg (*f*-PI), Sheng Luo, Kady-Ann Steen-Burrell, Kevin Anstrom, Bhargav Adagarla, Alicia Ellis, Stephen Ellis, Sarah George, Beth Harris, Jaclyn Holland, Yashika Johnson, Phyllis Kennel, Hwasoon Kim, Kristen Linney, Peter Merrill, Rania Metry, Rachel Myers, Wanda Parker, Gayle Passmore, Carol Pereira, Teji Rakhra-Burris, Lilin She, Jennifer Shepherd, Saira Siddiqui, and Jun Wen.

Indiana University and Eskenazi Health: Todd Skaar (PI), Abi Colwell, Brittany Davis, John Howard, Alia Jamison, Lydia Joyner, Ellie Kiihne, Sheryl Lynch, Johnathan Oliver, Ross Robinson, Libbie Silverman, Jordan Sonneville Jennifer Stuart, Laurel Tokar, and Ashley Vetor.

The Institute for Family Health: Neil Calman (PI), Saskia Shuman, Daviana Buck, Nicole Canchucaja Porshia Cook, Ariel Jacobs Danielle John, Samantha Guagliardo, Diane Hauser, Mirta (Mimi) Milanes, Wambui Ngari, and Nandini Shroff.

Meharry Medical College and Nashville General Hospital: Rajbir Singh (PI), Megan Triestch, Abraham Garcia, Carol Gutierrez, Steven Houtschilt, Maria Lopez, Alan Mejia, Anaite Montes Bu, Deanna Webb, and Sonja White.

Nemours Children's Health: Kathryn Blake (PI), Ben Duong, Taylor Sullivan, Ali Brewer, Kelsey Cook, Brittni Deadrick, and Julia Krutov.

Sanford Health: Lindsay Hines (PI), Jordan Baye (PI), Colette Free, Michelle Benck, Shane Gonnelly, Sarah Hedeen, Alexis Meyer, and Blair Svennes.

University of Florida at Gainesville: Larisa Cavallari (PI), Erica Elwood (PM), Hana Al Alshaykh, Tala Basha, Emily Cicali, Asia Cobb, Brendon Cooper, Elizabeth Eddy, Amanda Elchynski, Amanda Elsey, Anuksha Gotmare, Karla Giron, Lauren Lemke, Arielle Nelson, Robyn Nelson, Michael Read, Ryan Rhoden, Genevieve Rosier, Isabella Schultz, Chiara Chermont Spina, Joshua Terrell, Alexa Valko, and Precious Williams.

University of Florida at Gainesville Call Center: Tracy Leonard, David Smith, Marian Davis, Vincent Guerra, Jennifer Salo, Claudia Cartaya Torres, Jorge Valedon, and Paola Nieves Viana.

University of Florida at Jacksonville: Alexander Parker (PI), Jose Alonso, Jeffery Gainer, Morgan Howard, Fatoumata Kaba, Brianna Rivers, Liliana Serrano, Kimberly Vigal, and ADeia Williams.

Vanderbilt University Medical Center: Josh Peterson (PI), Kerri Cavanaugh (PI), Sara Van Driest (*f*-PI), Sara Block (PM), Chantel Bender, Angelo Lazaro, Salisha Merryshow, Nicole Neville, Henry Ong, and Erin Whiting.

eAppendix 4. Additional Collaborators

Asiyanbola Bolanle, John Thomas Callaghan, Justin Deen, Michael T. Eadon, MD, Daniel Getu, MD, Blake Goff, PharmD, Joseph King, Eric A. Larson, MD, Brandi Plunkett, RN, Luis Pulido, Marc B. Rosenman, Robert Van Demark, Jr, MD, Walter W. Virkus, MD, Thomas Wright, and Jonathan Wright.

© 2026 Cavallari LH et al. *JAMA Network Open*.

eAppendix 5. Participating Health Systems

- University of Florida Health, Gainesville, FL
- Sanford Health, Sioux Falls, SD
- Mount Sinai Health System, New York, NY
- Duke University Health System, Durham, NC
- Indiana University Health, Indianapolis, IN
- Nashville General Hospital, Nashville, TN
- Nemours Children's Health, Jacksonville and Orlando, FL
- Meharry Medical Group affiliated with Meharry Medical College, Nashville, TN

eAppendix 6. Inclusion and Exclusion Criteria

Inclusion Criteria:

- Age ≥ 8 years.
- English-speaking or Spanish-speaking.
- Elective/planned surgery types with planned or anticipated treatment with tramadol, hydrocodone, or codeine pain management at an enrolling site, which may include orthopedic surgeries (e.g., arthroplasty, spine, etc.), open abdominal surgery, or cardiothoracic surgery and others.

Exclusion Criteria:

- Receiving chronic opioid therapy, defined as use of opioids on most days for >3 months.
- Life expectancy less than 12 months.
- Are too cognitively impaired to provide informed consent and/or complete study protocol.
- Are institutionalized or too ill to participate (i.e., mental or nursing home facility or incarcerated).
- Have a history of allogeneic stem cell transplant or liver transplant.
- People with prior clinical pharmacogenetic test results for genes relevant for the study in which they will enroll (CYP2D6 for the pain studies and CYP2D6 or CYP2C19 for depression) or already enrolled in an ADOPT PGx trial.
- Any other medical, behavioral, or developmental condition that in the opinion of the investigator may confound study data/assessments.

eAppendix 7. Recruitment

Potential participants were identified when they presented to the clinic for surgery evaluation and scheduling or through electronic health record review and approached (in person or via telephone call) by a research team member.

eAppendix 8. Study Procedures

A genetic sample was collected from each participant at enrollment via venipuncture, saliva collection, or buccal swab. Samples were processed immediately for patients in the intervention arm, with results returned to the electronic health record (EHR), and after completion of study participation for patients in the control arm. Samples were genotyped for the *CYP2D6*2, *3, *4, *5, *6, *8, *9, *10, *17, *29,* and **41* alleles and copy number variation. Activity scores were assigned for each allele according to Clinical Pharmacogenetics Implementation Consortium (CPIC) guidelines (e.g., 0, 0.25 to 0.5, and 1 for no-function, decreased function, and normal function alleles, respectively), as previously described.[1,2] The total activity score (sum of scores for each allele) was multiplied by 0.5 for patients taking a moderate CYP2D6 inhibitor (e.g., duloxetine) and by 0 for strong inhibitors (e.g., bupropion, fluoxetine, paroxetine). The following phenotypes were assigned based on total activity score: 0, poor metabolizer (PM); >0-0.75, intermediate metabolizer (IM); 1-2, normal metabolizer (NM), and >2, ultra-rapid metabolizer

(UM). It is noted that this definition of IM is different than the current CPIC definition of CYP2D6 IM, where an activity score of 1 is also considered an IM.[3] CPIC added the activity score of 1 to the IM definition after this protocol had been finalized, and study investigators decided to exclude an activity score of 1 from the definition of IM in the trial to be consistent with the IM and NM phenotype assignments used in a pilot trial that suggested improved pain management with CYP2D6-guided postoperative pain management.[4]

Recommendations to avoid tramadol, hydrocodone, and codeine and to prescribe a non-CYP2D6–metabolized opioid (e.g., hydromorphone or morphine) or a non-opioid analgesic in CYP2D6 PMs, IMs, and UMs were provided to prescribers for patients in the intervention arm. Avoidance of oxycodone was also recommended in UMs given the potential risk for toxicity, but oxycodone was considered acceptable for PMs and IMs given inconsistent associations between CYP2D6 phenotype and pain response.[1] Recommendations were provided via automated clinical decision support at all sites and a written consult note placed in the electronic health record by a clinical pharmacist at all but one site. The ultimate prescribing decision was left to the provider. Consult note format and wording varied across sites, but all included the following required elements, per the ADOPT PGx Clinical Decision Support Working Group Guidelines for consult notes:

- Unique title containing, "Pharmacogenomics Consult Note" and "ADOPT-PGx."
- Patient name, MRN.
- Drug allergies.
- Current interacting drugs (i.e., CYP2D6 inhibitors).
- Pharmacogenetic test results (genotype and/or phenotype).
- Interpretation of the pharmacogenetic test results, including any phenoconversion and the predicted clinical outcome (efficacy/toxicity).
- Recommended alternatives.

Required elements of the automated alerts included:

- Statement of genotype, phenotype, impacted drug, and predicted drug response (efficacy/toxicity).
- Additional information on the nature of the drug-gene interaction and recommended alternatives.
- Instructions to discontinue current order and order alternative medication
- Instructions for how to obtain a consult or further information.

Adult participants were called, or if the patient preferred, contacted via an emailed or texted link to a web-survey at 10 (±3) days, and at 1 (±7), 3 (±14), and 6 (±14) months after surgery. Participants were also called by a clinical research assistant from a centralized call center at the 10-day time point for a full medication reconciliation. If the call center personnel could not reach the participant, a local site coordinator attempted to contact the participant. Surveys for pediatric patients were sent to the parent. At each time point, participants were asked to rate their average pain on a 10-point scale, per the Patient-Reported Outcomes Measurement Information System (PROMIS) Numeric Rating Scale, and their worst and average pain in the past 7 days and current pain on a 5-point Likert scale, according to the PROMIS Pain Intensity instrument.[5] At the 10-day and 1-month time points, participants were also asked about type, strength, quantity of opioid prescribed, tablets remaining from their original opioid prescription and any refills or new prescriptions. Opioid usage was not assessed for participants prescribed liquid opioid forms. Participants were also asked at the end of their participation about whether they became aware of their genotype results at any time over the course of the trial.

eAppendix 9. Subgroups Analyzed

1. **Per-Protocol Subgroup**

The per-protocol subgroup consisted of the subset of the actionable population who had their genotype results entered in the medical record prior to surgery for the intervention arm and after surgery for the control arm, and, for the intervention arm, had therapy that was concordant with recommendations (i.e., were not prescribed tramadol, hydrocodone, or codeine).

2. **Non-Oxycodone Subgroup**

The non-oxycodone subgroup consisted of the per-protocol population (described above), with the exclusion of participants prescribed oxycodone from the intervention arm. Specifically, for the immediate arm, the non-oxycodone population consisted of the subset of the actionable population who had their *CYP2D6* test result returned prior to surgery, did not have a conflict between their CYP2D6 metabolizer phenotype and the pain medication(s) prescribed for post-surgery pain, and were not prescribed oxycodone for post-surgery pain management. Participants in the control arm consisted of the subset of the actionable population who had their *CYP2D6* test result returned after surgery.

3. Non-Actionable Subgroup

The non-actionable subgroup was defined as the population who completed surgery and who did not have an IM or PM phenotype.

eAppendix 10. Trial Monitoring Plan

Monitoring was a critical component of this study to ensure data integrity, regulatory compliance, and adherence to the study protocol. The Clinical Monitoring Plan (CMP) outlined operational guidelines and responsibilities for the Duke Clinical Research Institute (DCRI), Clinical Groups (CGs), Clinical Research Associate (CRA), and the Lead Clinical Monitor. All monitoring activities were conducted remotely. The CMP was established prior to enrollment of the first study participant.

1. Central Monitoring Procedures

The DCRI provided high-level oversight, managing regulatory submissions and Institutional Review Board (IRB) approvals, site activation, and study closeout. The DCRI provided regulatory document templates and checklists, created the electronic Trial Master File (eTMF) structure, and provided eTMF training. The DCRI CRA held check-ins with the CGs 1-2 times per month. The monitor ensured all essential documents were filed in the eTMF, maintained regular communication with CGs, monitored the central database, resolved queries, and oversaw site closeout. The DCRI CRA and monitor reviewed and approved communication plans developed by the CGs to guide interactions with relying sites.

2. Site-Level Monitoring Processes

Site monitoring included structured check-ins and scheduled monitoring visits. The monitor conducted monitoring visits with the CGs at specified times. Three types of monitoring visits were conducted:

1. Site Initiation Visit (SIV): Conducted before participant enrollment, providing training and regulatory document review.
2. Periodic Monitoring Visits (PMVs): Conducted based on specific triggers to ensure ongoing compliance and data integrity.
3. Close-Out Visit: Performed at study completion to verify final documentation and regulatory compliance.

PMVs were triggered based on enrollment milestones:

- Phase 1: Enrollment of the first 5 participants in one study arm.
- Phase 2: Enrollment of 5 participants in another arm or 30 participants total.
- Phase 3: Enrollment of 5 participants in the last unmonitored arm or every 2 months.
- Ongoing monitoring: Conducted every 2 months throughout the study.

During monitoring visits, key study components were reviewed to ensure compliance and data accuracy. Participant enrollment was assessed to ensure screening and enrollment aligned with study expectations. Protocol compliance was evaluated by confirming that the study teams followed the study plan and verifying that any protocol deviations were properly documented and reported. The informed consent process was reviewed to ensure that consent was obtained and documented correctly and in accordance with regulatory requirements. The Study Staff Delegation and Signature Log (SSDSL) was checked to ensure that the principal investigator (PI) maintained

an up-to-date record of staff and delegated duties. Electronic Case Report Forms (eCRFs) were checked for accuracy and completeness and reportable events and unanticipated participant deaths were assessed for timely reporting. Monitoring also included a review of outstanding action items from previous visits and ensured that all research staff had undergone protocol training and current Human Subjects Protection and Good Clinical Practice training. Additionally, at each CG monitoring visit, a 5% random sample of participants' laboratory results was reviewed for accuracy. If discrepancies exceeded 25% of the sample, the review expanded to 10% for that site. If >25% discrepancies persisted, the issue was escalated to the Coordinating Center (CC) for corrective action.

As part of the final data cleaning, the statistics team conducted a comprehensive review of study data to identify missing data, outliers, and other discrepancies. Reports of these findings were generated and the monitor worked closely with the CGs to resolve outstanding data issues prior to database lock. This process helped ensure the completeness and accuracy of the final study data.

4. University of Florida College of Pharmacy Call Center Monitoring

Call Center monitoring was part of central oversight. Call Center staff training was assessed during the University of Florida CG PMVs. Every 3 months, the statistical team generated reports to evaluate the completion of follow-up assessments conducted by the Call Center. These reports included a 5% subsample of completed participant visits and follow-up assessments by the Call Center coordinator and a list of visits where active participants exited the visit window without follow-up data.

5. Manual of Procedures (MOP)

The Manual of Procedures (MOP) served as a reference document for the study staff. The DCRI provided a template to the CGs to make site-specific modifications. The MOP summarized the CMP and provided guidance regarding reportable events. It included job aids guiding research staff through key activities, such as the informed consent process, eCRF completion, sample collection, participant follow-up, results interpretation, and return of results. By standardizing processes, the MOP ensured protocol adherence and consistency across sites.

eAppendix 11. Randomization Procedure

Randomization was stratified by site and a random block size within each site. The unblinded statistician generated the randomization allocation sequence. The REDCap Randomization Module was used to store and conceal the pre-specified randomization allocations. Randomization allocations were concealed until research coordinators confirmed that all pre-randomization activities were complete and clicked the randomization button in the REDCap database.

eAppendix 12. Supplemental Statistical Methods

1. Opioid Use Derivation

Opioid prescriptions for post-surgery pain management based on chart review were recorded in the study database. For each of the recorded prescriptions, an 8-question pill count survey was administered and repeated up to 3 times per refill. This survey was developed for a previous pilot trial.[4] Opioid-specific pill counts were derived from the responses to the opioid medication pill count survey (see Opioid Consumption Survey Part 1: Prescribed Opioids below), tabulating the number of pills consumed based on the reported number of pills dispensed per bottle, number of pills dropped or taken by someone else, and number of pills remaining in each bottle. Per opioid pill counts were converted to total mg consumed using the recorded pill strength and converted into milligram morphine equivalents (MMEs). Finally, cumulative MMEs were summed across all opioids per person. Due to variability in the number of days between discharge and when the survey was completed, cumulative MMEs were converted to average daily MMEs, adjusting for the number of days between discharge and survey date.

2. SIA Score Derivation

© 2026 Cavallari LH et al. *JAMA Network Open*.

SIA score was derived as previously described[6] in two analytical populations: 1) the completed surgery population and 2) the actionable population. Within a given analytical population, opioid use (average MME/day) was ranked, allowing for ties and similarly, the numeric pain rating was ranked (allowing for ties). Ranks were rescaled to be centered at 0 and ranged from a theoretical minimum of -100 to +100. The rescaled ranks are summed to form the composite SIA score, with a theoretical minimum of -200, representing the lowest level of pain and lowest level of opioid use, and a theoretical maximum of 200, representing the highest level of pain and opioid use.

3. Imputation

Multiple imputation was conducted using the SAS proc mi fully conditional specification (FCS) regression method. Baseline characteristics, including age, race, surgery type, depression score, and 10-day pain and average daily MMEs (i.e., opioid use) were used to construct and update the FCS regression models. Overall, 20 imputed datasets were generated to account for variability in this process. For each imputed dataset, the SIA score was recalculated using the imputed pain and opioid usage values. Each imputed dataset was analyzed separately, and the results were pooled using Rubin's rules.

© 2026 Cavallari LH et al. *JAMA Network Open*.

eAppendix 13. Opioid Consumption Survey Part 1: Prescribed Opioids

Instructions: We would like to document your pain medicine use since surgery. Do you have your pain medicine pill bottle(s) with you where you can count how many pills are left?

1. It looks like you were given [medicine] for pain after surgery, did you pick that up from the pharmacy?
1. Yes
2. No **(GO to next medicine question or #9)**

2. How many [medicine] pills were given to you in your pill bottle at the pharmacy? (Check pill bottle label)
1. _____________(integer ≥ 1)

3. Did you take all of the [medicine] pills?
1. Yes **(GO TO Q5)**
2. No

4. How many [medicine] pills are left in your bottle?
1. ________

5. Were any of the [medicine] pills dropped or taken by someone else?
1. Yes
2. No **(GO TO Q7)**

6. How many [medicine] pills were dropped or taken by someone other than yourself?
1. ______ (integer ≥ 1)

7. Did you get a refill or new prescription for [medicine] from your provider?
1. Yes
2. No **(GO TO next medicine or #9)**

8. Did you pick up that refill of [medicine] from the pharmacy?
1. Yes **(REPEAT Qs 2-6 questions)**
2. No **(GO TO next medicine or Q9)**

© 2026 Cavallari LH et al. *JAMA Network Open*.

eTable 1. CYP2D6 Phenotype Based on *CYP2D6* Genotype Alone Versus *CYP2D6* Genotype Plus CYP2D6 Inhibitor Use for All Randomized Participants

	**All Participants[a] (n=1419)**	**CYP2D6-Guided Arm (n=719)**	**Control Arm (n=700)**
Actionable phenotype[b], No (%)	351/1415 (25)	176/716 (25)	175/699 (25)
Phenotype based on genotype alone, No (%)			
Poor metabolizer	85/1419 (6)	48/719 (7)	37/700 (5)
Intermediate metabolizer	116/1419 (8)	51/719 (7)	65/700 (9)
Normal metabolizer	1146/1419 (81)	580/719 (81)	566/700 (81)
Ultra-rapid metabolizer	39/1419 (3)	22/719 (3)	17/700 (2)
Other[c]	29/1419 (2)	15/719 (2)	14/700 (2)
Indeterminant	4/1419 (<1)	3/719 (<1)	1/700 (<1)
Phenotype based on genotype + drug interactions[d], No (%)			
Poor metabolizer	199/1419 (14)	105/719 (15)	94/700 (13)
Intermediate metabolizer	146/1419 (10)	68/719 (9)	78/700 (11)
Normal metabolizer	1009/1419 (71)	511/719 (71)	498/700 (71)
Ultra-rapid metabolizer	36/1419 (3)	20/719 (3)	16/700 (2)
Other[c]	22/1419 (2)	10/719 (1)	12/700 (2)
Indeterminant	7/1419 (<1)	5/719 (<1)	2/700 (<1)

[a]Data are reported for 1419 participants who had their genotype resulted and completed surgery. The percentage of participants with an actionable phenotype increased from 14% to 24% after phenoconversion was considered.
[b]Includes poor metabolizers, intermediate metabolizers, and three with an indeterminant phenotype with a specified lower-bound activity score, after accounting for phenoconversion.
[c]Ranged phenotypes, including intermediate metabolizer to normal metabolizer, intermediate metabolizer to ultra-rapid metabolizer, and normal metabolizer to ultra-rapid metabolizer.
[d]Medications causing phenoconversion were bupropion (n=77), fluoxetine (n=33), paroxetine (n=15), and terbinafine (n=4) for strong inhibitors and duloxetine (n=86) and mirabegron (n=15) for moderate inhibitors.

© 2026 Cavallari LH et al. *JAMA Network Open*.

eTable 2. Medications at Baseline Causing Phenoconversion

	All Participants with an Actionable Phenotype (n=351)	CYP2D6-Guided Arm (n=176)	Control Arm (n=175)	*P*-value[a]
Strong CYP2D6 inhibitors, No (%)				
Fluoxetine	33/351 (9)	17/176 (10)	16/175 (9)	0.868
Paroxetine	15/351 (4)	9/176 (5)	6/175 (3)	0.435
Bupropion	76/351 (22)	35/176 (20)	41/175 (23)	0.421
Terbinafine	4/351 (1)	4/176 (2)	0/175 (0)	0.123
Moderate CYP2C6 inhibitors, No (%)				
Duloxetine	59/351 (17)	31/176 (18)	28/175 (16)	0.686
Mirabegron	9/351 (3)	5/176 (3)	4/175 (2)	1.000

[a]*P*-values are for comparisons between the intervention and control arms based on a chi-squared test or Fisher's exact test (if any of the expected cell count was less than 5) for categorical variables.

© 2026 Cavallari LH et al. *JAMA Network Open*.

eTable 3. Other Secondary and Exploratory Outcomes in CYP2D6 Intermediate and Poor Metabolizers

	CYP2D6-Guided Arm (n=176)	Control Arm (n=175)	*P*-value
SIA score with composite pain intensity at 10 days[a], mean ± SD	-1.6 ± 94.4 (n=160)	1.7 ± 96.4 (n=152)	0.759
Numeric pain intensity at 1 month, mean ± SD	3.7 ± 2.0 (n=165)	3.8 ± 2.6 (n=163)	0.505
Composite pain intensity at 1 month[b], mean ± SD	7.4 ± 2.3 (n=165)	7.4 ± 2.3 (n=164)	0.843
Opioid usage at 1 month (MME/day), mean ± SD	7.1 ± 7.6 (n=163)	6.9 ± 8.0 (n=160)	0.646
Overall well-being domains at 1 month, mean ± SD			
Pain interference	59.0 ± 8.4 (n=165)	58.3 ± 9.0 (n=162)	0.533
Physical function	34.4 ± 7.8 (n=165)	35.3 ± 8.0 (n=162)	0.347
Depression	47.4 ± 9.6 (n=165)	45.6 ± 8.9 (n=162)	0.092
Anxiety	47.8 ± 10.0 (n=165)	48.1 ± 9.6 (n=162)	0.730
Fatigue	51.3 ± 8.2 (n=165)	50.5 ± 9.9 (n=162)	0.610
Sleep disturbance	53.9 ± 8.6 (n=165)	52.8 ± 9.7 (n=162)	0.234
Satisfaction with participation in social roles	44.7 ± 9.6 (n=165)	43.9 ± 9.8 (n=162)	0.236

SIA, 10-day Silverman Integrated Analgesic Assessment.
[a]SIA score derived from the composite pain intensity and prescribed opioid usage within the actionable population.
[b]Composite of worst and average pain over the past 7 days and current pain, each on a 5-point scale, with the total ranging from 3-15.

© 2026 Cavallari LH et al. *JAMA Network Open*.

eTable 4. Mixed-Model Analysis of Composite Pain Intensity Score Trends From 10 Days to 6 Months in the Actionable Population

Time Point	Genotype-Guided Arm (n=176)	Control Arm (n=175)	*P*-value
10 Day	9.0 (8.6-9.4, n=168)	9.1 (8.7-9.5, n=160)	0.943
Month 1	7.4 (7.0-7.8, n=165)	7.4 (7.0-7.8, n=164)	
Month 3	6.1 (5.7-6.4, n=160)	6.1 (5.7-6.5, n=159)	
Month 6	5.8 (5.5-6.2, n=162)	5.7 (5.4-6.1, n=158)	

Data are presented as the least squares mean estimates (95% confidence interval, n), estimated using a linear mixed effect model adjusted for repeated measures per participant. *P*-value is the ANOVA-F test for the overall treatment effect.

© 2026 Cavallari LH et al. *JAMA Network Open*.

eTable 5. SIA Score and PROMIS-43 Subscales at 10 Days by Surgery Type in the Actionable Population

	Total Knee Arthroplasty			Total Hip Arthroplasty		
	**CYP2D6-Guided Arm**	**Control Arm**	***P*-value**	**CYP2D6-Guided Arm**	**Control Arm**	***P*-value**
SIA score, mean ± SD (number of patients)	17.1 ± 92.1 (n=81)	12.2 ± 94.4 (n=80)	0.738	-11.2 ± 94.2 (n=42)	3.0 ± 83.0 (n=42)	0.478
PROMIS-43 domains, mean ± SD (number of patients)						
Pain interference	61.2 ± 7.2 (n=83)	60.0 ± 8.5 (n=85)	0.378	56.8 ± 9.2 (n=47)	57.8 ± 9.2 (n=45)	0.722
Physical function	32.2 ± 6.2 (n=83)	34.6 ± 6.9 (n=85)	0.032	34.9 ± 8.5 (n=47)	33.8 ± 7.8 (n=45)	0.631
Depression	46.5 ± 9.8 (n=83)	45.2 ± 8.7 (n=85)	0.436	48.4 ± 9.2 (n=47)	46.4 ± 9.6 (n=45)	0.243
Anxiety	47.3 ± 9.9 (n=83)	47.7 ± 9.3 (n=84)	0.690	47.9 ± 10.6 (n=47)	48.8 ± 10.5 (n=45)	0.751
Fatigue	50.9 ± 8.1 (n=83)	51.4 ± 9.5 (n=85)	0.722	52.1 ± 8.3 (n=47)	50.4 ± 10.3 (n=45)	0.588
Sleep disturbance	54.9 ± 8.4 (n=83)	54.2 ± 9.3 (n=84)	0.528	52.5 ± 9.0 (n=47)	53.2 ± 10.3 (n=44)	0.863
Satisfaction with participation in social roles	43.4 ± 9.6 (n=83)	43.7 ± 9.5 (n=84)	0.668	44.8 ± 9.0 (n=47)	41.5 ± 9.4 (n=45)	0.090

PROMIS, Patient-Reported Outcomes Measurement Information System; SIA, 10-day Silverman Integrated Analgesic Assessment.

© 2026 Cavallari LH et al. *JAMA Network Open*.

eReferences.

1. Crews KR, Monte AA, Huddart R, et al. Clinical pharmacogenetics implementation consortium guideline for CYP2D6, OPRM1, and COMT genotypes and select opioid therapy. *Clin Pharmacol Ther*. 2021;110(4):888–896. doi: 10.1002/cpt.2149
2. Cavallari LH, Cicali E, Wiisanen K, et al. Implementing a pragmatic clinical trial to tailor opioids for acute pain on behalf of the IGNITE ADOPT PGx investigators. *Clin Transl Sci*. 2022;15(10):2479–2492. doi:10.1111/cts.13376
3. Caudle KE, Sangkuhl K, Whirl-Carrillo M, et al. Standardizing CYP2D6 Genotype to Phenotype Translation: Consensus Recommendations from the Clinical Pharmacogenetics Implementation Consortium and Dutch Pharmacogenetics Working Group. *Clin Transl Sci*. 2020;13(1):116–124. doi:10.1111/cts.12692
4. Thomas CD, Parvataneni HK, Gray CF, et al. A hybrid implementation-effectiveness randomized trial of CYP2D6-guided postoperative pain management. *Genet Med*. 2021;23(4):621–628. doi:10.1038/s41436-020-01050-4
5. Patient Reported Outcomes Measurement Information System (PROMIS) HealthMeasures. https://www.healthmeasures.net/explore-measurement-systems/promis. Accessed March 20, 2024.
6. Dai F, Silverman DG, Chelly JE, Li J, Belfer I, Qin L. Integration of pain score and morphine consumption in analgesic clinical studies. *J Pain*. 2013;14(8):767–77 e8. doi:10.1016/j.jpain.2013.04.004

© 2026 Cavallari LH et al. *JAMA Network Open*.
