# Supplementary material for: CYP2D6-Guided Opioid Management and Postoperative Pain Control: A Randomized Clinical Trial
Source: JAMA Netw Open. 2026 Feb 20;9(2):e2558299. doi: 10.1001/jamanetworkopen.2025.58299 (PMC12924106; doi:10.1001/jamanetworkopen.2025.58299)
Supplement: Supplement 2. — Trial Protocol and Statistical Analysis Plan [file jamanetwopen-e2558299-s002.pdf]

Supplement 2. Trial Protocol and Statistical Analysis Plan

Original Trial Protocol	2
Final Trial Protocol	67
Summary of Changes	68
Original Statistical Analysis Plan	139
Final Statistical Analysis Plan	160
Summary of Changes	161

A Depression and Opioid Pragmatic Trial in Pharmacogenetics

(ADOPT PGx)

Protocol Number: < Number>

Principal Investigators:

Julie A. Johnson, Pharm. D.

Josh F. Peterson, MD, MPH

Todd C. Skaar, Ph.D.

Paul R. Dexter, MD

Statistical Investigator: Hrishikesh Chakraborty, DrPH

NIH Program Officer: Simona Volpi, PhD

Funded by: National Human Genome Research Institute

Version Number: Final v2
11 February 2020

PROTOCOL VERSION AND AMENDMENT TRACKING

Version Number/ Affected Section(s)	Summary of Revisions Made	Approval Date

PROTOCOL APPROVAL PAGE

INVESTIGATOR STATEMENT

By signing below, I agree to the conditions relating to this trial as set out in this protocol (XX dated XX).

I agree to conduct this clinical trial according to Good Clinical Practice (ICH GCP) and European Regulatory Requirements.

I fully understand that any changes instituted by me without previous discussion with the IGNITE PTN Coordinating Center or their designated representative constitute a violation of the protocol.

I agree to adhere to the protocol in all circumstances other than where necessary to protect the well-being of the subject.

Principal Investigators' Signatures

Name:_________________________________

Signature:____
Date:________________________

Name:_________________________________

Signature:____
Date:________________________

Name:_________________________________

Signature:____
Date:________________________

Name:_________________________________

Signature:____
Date:________________________

ABBREVIATIONS

ADOPT PGx	A Depression and Opioid Pragmatic Trial in Pharmacogenetics
AE	Adverse event
CC	Coordinating center
CDS	Clinical decision support
CLIA	Clinical Laboratory Improvement Amendments
CMP	Clinical Monitoring Plan
CMS	Centers for Medicare and Medicaid Services
CPIC	Clinical Pharmacogenetics Implementation Consortium
CRF	Case Report Form
DCRI	Duke Clinical Research Institute
DEA	Drug Enforcement Administration
DoB	Date of birth
DSMB	Data Safety Monitoring Board
EHR	Electronic Health Record
HIPAA	Health Insurance Portability and Accountability Act of 1996
ICD-10	The International Classification of Diseases, 10th Revision, Procedure Coding System
IGNITE PTN	Implementing Genomics in Practice Pragmatic Trials Network
IM	Intermediate metabolizer
LOS	Length of stay
M/M	Medicare/Medicaid
MED	Morphine Equivalent Doses
MME	Morphine Milligram Equivalents
MOP	Manual of Operations
NM	Normal metabolizer
PGx	Pharmacogenetics
PI	Principle investigator

PM	Poor metabolizer
PROMIS	Patient Reported Outcome Measurement System
QIDS	Quick Inventory of Depressive Symptomatology
RM	Rapid metabolizer
ROR	Return of results
SAE	Serious adverse event
SAP	Statistical analysis plan
SIA Score	Silverman Integrated Analgesic Score
SOP	Standard operating procedure
SSN	Social security number
SSRI	Selective serotonin reuptake inhibitors
UM	Ultra-rapid metabolizer

TABLES

Table 1. Genes to be tested and drugs with CPIC recommendations 28
Table 2. CYP2D6 allele to activity score 29
Table 3. CYP2D6 phenotype by Activity Score 29
Table 4. CYP2C19 phenotype by genotype 29
Table 5. CDS summary for acute and chronic pain treatments 30
Table 6. CDS summary for antidepressant medications 31
Table 7. PGx panel genes and drugs with CPIC recommendations 32
Table 8. Data collection schedule 38
Table 9. Sample size calculations 45

FIGURES

Figure 1. ADOPT PGx Trial Design 21

TABLE OF CONTENTS

1. PROTOCOL SYNOPSIS 10
2. INTRODUCTION 17
   2.1 Background, Significance, and Rationale 17
   2.2 Aims and Objectives of the Study 19
3. ENDPOINTS 20
   3.1 Primary Endpoint(s) 20
   3.2 Secondary Endpoints 20
4. STUDY ARMS & DESIGN 21
   4.1 Randomization 22
   4.2 Blinding 22
5. STUDY POPULATION 23
   5.1 Inclusion Criteria 23
   5.2 Exclusion Criteria 23
6. RECRUITMENT AND ENROLLMENT PROCEDURES 25
   6.1 Provider Recruitment and Assent 25
   6.2 Participant Consent Process 25
   6.3 Participant Discontinuation/Withdrawal from the Study 25
   6.4 Lost to Follow-Up 26
   6.5 Risk 26
   6.6 Benefit 27
   6.7 Costs to the Participants 27
   6.8 Compensation to Participants 27
7. STUDY PROCEDURES 28
   7.1 Interventions/Treatments 28
   7.2 Baseline Participant Assessments 32
   7.3 Follow-up Participant Assessments 32
   7.4 Specimen Collection 36
   7.5 Specimen Transfer and Genetic Testing Procedures 37
   7.6 Return of Results 37
   7.7 Clinical Decision Support Systems 37

7.8 Data Collection from the Electronic Health Record 37
7.9 Data Collection from CMS and State Medicaid Agencies 38
8. SAFETY ASSESSMENT AND MONITORING 40
8.1 Medication Side Effects 40
8.2 Events of Interest 40
8.3 Data Safety Monitoring Board 40
8.4 Early Termination and Participant Discontinuation 40
9. MEDICARE AND MEDICAID CLAIMS DATA COLLECTION AND ANALYSIS 42
9.1 Rationale 42
9.2 Data Collection 42
10. STATISTICAL ANALYSIS PLAN AND SAMPLE SIZE 44
10.1 Sample Size Determination 44
10.2 General Statistical Methods 46
10.3 Population for Analyses 46
10.4 Analysis of the Primary Endpoint 46
10.5 Analysis of the Secondary Endpoints 47
10.6 Other Planned Analyses Sub-groups that will be considered: 48
10.7 Interim Analyses 50
10.8 Handling of Missing Data 51
10.9 Multiplicity 51
11. DATA MANAGEMENT 53
11.1 Data Entry and Record Keeping 53
11.2 Data Element Definitions 53
11.3 Database Management and Quality Control of Data 54
11.4 Database Lock and Study Close Out 55
11.5 Data Sharing 55
12. ETHICAL AND HUMAN SUBJECTS CONSIDERATIONS 56
12.1 Institutional Review Board/Ethics Committee Review 56
12.2 Use and Disclosure of Protected Health Information (HIPAA) 56
12.3 Confidentiality and Privacy 56
12.4 Publication and Data Sharing Policies 56
13. PROTOCOL DEVIATIONS AND VIOLATIONS 58
14. APPENDICES 62

14.1 APPENDIX A. SIA SCORE BACKGROUND AND RATIONALE 62
14.2 APPENDIX B. TABLE OF MINIMUM REQUIRED VARIANTS 64
14.3 APPENDIX C. CYP2D6 INHIBITORS AS DEFINED BY THE FDA GUIDENCE ON DRUG INTERACTIONS 65

1. PROTOCOL SYNOPSIS

**Protocol Title**	A Depression and Opioid Pragmatic Trial in Pharmacogenetics **(ADOPT-PGx)**
**US IND Number**	
**Grant Number**	
**Product/Intervention**	Immediate vs. delayed pharmacogenetic testing and genotype-guided pain or depression therapy
**Objectives**	Acute Pain:<br>● To determine the effect of genotype-guided therapy on pain control and use of DEA schedule II opioids in post-surgical participants<br><br>Chronic Pain:<br>● To determine the effect of genotype-guided therapy on pain control in chronic pain participants<br><br>Depression:<br>● To determine the effect of genotype-guided selection and dosing of antidepressants on control of depression in participants with ≥3 months of depressive symptoms who require new or revised therapy<br><br>All Trials:<br>● To determine the effect of genotype-guided therapy for opioids, antidepressants, and optional additional, CPIC gene-drug pairs on overall well-being<br>● To determine the effect of genotype-guided therapy for opioids, antidepressants, and optional additional, CPIC gene-drug pairs on healthcare utilization
**Study Design**	ADOPT PGx is comprised of three separate trials, Acute Pain, Chronic Pain, and Depression. Each trial is a prospective, multicenter, two arm randomized pragmatic trial. For all trials, participants will be randomized in a 1:1 ratio to immediate pharmacogenetic testing and genotype-guided therapy (Intervention arm) or 6-month delayed testing on participants receiving standard care (Control arm). In each trial, the primary outcome comparison will be between the intervention group (i.e. immediate testing) and control group (i.e. delayed testing) in the subset of participants with actionable phenotypes, within each study.
**Rationale for Study Design**	Pain and depression are conditions that impact substantial proportions of the US population and have challenges associated with identifying the right therapy while minimizing adverse effects or opioid addiction. There is evidence that both opioid and antidepressant prescriptions can be guided by pharmacogenetics (PGx) data based on existing guidelines from the Clinical Pharmacogenetics Implementation

	Consortium (CPIC). Using such an approach, a recent single site Pragmatic Clinical Trial (PCT) demonstrated that among CYP2D6 poor (PM) and intermediate metabolizers (IM), there was greater improvement in pain control in the genotype-guided arm compared to usual care arm[1]. Similarly, existing studies of PGx tailored antidepressant therapy suggest that the genotype-guided approach is superior to usual care in remission and/or response rates, however these studies are small and often industry-sponsored.<br><br>A broader trial is needed to determine the importance of PGx testing and genotype guided therapy for improving symptom management (i.e. pain control or control of depression symptoms), DEA schedule II opioid use, well-being, and overall healthcare utilization.
**Study Population**	**Acute Pain**<br>Randomized Population<br>2004 participants with planned/elective surgery who are anticipated to start a pain control medication with existing CPIC guidelines after their surgery<br><br>Analytical Population<br>Approximately 302 participants from the randomized population who have an actionable phenotype defined as CYP2D6 IM or PM (i.e. CYP2D6 activity score ≤ 0.75)<br><br>**Chronic Pain**<br>Randomized Population<br>993 participants see in primary care or pain specialty clinics who are already prescribed or anticipated to be prescribed tramadol, codeine, or hydrocodone for pain control.<br><br>Analytical Population<br>Approximately 268 participants from the randomized population who have an actionable phenotype defined as CYP2D6 IM or PM (i.e. CYP2D6 activity score ≤0.75)<br><br>**Depression**<br>Randomized Population<br>1512 participants seen in primary care or psychiatry clinics who are already prescribed or anticipate to be prescribed an SSRI with existing CPIC guidelines<br><br>Analytical Population<br>Approximately 542 participants from the randomized population who have an actionable phenotype, defined as a CYP2D6 PM or ultra-rapid metabolizer (UM), or a CYP2C19 PM, rapid metabolizer (RM), or UM
**Number of Sites**	11 sites with approximately 60-80 clinics

**Duration of Subject Participation:**	Up to one year from consent to end of follow-up
**Description of implementation of intervention (e.g., dose, schedule, etc.)**	The intervention for ADOPT PGx is the immediate return of PGx testing results to the participant's healthcare provider.<br><br>The PGx testing is comprised of Clinical Laboratory Improvement Amendments (CLIA) validated, panel-based genetic testing of two required genes, *CYP2D6* and *CYP2C19.* The resulting *CYP2D6* genotypes will be converted to enzymatic activity scores and the activity scores converted to metabolizer phenotypes after also taking strong and moderate CYP2D6 enzyme inhibitor drug interactions into account. The *CYP219* genotypes will be converted to metabolizer phenotypes.<br><br>The results of the PGx test will be returned to the healthcare providers using standard site-specific laboratory return of results methods, a static report with both interpretation and recommendations, and where possible, interruptive clinical decision support (CDS) alerts within the electronic healthcare system. Both the static report and interruptive alerts will guide health care providers towards medications best suited to the participant.
**Inclusion/Exclusion Criteria**	<u>Inclusion Criteria:</u><br>**Acute Pain**<br>● Age ≥ 8 years (pediatrics), as this is the minimum age at which proposedoutcome measures (PROMIS) are validated without parent proxy<br>● English speaking or Spanish speaking<br>● Elective/planned surgery patients with an upcoming pre-surgery visit<br>● Elective/planned surgery types with pain management, which may include orthopedic surgeries (e.g. arthroplasty, spine, etc.), open abdominal surgery, or cardiothoracic surgery and others<br><br>**Chronic Pain**<br>● Age ≥ 18 years<br>● English speaking or Spanish speaking<br>● Seen at primary care clinics (such as, but not limited to, Internal Medicine, Family Medicine or Pediatrics) or patients seen in relevant specialty clinics<br>● Under the care of a physician for pain for at least 3 months and currently or being considered to be treated with tramadol, hydrocodone, or codeine to improve pain management<br>o Pain diagnosis<br>o Underlying conditions with chronic pain if a pain diagnosis does not exist (not a comprehensive list): arthritis, neuropathy, fibromyalgia, headache.<br>o Have a current or planned pain prescription: tramadol, hydrocodone, or codeine

	**Depression**<br>• Age ≥ 8 years, as this is the minimum age at which proposed outcome measures (PROMIS) are validated without parent proxy<br>• English speaking or Spanish speaking<br>• Patients seen at primary care clinics (such as, but not limited to, Internal Medicine, Family Medicine, or Pediatrics) or in psychiatry clinics<br>• Diagnosis of depression, defined as problem list entry or ICD-10 diagnosis code with at least 3 months of symptoms<br>• Anticipate need for revised or new SSRI therapy for depression<br><br><u>Exclusion Criteria</u><br>**Trial-wide:**<br>• Life expectancy less than 12 months<br>• Are too cognitively impaired to provide informed consent and/or complete study protocol<br>• Are institutionalized or too ill to participate (i.e. mental or nursing home facility)<br>• Plan to move out of the area within 6 months of enrollment<br>• Have a history of allogeneic stem cell transplant or liver transplant<br>• People with prior clinical pharmacogenetic test results for genes relevant for the study in which they will enroll (*CYP2D6* for the pain studies and *CYP2D6* or *CYP2C19* for depression) or already enrolled in an ADOPT PGx trial<br><br>**Acute Pain**<br>• Adults with a similar, previous surgery in whom pain control is well-defined and a genotype-guided approach would not likely be followed<br>• Undergoing a laparoscopic surgery<br>• Receiving chronic opioid therapy, defined as use of opioids on most days for >3 months<br><br>**Chronic Pain**<br>• Undergoing treatment for an active cancer diagnosis<br><br>**Depression**<br>• Have active psychosis or diagnosed psychotic disorders (schizophrenia, schizoaffective disorder, delusional disorder, psychotic depression, substance induced psychosis, schizophreniform disorder)<br>o ICD10: F06.2, F20.x, F25.x, F22, F20.81<br>o Or a problem list entry indicating the diagnosis of one of these disorders<br>• Have dementia or other neurocognitive disorders (due to any cause, such as Alzheimer's disease, vascular/subcortical, lewy body, frontotemporal lobar degeneration)<br>o ICD10: F01, F03.x, G31.83, G30.x, G31.09<br>o Or a problem list entry indicating the diagnosis of one of these disorders<br>• Have cognitive developmental delay and/or cognitive disability, including autism spectrum disorders (Note: this list does not include ADHD)<br>o ICD10: F80.x, F81.x, F82.x, F83.x, F84.x, F85.x, F88.x, F89.x

	o Or a problem list entry indicating the diagnosis of one of these disorders<br>● Have seizures, or who are on a neuroleptic medication<br>o ICD10: G40.x, G41.x<br>o Medications: aripiprazole, asenapine, cariprazine, clozapine, lurasidone, olanzapine, quetiapine, risperidone<br>o Or a problem list entry indicating the diagnosis of one of these disorders<br>● Have bipolar disorder<br>o IDC10: F31.x<br>o Or a problem list entry indicating the diagnosis of one of these disorders<br>● Depression secondary to substance abuse disorder or general medical condition
**Primary Endpoint**	**Acute Pain**: Silverman Integrated Analgesic Assessment (SIA) score (a compositeof pain and opioid usage) at 10 days post-surgery in participants who are genetically or pheno-converted CYP2D6 IM or PM<br><br>**Chronic Pain**: Pain control, defined as change in the composite pain intensity scorefrom baseline to 3-months in participants who are genetically or pheno-converted CYP2D6 IM or PM<br><br>**Depression**: Depression symptom control, defined as change in PROMIS depression 8A scores from baseline to 3-months in genetically or pheno-converted CYP2D6 UM/PM or CYP2C19 UM/RM/PM
**Secondary Endpoints**	**Acute Pain**:<br>● Pain intensity at 10 days post-surgery<br>● Opioid usage at 10 days post-surgery<br>● Prescription pain medication misuse score 3 months post-surgery<br>● Mobility score 1-month post-surgery<br>● Opioid persistence 6 months post-surgery<br><br>**Chronic Pain**:<br>● Pain reduction magnitude at 3-month follow-up, relative to baseline<br>● Achievement of clinically significant pain reduction (30%) by 3-month follow-up, relative to baseline<br>● Prescription pain medication misuse score at 3-month follow-up<br><br>**Depression**:<br>● Change in QIDS scores between baseline and 3 months<br>● Medication side effects severity burden at 3 months<br>● Participant medication adherence at 3 months<br>● Remission at 6 months<br><br>**All Trials**:<br>● Overall well-being at 6 months in all randomized participants<br>● Concordance between metabolizer phenotype and prescribed medication

	• Sub-domains of the PROMIS 43 survey: pain interference, physical function, sleep disturbance, social role and activities functioning, fatigue, anxiety, and depression at 6-month follow-up
**Statistical Analyses**	**Acute Pain**:<br>• The effect of genotype guided opioid therapy on pain control will be determined by comparing the 10-day post-surgery SIA scores of the Intervention arm participant with CYP2D6 IM or PM phenotypes to the 10-day post-surgery SIA scores of the Control arm participant with CYP2D6 IM or PM phenotypes using a two-sided t-test or a two-sided Mann Whitney test, as appropriate, with a two-sided type 1 error rate of 0.049<br>• Similarly, the effect of genotype guided opioid therapy on all secondary endpoints will be compared between the Intervention arm CYP2D6 IM or PM phenotype and the Control arm CYP2D6 IM or PM phenotype using a two-sided t-test or a two-sided Mann Whitney test<br>• Additional analyses will include time trends in primary and secondary endpoints, subset analyses, covariate adjustments, and exploratory healthcare utilization and cost effectiveness analyses<br><br>**Chronic Pain**:<br>• The effect of genotype guided opioid therapy on pain control will be determined by comparing the change in baseline to 3-month follow-up composite pain scores of the Intervention arm participants with CYP2D6 IM or PM phenotypes to the change in baseline to 3-month follow-up composite pain scores of the Control arm participants with CYP2D6 IM or PM phenotypes using a two-sided t-test with type 1 error rate of 0.049<br>• Similarly, the effect of genotype guided opioid therapy on all chronic pain secondary endpoints will be compared between the Intervention arm CYP2D6 IM or PM subgroup and the Control arm CYP2D6 IM or PM subgroup using either a two-sided t-test or a test of two proportions, as appropriate.<br>• Additional analyses will include time trends in primary and secondary endpoints, subset analyses, covariate adjustments, and exploratory healthcare utilization and cost effectiveness analyses<br><br>**Depression**:<br>• The effect of genotype guided antidepressant therapy on depression symptoms will be determined by comparing the 3-month follow-up composite depression scores of the Intervention arm participant subgroup with CYP2D6 UM or PM or CYP2C19 UM, RM, or PM phenotypes to the 3-month follow-up composite depression scores of the Control arm participant subgroup with CYP2D6 UM or PM or CYP2C19 UM, RM, or PM phenotypes using a two-sided t-test with type 1 error rate of 0.049<br>• Similarly, the effect of genotype guided antidepressant therapy on all depression secondary endpoints will be compared between the Intervention arm CYP2D6 or CYP2C19 UM, RM or PM subgroup and the Control arm CYP2D6 or CYP2C19 UM, RM, or PM subgroup using either a two-sided t-test, a two-sided Mann Whitney test, or a test of two proportions, as appropriate.

	• Additional analyses will include time trends in primary and secondary endpoints, subset analyses, covariate adjustments, and exploratory healthcare utilization and cost effectiveness analyses<br><br>**All Trials:**<br>• Overall well-being of all Intervention participants will be compared to the overall well-being of all Control participants at 6-month follow-up using ANOVA, adjusting for baseline differences in the two groups<br>• The effect of genotype guided therapy on concordance between phenotype and prescribed medication will be compared between Intervention and Control participants from the metabolizer phenotypic subgroups specified for each trial's primary endpoint.<br>• Additional analyses will include time trends, subset analyses, and covariate adjustments analyses

2. INTRODUCTION

2.1 Background, Significance, and Rationale

Study Rationale

Pain and depression are conditions that impact substantial proportions of the US population. Finding safe and effective drug therapies for both conditions is challenging. In the case of treatment for acute and chronic pain, the challenge is finding effective therapy while minimizing adverse effects or opioid addiction (and the ensuing consequences). For depression, there are few clinically relevant predictors of successful treatment leading to multiple trials of inadequate therapy for some patients. Both opioid and antidepressant prescriptions can be guided by pharmacogenetics (PGx) data based on existing guidelines from the Clinical Pharmacogenetics Implementation Consortium (CPIC). A pilot study conducted during IGNITE-1, in patients with chronic pain supports the potential benefit of a genotype-guided approach to pain therapy.[1] Existing studies of tailored antidepressant therapy are small and often industry-sponsored but suggest the genotype-guided approach is superior to usual care. We propose a randomized pragmatic clinical trial that enrolls patients into three PGx-guided therapy scenarios: acute post-surgical pain, chronic pain, and depression. For each scenario, participants will be randomized to genotype-guided drug therapy versus usual approaches to drug therapy selection (hereafter referred to as usual care). Changes in patient reported outcomes representing pain and depression control using standard PROMIS scales define the primary endpoints. Secondary analyses include safety endpoints, changes in overall well-being, and economic impact represented by differences in healthcare utilization and cost effectiveness.

Background and Significance

Acute and chronic pain represents the most prevalent and expensive public health condition in the U.S., affecting an estimated 100M Americans with annual costs to society estimated at $635B dollars.[2, 3] This exceeds the combined costs of cancer, AIDS and heart disease.[3] Opioids have become a mainstay of treatment for chronic pain, yet analgesic responses to opioids are widely variable in both acute and chronic pain.[4, 5] Opioid prescribing rates have more than tripled since 1999, with 65 in 100 people getting an opioid prescription in 2016, and nearly 215M opioid prescriptions dispensed.[6, 7] Nearly half of all opioid prescriptions originate in primary care, and approximately 35% are from surgeons.[8]

Tramadol, codeine, hydrocodone, and oxycodone comprise the vast majority of opioids prescribed in the U.S.[9] Codeine and tramadol are dependent on bioactivation by the CYP2D6 enzyme to morphine and O-desmethyltramadol, respectively, which have 200-fold greater affinity for the μ-opioid receptor than their parent compounds. *CYP2D6* genotype has important relevance for response to codeine and tramadol. Specifically, 5-10% of individuals are poor metabolizers (PMs), with no active CYP2D6 enzyme secondary to frameshift mutations (*3, *6), splicing defects (*4), or complete gene deletion (*5). As a result, PMs are unable to generate the active metabolites of codeine and tramadol and may derive no pain relief from these drugs.[10] Another 2-11% are intermediate metabolizers (IMs), with significantly impaired enzyme activity secondary to having both a nonfunctional and a reduced function *CYP2D6* allele and may derive little pain relief from codeine and tramadol. At the opposite extreme, approximately 1-2% of individuals are ultra-rapid metabolizers (UMs) with *CYP2D6* gene duplication/multiplication. UMs are at increased risk for toxic concentrations of active opioid metabolites, with reports of life-threatening toxicity and death with codeine or tramadol.[11-15] Hydrocodone and oxycodone undergo similar metabolism via CYP2D6 to compounds with 10- to 40-fold higher receptor affinity, respectively, and recent data support *CYP2D6* genotype as an important contributor to hydrocodone efficacy, though risk exists for both drugs in those with UM phenotypes.[1]

Guidelines support *CYP2D6* genotype-guided use of opioid analgesics, but this is rarely done in clinical practice.[10] In a single center PCT[1], *CYP2D6* genotype-guided prescribing led to improved pain control in PMs and IMs compared to a traditional pain management approach. We now propose a multi-center PCT in which we will make recommendations based on *CYP2D6* genotype and CYP2D6 enzyme inhibitor drug interactions that can convert individuals to PM or IM phenotypes. In PM, IM and UM we will recommend avoidance of hydrocodone, tramadol and codeine, and for normal metabolizers (NM), tramadol will be recommended as the preferred opioid, given its opioid and non-opioid mechanisms and purported lower risk for misuse.[16, 17] One study suggested the potential for abuse and dependence with tramadol in patients with chronic non-cancer pain was significantly less than for hydrocodone, and not different from that of non-opioid analgesics.[16]

The prevalence of major depressive disorder ranges from 5 to 10% in primary care, and it can be as high as 37% after critical care hospitalizations and surgeries.[18] SSRI prescriptions have increased 5.8-fold between 1991 and 2011.[19] Response to SSRIs and other antidepressants depends on numerous factors, but pharmacokinetic adjustments based on drug-drug interactions, renal and hepatic function, and pharmacogenomic variants within *CYP2D6* and *CYP2C19* play a substantial role in interindividual drug response.[20]

Existing clinical trials of PGx-guided treatment of depression have been primarily industry-sponsored and often investigate proprietary treatment algorithms.[21] One open label, non-randomized study demonstrated significantly improved depression outcomes in 227 adults with major depressive disorder (MDD) treated with PGx-guided prescribing of psychotropic medications relative to unguided participants.[22] Another 12-week randomized, double-blind trial of 144 adults with MDD receiving PGx-guided prescribing reported a 2.52-fold greater chance of remission of depressive symptoms. A randomized clinical trial of 685 adults with anxiety and depression identified significantly improved outcomes relative to controls in patients diagnosed with depression or anxiety using pharmacogenetic-guided medication selection.[23] A meta-analysis of randomized controlled trials of pharmacogenetic tests and depressive symptom remission concluded that individuals receiving treatment of depression with pharmacogenetic-guided decision support tools were 1.71 (95% CI: 1.17-2.48; p = 0.005) times more likely to achieve symptom remission relative to individuals who receive treatment as usual.[24] Another industry-sponsored, randomized, double-blind prospective trial with only 51 study subjects (26 pharmacogenetic-guided versus 25 unguided) reported a trend toward improved clinical outcomes in a 10-week trial. PGx-guided participants with depression had greater than double the likelihood of response and remission. Mean percent improvement in depressive symptoms was higher for the PGx-guided group over Treatment as Usual (TAU). PGx-guided treatment doubled the likelihood of response in patients with treatment resistant depression.[25] A more recent double-blind randomized controlled trial of 316 adults with MDD failed to report a difference in sustained response within a 12-week period. However, the PGx-guided treatment group had a higher responder rate compared to treatment as usual.[26]

While the above clinical trials suggest improved depression outcomes with use of pharmacogenomic-guided management of psychotropic medications when treating major depression in outpatient psychiatric practices, the majority of the trials were small and sponsored by industry. Importantly, a large, definitive trial with non-proprietary drug selection algorithms has not yet been published. Three of the six most commonly used SSRIs (sertraline, citalopram, and escitalopram) require functional CYP2C19 enzyme activity for their hepatic inactivation in vivo, and CPIC recommends dose reduction in the setting of a CYP2C19 poor metabolizer phenotype (to reduce probability of side effects) and alternative drug in the setting

of a CYP2C19 ultra-rapid or rapid phenotype (to reduce the probability of pharmacotherapy failure).[27] Two additional common antidepressants (fluvoxamine, paroxetine) are oxidized by CYP2D6, one of the most polymorphic of all human enzymes. For the present investigation, PGx-guided antidepressant selection will follow CPIC guidelines for *CYP2D6* and *CYP2C19* phenotypes with regard to the selection or dosing of antidepressants.

2.2 Aims and Objectives of the Study

Our rationale for examining a genotype-guided approach to acute and chronic pain management is based on the importance of CYP2D6 for the bioactivation of tramadol, codeine, and hydrocodone and data from a pilot study supporting improved pain control in IM/PMs in the genotype-guided arm who are taking these drugs at baseline. Similarly, the rationale for examining a genotype-guided approach to depression medication therapy is based on the demonstrated role of CYP2D6 in the bio inactivation and CYP2C19 oxidation of select, commonly used SSRIs. Secondly, data from industry sponsored trials support the hypothesis of improved symptom control in a genotype-guided arm.

Acute Pain: Determine if a genotype-guided approach to acute post-surgical pain therapy leads to improved pain control compared to usual care, as defined by a decrease in the SIA score. Secondarily, we will evaluate whether this approach leads to reduced use of DEA Schedule II opioids and reduced pain intensity.

Chronic Pain: Determine if a genotype-guided approach to pain therapy in participants with atleast 3 months of chronic pain leads to improved pain control compared to usual care.

Depression: Determine if genotype-guided dosing or selection of antidepressants amongparticipants with at least 3 months of depressive symptoms who require new or revised antidepressant therapy leads to improved control of depression, compared to usual care.

3. ENDPOINTS

3.1 Primary Endpoint(s)

Acute pain: The primary endpoint for the acute pain study is the SIA score, a composite of pain and opioid usage, at 10 days post-surgical procedure. See Appendix A for the SIA score rationale.

Chronic pain: The primary endpoint for the chronic pain study is change in composite pain intensity score, assessed using the PROMIS pain intensity survey, from baseline to 3 months post return of genetic testing results to the provider.

Depression: The primary endpoint for the depression study is change in depression score, assessed using the PROMIS Emotional Distress - Depression 8b survey (adults) or PROMIS pediatric depressive symptoms (pediatric), from baseline to 3 months post return of genetic testing results to provider.

3.2 Secondary Endpoints

Secondary endpoints include:

All Trials

1. Overall well-being at 6-month follow-up
2. Sub-domains of overall well-being: pain interference, physical function, sleep disturbance, social role and activities functioning, fatigue, anxiety, and depression at 6-month follow-up
3. Concordance between metabolizer phenotype and prescribed medication

Acute Pain

1. Pain intensity at 10 days post-surgery
2. Opioid usage at 10 days post-surgery
3. Prescription pain medication misuse score at 3-months post-surgery
4. Mobility at 1-month post-surgery
5. Opioid persistence 6 months post-surgery

Chronic Pain

1. Pain reduction magnitude at 3-month post return of genetic testing results to provider, relative to baseline
2. Proportion of participants achieving clinically significant pain reduction (30% reduction from baseline) at 3-months post return of genetic testing results to provider
3. Prescription pain medication misuse at 3-months post return of genetic testing results to provider

Depression

1. Change in QIDS score between baseline and 3 months
2. Medication side effects severity burden at 3 months
3. Participant medication adherence at 3 months
4. Remission at 6 months

4. STUDY ARMS & DESIGN

This is a prospective, multicenter, subset analysis of 1:1 randomized Intervention (immediate PGx testing and genotype-guided opioid or SSRI therapy with clinical decision support) vs. Control (usual care with delayed PGx testing) pragmatic, open label clinical trial (Figure 1). Thethree trials are 1) genotype-guided opioid therapy among post-surgical acute pain participants(Acute Pain, Figure 1A), 2) genotype-guided opioid therapy among chronic pain participants (Chronic Pain, Figure 1B), and 3) genotype-guided SSRI therapy in participants with depression (Depression, Figure 1C). Trial-specific outcomes will be compared between participants in the intervention arm and control arms who have an actionable phenotype. Actionable phenotypesare defined as CYP2D6 IM and PMs (i.e. CYP2D6 activity score ≤ 0.75) for the acute pain and chronic pain trials and CYP2C19 UM, RM and PMs or CYP2D6 UM and PMs for the depression trial.

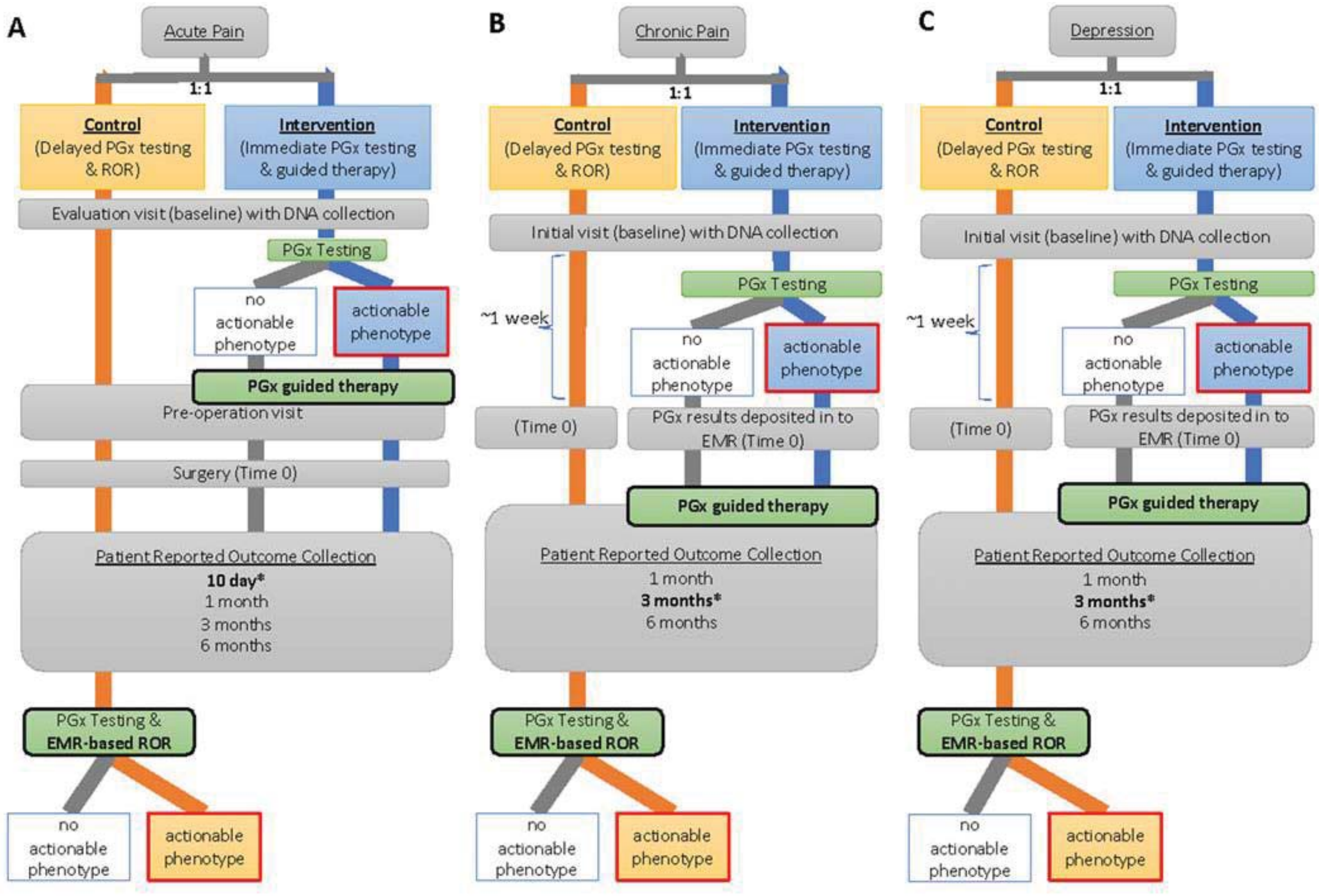

Figure 1. ADOPT PGx Trial Design

Intervention participants are denoted by blue boxes and blue lines, control participants are denoted by orange boxes and orange lines. Return of results are denoted with black outlines, endpoint comparison groups (actionable phenotypes in the control and intervention arms) are denoted with red outlines, and *

denote timing of the primary endpoint collection. Time 0 denotes the time from which the follow-up assessment timing begins.

4.1 Randomization

After participants provide informed consent/assent, participants will be randomized in a 1:1 allocation to the Intervention (i.e. immediate PGx testing and genotype-guided opioid or SSRI therapy with clinical decision support) and Control (i.e. usual care with delayed PGx testing) arms. Randomization will be stratified by trial and site or clinic with a random block size within each site or clinic. We anticipate variability in patient populations between recruiting sites or clinics and aim to balance the intervention assignments within the site or clinic unit. Additionally, the randomization for the chronic pain trial will be stratified by presence/absence of depression, and the randomization for the acute pain and depression trials will be stratified by pediatric/adult. The randomization scheme will be generated by an unblinded statistician. Randomization assignments will be generated in real time in REDCap at the Coordinating Center, and revealed after baseline data are collected so study assignment does not impact the baseline survey responses.

4.2 Blinding

ADOPT PGx randomization assignments will not be blinded to the participants or their providers but will be masked to the UF call center personnel who may be administering some of the participants follow-up surveys. Due to the nature of the intervention, it is impossible to completely mask participants and providers from the pharmacogenetic testing and return of results, hence participants, providers, and local study personnel will not be blinded. While the UF call center personnel administering follow-up surveys will be masked from the results and randomization, study-arm specific questions in the final (6 month) survey may reveal study arm randomization. Additionally participants may volunteer to reveal their randomization or PGx testing results. If the randomization or phenotype is revealed to the call center, prior to administering the 6 month surveys, it will be documented in the database.

5. STUDY POPULATION

5.1 Inclusion Criteria

Acute Pain

- Age ≥ 8 years, as this is the minimum age at which proposed outcome measures(PROMIS) are validated without parent proxy
- English speaking or Spanish speaking
- Elective/planned surgery patients with an upcoming pre-surgery visit
- Elective/planned surgery types with pain management, which may include orthopedic surgeries (e.g. arthroplasty, spine, etc.), open abdominal surgery, or cardiothoracic surgery and others

Chronic Pain

- Age ≥ 18 years
- English speaking or Spanish speaking
- Seen at primary care clinics (for example: Internal Medicine, Family Medicine, Pediatrics) or patients seen in relevant specialty clinics
- Under the care of a physician for pain for at least 3 months and currently or being considered to be treated with tramadol, hydrocodone, or codeine to improve pain management
    - Pain diagnosis (e.g.: M54.5x; R10.x; G89; R32)
    - Underlying conditions with chronic pain if a pain diagnosis does not exist (e.g.: arthritis, neuropathy, fibromyalgia, headache).
    - Have a current or planned pain prescription: tramadol, hydrocodone, or codeine

Depression

Age ≥ 8 years, as this is the minimum age at which proposed outcome measures(PROMIS) are validated without parent proxy

- English speaking or Spanish speaking
- Patients seen at primary care clinics (for example: Internal Medicine, Family Medicine, or Pediatrics) or in psychiatry clinics
- Diagnosis of depression, defined as problem list entry or ICD-10 diagnosis code (e.g. F33.x, F32.x, or F34.1) with at least 3 months of symptoms
- Anticipate need for revised or new SSRI therapy for depression

5.2 Exclusion Criteria

Trial-wide:

- Life expectancy less than 12 months
- Are too cognitively impaired to provide informed consent/assent and/or complete study protocol
- Are institutionalized or too ill to participate (i.e. mental or nursing home facility)
- Plan to move out of the area within 6 months of enrollment
- Have a history of allogeneic stem cell transplant or liver transplant

- People with prior clinical pharmacogenetic test results for genes relevant for the study in which they will enroll (*CYP2D6* for the pain studies and *CYP2D6* or *CYP2C19* for depression) or already enrolled in an ADOPT PGx trial

Acute Pain

- Adults with a similar, previous surgery in whom pain control is well-defined and a genotype-guided approach would not likely be followed
- Undergoing a laparoscopic surgery
- Receiving chronic opioid therapy, defined as use of opioids on most days for >3 months

Chronic Pain

- Undergoing treatment for an active cancer diagnosis

Depression

- Have active psychosis or diagnosed psychotic disorders (schizophrenia, schizoaffective disorder, delusional disorder, psychotic depression, substance induced psychosis, schizophreniform disorder)
    - ICD10: F06.2, F20.x, F25.x, F22, F20.81
    - Or a problem list entry indicating the diagnosis of one of these disorders
- Have dementia or other neurocognitive disorders (due to any cause, such as Alzheimer's disease, vascular/subcortical, lewy body, frontotemporal lobar degeneration)
    - ICD10: F01, F03.x, G31.83, G30.x, G31.09
    - Or a problem list entry indicating the diagnosis of one of these disorders
- Have cognitive developmental delay and/or cognitive disability, including autism spectrum disorders (Note: this list does not include ADHD)
    - ICD10: F80.x, F81.x, F82.x, F83.x, F84.x, F85.x, F88.x, F89.x
    - Or a problem list entry indicating the diagnosis of one of these disorders
- Have seizures, or who are on a neuroleptic medication
    - ICD10: G40.x, G41.x
    - Medications: aripiprazole, asenapine, cariprazine, clozapine, lurasidone, olanzapine, quetiapine, risperidone
    - Or a problem list entry indicating the diagnosis of one of these disorders
- Have bipolar disorder
    - IDC10: F31.x
    - Or a problem list entry indicating the diagnosis of one of these disorders
- Depression secondary to substance abuse disorder or general medical condition

6. RECRUITMENT AND ENROLLMENT PROCEDURES

Outlined below are **suggested strategies** for recruitment and enrollment. It is anticipated that each site will need to optimize the strategies that work best for the clinic and patient population and are in accordance with local regulations and procedures. Strategies utilized will be documented for each recruiting site (see MOP for details). Sites shall maintain local recruitment logs per local policies and share aggregated data with the CC.

6.1 Provider Recruitment and Assent

Acute Pain: Surgeon providers for eligible participants will be identified, approached by qualified study personnel, and notified that their patients will be contacted by study site recruiters to participate.

Chronic Pain: Primary care providers, pain clinic providers, and/or anesthesiologists specializingin pain control who treat patients meeting eligibility criteria will be identified, approached by qualified study personnel, and notified that their patients will be contacted by study site recruiters to participate.

Depression: Primary care providers and psychiatric providers who manage the care of eligible patients will be identified, approached by qualified study personnel, and given the opportunity to participate as a study provider. They then will be notified that their patients will be contacted by study site recruiters. to participate

6.2 Participant Consent Process

Pre-screened participants that meet the inclusion criteria will be asked to provide an informed consent/assent.

Original informed consent documents will be maintained at the site. Copies of the signed informed consent will be given to the participant. The consenting process can be remote, via a phone or electronically if approved by the reviewing IRB.

6.3 Participant Discontinuation/Withdrawal from the Study

Participants may stop participating and withdraw from the study at any time. All information and data collected up until that point will be used in the study.

If a participant wishes to withdraw consent, they should contact study staff. A participant may also revoke HIPAA authorization and must provide the revocation in writing. Study staff may attempt to obtain a reason for withdrawal from the participant and record it in the study database.

In the acute pain trial, the principal investigators or clinical site investigators may withdraw a participant from the study for <u>any</u> of these reasons:

- the participant does not have the surgery within 12 months of study enrollment
- the participant has the surgery at a healthcare system that is different from where the participant enrolled
- the participant's surgery is scheduled for a date that is ≥ 3 months after the close of the enrollment period for that clinical site
- the participant is unable to have the surgery for other medical reasons

6.4 Lost to Follow-Up

Participant status of lost to follow-up will be minimized and retention maximized through various mechanisms to collect study survey responses. The following options will be available: via phone, a web-based link, text or email. Study staff should confirm the best contact information for the participant at each study encounter.

Additionally, data collected from the EHR and Medicare and Medicaid claims data (see Section 9 for details) will be robust to missing data due to participants that are lost to follow up.

6.5 Risk

The potential risks described below are minimal and reasonable in relation to the potential benefit for genotype-guided therapy to improve the management of pain and depression and reduce the pain and depression burden to society.

<u>Blood Draw</u>
The risks of a blood draw include pain, bruising, and the slight possibility of infection at the place of needle insertion. Some people feel dizzy or may faint during or after a blood draw.

<u>Off FDA Label Use of Antidepressants</u>
In the depression trial, specifically in the pediatric population, the standard practice of medicine may include use of antidepressants that are currently not FDA approved to treat depression in pediatrics. Use of drugs not labeled for use in pediatrics, but prescribed by their treating physician, is not a risk due to study participation.

<u>Prescription Changes</u>
It is possible that the PGx-PGx-recommended drug therapy change may lead to worse pain control or worse control of depression symptoms, though the likelihood is that is no different than with the usual trial and error approach typically used for defining pain management or antidepressant therapy. Pharmacogenetic information is expected to lead to safer and more effective drug therapy, and the ultimate prescribing decision in this study will be left to the physicians. Thus, there are no anticipated risks with basing therapy on pharmacogenetic test results and our preliminary data support a clinical benefit, not risk.

<u>Genetic Information Privacy</u>
The risks of study participation are primarily those related to genetic studies, including risks related to confidentiality surrounding the genetic information and the chance that the genetic information could in some way expose the participant to increased risk regarding employment or that future life, health, disability or long-term care insurance providers could potentially use this genetic information to deny, limit or raise rates for insurance coverage. The Genetic Information Nondiscrimination Act (GINA) makes it illegal for health insurance companies, group health plans, and most employers to discriminate based on genetic information, but other insurers may still use genetic information to discriminate. The pharmacogenetics examples included in our study are only known to be associated with drug response, which is unlikely to lead to insurance discrimination as long as effective, alternative therapies are available, as they are for all gene-drug pairs included in this study.

6.6 Benefit

Study participation may not directly benefit participants of the study especially those who do not have an actionable phenotype. It is possible however that those individuals who have an actionable phenotype may benefit from this study.

Improved Drug Therapy
The genotype information could lead to improved drug therapy management for select participants. For example, *CYP2D6* genotype may help to identify participants unlikely to respond to codeine, tramadol, or hydrocodone or who are at high risk for toxicity with these opiates plus oxycodone. This could lead to prescribing of alternative opiates or other non-opioid therapies more likely to reduce pain without compromising participant safety. In addition, through improved pain management and use of lower potency opioids in individuals expected to respond well to these drugs based on genotype, the study may indirectly have positive impacts on the opioid crisis by helping to reduce the individual participant opioid burden.

Similar to the above, potential benefits exist for the SSRIs based on more appropriate dosing or selection of an alternative antidepressant. Finally, participants may benefit from optimized therapy for other drugs that may have recommendations based on the panel-based pharmacogenetic testing.

Though the study participants that are randomized to the usual care/control arm have no potential to derive benefit during their participation in the clinical trial, when they complete the 6-month follow-up, they will have their genotype recorded in the EHR, whereby it could be used to guide any future relevant therapies.

6.7 Costs to the Participants

The cost of clinical genotyping and collection of PRO outcomes will be covered by the clinical trial. The cost of their drug therapy will not be covered by the trial since they would be prescribed a medication regardless of participation in the trial. However, taking part in this study may lead to added costs to the participant, specifically the costs of their care, including the physician-prescribed drug therapy, which will generally be covered by the participant's insurance (if insured), and will not be covered by the study.

6.8 Compensation to Participants

Participants will be reimbursed for their time and effort, prorated by study completion. Sites will follow local policies and procedures for amount, timing of, and mechanism for issuing compensation to participants. Participants may be reimbursed for travel or parking expenses per institutional specific policies.

7. STUDY PROCEDURES

An overview of the study procedures to be followed is presented in this section. Recruiting sites will receive training on the protocol and MOP before site activation for enrollment.

7.1 Interventions/Treatments

The intervention, genotype-guided pain or depression therapy, is intended to reflect the practices and procedures that are likely to be implemented if PGx testing were to be integrated into standard clinical practice. The intervention has two technical components: the PGx panel testing and the clinical decisions support/clinical recommendations for providers, described below. The participant and provider facing components of the intervention include the following:

Patient Participants

1. The return of the PGx testing results to the participant's medical record that is analogous to typical lab results. Participants may discuss their results with their provider.

Providers

2. The return of PGx testing results to the participant's provider via standard site-specific laboratory return of results methods
3. Clinical decision support will be provided to all providers, how that is provided may vary. At least one of the two options below that includes drug-drug interactions, is required.
    a. A static report/consult note with interpretation of the genetic testing results and drug-drug interactions, and treatment recommendations
    b. Where possible, a provider prescribing alert for actionable phenotypes, triggered when a relevant opioid or SSRI medication is ordered that indicates the participant's genetic results and/or metabolizer phenotype, the predicted phenotype (i.e. efficacy of various opioid or SSRI medications), other considerations, and treatment recommendations

PGx Panel Testing

For all trials, the PGx testing panel includes two required genes: *CYP2C19* and *CYP2D6*. The list of drugs with responses affected by these genes that will be the focus of recommendations in the trial are found in **Table 1**. Reflecting the pragmatic nature of the trial, there may be site to site variability in the testing of specific variants due to site-specific institutional approvals. The minimal required set of *CYP2C19* and *CYP2D6* variants are listed in Appendix B.

Table 1. Genes to be tested and drugs with CPIC recommendations

Genes	Drugs
CYP2C19	citalopram, escitalopram, sertraline
CYP2D6	codeine, oxycodone, tramadol, hydrocodone, fluvoxamine, paroxetine

CYP2D6 phenotypes will be inferred based on the activity scoring system as shown in **Table 2 and Table 3**. The activity score value of each allele (**Table 2**) is added together to determine the total activity score for the diplotype. The activity scores are converted to phenotypes per

Table 3. The IM phenotype will be defined as an activity score of >0 to 0.75, not >0.75 to 1.0. As there is debate about how to define the IM phenotype, we will have an *a priori* analysis plan to determine whether participants in the usual care arm with an activity score of >0.75 to 1 (who we will define as NM) have worse pain control or depressive symptoms than NMs with an activity score > 1 to 2. If an allele is duplicated, and it is unknown which allele is duplicated, then the AS may be a ranged number, resulting in a ranged phenotype. If the ranged phenotype is NM-UM, the individual will be treated clinically as is if they were a UM.

Table 2. CYP2D6 allele to activity score

Alleles	Activity value
*1, *2	1
*9, *10, *14, *17, *29, *41	0.5
*3, *4, *5, *6, *7, *8	0

Table 3. CYP2D6 phenotype by Activity Score

Inferred CYP2D6 phenotype	*CYP2D6* activity score (AS)
UM	> 2.0
NM	>0.75 to 2.0
IM	>0 to 0.75
PM	0

CYP2C19 phenotypes will be inferred from the genotypes, as shown in **Table 4**.

Table 4. CYP2C19 phenotype by genotype

Inferred CYP2C19 phenotype	Example *CYP2C19* genotypes
UM	*17/*17
RM	*1/*17
NM	*1/*1
IM	*1/*2,*1/*3, *2/*17, *3/*17
PM	*2/*2, *2/*3, *3/*3

The CYP2C19 metabolizer phenotype and the CYP2D6 metabolizer phenotypes and/or activity scores will be included in the PGx testing report generated by the laboratory, which is then returned to the provider and deposited in the EMR, where available.

Clinical Decisions Support / Clinical Recommendations

Acute and Chronic Pain
Standardized clinical consult notes with or without pharmacogenetics expert consultations will be generated based on the genotype-inferred phenotype, and in the case of drugs including *CYP2D6* guidance, will include consideration of drug interactions.

For *CYP2D6*, those taking concomitantly a strong CYP2D6 inhibitor (as defined by FDA guidance on drug interactions[28]) will be considered to have been pheno-converted to a PM and recommendations will be consistent with that for a PM. Examples of strong inhibitors include, but are not limited to: bupropion, fluoxetine and paroxetine. Moderate inhibitors include, but are not limited to: duloxetine and mirabegron and reduce *CYP2D6* activity scores by 50%, and thus the inferred phenotype will be based on the genotype activity score x 0.5. Activity scores that align with a given phenotype, and which will ultimately be based on genotype and drug interaction data, are shown in Tables 2 and 3. A full list of strong and moderate inhibitors can be found in Appendix C.

While we will not be including oxycodone as a drug on which we will make recommendations in IMs and PMs, based on UF data and other data in the literature that make the importance of *CYP2D6* for the pain response unclear, there are data that suggest that CYP2D6 UMs can have significant toxicities (especially respiratory depression).[1, 29-31] Thus, oxycodone will be a drug for which we will make strong recommendations about avoiding use in UMs.
Using a standardized consult note/CDS or pharmacogenetics expert consultation, recommendations will be made to avoid tramadol, hydrocodone, or codeine in PMs, IMs, and UMs and to use an alternative opioid (e.g. morphine, hydromorphone) or non-opioid (e.g. NSAID), as noted in Table 5. Consideration of tramadol as the first line opioid will be recommended for NMs. While not part of the primary hypothesis, for safety reasons, avoidance of oxycodone will also be recommended in UMs.

Additionally, where possible, the CDS will include a provider alert for actionable phenotypes, triggered when a relevant opioid medication is ordered that indicates the participant's genetic results, the predicted phenotype, other considerations, and treatment recommendations.

Table 5. CDS summary for acute and chronic pain treatments

Treatment	CYP2D6 Phenotypes				
	**Ultra-rapid**	**Normal to Ultra-Rapid**	**Normal**	**Intermediate**	**Poor**
Tramadol	Avoid	Avoid	Preferred opioid	Avoid	Avoid
Codeine	Avoid	Avoid	Acceptable	Avoid	Avoid
Hydrocodone	Avoid	Avoid	Acceptable	Avoid	Avoid
Oxycodone	Avoid	Avoid	Acceptable	Acceptable	Acceptable

Depression
Clinical decision support in the form of computerized alerts, standardized clinical consult notes, and/or pharmacist consultations will be generated to guide prescribers on dosing or selection of

SSRIs or selection of alternate antidepressants based on predicted phenotype. Recommendations on drug choice and starting dose will be made in accordance with CPIC guidelines and/or FDA label information for paroxetine, fluvoxamine, citalopram, escitalopram, and sertraline, as outlined in **Table 6.** Drug-drug interactions leading to pheno-conversion will be incorporated into recommendations. For CYP2D6, concomitant use of a strong inhibitor (listed above) will result in a predicted phenotype of poor metabolizer, and the effect of concomitant moderate inhibitor (listed above) will be estimated by multiplying the CYP2D6 activity score by 0.5.

Table 6. CDS summary for antidepressant medications

	***CYP2D6* Phenotypes**				
	**Ultra-rapid**	**Normal to Ultra-rapid**	**Normal**	**Intermediate**	**Poor**
Paroxetine	Avoid	Avoid	Acceptable	Acceptable	Avoid or 50% Dose Reduction*
Fluvoxamine	No recommend ation	No recommend ation	Acceptable	Acceptable	Avoid or 25-50% Dose Reduction*
	***CYP2C19* Phenotypes**				
	**Ultra-rapid and Rapid**		**Normal**	**Intermediate**	**Poor**
Citalopram	Avoid		Acceptable	Acceptable	Avoid or 50% Reduction**
Escitalopram	Avoid		Acceptable	Acceptable	Avoid or 50% Reduction*
Sertraline	Monitor for Non-response		Acceptable	Acceptable	Avoid or 50% Reduction*

* Dose reductions refer to starting dose of medication; Avoid refers to recommendation to switch to a drug not predominantly metabolized by the listed drug metabolizing enzyme. No recommendation refers to a scenario where CDS is not triggered but the drug is also not offered as an alternative given the phenotype.
** Per the FDA warning, citalopram 20 mg/day is the maximum recommended dose in *CYP2C19* poor metabolizers due to the risk of QT prolongation

Context of the Intervention Implementations

Reflecting the pragmatic nature of the clinical trial, the intervention may be implemented in environments in which there are existing PGx testing and CDS. The PGx testing may be completed as part of larger, CLIA validated, PGx panel that include optional gene-drug pairs

(Table 7). Additionally, clinical decision support may be in place for the study gene-drug pairs as well as other CPIC drug-gene pairs and/or other drugs affected by the study genes.

Table 7. PGx panel genes and drugs with CPIC recommendations

Genes	Drugs
CYP2C19 ***(Required)***	amitriptyline, clopidogrel, clomipramine, doxepin, imipramine, trimipramine, voriconazole, omeprazole, esomeprazole, pantoprazole, lansoprazole, dexlansoprazole
CYP2D6 ***(Required)***	amitriptyline, clomipramine, desipramine, doxepin, imipramine, nortriptyline, ondansetron, trimipramine, tropisetron
CYP2C9	warfarin, phenytoin
CYP3A5	Tacrolimus
SLCO1B1	Simvastatin
TPMT	azathioprine, mercaptopurine, thioguanine
NUDT15	azathioprine, mercaptopurine, thioguanine
VKORC1	Warfarin
CYP4F2	Warfarin

7.2 Baseline Participant Assessments

All study participants will complete baseline surveys for demographics and participant reported outcomes (PROs) (see Table 8 for details). These data will be collected by the study coordinator in person or alternatively by using telephone, email, or text.

Baseline data will be collected for the Chronic Pain and Depression trials following consent, for the Acute pain trial, baseline data will be collected within 30 days before surgery. The type of surgery information will be collected after the completed procedure.

Participant demographics – including but not limited to age, sex, gender, race, Hispanic/Latino ethnicity (Yes or no), and smoking status will be administered at baseline as a survey.

Past medical history – A snapshot of the participant’s medical history in the medical record.

Baseline medications - Participant medications will be collected through EHR. Additionally, participant prescription and over the counter medications will be collected using patient surveys.

7.3 Follow-up Participant Assessments

The event that starts the post-intervention data collection timing are as follows.

Acute Pain:

1. Date of surgery (intervention and control arms)

Chronic Pain and Depression:

2. Intervention arm: When the pharmacogenetics results are returned to the provider and deposited in the EMR
3. Control arm: At date of DNA sample collection plus one week, where one week reflects the average time from sample collection to returning the results to the EHR
4. Note: There will be variability in time the results are returned to provider and when genotype-guided therapy is delivered to the participant, time from results being available to the next provider-participant interaction (e.g. email, clinic visit, new prescription order, phone visit, etc.) will be recorded by study personnel.

Post intervention assessment collection will be done by the University of Florida College of Pharmacy Call Center. The center agents will be trained survey collectors*, using an IRB approved telephone script. The collected survey responses will be entered into the study database. If preferred, the participant will have the option to complete the study follow-up survey by a URL link sent by text (to mobile phone) or email and captured in REDCap database

In the event that the call center is unable to reach a participant, local study coordinators may contact participants to facilitate completion of the follow-up surveys.

Data will be collected in the following timeframes*:

- Acute pain: 10 days , 1 month, 3 months, and 6 months post-surgery
- Chronic pain and depression: 1-month , 3 months , and 6 months post return of results for the intervention arm, and post baseline assessments for the control arm.
  **See MOP for call interval windows*

All Trials

PROMIS 43 – Adults (≥ 18 years of age) will complete a 43-question survey assessing well-beingand sub domains: pain interference, physical function, sleep disturbance, social role and activitiesfunctioning, fatigue, anxiety, and depression[32]. This survey will be administered to adult participants at baseline, 1-month, 3-months, and 6-months post-surgery (acute pain), or baseline,1-month, 3-months, and 6-months post return of results (chronic pain, depression).

PROMIS pediatrics 37 – Pediatric study participants (ages 8-17) will complete a 37-question survey assessing pediatric well-being and sub domains: pain interference, physical function, sleep disturbance, social role and activities functioning, fatigue, anxiety, and depression. This survey will be administered to pediatric participants at baseline, 1-month, 3-months, and 6-months post-surgery (acute pain), or baseline, 1 month, 3 months, and 6 months post return of results (chronic pain, depression).

Health care utilization – Survey of the number of significant cost driver health care encounters (e.g. hospitalizations, clinic visits, etc.). This survey will be administered to participants at 10-day, 1-month, 3-months, and 6-months post-surgery (acute pain), or baseline, 1-month, 3-months, and 6-months post return of results (chronic pain, depression).

Medications - Participant prescription and over the counter medications will be collected using patient surveys at the primary endpoint timepoints: 10-days and 1 month for the acute pain participants, 3-month for the chronic pain and depression participants.

Productivity loss – Survey of the time and pay lost due to the participant's depression or pain. This survey will be administered to participants at 10-day, 1-month, 3-months, and 6-months post-surgery (acute pain), or baseline, 1-month, 3-months, and 6-months post return of results (chronic pain, depression)

Acute Pain:

PROMIS Numeric Rating Scale - Pain Intensity - An 11-point numeric scale ranging from 0 (no pain) to 10 (worst possible pain) for average pain. This survey will be administered to all participants at baseline, 10 days, 1-month, 3-months, and 6-months post-surgery.

PROMIS pain intensity scale – A participant completed 3-question survey of average pain over the last 7 days, worst pain over the last 7 days, and current pain. Each question is on a 1-5 integer scale, the higher the value, the more intense the pain. This survey will be administered to all participants at baseline, 10 days, 1-month, 3-months, and 6-months post-surgery.

Opioid consumption questionnaire – A participant completed survey of pain prescription medication consumption, including the type of opioid pain medication, the date the prescription was filled, number of pills dispensed, if a refill has been obtained, and the number of pills left for each opioid pain medication selected. This survey will be administered to all participants at 10 days and 1-month post-surgery. Study participants will also be asked to read the tablet strength from their prescription bottle but this will be verified by local study coordinators by reviewing the EHR for the prescription written.

PROMIS prescription medication mis-use scale - A participant completed survey of prescription medication misuse over the past 3 months. The survey is comprised of 7 questions on a 5-point ordinal scale of never/rarely/sometimes/frequently/almost always or not at all/a little bit/somewhat/quite a bit/very much[33]. This survey will be administered at baseline, 3-months, and 6-months post-surgery.

PROMIS Item Bank v2.0 Mobility – A participant completed survey of the participant's level of difficulty in completing different physical activities such as standing unassisted, walking, and sprinting or activities that their health currently limits. The survey is comprised of 15 questions, each answered on a 5-point scale ranging from without difficulty to unable to do, or not at all through cannot do[34]. This survey will be administered at baseline, 10 days, 1-month, 3-months, and 6-months post-surgery to the adult participants.

PROMIS Pediatric Mobility – Short Form 8a – A participant completed 8 item survey of difficulty in completing different physical activities such as getting up from the floor, standing on tiptoes, and ability to do sports and exercises that their peers can complete [35]. The survey will be administered at baseline, 10 days, 1-month, 3-months, and 6-months post-surgery to the pediatric participants.

Acute pain specific health care utilization – A participant completed survey of hospital length of stay for the surgical admission, administered at 1-month post-surgery.

Opioid side effects – A participant completed survey of common opioid side effects experienced and the extent to which those side effects bothered the participant.

Opioid persistence - Whether or not the participant had an opioid prescription refill 90 – 180 days post-surgery. This survey will be administered to participants at 6 months post-surgery.

Chronic Pain:
PROMIS pain intensity scale – A participant completed 3-question survey of average pain over the last 7 days, worst pain over the last 7 days, and current pain. Each question is on a 1-5 integer scale, the higher the value, the more intense the pain. This survey will be administered at baseline, 1 month, 3 months, and 6 months post-index return of results.

PROMIS prescription pain medication mis-use scale - A participant completed survey of prescription medication misuse over the past 30 days. The survey is comprised of 7 questions on a 5-point ordinal scale of never/rarely/sometimes/frequently/almost always or not at all/a little bit/somewhat/quite a bit/very much[33]. This survey will be administered at baseline, 3 months, and 6 months post-index return of results.

PROMIS emotional distress depression 8b survey - A participant completed eight question survey assessing frequency of depression symptoms over the past 7 days[36]. This survey will be administered at baseline, 3 months, and 6 months post return of results to the adult participants.

Opioid side effects – A participant completed survey of common opioid side effects experienced and the extent to which those side effects bothered the participant. This survey will be administered at baseline, 3 months, and 6 months post return of results to the adult participants.

Depression:
PROMIS emotional distress depression 8b survey - A participant completed eight question survey assessing frequency of depression symptoms over the past 7 days[36]. This survey will be administered at baseline, 3 months, and 6 months post return of results to the adult participants.

PROMIS Pediatric Depressive Symptoms Short Form 8a- A pediatric (ages 8-17 years) participant completed eight question survey assessing frequency of depression symptoms over the past 7 days[37]. This survey will be administered at baseline, 3 months, and 6 months post return of results to the pediatric study participants .

Quick Inventory of Depressive Symptomatology – A 16 item survey completed by the participant assessing depression symptoms[38]. This survey will be administered at baseline, 3 months, and 6 months post return of results.

Antidepressant side effects – A participant completed survey of relevant antidepressant side effects. This survey will be administered at baseline, 3 months, and 6 months post return of results.

Voils Medication Adherence - A participant completed three question survey, 5-point scale survey assessing participant medication adherence[39]. This survey will be administered to depression trial participants at baseline and 3-months post return of results.

All of the PROMIS® surveys can be found online[40].

7.4 Specimen Collection

DNA sample collection will be by whole blood collection by venipuncture, saliva, buccal swab, or mouthwash collection, whichever is most appropriate for the site, study population, and if possible, participant preferences. This will occur in person at or after the baseline visit or by mailed kits for all randomized participants by the site personnel..

Blood Draw Protocol
After participants consent to the study and complete the baseline assessments, a trained phlebotomist will collect 1 tube (approximately 2 tablespoons) of blood via venipuncture from participants willing to provide a blood sample. Participants will then be randomized to the Control or Intervention arm. Recruiters will label the tube with participant ID from the study database and the date the sample was collected, and follow the MOP for the collection, storage, and delivery of the sample to the designated laboratory.

Saliva Protocol
Alternatively, after participants consent to the study and complete the baseline assessments, participants may provide a saliva sample in lieu of a blood sample. This collection may be completed with the assistance of research coordinators in-person or with detailed instructions mailed to the participant along with the saliva collection kit . Sample collection tubes will be labeled by recruiters, and collection, storage, and delivery to the designated laboratory will follow the Saliva Collection Protocol (to be provided in the MOP) for both in-person. Participants providing a saliva sample cannot eat, drink, smoke, or chew gum for 30 minutes before sample collection.

Buccal Cell Protocol
Alternatively, after participants consent to the study and complete the baseline assessments, participants may provide a buccal cell sample in lieu of a blood sample. This collection may be completed with the assistance of research coordinators in-person or with detailed instructions mailed to the participant along with the saliva collection kit. Sample collection swabs/brushes will be labeled by recruiters, and collection, storage, and delivery to the designated laboratory will follow the Buccal Cell Collection Protocol provided in the MOP. Participants providing a buccal cell sample cannot eat, drink, smoke, or chew gum for 30 minutes before sample collection.

Mouthwash Protocol
Alternatively, after participants consent to the study and complete the baseline assessments, participants may provide a mouthwash sample in lieu of a blood sample. This collection may be completed with the assistance of research coordinators in-person or with detailed instructions mailed to the participant along with the mouthwash collection kit. Sample collection tubes will be labeled by recruiters, and collection, storage, and delivery to the designated laboratory will follow the Buccal Cell Collection Protocol provided in the MOP. Participants providing a mouthwash sample cannot eat, drink, smoke, or chew gum for 30 minutes before sample collection.

7.5 Specimen Transfer and Genetic Testing Procedures

All collected specimens will be clearly marked with two patient identifiers (CLIA requirement). Samples from the Intervention arm participants will be processed, using analytically validated PGx testing procedures (see Table 1 for gene and variant list). For Control arm samples, designated laboratories will either extract DNA after sample receipt, and store the DNA for later analysis or store the sample and extract DNA after the Control participant has completed the 6-month follow-up assessment. After the Control participant has completed the 6-month follow-up assessment, Control Arm samples will be processed using analytically validated procedures. Details of sample storage and transport will be presented in the MOP.

7.6 Return of Results

The designated laboratory will transfer participant genetic testing results to the EHR. Genetic results will be imported into the study database. Providers will be notified of participant results via standard site-specific laboratory return of results methods, a static report/consult note with interpretation and treatment recommendations, and, where possible, a CDS within the EHR as described in Sections 7.1 and 7.7.

After participants have completed their 6-month assessments, the PGx testing results will be actively returned to the participants. The PGx testing results that are returned to the participant will follow current FDA guidance.

7.7 Clinical Decision Support Systems

Providers will be notified of participant results via standard site-specific laboratory return of results methods, a static report/consult note with both interpretation and treatment recommendations, and, where possible, a just in time alert within the electronic health system. Sites will work with their institutional information technology departments to set up provider alerts for actionable PGx phenotypes when a relevant opioid or SSRI medication is ordered. Provider alerts will indicate the participant's genetic results, the predicted phenotype (e.g. efficacy of various opioid or SSRIs), and prescribing recommendations.

Continuing on the health IT collaborative efforts established in IGNITE I through CDSKB (http://cdskb.org), all participating sites will seek to harmonize their CDS logic, recommendations, and provider alerts using a framework of required and optional elements for both the static report and the just in time alert. However, due to the pragmatic nature of the trial, the many involved research sites, and variations in local CDS policies, it is anticipated that there will be differences in CDS implementation details, but all sites will ensure that providers are alerted to the PGx test results and associated significance. Details of the alerts will be provided in the MOP.

7.8 Data Collection from the Electronic Health Record

Data from the local EHR will be used to assess participant's medications, medication changes, medication discontinuations, and, if available, reasons for medication discontinuations for the study interval baseline through six-month follow up. Sites will extract data using prespecified programmatic algorithms, site-developed algorithms, or manual chart abstractions. EHR query results will be sent to the IGNITE PTN CC via secure data transfer and formatted for consistency across sites. Details for the EHR download, manual chart abstractions, and transfer process will be provided in the MOP.

7.9 Data Collection from CMS and State Medicaid Agencies

Medicare and Medicaid claims data from CMS and state Medicaid agencies will be used to assess health care utilization, costs, and medications among participants covered by either Medicare or Medicaid. Claims made in the period of 12 months prior to surgery (acute pain) or return of results (chronic pain, depression) through 6 months following those events will be collected. See Section 9 and the MOP for additional details. Schedule of Activities and Timeframe for Collection of Endpoints

Table 8. Data Collection Schedule

Data collection schedule, method of data collection, and timing

Outcome	Data source/instrument	Ascertainment method	Baseline	10 d ± 3 d	1 mo. ± 7 d	3 mo. ± 14 d	6 mo. ± 14 d
**Pain Assessments**							
Pain intensity	PROMIS Pain intensity scales	Personal device/ Call center	AP, CP	AP	AP, CP	AP, CP*	AP, CP
Pain intensity	PROMIS Numeric Rating Scale - Pain Intensity (NPRS)	Personal device/ Call center	AP	AP*	AP	AP	AP
Daily opioid dose & type of opioid use	Average daily mg morphine equivalents (MED) since discharge use of opioids: tramadol/ codeine vs others	Personal device/ Call center		AP*	AP		
Opioid use disorder	PROMIS - Prescription pain medication misuse subscale	Personal device/ call center	AP, CP			AP, CP	AP, CP
Mobility	PROMIS mobility	Personal device/ Call center	AP	AP	AP	AP	AP
**Depression Assessments**							
Depressive state	PROMIS Emotional Distress - Depression 8b survey	Personal device/ call center	CP, D		CP, D	CP, D*	CP, D
Depressive state	Quick Inventory of Depressive Symptomology	Personal device/ call center	D			D	D
**Participant Medications**							
Assessment of prescriptions	Participant visits	EHR	AP, CP, D	AP	AP, CP, D	AP, CP, D	AP, CP, D
Assessment of prescriptions	Custom survey	Personal device/Call center	AP, CP, D	AP		CP, D	
Assessment of filled prescriptions	Medicaid and Medicare billing records for 12 months prior to time 0 through 6 months after time 0	Site States and CMS	AP, CP, D				
Adherence	Voils Medication Adherence	Personal device/Call center	D		D	D	D
Changes in Prescription	Participant visits	Abstracted from EHR			D, CP	D, CP	D, CP
Reason for discontinuation	Participant visits	Abstracted from EHR	D		D	D	D

SSRI AE Survey	Custom survey	Personal device/Call center	D			D	
Opioid side effect survey	Adapted medication side effect survey (SPACE)	Personal device/Call center	AP, CP		AP, CP	AP, CP	AP, CP
Opioid persistence	Custom survey	EHR and Personal device/ call center					AP
**Participant Actions**							
Interaction with provider	Participant encounters	Abstracted from EHR		CP, D			
Interaction with test result	Survey	Personal device/ Call center					AP, CP, D
**Quality of life and well being**							
Past Medical History	Participant visits	Abstracted from EHR	AP, CP, D				
Well-being	PROMIS43/PROMIS Peds37	Personal device/call center	AP, CP, D		AP, CP, D	AP, CP, D	AP, CP, D
**Healthcare Utilization and Costs**							
Medicare/Medicaid billing records	Medicaid and Medicare billing records for 12 months prior to time 0 through 6 months after time 0	Site States and CMS	AP, CP, D				
LoS	LoS for index admission	Personal device/Call center		AP			
ED/urgent care & inpatient visits	Participant reported visits	Personal device/Call center			AP, CP, D	AP, CP, D	AP, CP, D
Outpatient visits	Participant reported primary care & pain clinic visits	Personal device/Call center			AP, CP, D	AP, CP, D	AP, CP, D
Outpatient visits	Long-term care or in-patient rehab days, home healthcare days	Personal device/Call center			AP	AP	AP
Productively Loss	Survey of lost work time and income	Personal device/Call center			AP, CP, D	AP, CP, D	AP, CP, D

Abbreviations: CP – chronic pain, AP – acute post-surgical pain, D-depression, ED – emergency department, EHR – electronic health record; LoS – length of stay; PT – physical therapy
*Denote primary endpoints

8. SAFETY ASSESSMENT AND MONITORING

This is an observational study that does not include a drug or device intervention. Unanticipated events or problems related to the research study and that involves risks to subjects or other will be reported to the IRB. These events are defined as unexpected in terms of nature, severity or frequency given the study procedures, informed consent and characteristics of the subject population.

The IRB reporting timeline requirements are:

- Immediately (within 24 hours) upon learning of an unanticipated study-related death. Study personnel will notify the IRB via phone or email by providing a brief summary of the event; then within 1 week (5 business days), study personnel should submit report to IRB
- Within 5 business days for unanticipated events
- Within 10 business days for any other problem or event

8.1 Medication Side Effects

Participant reported medication side effects will be collected in the baseline and follow-up surveys. The opioid medication side effects that will be surveyed include: problems with sleep; nausea, gas or indigestion; constipation or diarrhea; and dizziness or balance problems. The SSRI medication side effects that will be surveyed include: fatigue, change in weight, GI upset, sedation/somnolence, anxiety, insomnia, irritability/hostility, and sexual dysfunction.

8.2 Events of Interest

Participant reported emergency department visits and hospitalizations will be collected in the 1, 3, and 6-month follow-up participant surveys. EHR data will also be reviewed to monitor these events. Sites will conduct two EHR data downloads, one after approximately half of the participants have completed their 6 surveys, and another approximately 12 months after the last patient is randomized. EHR results will be sent to the CC via secure data transfer and formatted for data analyses. Details of the EHR download and transfer process will be provided in the MOP.

8.3 Data Safety Monitoring Board

The DSMB, appointed by the NHGRI, will be responsible for providing recommendations regarding the conduct of the study and guidance to ensure the safety and well-being of participants. The DSMB will meet semi-annually. A DSMB Charter will be developed detailing the procedures to be followed. A Data and Safety Monitoring Plan and a separate DSMB statistical analysis plan (SAP) will be developed in collaboration with the IGNITE PTN, IGNITE PTN CC, and NHGRI, and enacted by the DSMB.

8.4 Early Termination and Participant Discontinuation

Early termination considerations will generally apply only to emerging issues of major concern, or problems with trial conduct that suggest the trial could not be completed successfully with a reliable conclusion in a feasible time frame.

The site investigator, sponsor or institution may stop involvement of any participant in this research study at any time without their consent. This may be because the research study is

being stopped, the instructions of the study team have not been followed, the investigator believes it is in the participant’s best interest, or for any other reason. If specimens or data havebeen stored as part of the research study, they too may be destroyed without participant consent.

9. MEDICARE AND MEDICAID CLAIMS DATA COLLECTION AND ANALYSIS

9.1 Rationale

The economic analyses will be conducted from the perspective of the payer with the primary goal to provide cost-effectiveness data that can inform reimbursement decisions for genotyping. Reporting metrics will include:

1. Average costs for each study arm, considering overall cost to the payer and select services directly relevant to the acute pain, chronic pain, and depression groups
2. Differences in cost utility between the intervention arm, considering overall cost to the payer and select services directly relevant to the acute pain, chronic pain, and depression groups
3. Incremental cost-effectiveness ratio (ICER), i.e., the incremental change in cost per unit improvement of effectiveness.

All metrics will be ascertained over a 6-month follow-up period with the assumption that beneficial effects of genotype-guided therapy are fully realized within this time period and that the control arm participants have not yet developed any cross-over effects from the 6-month delayed testing.

In a population of mixed payer types, collecting actual costs for all participants is not feasible. Therefore, costs will be obtained from Medicare and Medicaid claims data and imputed or cross-walked to the other payer types. Based on data in the National Inpatient Sample, 53% and 4% of knee and 59% and 4% of hip arthroplasties were reimbursed by Medicare and Medicaid, respectively. For chronic pain participants, data from the University of Florida suggest 40% and 20% are covered by Medicare and Medicaid, respectively. Thus, considering enrollment gaps, at least 60% of the study population is anticipated to have claims data, from which comprehensive cost analyses can be performed and extrapolated to the entire cohort. Major cost drivers associated with pain and depression will be ascertained from the participant using validated resource questionnaires, and verified through secondary claims data whenever possible.

9.2 Data Collection

Data for this specialized analysis will come from these sources:

1. Participant report of healthcare utilization (e.g. emergency department, urgent care, office) for all participants, these will focus on high-cost items and/or items that are expected to be sensitive to the intervention
2. Medicare claims data for Medicare enrollees
3. Medicaid claims data for Medicaid enrollees

Participant reported visits: Participant reported assessment for health care utilization is previously described in section 7.3.

Medicare claims data: For Medicare enrollees in fee-for-service (FFS) plans or Medicare Advantage, clinical groups will ascertain Medicare claims data (Part A-D) directly from the Centers for Medicare and Medicaid Services (CMS).

Medicaid claims data: For Medicaid enrollees, clinical groups will ascertain claims data from their states' Medicaid agencies directly.

To ensure full adjudication, requests to Medicare/Medicaid for claims data will be timed to occur between 6-12 months after the end of the 6-months follow-up of the last enrolled patient. Claims

data will also be requested for one year before trial enrollment up to 6 months thereafter.

The procedure for obtaining claims data will require a database linkage step – mapping the participant study ID to the Medicare/Medicaid identifiers. The data linkage will rely on social security number (SSN) or Medicare ID and date of birth (DoB), which can be collected in two possible ways:

1. Both SSN and/or Medicare ID and DoB are collected as part of the trial by the recruiting site
2. One or both variables are extracted from the medical record of the participating health center by a healthcare data security officer or the local equivalent, on behalf of the study team

Costs Imputation

The Medicare/Medicaid (M/M) claims will be used to impute healthcare utilization and costs for participants who are enrolled in other plans or not insured as follows. First, the subset of the study participants with both the participant reported set of medical encounters and M/M claims data will be identified. In this subgroup, claims data and self-report data will be used to derive extrapolation factors for total healthcare utilization and cost across all study participants. Additionally, for participants in other plans or uninsured, we will use M/M data to assign average cost to each of the self-reported items.

10. STATISTICAL ANALYSIS PLAN AND SAMPLE SIZE

10.1 Sample Size Determination

All trials power analyses are based on a modified intent to treat analysis, comparing the subset of intervention participants with have an actionable phenotype to the subset of control participants with an actionable phenotype. Effect sizes used to estimate power are based on prior studies of pain and depression pharmacogenetic testing and described below for each trial and shown in Table 8. Several steps were taken to generate a conservative sample size estimate. First, the power requirement was set to 90%. Second, sample sizes are adjusted for a 10% drop-out (for which UF has data from several studies that this is a good estimate). Third, slightly smaller effect sizes than what was observed in prior studies was used. Finally, sample size calculations are based on an alpha of 0.049, to allow one interim analysis with an alpha of 0.001 in each study.

Acute Pain

In the University of Florida's pilot acute pain study, the difference between study arms in SIA score was –38.55 with a SD of 93.5, for a standardized effect size of 0.412. Assuming a reduced standardized effect size of 0.375, 302 participants with an actionable phenotype per group, 151 from the intervention arm and 151 from control arm, are required to achieve 90% power for a two-sided two-sample t-test. Preliminary data from the University of Florida's acute pain study indicates the proportion of participants with an actionable phenotype, genotypic or pheno-converted *CYP2D6* IM or PM, will be approximately 18%. After adjusting for the subset anticipated to have an actionable phenotype, 839 participants completing all assessments, per group, are required to achieve 90% power in the sub-group analysis (1678 total). Assuming 10% drop out or lost to follow-up and 7% of participants not going on to surgery, a total of 2004 randomized participants is required to sufficiently power this study. Factoring in +/- 3% on the proportion of the randomized population that are IMs or PMs, 1718 to 2406 randomized subjects would be need to achieve 90% power if the proportion of IMs or PMs is 21% or 15%.

Chronic Pain

In the University of Florida's prior chronic pain study, the difference between study arms in composite pain score was 0.6 with a SD of 1.4, for a standardized effect size of 0.43. The same effect size was observed when assessing 3-month changes in pain scores. Assuming a standardized effect size of 0.40, 264 participants within the specified subgroup, 134 from the intervention arm and 134 from control arm, are required to achieve 90% power for a two-sided two-sample t-test. Preliminary data indicate the proportion of participants with an actionable phenotype will be 30-35%; 10-15% of participants with an actionable genotype-based phenotype (*CYP2D6* IM or PM) and an additional 20% with a pheno-converted PM/IM based on drug-drug-gene interactions. After adjusting for the subset anticipated to have an actionable phenotype, 447 participants completing all assessments, per group, are required to achieve 90% power in the sub-group analysis (894 total). Assuming 10% drop out or lost to follow-up, a total of 993 randomized participants is required to sufficiently power this study. Factoring in +/- 3% on the proportion of the randomized population that are IMs or PMs, 903 to 1102 randomized subjects would be need to achieve 90% power if the proportion of IMs or PMs is 33% to 27%.

Depression

In prior studies of depression, the difference between study arms in PROMIS 8a T-scores was –6.7 with a SD of 10, for a standardized effect size of -.67. For the purposes of power analysis, a

4-point change is considered the minimally important difference representing a response to therapy that is congruent with the predicted CYP enzyme phenotype compared to the response to therapy which conflicts with the phenotype.

Preliminary data from published studies indicate the proportion of participants with an actionable phenotype (CYP2C19 PM, RM, UM or CYP2D6 PM or UM, or pheno-converted *CYP2D6* PM or UM) will be 40%. However, due to the distribution of study-related antidepressants in primary care and behavioral health settings, an estimated 67% of the participants with an actionable phenotype are anticipated to also have a study-related dosing or drug selection intervention. In other words, the analytic group of CYP2DC UM, PM, or CYP2C19UM, RM, or PM phenotypes will represent a mixture of participants that may have a clinically meaningful change in depression due to being prescribed a study-relevant antidepressant and participants that may have changes in depression typical of the standard of care arm since they are not being prescribed a study-relevant antidepressant. In accounting for this population mixture, we incorporated a dilution factor of 67% into the clinically meaningful effect size projection and assume the effective difference of 0.28, representing a 2.8-point change to the T-score. We will need 544 modified intent to treat participants, 272 in each arm, to detect a standardized effect size of 0.28 with 90% power. After adjusting for the subset anticipated to have an actionable phenotype (i.e. 40%), 680 participants completing all assessments, per group, are required to achieve 90% power in the mITT analysis (1360 total). After accounting for 90% retention, 756 participants from the intervention arm and 756 participants from control arm (1512 total) are required to achieve 90% power for a two-sided two-sample t-test. Factoringin +/- 3% on the mITT proportion of the randomized population, 1401 to 1628 randomized subjects would be needed to achieve 90% power.

Table 9. Sample size calculations

Sample size calculations for each trial, assuming an alpha of 0.049 to allow for one interim analysis

Study	Assumed Actionable Phenotype	Total N	Completed N (90% assumed)	Intervention	Usual Care	Intervention: Actionable Phenotype	Usual Care: Actionable Phenotype	Effect Size	Power
Acute Pain (*CYP2D6* IM/PM)	18%	2,004	1,678*	839	839	151	151	0.375	90.00%
Chronic Pain (*CYP2D6* IM/PM)	30%	993	893	447	447	134	134	0.4	90.00%
Depression (*CYP2D6* or *CYP2C19* PM/UM)	40%	1,512	1,360	680	680	272	272	0.28	90.00%
**Total**		4,509	3,931	1,966	1,966	557	557		

*Accounts for an additional 7% of participants not completing surgery

10.2 General Statistical Methods

A detailed statistical analysis plan will be developed for each trial (acute pain, chronic pain, and depression) and contained in a separate document. Prior to analysis, each trial study population details, including the number randomized, in each treatment arm, and lost to follow-up will be described. Baseline participant characteristics will be summarized as means, standard deviations, medians, and 25th, 75th percentiles for continuous variables, and as counts and percentages for categorical variables. Model assumptions will be examined prior to analysis and transformations implemented, if necessary, to more adequately meet the assumptions. Unless otherwise stated, hypotheses will be tested as two-sided with a significance level of 0.05.

10.3 Population for Analyses

Acute Pain

Participants randomized who have an actionable phenotype, specifically participants who are genotypically or pheno-converted CYP2D6 IM or PMs (i.e. CYP2D6 activity score ≤ 0.75).

Chronic Pain

Participants randomized who have an actionable phenotype, specifically participants who are genotypically or pheno-converted CYP2D6 IM or PMs (i.e. CYP2D6 activity score ≤ 0.75).

Depression

Participants randomized who have an actionable phenotype, specifically participants who are genotypically or pheno-converted CYP2D6 PM or UMs or CYP2C19 PM, RM, or UMs.

10.4 Analysis of the Primary Endpoint

Acute Pain

To determine the effect of genotype-guided drug therapy on pain control in acute pain participants, we will conduct a subset analysis comparing SIA scores at 10-days post-surgery in the Intervention group subset with CYP2D6 IM or PM phenotypes to the Control group subset with CYP2D6 IM or PM phenotypes. Participants that have other CYP2D6 phenotypes will not be included. The 10-day post-surgery SIA scores of the Intervention CYP2D6 IM/PM group will

be compared to the 10-day post-surgery SIA scores of the Control CYP2D6 IM/PM group using a two-sided two-sample t-test or Mann Whitney test, as appropriate, with a type 1 error of 0.049. This primary analysis will be conducted as a modified intent-to-treat analysis, with participants analyzed and endpoints attributed according to the treatment arm to which the participants were randomized, regardless of subsequent crossover or post-randomization medical care.

Chronic Pain

To determine the effect of genotype-guided drug therapy on pain control in chronic pain participants, we will conduct a subset analysis comparing change in pain intensity score from baseline to 3-month follow-up in the Intervention group subset with CYP2D6 IM or PM phenotypes to the Control group subset with CYP2D6 IM or PM phenotypes. Participants that have other CYP2D6 phenotypes will not be included. The baseline to 3-month change in pain intensity of the Intervention CYP2D6 IM/PM group will be compared to the baseline to 3-month change in pain intensity of the Control CYP2D6 IM/PM group using a two-sided two-sample t-test or Mann Whitney test, as appropriate, with a type 1 error of 0.049. This primary analysis will be conducted according to modified intent-to-treat design, with participants analyzed and endpoints attributed according to the treatment arm to which the participants were randomized, regardless of subsequent crossover or post-randomization medical care.

Depression

To determine the effect of genotype-guided drug therapy on depression symptoms in depression participants, we will conduct a subset analysis comparing change in depression T-scores from baseline to 3-month follow-up in the Intervention group subset with either CYP2D6 PM or UM or CYP2C19 PM, RM or UM phenotypes to the Control group subset with CYP2D6 PM or UM or CYP2C19 PM, RM, or UM phenotypes. Participants that have other CYP2D6 or CYP2C19 phenotypes will not be included. The baseline to 3-month change in depression T-scores of the Intervention CYP2D6 PM/UM or CYP2C19 PM/RM/UM group will be compared to the baseline to 3-month change in depression T-scores of the Control CYP2D6 PM/UM or CYP2C19 PM/RM/UM subgroup using a two-sided two-sample t-test with a type 1 error of 0.049. This primary analysis will be conducted according to a modified intent-to-treat design, with participants analyzed and endpoints attributed according to the treatment arm to which the participants were randomized, regardless of subsequent crossover or post-randomization medical care.

10.5 Analysis of the Secondary Endpoints

All Trials

To determine the effect of genotype-guided drug therapy on overall well-being and the well-being sub-domains (pain interference, physical function, sleep disturbance, social role and activities functioning, fatigue, anxiety, and depression) in acute pain, chronic pain, and depression participants, we will conduct an intent to treat analysis, comparing the well-being (or sub-domain) T-scores from the 6-month follow-up assessments in the Intervention group compared to the control group using a two-sided two-sample t-test or Mann Whitney test, as appropriate, stratified by participant population (acute pain, chronic pain, depression), with a type 1 error of 0.05.

Acute Pain

The effect of genotype-guided opioid therapy on secondary endpoints pain intensity, opioid usage, opioid misuse score, and mobility score will be assessed by comparing the Intervention group subset with CYP2D6 IM or PM phenotypes to the Control group subset with CYP2D6 IM or PM phenotypes using a two-sided two-sample t-test or Mann Whitney test, as appropriate, with a type 1 error of 0.05. The effect of genotype guided opioid therapy on the secondary

endpoint opioid persistence will be assessed by comparing the Intervention group subset with CYP2D6 IM or PM phenotypes the Control group subset with CYP2D6 IM or PM phenotypes using a test of two-proportions with an alpha of 0.05. Participants with other CYP2D6 phenotypes will not be included in these analyses.

Chronic Pain
The effect of genotype-guided opioid therapy on secondary endpoints pain intensity reduction and opioid misuse score will be assessed by comparing the Intervention group subset with CYP2D6 IM or PM phenotypes to the Control group subset with CYP2D6 IM or PM phenotypes using a two-sided two-sample t-test or Mann Whitney test, as appropriate, with a type 1 error of 0.05. The effect of genotype guided opioid therapy on the secondary endpoint clinically significant pain reduction, defined as a 30% decrease, will be assessed by comparing the Intervention group subset with CYP2D6 IM or PM phenotypes the Control group subset with CYP2D6 IM or PM phenotypes using a test of two-proportions with an alpha of 0.05. Participants that have other CYP2D6 phenotypes will not be included in these analyses.

Depression
The effect of genotype guided antidepressants therapy on the secondary endpoint depression remission will be assessed by comparing the Intervention group subset with CYP2D6 UM or PM or CYP2C19 UM, RM, or PM phenotypes the Control group subset with CYP2D6 UM or PM or CYP2C19 UM, RM, or PM phenotypes using a test of two-proportions with an alpha of 0.05. The effect of genotype guided antidepressants therapy on the secondary endpoints, medication adherence and medication side effect severity burden, will be assessed by comparing the Intervention group subset with CYP2D6 UM or PM or CYP2C19 UM, RM, or PM phenotypes the Control group subset with CYP2D6 UM or PM or CYP2C19 UM, RM, or PM phenotypes using a two-sided Mann Whitney test with an alpha of 0.05. Participants that do not have metabolizer phenotypes will not be included in these analyses.

10.6 Other Planned Analyses Sub-groups that will be considered:

1. All randomized participants
2. Metabolizer phenotypes: UMs, RMs, NMs, IMs, and PMs.
3. Pediatric participants
4. Adult participants
5. “Per-protocol” - participants with both an actionable phenotype and concordance between the recommended medication/dosage and the prescribed medication and/or dosage
6. Participants with an actionable phenotype, a discordant medication at baseline, and a concordant medication at the end of the study

Specified analyses:

All Trials
Medication side effects. The frequency and severity of medication side effects within each trial and trial arm will be summarized using standard descriptive statistics for discrete data (counts and percentages).

Acute Pain
Trends in endpoints. In addition to the primary and secondary endpoints described above, we will compare the time trends in the endpoints: pain scores, opioid usage, opioid misuse score, and mobility score at baseline, 1, 3, and 6 months among the CYP2D6 IM or PM subgroup,

using a repeated-measures mixed effect model or generalized repeated-measures mixed effect model, as appropriate. Since participants are randomized to Intervention and Control groups and not to the CYP2D6 IM or PM subgroup within Intervention and Control groups, there could be important differences in baseline characteristics between the two groups. For these reasons, we will conduct covariate-adjusted repeated measures mixed models that will account for differences in baseline characteristics that differ between the Intervention – CYP2D6 IM or PM subgroup and the Control – CYP2D6 IM or PM subgroup. Potential baseline characteristics may include age, sex, income, education, insurance, surgical procedure type. We will select the appropriate covariance matrix (e.g. compound symmetry, autoregressive, unstructured, or other covariance structure) based on the data.

Difference within the NM group. In the control arm participants, we will compare pain scores in participants with a CYP2D6 activity score >0.75 – 1 to participants with an activity scores >1-2 using a two-sided two-sample t-test or Mann Whitney test, as appropriate, with a type 1 error of 0.05. Additionally, we will use linear regression to adjust for differences in baseline characteristics.

Chronic Pain

Trends in endpoints. In addition to the primary and secondary endpoints described above, we will compare the time trends in the endpoints: pain control, pain reduction, clinically significant pain reduction, and opioid misuse score at baseline, 1, 3, and 6 months among the CYP2D6 IM or PM subgroup, using a repeated-measures mixed effect model or generalized repeated-measures mixed effect model, as appropriate. Since participants are randomized to Intervention and Control groups and not to the CYP2D6 IM or PM subgroup within Intervention and Control groups, there could be important differences in baseline characteristics between the two groups. For these reasons, we will conduct covariate-adjusted repeated measures mixed models that will account for differences in baseline characteristics that differ between the Intervention – CYP2D6 IM or PM subgroup and the Control – CYP2D6 IM or PM subgroup. Potential baseline characteristics may include age, sex, income, education, insurance, and medical conditions. We will select the appropriate covariance matrix (e.g. compound symmetry, autoregressive, unstructured, or other covariance structure) based on the data.

Difference within the NM group. In the control arm participants, we will compare pain scores and pain control in participants with a CYP2D6 activity score >0.75 – 1 to participants with an activity scores >1-2 using a two-sided two-sample t-test or Mann Whitney test, as appropriate, with a type 1 error of 0.05. Additionally, we will use linear regression to adjust for differences in baseline characteristics.

Depression

Trends in endpoints. In addition to the primary and secondary endpoints described above, we will compare the time trends in the endpoint depression symptom scores at baseline, 1, 3, and 6 months among the CYP2D6 PM or UM or CYP2C19 UM, RM or PM subgroup, using a repeated-measures mixed effect model. Since participants are randomized to Intervention and Control groups and not to the CYP2D6 PM or UM or CYP2C19 UM, RM or PM subgroup within Intervention and Control groups, there could be important differences in baseline characteristics between the two groups. For these reasons, we will conduct covariate-adjusted repeated measures mixed models that will account for differences in baseline characteristics that differ between the Intervention – CYP2D6 PM or UM or CYP2C19 UM, RM or PM subgroup and the Control – CYP2D6 PM or UM or CYP2C19 UM, RM or PM subgroup. Potential baseline

characteristics may include age, sex, income, education, insurance, medical conditions. We will select the appropriate covariance matrix (e.g. compound symmetry, autoregressive, unstructured, or other covariance structure) based on the data.

Difference within the NM group. In the control arm participants, we will compare depression scores and depression control in participants with a CYP2D6 activity score >0.75 – 1 to participants with an activity scores >1-2 using a two-sided two-sample t-test or Mann Whitney test, as appropriate, with a type 1 error of 0.05. Additionally, we will use linear regression to adjust for differences in baseline characteristics

Cost Effectiveness

Healthcare costs: In the Medicare fee-for-service or Medicaid enrollees, log-transformed overall expenditures (between index visit and 6 months follow-up) will be compared using a 2-sample t-test between the study arms (after ensuring that the variances in the log-scale are equal). Alternatively, cost comparisons will be made using appropriate regression models such as generalized linear models. For all participants, regardless of insurance type, we will estimate and aggregate participant-reported healthcare utilization during follow-up and assign cost for each item based on unit weighted mean cost estimates from the Medicare/ Medicaid data.

Within the Medicare/Medicaid population, we will compare estimated cost differences between genotype-guided and usual care based on billing records versus participant report to further validate the cost comparisons for the entire population. Cost for the index hospitalization for surgery participants will not be included in the cost estimate, but we will compare length of stay between groups as reference for hospitals regarding potential cost savings in capitation-based reimbursement schemes. All costs will be converted to 2021 $US using the chain-weighted Consumer Price Index (CPI).

Finally, we will compare the Medicare/Medicaid beneficiaries among our trial enrollees to a larger random sample of beneficiaries in the participating states and compare demographic and key clinical characteristics to explore representativeness of our study sample, evaluate the impact of attrition and loss to follow-up and make inferences about the generalizability of our results.

Cost utility: For the cost-utility analysis, the PROMIS-43 responses will be converted into utilities using previously validated crosswalks.[41] Cost and quality adjusted life year (QALY) estimates, adjusted for clustering within site and baseline utility for QALYs, will be obtained using appropriate regression models such as generalized linear models. The analysis of maximum likelihood parameter estimates will allow us to capture differences in cost and QALYs between the intervention arm and control arm of the trial. The Incremental cost-effectiveness ratio (ICER) will be calculated from costs and QALY. A bootstrapping approach will be used to characterize sampling uncertainty and calculate confidence intervals (CI) around the ICER estimate (2.5thand 97.5thpercentiles corresponding to the lower and upper bounds of the CI, respectively). This sampling uncertainty will be summarized using cost-effectiveness acceptability curves.

Details of these statistical analyses, along with other exploratory analyses will be described in the study SAP.

10.7 Interim Analyses

A single specified interim analysis is planned for each trial. To account for repeated significance testing of the accumulating data, the group sequential method of Lan and DeMets will be used as a guide for interpreting this interim analysis. Monitoring boundaries for the primary endpoint

will be based on a two-sided symmetric O'Brien-Fleming type spending function with an overall two-sided significance level of α=0.05. The O'Brien-Fleming approach requires large critical values early in the study but relaxes (i.e., decreases) the critical value as the trial progresses.

Acute Pain
The interim analysis of the acute pain primary endpoint will compare SIA scores at 10 days post-surgery among participants with *CYP2D6* IM or PM phenotypes in the Control arm to participants with *CYP2D6* IM or PM phenotypes in the intervention arm.

Chronic Pain
The interim analysis of the chronic pain primary endpoint, pain control at 3 months post return of PGx testing result to providers, will be compared between the participants with *CYP2D6* IM or PM phenotypes in the Control arm and the participants with *CYP2D6* IM or PM phenotypes in the intervention arm.

Depression
The interim analysis of the depression primary endpoint, depression control at 3 months post return of PGx testing result to providers, will compare the participants with *CYP2D6* PM/UM or *CYP2C19* PM, RM and UM phenotypes in the Control arm to the participants with *CYP2D6* PM/UM or *CYP2C19* PM/RM/UM phenotypes in the intervention arm.

The primary analysis is a modified intent to treat design and the analytical subgroup is defined by the results of the PGx testing. Because the drug metabolizer phenotypes for participants in the Control arm is determined around the time of the 6-month follow up assessments for all trials (Acute Pain, Chronic Pain, and Depression), only participants that have completed their 6-month assessments will be included in the interim analysis. To account for the delays in receiving genetic testing results, the interim analysis will be targeted to occur when approximately 50% of the participants have completed their 6-month assessments. If the interim analysis does not propose stopping for efficacy (p-value <0.0003 and the intervention has the larger reduction in the primary endpoints), then the conditional power to detect a significant result at the end of the trial will be estimated. The conditional power will be presented to the DSMB to facilitate discussion of whether the trial should be stopped for futility.

10.8 Handling of Missing Data

For all primary and secondary statistical analyses described above, multiple imputation will be used for all missing values except those due to participant death. To ensure that the missing at random assumption for multiple imputation is valid, we will compare baseline pain or depression scores and other patient characteristics in those lost to follow up to participants retained on their randomization assignment. We will also do sensitivity analyses where we compare results obtained with multiple imputation to those obtained without imputation. Missing values due to death will not be imputed and will therefore not be included in the analyses. For exploratory repeated measures analyses, missing data are easily handled as long as the missing at random assumption is valid. However, the missing at random assumption cannot be tested. Accordingly, for exploratory analyses using repeated measures mixed models, we will also conduct a sensitivity analysis where missing values are imputed using multiple imputation. The results with and without multiple imputation will be compared. More complete details of the handling of missing values under different circumstances will be described in the SAP.

10.9 Multiplicity

With the primary and various secondary endpoints that have been outlined, there is a multiplicity of analyses to be performed, which leads to an increased probability that at least one of the

comparisons could be "significant" by chance. Adjusting for the effects of the repeated significance testing for the multiplicity of secondary endpoints would require that very small significance levels be used for every comparison. Therefore, rather than adjusting for multiple comparisons, we will be conservative in the interpretation of the analyses, considering the degree of significance, and looking for consistency across endpoints. The nominal (unadjusted) p-value for each comparison will be reported to aid in the overall interpretation. We have also prespecified the primary and secondary outcome variables to avoid over-interpretation of strictly exploratory comparisons.

11. DATA MANAGEMENT

11.1 Data Entry and Record Keeping

ADOPT PGx data sources include: data collected from participant surveys, genetic testing results, data from the EHR, and CMS billing records. Data will be entered into a REDCap database maintained by the CC by trained and qualified personnel at each clinical site for baseline assessments and by the UF call center for follow-up assessments. Site staff will receive training on the use of the REDCap database. After staff are trained, they will receive a unique user identification and password to access data entry forms for their site. Access codes should not be shared and are non-transferable.

Laboratories will transfer genetic testing results to the IGNITE PTN CC and follow standard site-specific return of results procedures for providers. The genotype and result (i.e. metabolizer phenotype) for each participant will then be automatically imported to the participant's study database record from laboratory produced output files. To verify the accuracy of automatic import of results into the participant's records, laboratories will also transfer raw data in whatever format produced locally to the CC via secure file transfer after the first patient sample has been processed, and at other intervals as described in the MOP.

Sites will extract the relevant data from their EHR (see Section 7.7) and transfer of those data to the IGNITE PTN CC following procedures that will be specified in the MOP. Briefly, sites will be given a list of trial-specific information to extract for their ADOPT PGx participants, a common format that data should be converted to, and procedures for secure file transfer to the CC.

11.2 Data Element Definitions

<u>Clinically significant pain reduction</u>: Whether or not the pain reduction score is ≤ 0.7. If the pain reduction score is ≤ 0.7, clinically significant pain reduction is achieved, if pain reduction scores is > 0.7, clinically significant pain reduction is not achieved.

<u>Depression symptom control</u>: The change in the PROMIS Emotional Distress - Depression 8b or PROIMIS pediatric Depression 8a survey depression score from baseline to time t.

<u>Depression remission:</u> Whether or not the summed raw PROMIS emotional distress depression 8b survey responses are <= 16, which is equivalent to the participant responding "rarely" or "never" to most or all of the PROMIS emotional distress - depression 8b survey questions.

<u>Depression (T) score</u>: The T-score converted summed responses to the PROMIS emotional distress depression 8b survey. The T-score conversion is centered around 50 with standard deviation of 10.

<u>Depression severity:</u> The depression score converted to depression severity (none, mild, moderate, severe) based on the scoring guide for the PROMIS emotional distress depression 8b survey.

<u>Medication adherence:</u> The scored responses to the Voils Medication Adherence survey.

Medication concordance: Whether or not the medication the participant is prescribed after the intervention is concordant with the participants CYP2D6 and, for the depression trial, CYP2C19 phenotype.

Medication side effects severity burden: (Depression only) The sum of the scored severity (none = 0, mild = 1, moderate = 2, severe = 3) for common SSRI side effects.

Opioid misuse score: The T-score converted summed responses to the PROMIS prescription medication misuse 7a survey. The T-score conversion is centered around 50 with standard deviation of 10.

Opioid persistence: Defined as ≥1 opioid prescription refill between 90 days and 180 days after the surgical procedure.

Opioid usage: The average daily morphine equivalents in mg (MED) in past 7 days. MEDs will be calculated using data from the opioid usage survey and inpatient opioid usage immediate post-surgery collected from the EHR queries.

Overall well-being: Defined as the T-score converted summed responses to the PROMIS 43/PROMIS 37 pediatrics survey. The T-score conversion is centered around 50 with standard deviation of 10.

Pain control: The difference in pain intensity score at follow-up time t and the baseline pain intensity score.

Pain intensity score: The mean of average pain in the last 7 days, current pain, and worst pain in the last 7 days. Average pain, current pain, and worst pain will be collected using the 3-question PROMIS pain intensity scale.

Pain reduction score: The ratio of the pain intensity score (derived from the PROMIS pain intensity survey) at follow-up time t to the baseline pain intensity score.

Silverman integrative analgesic assessment (SIA) score: This is an integrated measure of pain and opioid usage, calculated as follows: pain and opioid usage are ranked, the ranks are converted to percentiles and linearly transformed such that the scores are centered at 0 and range -100 to 100. The pain scores and opioid usage transformed percentiles are summed to generate the SIA score that ranges -200 to 200. Negative values indicate low pain with minimal opioid usage, while positive values indicate higher pain with higher opioid usage. See Appendix A for additional details and supporting rationale.

11.3 Database Management and Quality Control of Data

The IGNITE PTN CC will develop and manage the ADOPT-PGx study database and perform internal database quality-control checks. The CC will conduct data audits throughout the course of the trial. These audits are intended to identify data errors, protocol deviations, failure of standardization, missing data, or inconsistencies. Any out-of-range values and missing or inconsistent key variables are flagged and addressed/answered at the site in real time during the data entry process.

The CC will periodically perform additional data quality checks in SAS. Clinical sites may also be given regular feedback directly to discuss issues identified by QC assessments.

11.4 Database Lock and Study Close Out

The end of the study is defined as the completion of the final participant follow-up. The designated central clinical research monitor with oversight by the CC will coordinate participating site close-out process according to the Clinical Monitoring Plan (See MOP). The CC will follow the database lock process in the study Data Management Plan.

11.5 Data Sharing

In accordance with the NIH Genomic Data Sharing policy, https://grants.nih.gov/grants/guide/notice-files/NOT-HG-19-024.html deidentified genotypes, linked phenotype, and clinical outcome data, excluding Medicare or Medicaid claims data, will be deposited in an NIH-designated data repository.

12. ETHICAL AND HUMAN SUBJECTS CONSIDERATIONS

12.1 Institutional Review Board/Ethics Committee Review

This study will be initiated after all required documentation has been reviewed and approved by the central IRB according to national and international regulations. All participating sites will be required to have central IRB approval prior to activation. The CC will be responsible for the coordination of all IRB activities.

12.2 Use and Disclosure of Protected Health Information (HIPAA)

For clinical trial sites, an authorization for the use and disclosure of protected health information (PHI) under the HIPAA Privacy Rule [45 CFR § 164.102 *et seq*] will be obtained from every trial participant prior to, or at the time of, enrollment. HIPAA Authorization may either be a separate form or included in the study ICF, dependent upon local requirements. It will be presented to, and signed by, the subject at the same time as the Informed Consent Form (ICF). See Section 6.4 Participant Consent Process .

12.3 Confidentiality and Privacy

Participant confidentiality will be maintained throughout the clinical study in a way that ensures the information can always be tracked back to the source data. For this purpose, a unique subject identification (ID number) will be used that allows identification of all data reported for each subject. Subject information collected in this study and all records will be kept confidential and the subject's name will not be released by study staff at any time.

Clinical data will be entered into a data entry system provided by the CC. The data system includes password protection and internal quality checks, such as automatic range checks to identify data that appear inconsistent, incomplete, or inaccurate. All data collection and storage devices will be password protected with a strong password and all sensitive research information on portable devices will be encrypted. Access to identifiable data will be limited to members of the study team and all identifiers, data, and keys will be placed in separate, password protected/encrypted files and each file will be stored in a different secure location. If it is necessary to use portable devices for initial collection of identifiers, the data files will be encrypted and the identifiers moved to a secure system as soon as possible. The portable device(s) will be locked up in a secure location when it is not in use.

12.4 Publication and Data Sharing Policies

The IGNITE PTN will have a Publications and Presentations Committee (PPC) with the primary responsibility for coordinating, monitoring, and reviewing all publications and presentations resulting from IGNITE PTN studies. In addition, the PPC will oversee the review, approval, and supervision of the secondary analyses and ancillary studies that are conducted within the Network. The goal of the PPC is to facilitate dissemination of the maximum amount of information from these studies in a scientifically sound and ethically responsible fashion in accordance with the unique nature of the IGNITE PTN mission. The IGNITE PTN Coordinating Center will draft a PPC charter in collaboration with NHGRI, PPC, and the IGNITE PTN which specifies the publication policies and procedures. The primary outcomes from each trial, Acute Pain, Chronic Pain, Depression, will be published as separate publications.

The sharing of datasets will be performed per DCRI SOPs and requirements for NIH policy for

data sharing, and guidelines for NIH Data Set Preparation. The de-identified and anonymized data, excluding Medicare and Medicaid claims data, and documentation in standardized formatswill be made available in an NIH-designated data repository for sharing to the larger scientific community. Requested unrestricted data may be made available after database lock to parties who sign a data sharing agreement, which stipulates that data must be: 1) used solely for research purposes, 2) properly acknowledged in resulting publications, 3) kept confidential and inaccessible to third parties, and 4) destroyed or returned after analyses are completed. Additionally, users must agree not to use data to identify individual participants.

13. PROTOCOL DEVIATIONS AND VIOLATIONS

A protocol deviation is defined as an event where the Investigator or site personnel did not conduct the study according to the protocol or the Investigator Agreement. A protocol violation is an intentional act in which the protocol is not followed. (See Protocol Section 8 for IRB reporting timelines)

Protocol deviations and violations will be reported to the IRB if they affect:

- subject rights and welfare
- affects subject safety
- affects the integrity of study data
- affects the subject's willingness to continue in the study
- is specifically requested by a government agency, internal/external auditor, medical monitor, or the IRB.

REFERENCES

1. Smith, D.M., et al., *CYP2D6-guided opioid therapy improves pain control in CYP2D6 intermediate and poor metabolizers: a pragmatic clinical trial.* Genet Med, 2019.
2. Gaskin, D.J. and P. Richard, *The economic costs of pain in the United States.* J Pain,2012. **13**(8): p. 715-24.
3. *Relieving Pain in America: A Blueprint for Transforming Prevention, Care, Education, and Research.* Mil Med, 2016. **181**(5): p. 397-9.
4. Aubrun, F., et al., *Relationships between measurement of pain using visual analog scoreand morphine requirements during postoperative intravenous morphine titration.* Anesthesiology, 2003. **98**(6): p. 1415-21.
5. Bruehl, S., et al., *Personalized medicine and opioid analgesic prescribing for chronic pain: opportunities and challenges.* J Pain, 2013. **14**(2): p. 103-13.
6. Guy, G.P., Jr., et al., *Vital Signs: Changes in Opioid Prescribing in the United States,2006-2015.* MMWR Morb Mortal Wkly Rep, 2017. **66**(26): p. 697-704.
7. *U.S. Prescribing Rate Maps*. 2017 2017-10-5]; Available from: https://www.cdc.gov/drugoverdose/maps/rxrate-maps.html.
8. Levy, B., et al., *Trends in Opioid Analgesic-Prescribing Rates by Specialty, U.S., 2007-2012.* Am J Prev Med, 2015. **49**(3): p. 409-13.
9. Informatics, M.I.f.H. *Medication use and athe shifting cost of healthcare - a review of use of medicine in teh United States in 2013*. [cited 2017; Available from: https://democrats-oversight.house.gov/sites/democrats.oversight.house.gov/files/documents/IMS-Medicine%20use%20and%20shifting%20cost%20of%20healthcare.pdf.
10. Crews, K.R., et al., *Clinical Pharmacogenetics Implementation Consortium guidelines for cytochrome P450 2D6 genotype and codeine therapy: 2014 update.* Clin PharmacolTher, 2014. **95**(4): p. 376-82.
11. Ciszkowski, C., et al., *Codeine, ultrarapid-metabolism genotype, and postoperative death.* N Engl J Med, 2009. **361**(8): p. 827-8.
12. Gasche, Y., et al., *Codeine intoxication associated with ultrarapid CYP2D6 metabolism.* N Engl J Med, 2004. **351**(27): p. 2827-31.
13. Kelly, L.E., et al., *More codeine fatalities after tonsillectomy in North American children.* Pediatrics, 2012. **129**(5): p. e1343-7.
14. Koren, G., et al., *Pharmacogenetics of morphine poisoning in a breastfed neonate of acodeine-prescribed mother.* Lancet, 2006. **368**(9536): p. 704.
15. Orliaguet, G., et al., *A case of respiratory depression in a child with ultrarapid CYP2D6 metabolism after tramadol.* Pediatrics, 2015. **135**(3): p. e753-5.
16. Adams, E.H., et al., *A comparison of the abuse liability of tramadol, NSAIDs, and hydrocodone in patients with chronic pain.* J Pain Symptom Manage, 2006. **31**(5): p.465-76.
17. Preston, K.L., D.R. Jasinski, and M. Testa, *Abuse potential and pharmacological comparison of tramadol and morphine.* Drug Alcohol Depend, 1991. **27**(1): p. 7-17.
18. Taylor, W.D., *Clinical practice. Depression in the elderly.* N Engl J Med, 2014. **371**(13): p. 1228-36.

19. Noordam, R., et al., *Identifying genetic loci associated with antidepressant drug responsewith drug-gene interaction models in a population-based study.* J Psychiatr Res, 2015. **62**: p. 31-7.
20. Thase, M.E., et al., *Remission rates following antidepressant therapy with bupropion or selective serotonin reuptake inhibitors: a meta-analysis of original data from 7 randomized controlled trials.* J Clin Psychiatry, 2005. **66**(8): p. 974-81.
21. Singh, A.B., *Improved Antidepressant Remission in Major Depression via a Pharmacokinetic Pathway Polygene Pharmacogenetic Report.* Clin PsychopharmacolNeurosci, 2015. **13**(2): p. 150-6.
22. Hall-Flavin, D.K., et al., *Utility of integrated pharmacogenomic testing to support the treatment of major depressive disorder in a psychiatric outpatient setting.* PharmacogenetGenomics, 2013. **23**(10): p. 535-48.
23. Bradley, P., et al., *Improved efficacy with targeted pharmacogenetic-guided treatment of patients with depression and anxiety: A randomized clinical trial demonstrating clinical utility.* J Psychiatr Res, 2018. **96**: p. 100-107.
24. Bousman, C.A., et al., *Pharmacogenetic tests and depressive symptom remission: a meta- analysis of randomized controlled trials.* Pharmacogenomics, 2019. **20**(1): p. 37-47.
25. Winner, J.G., et al., *A prospective, randomized, double-blind study assessing the clinicalimpact of integrated pharmacogenomic testing for major depressive disorder.* Discov Med, 2013. **16**(89): p. 219-27.
26. Perez, V., et al., *Efficacy of prospective pharmacogenetic testing in the treatment of major depressive disorder: results of a randomized, double-blind clinical trial.* BMC Psychiatry, 2017. **17**(1): p. 250.
27. Hicks, J.K., et al., *Clinical Pharmacogenetics Implementation Consortium (CPIC) Guideline for CYP2D6 and CYP2C19 Genotypes and Dosing of Selective SerotoninReuptake Inhibitors.* Clin Pharmacol Ther, 2015. **98**(2): p. 127-34.
28. Administration, U.S.F.a.D. *Drug Development and Drug Interactions: Table of Substrates, Inhibitors and Inducers*. 2017 11/14/2017 [cited 2019; Available from: https://www.fda.gov/drugs/drug-interactions-labeling/drug-development-and-drug-interactions-table-substrates-inhibitors-and-inducers.
29. Zwisler, S.T., et al., *Impact of the CYP2D6 genotype on post-operative intravenousoxycodone analgesia.* Acta Anaesthesiol Scand, 2010. **54**(2): p. 232-40.
30. Andreassen, T.N., et al., *Do CYP2D6 genotypes reflect oxycodone requirements for cancer patients treated for cancer pain? A cross-sectional multicentre study.* Eur J ClinPharmacol, 2012. **68**(1): p. 55-64.
31. Lemberg, K.K., et al., *Does co-administration of paroxetine change oxycodone analgesia: An interaction study in chronic pain patients.* Scand J Pain, 2010. **1**(1): p. 24-33.
32. Cella, D., et al., *PROMIS((R)) Adult Health Profiles: Efficient Short-Form Measures of Seven Health Domains.* Value Health, 2019. **22**(5): p. 537-544.
33. Pilkonis, P.A., et al., *An Item Bank for Abuse of Prescription Pain Medication from the Patient-Reported Outcomes Measurement Information System (PROMIS(R)).* Pain Med,2017. **18**(8): p. 1516-1527.
34. Hays, R.D., et al., *Upper-extremity and mobility subdomains from the Patient-Reported Outcomes Measurement Information System (PROMIS) adult physical functioning itembank.* Arch Phys Med Rehabil, 2013. **94**(11): p. 2291-6.

35. Quinn, H., et al., *Using item response theory to enrich and expand the PROMIS pediatricself report banks.* Health and quality of life outcomes, 2014. **12**: p. 160.
36. Pilkonis, P.A., et al., *Item banks for measuring emotional distress from the Patient-Reported Outcomes Measurement Information System (PROMIS(R)): depression, anxiety, and anger.* Assessment, 2011. **18**(3): p. 263-83.
37. Forrest, C.B., et al., *Development and Evaluation of the PROMIS((R)) Pediatric Positive Affect Item Bank, Child-Report and Parent-Proxy Editions.* J Happiness Stud, 2018. **19**(3): p. 699-718.
38. Rush, A.J., et al., *The Inventory of Depressive Symptomatology (IDS): psychometricproperties.* Psychol Med, 1996. **26**(3): p. 477-86.
39. Voils, C.I., et al., *Content Validity and Reliability of a Self-Report Measure of Medication Nonadherence in Hepatitis C Treatment.* Dig Dis Sci, 2019. **64**(10): p. 2784-2797.
40. *PROMIS (Patient-Reported Outcomes Measurement Information System)*. 2020; Available from: http://www.healthmeasures.net/explore-measurement-systems/promis.
41. Hartman, J.D. and B.M. Craig, *Comparing and transforming PROMIS utility values to the EQ-5D.* Qual Life Res, 2018. **27**(3): p. 725-733.

14. APPENDICES

14.1 APPENDIX A. SIA SCORE BACKGROUND AND RATIONALE

For the primary endpoint, we propose to use a composite score that captures both opioid consumption (MED) and pain intensity. This is based on preliminary data from a University of Florida (UF) pilot study in which CYP2D6 genotype-guided opioid prescribing after arthroplasty surgery was compared to a usual care approach (ClinicalTrials.gov Identifier: NCT03534063). While not powered to detect differences in clinical endpoints, preliminary results from 174 patients showed similar pain intensity at 2-week post-surgery between genotype-guided and usual care groups, but significantly lower MMEs in the genotype-guided group. The combination of lower opioid consumption with similar pain intensity indicates better pain control in the genotype-guided arm. However, assessing opioid consumption and pain intensity as two separate variables fails to characterize the inter-individual differences in opioid use as pain intensity changes over time, which is why we believe a composite endpoint that integrates the two is justifiable.

The Silverman Integrating approach (SIA) score has been shown to provide superior statistical power with appropriate control of type 1 error compared to methods that integrate pain score and post-operative opioid consumption.[1, 2] When comparing the SIA score between genotype-guided and usual care arms in the UF study described above, the score indicated lesser pain despite fewer opioids consumed in the genotype-guided arm compared with greater pain despite more opioids consumed in the usual care group (p=0.07). To date, at least 11 clinical trials have used the SIA score in the post-operative setting.[3-13] While the majority utilized patient-controlled analgesia pumps, a pain expert at UF proposes the SIA score applicability warrants consideration in an acute post-surgical pain population. The SIA score is referenced in recommendations by the Initiative on Methods, Measurement, and Pain Assessment in Clinical Trials (IMMPACT), which aims to develop consensus recommendations for design and execution of clinical trials of pain management [14], as well as in a systematic review of predictive experimental pain studies.[15] However, both acknowledge that the SIA score is rarely used in pain research and that additional research is needed on its utility. While this approach is not part of current consensus recommendations for pain trials, consensus bodies have expressed interest in its utility. Importantly, it allows us to jointly assess two important, interlinked parameters in the setting of acute post-surgical pain – both opioid use and pain control. While we could focus on MMEs as the primary endpoint based on the pilot data described above, we believe that MME alone, without knowing the pain control, is difficult to interpret clinically, and thus suboptimal.

References

1. Silverman, D.G., T.Z. O'Connor, and S.J. Brull, Integrated assessment of pain scores and rescue morphine use during studies of analgesic efficacy. Anesth Analg, 1993. 77(1): p. 168-70.
2. Dai, F., et al., Integration of pain score and morphine consumption in analgesic clinical studies. J Pain, 2013. 14(8): p. 767-77 e8.
3. Ahdieh, H., et al., Efficacy of oxymorphone extended release in postsurgical pain: a randomized clinical trial in knee arthroplasty. J Clin Pharmacol, 2004. 44(7): p. 767-76.
4. Gambling, D., et al., A comparison of Depodur, a novel, single-dose extended-release epidural morphine, with standard epidural morphine for pain relief after lower abdominal surgery. Anesth Analg, 2005. 100(4): p. 1065-74.

5. Gilron, I., et al., A placebo-controlled randomized clinical trial of perioperative administration of gabapentin, rofecoxib and their combination for spontaneous and movement-evoked pain after abdominal hysterectomy. Pain, 2005. 113(1-2): p. 191-200.
6. Katz, J., et al., High dose alfentanil pre-empts pain after abdominal hysterectomy. Pain, 1996. 68(1): p. 109-18.
7. Liu, S., et al., Effects of epidural bupivacaine after thoracotomy. Reg Anesth, 1995. 20(4): p. 303-10.
8. Liu, S., et al., Intravenous versus epidural administration of hydromorphone. Effects on analgesia and recovery after radical retropubic prostatectomy. Anesthesiology, 1995. 82(3): p. 682-8.
9. Reinhart, D.J., et al., Postoperative analgesia after peripheral nerve block for podiatric surgery: clinical efficacy and chemical stability of lidocaine alone versus lidocaine plus clonidine. Anesth Analg, 1996. 83(4): p. 760-5.
10. Reinhart, D.J., et al., Postoperative analgesia after peripheral nerve block for podiatric surgery: clinical efficacy and chemical stability of lidocaine alone versus lidocaine plus ketorolac. Reg Anesth Pain Med, 2000. 25(5): p. 506-13.
11. Romundstad, L., et al., Methylprednisolone reduces pain, emesis, and fatigue after breast augmentation surgery: a single-dose, randomized, parallel-group study with methylprednisolone 125 mg, parecoxib 40 mg, and placebo. Anesth Analg, 2006. 102(2): p. 418-25.
12. Therasse, E., et al., Percutaneous biliary drainage: clinical trial of analgesia with interpleural block. Radiology, 1997. 205(3): p. 663-8.
13. Wininger, S.J., et al., A randomized, double-blind, placebo-controlled, multicenter, repeat-dose study of two intravenous acetaminophen dosing regimens for the treatment of pain after abdominal laparoscopic surgery. Clin Ther, 2010. 32(14): p. 2348-69.
14. Cooper, S.A., et al., Research design considerations for single-dose analgesic clinical trials in acute pain: IMMPACT recommendations. Pain, 2016. 157(2): p. 288-301.
15. Werner, M.U., et al., Prediction of postoperative pain: a systematic review of predictive experimental pain studies. Anesthesiology, 2010. 112(6): p. 1494-502.

14.2 APPENDIX B. TABLE OF MINIMUM REQUIRED VARIANTS

Gene	Allele	Variant	dbSNP
**CYP2C19**	*2	681G>A	rs4244285
	*3	636G>A	rs4986893
	*4	1A>G	rs28399504
	*6	395G>A	rs72552267
	*8	358T>C	rs41291556
	*17 (also *4 haplotype [*4B])	-806C>T	rs12248560
**CYP2D6**	*2	2850C>T	rs16947
	*2	4180G>C	rs1135840
	*3	2549delA	rs35742686
	*4	1846G>A	rs3892097
	*5	CYP2D6 deleted	
	*6	1707delT	rs5030655
	*8	1758G>T	rs5030865(A)
	*9	2615_2617delAAG	rs5030656
	*10 (also *36 gene conversion)	100C>T	rs1065852
	*17	1023C>T	rs28371706
	*29 (also *70)	3183G>A	rs59421388
	*41	2988G>A	rs28371725
	1XN	copy number	
	2XN	copy number	
	4XN	copy number	
	9XN	copy number	
	17XN	copy number	
	29XN	copy number	
	41XN	copy number	

14.3 APPENDIX C. CYP2D6 INHIBITORS AS DEFINED BY THE FDA GUIDENCE ON DRUG INTERACTIONS

As of 3/6/20:

Strong Inhibitors	bupropion, fluoxetine, paroxetine, quinidine, terbinafine
Moderate Inhibitors	abiraterone, cinacalcet, duloxetine, lorcaserin, mirabegron

A Depression and Opioid Pragmatic Trial in Pharmacogenetics

(ADOPT PGx)

Protocol Numbers

Coordinating Center IRB: PRO00104948

Single IRB: PRO00105183

Principal Investigators:

Julie A. Johnson, Pharm. D.

Larisa Cavallari, Pharm. D.

Josh F. Peterson, MD, MPH

Kerri Cavanaugh, MD MHS

Todd C. Skaar, Ph.D.

Paul R. Dexter, MD

Statistical Investigator: Hrishikesh Chakraborty, DrPH

NIH Program Officer: Simona Volpi, PhD

Funded by: National Human Genome Research Institute

Version Number: Final v2

11 February 2020

Amendment 1: 28 April 2020

Amendment 2: 03 June 2020

Amendment 3: 17 August 2020

Amendment 4: 20 November 2020

Amendment 5: 17 June 2021

Amendment 6: 24 August 2023

Amendment 7: 27 September 2023

Amendment 8: 23 May 2024

PROTOCOL VERSION AND AMENDMENT TRACKING

Version Number/ Affected Section(s)	Summary of Revisions Made	Approval Date
Blinding Section 4.2	UF Pharmacy Call Center personnel administration of the final 6 month survey may reveal study arm randomization	5/18/2020
Interventions/Treatments Section 7.1	Recent FDA update of strong and moderate inhibitors, Appendix C	5/18/2020
Interventions/Treatments Section 7.1, Table 2 CYP2D6 allele to activity score	Allele *10 activity value change	6/15/2020
Interventions/Treatments Section 7.1, Table 3 CYP2D6 phenotype by Activity Score	Inferred CYP2D6 phenotype, Intermediate metabolizer, definition differences from current CPIC guidelines and may be different from what appears in the lab reported phenotypes	6/15/2020
Interventions/Treatments Section 7.1, Table 5 CDS summary for acute and chronic pain treatments	Phenotype - normal defined as activity score >0.75 and ≤ 2.0<br><br>Phenotype - intermediate defined as activity score >0 and ≤ 0.75	6/15/2020
Data Collection from CMS and State Medicaid Agencies Table 8 Data Collection Schedule	Adjustments to trial arm collection time points	6/15/2020
Analysis of the Primary Endpoint 10.4	Text added for pediatric depression T score conversions	6/15/2020
Cover Page	Added Principal Investigators, Larisa Cavallari, Pharm. D. and Sara Van Driest, MD. PhD.	9/15/2020

PROTOCOL VERSION AND AMENDMENT TRACKING

Version Number/ Affected Section(s)	Summary of RevisionsMade	Approval Date
Randomization 4.1	Removed text: … and revealed after baseline dataare collected so study assignment does not impactthe baseline survey responses.	9/15/2020
Safety Assessment and Monitoring 8	Deleted text…This is an observational study that doesnot include a drug or device intervention.	9/15/2020
Analysis of the Primary Endpoint 10.4	Depression – text deleted …Pediatric depression T- scores will be converted to adult depression T-scores using a published crosswalk linking the scores from the twosurveys.<br><br>Added text: Pediatric depression T-scores will be transformed to the adult scaleusing a published crosswalk for a combined analysis.	9/15/2020
Specimen Collection Section 7.4	Clarification that DNA specimen collection should bedone after consenting and completed prior to randomization	12/22/2020
Throughout the Document	Removal of Quick Inventory of Depressive Symptomatology (QIDS) depression scale and replaced with Patient Health Questionnaire-8 (PHQ-8) depression scale.	7/2/2021
Protocol Synopsis-Inclusion Criteria<br>Section 1<br><br>Inclusion Criteria<br>Section 5.1	Acute Pain:<br>• Removal of “Elective/planned surgery patients with an upcoming pre-surgery visit”<br>• Removal of “as this is the minimum age at which proposed outcome measures (PROMIS) are validated without parent proxy” at age inclusion<br>• Added clarifying text for targeted opioids for Elective/planned surgery types<br><br>Chronic Pain:<br>• Added clarifying text for primary	7/2/2021

	clinics definition and for treatment of chronic pain with specific opioids<br>• Removed medical diagnosis codes for pain diagnosis, and symptoms<br><br>Depression:<br>• Removal of “as this is the minimum age at which proposed outcome measures (PROMIS) are validated without parent proxy” at age inclusion<br>• Added clarifying text for psychiatry and primary clinics definition and depression diagnosis<br>• Removed medical diagnosis codes for depression	
Protocol Synopsis-Exclusion Criteria<br>Section 1<br><br>Exclusion Criteria<br>Section 5.2	Trial Wide:<br>• Include “incarcerated” as an exclusion example for “Are institutionalized or too ill to participate (i.e. mental or nursing home facility)<br>• Removal of “Plan to move out of the area within 6 months of enrollment”<br><br>Acute Pain:<br>• Removal of “Plan to move out of the area within 6 months of enrollment”<br>• Removal of “Adults with a similar, previous surgery in whom pain control is well defined and a genotype guided approach would not likely be followed”<br><br>Chronic Pain:<br>• Addition of “Plan to move out of the area within 6 months of enrollment”<br>• Addition of “Currently taking daily opioids other than tramadol, codeine or hydrocodone<br><br>Depression:<br>• Addition of “Plan to move out of the area within 6 months of enrollment”<br>• Removal of medical diagnosis codes for psychotic, neurocognitive, cognitive developmental delay or disability, seizure and bipolar disorders<br>• Removal of antipsychotic medications<br>• Removal of “Depression secondary to substance abuse disorder or general medical condition”	7/2/2021

Protocol Synopsis-Secondary Endpoints<br>Section 1<br><br>Secondary Endpoints<br>Section 3.2	Depression:<br>• Updated the secondary endpoints using PHQ-8 depression scale	
Provider Recruitment and Assent<br>Section 6.1	Acute Pain:<br>• Refined the text: "Providers with a predominant use of codeine, tramadol and hydrocodone for pain control before and during the 10-day primary endpoint may be approached by qualified study personnel. Providers will be notified per site institutional guidelines that their patients may be contacted by study site recruiters to participate. Surgical procedures where there is persistent pain at the10-day primary endpoint should be prioritized for inclusion in the study."<br><br>Chronic Pain:<br>• Removed text "approached by qualified study personnel, and notified that their patients will be contacted by study site recruiters to participate."<br>• Added text: ""may be approach by qualified personnel. Providers will be notified per site institutional guidelines that their patients may be contacted by study site recruiters to participant."<br><br>Depression:<br>• Removed text "They then will be notified that their patients will be contacted by study site recruiters to participate."<br>• Added text "and may be approached by qualified study personnel, and given the opportunity to participate as a study provider. Providers will be notified per site institutional guidelines that their patients may be contacted by study site recruiters to participate."	7/2/2021

Baseline Participant Assessments<br>Section 7.2	Baseline:<br>• Removed text “baseline data will be collected”<br>• Added text “survey data will be collected”<br><br>Past Medical History:<br>• Removed text “in the medical record.”<br>• Added text: See MOP for details.”<br><br>Baseline medication:<br>• Added text “the EHR and/or participant self-report.”	7/2/2021
Follow-up Participant Assessment All Trials<br>Section 7.3	All Trials: Medications:<br>• Removed text “patient surveys at the primary endpoint time points: at baseline, 10-days and 1-month, 3-months and 6-months for the acute pain participants, and baseline, 1-month, 3-months and 6-months for the chronic pain and depression participants.”<br>• Added text ““patient surveys at baseline and the primary endpoint time points: 10-days for Acute Pain and 3-months for Depression and Chronic pain.”<br><br>Acute Pain:Opioid Consumption Questionnaire:<br>• Removed text<br>o “the date the prescription was filled”<br>o “Study participants will also be asked to read the tablet strength from their prescription bottle but this will be verified by local study coordinators by reviewing the EHR for the prescription written.”<br><br>Depression:<br>• Removed reference text to the QIDS: “Quick Inventory of Depressive Symptomatology: A 16 item survey completed by the participant assessing depression symptoms[38]. This survey will be administered at baseline, 3 months, and 6 months post return of results.<br>• Added reference text for the PHQ-8: “*Patient Health Questionnaire-8* – An	

	8 item survey completed by the participant assessing depression symptoms over the last two weeks. This survey will be administered at baseline, 3 months, and 6 months post return of results[38].	
Data Collection from Electronic Health Record Section 7.8	• Removed text: “Data from the local EHR will be used to assess participant’s medications, medication changes, medication discontinuations, and, if available, reasons for medication discontinuations”<br>• Added text: “Data collected from the local EHR will include prescription information and encounters including clinic visits, hospitalizations, and emergency department visits and associated diagnoses”	7/2/2021
Table 8. Data Collection Schedule	• Updated the Data Collection Timing for the Acute Pain and Immediate Chronic Pain and Depression arms. Including timings for Delayed Chronic Pain and Depression arms<br>• Updated the data collection schedule for the Assessment of Prescriptions	7/2/2021
Safety Assessment and Monitoring-Events of Interest Section 8.2	Redefined the text to:<br>• “Participant reported emergency department visits and hospitalizations will be collected in the 1, 3, and 6-month follow-up participant surveys. EHR will be used as an additional source for emergency department visits and hospitalizations, up to approximately 12 after the last patient is randomized. For the details of collecting EHR data, see Protocol section 7.8 and MOP. EHR results will be sent to the CC via secure data transfer and formatted for data analyses.”	7/2/2021
Statistical Analysis Plan and Sample Size: Analysis of Secondary Endpoints Section 10.5	Depression:<br>• Added text “and achieving 5% reduction in PHQ-8 scores “and “PHQ-8 scores”	7/2/2021

Data Management: Data Entry and Record Keeping Section 11.1	• Removed text: "automatically " and "To verify the accuracy of automatic import of results into the participant's records, laboratories will also transfer raw data in whatever format produced locally to the CC via secure file transfer after the first patient sample has been processed and at other intervals as described in the MOP."<br>• Added text: "The accuracy of the import of laboratory results will be verified by comparing the results recorded in the study database to an external record of the result for a subset of the study participants. See MOP for details."	7/2/2021
Data Management: Data Element Definitions Section 11.2	Depression Remission:<br>• Added text: B. Whether or not the PHQ-8 scores are ≤ 4, which is equivalent to depression severity being none-mild."<br>• Added term definition: "Depression (PHQ-8) score: The sum of the responses to the PHQ-8 survey, range from 0 to 24."<br>• Removed text in Opioid Usage: "and post discharge inpatient opioid usage immediate post-surgery collected from EHR queries"	7/2/2021
References	Removed reference for QIDS depression scale and added in reference for PHQ-8 depression scale.	7/2/2021
Title page; Protocol Version and Amendment Tracking; Investigator Statement; Abbreviations; Protocol Synopsis;	• Reformatted the entire document and updated page numbers<br>• Title page updates and PI names<br>• Protocol Version and Amendment Tracking -- Added Amendment 6 change information<br>• Investigator_Statement–updated protocol version date<br>• Abbreviations corrected typo<br>• Protocol Synopsis – Study Population updated the enrollment numbers to reflect the update milestones	8/30/23

Updates found in Protocol Clarification Memo #2-28Aug2021 in Protocol Synopsis, Study Population, and Inclusion/Exclusion Criteria Section 5 and Study Procedures	• Updated Inclusion criteria for Depression:<br>o Remove "the last" from "Evidence of depressive symptoms for at least the last 3 months based on patient interview or documentation in electronic health records"<br>• Updated Exclusion criteria:<br>o Add to Trial wide: Any other medical, behavioral, or developmental condition that in the opinion of the investigator may confound study data/assessments<br>o Add to Chronic Pain: Include "for treatment of pain" to "Currently taking daily opioids other than tramadol, codeine, or hydrocodone for treatment of pain"<br>o Add to Chronic Pain: Include "Using a pain pump"<br>• Updated text for baseline participant assessments in Section 7.2	
Updates found in Protocol Clarification Memo #3-22Oct2021 in Protocol Synopsis-Inclusion/Exclusion Criteria and section 5.2 Exclusion Criteria	• Updated Exclusion criteria: removal of "Undergoing a laparoscopic surgery"	8/30/23
Section 7 Study Procedures	• Section 7.1 Interventions/Treatments: Table 2 Corrected a typo in the table<br>• Section 7.2: Baseline medications-Simplified the text by removing "Additionally, participant prescription and over the counter medications may be collected using patient surveys." And adding "See MOP for details."<br>• Section 7.3: Follow-up Participant Assessments All Trials Medications: Removed "prescription and over the counter"<br>• Section 7.4: Specimen Collection-corrected verb tense.<br>• Section 7.8: Data Collection from the Electronic Health Record-corrected text from "baseline" to "time-zero".<br>• Section 7.9 Table 8. Data Collection Schedule-- Reverted the Table 8 to	8/30/23

	the prior version—removed the specific timing from the last amendment	
Section 8: Safety Assessment and Monitoring	• Revised the language to reflect what type of Adverse Device Effect (ADE) and unanticipated Adverse Device Effect (UADE) events to be reported to the IRB based on the trial regulated under an Abbreviated Investigational Device Exemption.<br>• Section 8.2: Event of Interest: clarified the text to “EHR may be used…” and the timing of the data collection	8/30/23
Section 9: Medicare and Medicaid Claims Data Collection and Analysis	• Added in clarifying text for the Medicare and Medicaid data collection and analysis.	8/30/23
Section 10: Statistical Analysis Plan and Sample Size	• Section 10.1: Sample Size Determination: Added language to define the mITT population and updated the enrollment numbers to reflect the update milestones<br>• Table 9: Sample size calculations: updated the numbers	8/30/23
Title page; Protocol Version and Amendment Tracking; Investigator Statement;	• Title page updates<br>• Protocol Version and Amendment Tracking -- Added Amendment 7 change information<br>• Investigator Statement–updated protocol version date	10/9/23
Section 6.3: Participant Discontinuation/Withdrawal from the Study	• Removal of “that is ≥ 3 months”.	10/9/23
Title page; Protocol Version and Amendment Tracking; Investigator Statement;	• Title page updates<br>• Protocol Version and Amendment Tracking -- Added Amendment 8 change information<br>• Investigator Statement–updated protocol version date	
Throughout the Document	• Corrected 4 instances of ADOPT-PGx to ADOPT PGx	

Section 10.6: Other Planned Analyses	• Added a couple of primary research exploratory analyses<br>• Updated exploratory endpoints or comparison of endpoints<br>• Updated sub-groups	
Section 12.3: Confidentiality and Privacy	• Updated language to reflect what is occurring in the study. Removed the text: and all identifiers, data, and keys will be placed in separate, password protected/encrypted files and each file will be stored in a different secure location.	
Section 12.4: Publication and Data Sharing Polices	• Updated language from “The de-identified **and** anonymized data” to “The de-identified **or** anonymized data”	

INVESTIGATOR STATEMENT

By signing below, I agree to the conditions relating to this trial as set out in this protocol, Amendment 8 dated 23 May, 2024.

I agree to conduct this clinical trial according to Good Clinical Practice (ICH GCP) and European Regulatory Requirements.

I fully understand that any changes instituted by me without previous discussion with the IGNITE PTN Coordinating Center or their designated representative constitute a violation of the protocol.

I agree to adhere to the protocol in all circumstances other than where necessary to protect the well-being of the subject.

Principal Investigators' Signatures

Name:________________________________

Signature:____Date:________________________

Name:________________________________

Signature:____Date:________________________

Name:________________________________

Signature:____Date:________________________

Name:________________________________

Signature:____Date:________________________

ABBREVIATIONS

ADOPT PGx	A Depression and Opioid Pragmatic Trial in Pharmacogenetics
AE	Adverse event
CC	Coordinating center
CDS	Clinical decision support
CLIA	Clinical Laboratory Improvement Amendments
CMP	Clinical Monitoring Plan
CMS	Centers for Medicare and Medicaid Services
CPIC	Clinical Pharmacogenetics Implementation Consortium
CRF	Case Report Form
DCRI	Duke Clinical Research Institute
DEA	Drug Enforcement Administration
DoB	Date of birth
DSMB	Data Safety Monitoring Board
EHR	Electronic Health Record
HIPAA	Health Insurance Portability and Accountability Act of 1996
ICD-10	The International Classification of Diseases, 10th Revision, Procedure Coding System
IGNITE PTN	Implementing Genomics in Practice Pragmatic Trials Network
IM	Intermediate metabolizer
LOS	Length of stay
M/M	Medicare/Medicaid
MED	Morphine Equivalent Doses
MME	Morphine Milligram Equivalents
MOP	Manual of Operations
NM	Normal metabolizer
PGx	Pharmacogenetics

PHQ-8	Patient Health Questionnaire-8
PI	Principal investigator
PM	Poor metabolizer
PROMIS	Patient Reported Outcome Measurement System
RM	Rapid metabolizer
ROR	Return of results
SAE	Serious adverse event
SAP	Statistical analysis plan
SIA Score	Silverman Integrated Analgesic Score
SOP	Standard operating procedure
SSN	Social security number
SSRI	Selective serotonin reuptake inhibitors
UM	Ultra-rapid metabolizer

TABLES

Table 1. Genes to be tested and drugs with CPIC recommendations 29
Table 2. CYP2D6 allele to activity score 30
Table 3. CYP2D6 phenotype by Activity Score 30
Table 4. CYP2C19 phenotype by genotype 30
Table 5. CDS summary for acute and chronic pain treatments 31
Table 6. CDS summary for antidepressant medications 32
Table 7. PGx panel genes and drugs with CPIC recommendations 33
Table 8. Data collection schedule 39
Table 9. Sample size calculations 45

FIGURES

Figure 1. ADOPT PGx Trial Design 22

TABLE OF CONTENTS

1. PROTOCOL SYNOPSIS 11
2. INTRODUCTION 18
2.1 Background, Significance, and Rationale 18
2.2 Aims and Objectives of the Study 20
3. ENDPOINTS 21
3.1 Primary Endpoint(s) 21
3.2 Secondary Endpoints 21
4. Study Arms & Design 22
4.1 Randomization 23
4.2 Blinding 23
5. STUDY POPULATION 24
5.1 Inclusion Criteria 24
5.2 Exclusion criteria 24
6. RECRUITMENT AND ENROLLMENT PROCEDURES 26
6.1 Provider Recruitment and Assent 26
6.2 Participant Consent Process 26
6.3 Participant Discontinuation/Withdrawal from the Study 26
6.4 Lost to Follow-Up 27
6.5 Risk 27
6.6 Benefit 27
6.7 Costs to the Participants 28
6.8 Compensation to Participants 28
7. STUDY PROCEDURES 29
7.1 Interventions/Treatments 29
7.2 Baseline Participant Assessments 33
7.3 Follow-up Participant Assessments 34
7.4 Specimen Collection 37
7.5 Specimen Transfer and Genetic Testing Procedures 37
7.6 Return of Results 37
7.7 Clinical Decision Support Systems 37
7.8 Data Collection from the Electronic Health Record 38
7.9 Data Collection from CMS and State Medicaid Agencies 38

8. SAFETY ASSESSMENT AND MONITORING 40
8.1 Medication Side Effects 40
8.2 Events of Interest 40
8.3 Data Safety Monitoring Board 40
8.4 Early Termination and Participant Discontinuation 40
9. MEDICARE AND MEDICAID CLAIMS DATA COLLECTION AND ANALYSIS 41
9.1 Rationale 41
9.2 Data Collection 41
10. STATISTICAL ANALYSIS PLAN AND SAMPLE SIZE 42
10.1 Sample Size Determination 42
10.2 General Statistical Methods 44
10.3 Population for Analyses 44
10.4 Analysis of the Primary Endpoint 44
10.5 Analysis of the Secondary Endpoints 45
10.6 Other Planned Analyses 46
10.7 Interim Analyses 48
10.8 Handling of Missing Data 49
10.9 Multiplicity 49
11. DATA MANAGEMENT 50
11.1 Data Entry and Record Keeping 50
11.2 Data Element Definitions 50
11.3 Database Management and Quality Control of Data 51
11.4 Database Lock and Study Close Out 51
11.5 Data Sharing 52
12. ETHICAL AND HUMAN SUBJECTS CONSIDERATIONS 53
12.1 Institutional Review Board/Ethics Committee Review 53
12.2 Use and Disclosure of Protected Health Information (HIPAA) 53
12.3 Confidentiality and Privacy 53
12.4 Publication and Data Sharing Policies 53
13. PROTOCOL DEVIATIONS AND VIOLATIONS 54
REFERENCES 55
14. APPENDICES 58
14.1 Appendix A. SIA Score Background and Rationale 58
14.2 Appendix B. Table of Minimum Required Variants 60

14.3 Appendix C. CYP2D6 Inhibitors as defined by the FDA guidance on drug interactions 61

1. PROTOCOL SYNOPSIS

**Protocol Title**	A Depression and Opioid Pragmatic Trial in Pharmacogenetics **(ADOPT-PGx)**
**US IND Number**	
**Grant Number**	
**Product/Intervention**	Immediate vs. delayed pharmacogenetic testing and genotype-guided pain or depression therapy
**Objectives**	Acute Pain:<br>● To determine the effect of genotype-guided therapy on pain control and use of DEA schedule II opioids in post-surgical participants<br><br>Chronic Pain:<br>● To determine the effect of genotype-guided therapy on pain control in chronic pain participants<br><br>Depression:<br>● To determine the effect of genotype-guided selection and dosing of antidepressants on control of depression in participants with ≥3 months of depressive symptoms who require new or revised therapy<br><br>All Trials:<br>● To determine the effect of genotype-guided therapy for opioids, antidepressants, and optional additional, CPIC gene-drug pairs on overall well-being<br>● To determine the effect of genotype-guided therapy for opioids, antidepressants, and optional additional, CPIC gene-drug pairs on healthcare utilization
**Study Design**	ADOPT PGx is comprised of three separate trials, Acute Pain, Chronic Pain, and Depression. Each trial is a prospective, multicenter, two arm randomized pragmatic trial. For all trials, participants will be randomized in a 1:1 ratio to immediate pharmacogenetic testing and genotype-guided therapy (Intervention arm) or 6-month delayed testing on participants receiving standard care (Control arm). In each trial, the primary outcome comparison will be between the intervention group (i.e. immediate testing) and control group (i.e. delayed testing) in the subset of participants with actionable phenotypes, within each study.
**Rationale for Study Design**	Pain and depression are conditions that impact substantial proportions of the US population and have challenges associated with identifying the right therapy while minimizing adverse effects or opioid addiction. There is evidence that both opioid and antidepressant prescriptions can be guided by pharmacogenetics (PGx) data based on existing guidelines from the Clinical Pharmacogenetics Implementation Consortium (CPIC). Using such an approach, a recent single site Pragmatic Clinical Trial (PCT) demonstrated that among CYP2D6 poor (PM) and intermediate

	metabolizers (IM), there was greater improvement in pain control in the genotype-guided arm compared to usual care arm[1]. Similarly, existing studies of PGx tailored antidepressant therapy suggest that the genotype-guided approach is superior to usual care in remission and/or response rates, however these studies are small and often industry-sponsored.<br><br>A broader trial is needed to determine the importance of PGx testing and genotype guided therapy for improving symptom management (i.e. pain control or control of depression symptoms), DEA schedule II opioid use, well-being, and overall healthcare utilization.
**Study Population**	**<u>Acute Pain</u>**<br><u>Randomized Population</u><br>Approximately 1730* participants with planned/elective surgery who are anticipated to start a paincontrol medication with existing CPIC guidelines after their surgery<br><br><u>Analytical Population</u><br>Approximately 304 participants from the randomized population who have an actionable phenotype defined as CYP2D6 IM or PM (i.e. CYP2D6 activity score ≤ 0.75)<br><br>**Chronic Pain**<br><u>Randomized Population</u><br>Approximately 985* participants see in primary care or pain specialty clinics who are already prescribed or anticipated to be prescribed tramadol, codeine, or hydrocodone forpain control.<br><br><u>Analytical Population</u><br>Approximately 268 participants from the randomized population who have an actionable phenotype defined as CYP2D6 IM or PM (i.e. CYP2D6 activity score ≤ 0.75)<br><br>**Depression**<br><u>Randomized Population</u><br>Approximately 1540* participants seen in primary care or psychiatry clinics who are already prescribed or anticipate to be prescribed an SSRI with existing CPIC guidelines<br><br><u>Analytical Population</u><br>Approximately 542 participants from the randomized population who have an actionable phenotype, defined as a CYP2D6 PM or ultra-rapid metabolizer (UM), or a CYP2C19 PM, rapid metabolizer (RM), or UM<br><br>*Subject to change based on the actionable phenotype rates and missing data.
**Number of Sites**	11 sites with approximately 60-80 clinics
**Duration of Subject Participation:**	Up to one year from consent to end of follow-up

**Description of implementation of intervention (e.g., dose, schedule, etc.)**	The intervention for ADOPT PGx is the immediate return of PGx testing results to the participant's healthcare provider.<br><br>The PGx testing is comprised of Clinical Laboratory Improvement Amendments (CLIA) validated, panel-based genetic testing of two required genes, *CYP2D6* and *CYP2C19.* The resulting *CYP2D6* genotypes will be converted to enzymatic activity scores and the activity scores converted to metabolizer phenotypes after also taking strong and moderate CYP2D6 enzyme inhibitor drug interactions into account. The *CYP2C19* genotypes will be converted to metabolizer phenotypes.<br><br>The results of the PGx test will be returned to the healthcare providers using standard site-specific laboratory return of results methods, a static report with both interpretation and recommendations, and where possible, interruptive clinical decision support (CDS) alerts within the electronic healthcare system. Both the static report and interruptive alerts will guide health care providers towards medications best suited to the participant.

**Inclusion/Exclusion Criteria**	Inclusion Criteria:<br>**Acute Pain**<br>● Age ≥ 8 years<br>● English speaking or Spanish speaking<br>● Elective/planned surgery types with planned or anticipated to be treated with tramadol, hydrocodone, or codeine pain management at an enrolling site, which may include orthopedic surgeries (e.g. arthroplasty, spine, etc.), open abdominal surgery, or cardiothoracic surgery and others<br><br>**Chronic Pain**<br>● Age ≥ 18 years<br>● English speaking or Spanish speaking<br>● Seen at primary care clinics (such as, but not limited to, Internal Medicine, Family Medicine or Pediatrics) or patients seen in pain-relevant specialty clinics<br>● History of pain for at least the last 3 months<br>● Currently treated or being considered for treatment with tramadol, hydrocodone, or codeine to improve pain management<br><br>**Depression**<br>● Age ≥ 8 years<br>● English speaking or Spanish speaking<br>● Patients followed at psychiatry clinics or primary care clinics at an enrolling site (such as, but not limited to, InternalMedicine, Family Medicine, or Pediatrics)<br>● Documentation of depression and/or provider report of depression<br>● Evidence of depressive symptoms for at least 3 months based on patient interview or documentation in electronic health records<br>● Recent initiation of SSRI therapy, recent revised SSRI therapy, or anticipated need for revised or new SSRI therapy per health care provider

	<u>Exclusion Criteria</u><br>**Trial-wide:**<br>● Life expectancy less than 12 months<br>● Are too cognitively impaired to provide informed consent and/or complete study protocol<br>● Are institutionalized or too ill to participate (i.e. mental or nursing home facility or incarcerated)<br>● Have a history of allogeneic stem cell transplant or liver transplant<br>● People with prior clinical pharmacogenetic test results for genes relevantfor the study in which they will enroll (*CYP2D6* for the pain studies and *CYP2D6* or *CYP2C19* for depression) or already enrolled in an ADOPT PGx trial<br>● Any other medical, behavioral, or developmental condition that in the opinion of the investigator may confound study data/assessments<br><br>**Acute Pain**<br>● Receiving chronic opioid therapy, defined as use of opioids on most days for >3 months<br><br>**Chronic Pain**<br>● Plan to move out of the area within 6 months of enrollment<br>● Undergoing treatment for an active cancer diagnosis<br>● Currently taking daily opioids other than tramadol, codeine or hydrocodone for treatment of pain<br>● Using a pain pump<br><br>**Depression**<br>● Plan to move out of the area within 6 months of enrollment<br>● Have active psychosis or diagnosed psychotic disorders (schizophrenia, schizoaffective disorder, delusional disorder, psychotic depression, substance induced psychosis, schizophreniform disorder)<br>● Have dementia or other neurocognitive disorders due to any cause, such as Alzheimer's disease, vascular/subcortical, lewy body disease, frontotemporal lobar degeneration<br>● Have cognitive developmental delay and/or cognitive disability, including autism spectrum disorders (Note: ADHD is not an exclusion criteria)<br>● Has a seizure disorder<br>● Have bipolar disorder

**Primary Endpoint**	Acute Pain: Silverman Integrated Analgesic Assessment (SIA) score (a composite of pain and opioid usage) at 10 days post-surgery in participants who are genetically or pheno-converted CYP2D6 IM or PM<br><br>Chronic Pain: Pain control, defined as change in the composite pain intensity score from baseline to 3-months in participants who are genetically or pheno-converted CYP2D6 IM or PM<br><br>Depression: Depression symptom control, defined as change in PROMIS depression 8A scores from baseline to 3-months in genetically or pheno-converted CYP2D6 UM/PM or CYP2C19 UM/RM/PM

**Secondary Endpoints**	<u>Acute Pain:</u><br>● Pain intensity at 10 days post-surgery<br>● Opioid usage at 10 days post-surgery<br>● Prescription pain medication misuse score 3 months post-surgery<br>● Mobility score 1-month post-surgery<br>● Opioid persistence 6 months post-surgery<br><br><u>Chronic Pain:</u><br>● Pain reduction magnitude at 3-month follow-up, relative to baseline<br>● Achievement of clinically significant pain reduction (30%) by 3-month follow-up, relative to baseline<br>● Prescription pain medication misuse score at 3-month follow-up<br><br><u>Depression:</u><br>● Change in PHQ-8 scores between baseline and 3 months<br>● Achieve 50% reduction in PHQ-8 scores at 3 months, relative to baseline<br>● Medication side effects severity burden at 3 months<br>● Participant medication adherence at 3 months<br>● Achieve remission at 6 months defined as PROMIS depression score ≤ 16<br>● Achieve remission at 6 months, defined as PHQ-8 score ≤ 4<br><br><u>All Trials:</u><br>● Overall well-being at 6 months in all randomized participants<br>● Concordance between metabolizer phenotype and prescribed medication<br>● Sub-domains of the PROMIS 43 survey: pain interference, physical function, sleep disturbance, social role and activities functioning, fatigue, anxiety, and depression at 6-month follow-up
**Statistical Analyses**	<u>Acute Pain:</u><br>● The effect of genotype guided opioid therapy on pain control will be determined by comparing the 10-day post-surgery SIA scores of the Intervention arm participant with CYP2D6 IM or PM phenotypes to the 10-day post-surgery SIA scores of the Control arm participant with CYP2D6 IM or PM phenotypes using a two-sided t-test or a two-sided Mann Whitney

test, as appropriate, with a two-sided type 1 error rate of 0.049

- Similarly, the effect of genotype guided opioid therapy on all secondary endpoints will be compared between the Intervention arm CYP2D6 IM or PM phenotype and the Control arm CYP2D6 IM or PM phenotype using a two-sided t-test or a two-sided Mann Whitney test
- Additional analyses will include time trends in primary and secondary endpoints, subset analyses, covariate adjustments, and exploratory healthcare utilization and cost effectiveness analyses

<u>Chronic Pain:</u>

- The effect of genotype guided opioid therapy on pain control will be determined by comparing the change in baseline to 3-month follow-up composite pain scores of the Intervention arm participants with CYP2D6 IM or PM phenotypes to the change in baseline to 3-month follow-up composite pain scores of the Control arm participants with CYP2D6 IM or PM phenotypes using a two-sided t-test with type 1 error rate of 0.049
- Similarly, the effect of genotype guided opioid therapy on all chronic pain secondary endpoints will be compared between the Intervention arm CYP2D6 IM or PM subgroup and the Control arm CYP2D6 IM or PM subgroup using either a two-sided t-test or a test of two proportions, as appropriate.
- Additional analyses will include time trends in primary and secondary endpoints, subset analyses, covariate adjustments, and exploratory healthcare utilization and cost effectiveness analyses

<u>Depression</u>:

- The effect of genotype guided antidepressant therapy on depression symptoms will be determined by comparing the 3-month follow-up composite depression scores of the Intervention arm participant subgroup with CYP2D6 UM or PM or CYP2C19 UM, RM, or PM phenotypes to the 3-month follow-up composite depression scores of the Control arm participant subgroup with CYP2D6 UM or PM or CYP2C19 UM, RM, or PM phenotypes using a two-sided t-test with type 1 error rate of 0.049
- Similarly, the effect of genotype guided antidepressant therapy on all depression secondary endpoints will be compared between the Intervention arm CYP2D6 or CYP2C19 UM, RM or PM subgroup and the Control arm CYP2D6 or CYP2C19 UM, RM, or PM subgroup using either a two-sided t-test, a two-sided Mann Whitney test, or a test of two proportions, as appropriate.
- Additional analyses will include time trends in primary and secondary endpoints, subset analyses, covariate adjustments, and exploratory healthcare utilization and cost effectiveness analyses

<u>All Trials:</u>

- Overall well-being of all Intervention participants will be compared to the overall well-being of all Control participants at 6-month follow-up using ANOVA, adjusting for baseline differences in the two groups
- The effect of genotype guided therapy on concordance between phenotype and prescribed medication will be compared between Intervention and Control participants from the metabolizer phenotypic subgroups specified

	for each trial's primary endpoint.<br>● Additional analyses will include time trends, subset analyses, and covariate adjustments analyses

2. INTRODUCTION

2.1 Background, Significance, and Rationale

Study Rationale

Pain and depression are conditions that impact substantial proportions of the US population. Finding safe and effective drug therapies for both conditions is challenging. In the case of treatment for acute and chronic pain, the challenge is finding effective therapy while minimizing adverse effects or opioid addiction (and the ensuing consequences). For depression, there are few clinically relevant predictors of successful treatment leading to multiple trials of inadequate therapy for some patients. Both opioid and antidepressant prescriptions can be guided by pharmacogenetics (PGx) data based on existing guidelines from the Clinical Pharmacogenetics Implementation Consortium (CPIC). A pilot study conducted during IGNITE-1, in patients with chronic pain supports the potential benefit of a genotype-guided approach to pain therapy.[1] Existing studies of tailored antidepressant therapy are small and often industry-sponsored but suggest the genotype-guided approach is superior to usual care. We propose a randomized pragmatic clinical trial that enrolls patients into three PGx-guided therapy scenarios: acute post-surgical pain, chronic pain, and depression. For each scenario, participants will be randomized to genotype-guided drug therapy versus usual approaches to drug therapy selection (hereafter referred to as usual care). Changes in patient reported outcomes representing pain and depression control using standard PROMIS scales define the primary endpoints. Secondary analyses include safety endpoints, changes in overall well-being, and economic impact represented by differences in healthcare utilization and cost effectiveness.

Background and Significance

Acute and chronic pain represents the most prevalent and expensive public health condition in the U.S., affecting an estimated 100M Americans with annual costs to society estimated at $635B dollars.[2, 3] This exceeds the combined costs of cancer, AIDS and heart disease.[3] Opioids have become a mainstay of treatment for chronic pain, yet analgesic responses to opioids are widely variable in both acute and chronic pain.[4, 5] Opioid prescribing rates have more than tripled since 1999, with 65 in 100 people getting an opioid prescription in 2016, and nearly 215M opioid prescriptions dispensed.[6, 7] Nearly half of all opioid prescriptions originate in primary care, and approximately 35% are from surgeons.[8]

Tramadol, codeine, hydrocodone, and oxycodone comprise the vast majority of opioids prescribed in the U.S.[9] Codeine and tramadol are dependent on bioactivation by the CYP2D6 enzyme to morphine and O-desmethyltramadol, respectively, which have 200-fold greater affinity for the μ-opioid receptor than their parent compounds. *CYP2D6* genotype has important relevance for response to codeine and tramadol. Specifically, 5-10% of individuals are poor metabolizers (PMs), with no active CYP2D6 enzyme secondary to frameshift mutations (*3, *6), splicing defects (*4), or complete gene deletion (*5). As a result, PMs are unable to generate the active metabolites of codeine and tramadol and may derive no pain relief from these drugs.[10] Another 2-11% are intermediate metabolizers (IMs), with significantly impaired enzyme activity secondary to having both a nonfunctional and a reduced function *CYP2D6* allele and may derive little pain relief from codeine and tramadol. At the opposite extreme, approximately 1-2% of individuals are ultra-rapid metabolizers (UMs) with *CYP2D6* gene duplication/multiplication. UMs are at increased risk for toxic concentrations of active opioid metabolites, with reports of life-threatening toxicity and death with codeine or tramadol.[11-15] Hydrocodone and oxycodone undergo similar metabolism via CYP2D6 to compounds with 10- to 40-fold higher receptor affinity, respectively, and recent data support *CYP2D6* genotype as an important contributor to hydrocodone efficacy, though risk exists for both drugs in those with UM phenotypes.[1]

Guidelines support *CYP2D6* genotype-guided use of opioid analgesics, but this is rarely done in clinical practice.[10] In a single center PCT[1], *CYP2D6* genotype-guided prescribing led to improved pain control in PMs and IMs compared to a traditional pain management approach. We now propose a multi-center PCT in which we will make recommendations based on *CYP2D6* genotype and CYP2D6 enzyme inhibitor drug interactions that can convert individuals to PM or IM phenotypes. In PM, IM and UM we will recommend avoidance of hydrocodone, tramadol and codeine, and for normal metabolizers (NM), tramadol will be recommended as the preferred opioid, given its opioid and non-opioid mechanisms and purported lower risk for misuse.[16, 17] One study suggested the potential for abuse and dependence with tramadol in patients with chronic non-cancer pain was significantly less than for hydrocodone, and not different from that of non-opioid analgesics.[16]

The prevalence of major depressive disorder ranges from 5 to 10% in primary care, and it can be as high as 37% after critical care hospitalizations and surgeries.[18] SSRI prescriptions have increased 5.8-fold between 1991 and 2011.[19] Response to SSRIs and other antidepressants depends on numerous factors, but pharmacokinetic adjustments based on drug-drug interactions, renal and hepatic function, and pharmacogenomic variants within *CYP2D6* and *CYP2C19* play a substantial role in interindividual drug response.[20]

Existing clinical trials of PGx-guided treatment of depression have been primarily industry-sponsored and often investigate proprietary treatment algorithms.[21] One open label, non-randomized study demonstrated significantly improved depression outcomes in 227 adults with major depressive disorder (MDD) treated with PGx-guided prescribing of psychotropic medications relative to unguided participants.[22] Another 12-week randomized, double-blind trial of 144 adults with MDD receiving PGx-guided prescribing reported a 2.52-fold greater chance of remission of depressive symptoms. A randomized clinical trial of 685 adults with anxiety and depression identified significantly improved outcomes relative to controls in patients diagnosed with depression or anxiety using pharmacogenetic-guided medication selection.[23] A meta-analysis of randomized controlled trials of pharmacogenetic tests and depressive symptom remission concluded that individuals receiving treatment of depression with pharmacogenetic-guided decision support tools were 1.71 (95% CI: 1.17-2.48; p = 0.005) times more likely to achieve symptom remission relative to individuals who receive treatment as usual.[24] Another industry-sponsored, randomized, double-blind prospective trial with only 51 study subjects (26 pharmacogenetic-guided versus 25 unguided) reported a trend toward improved clinical outcomes in a 10-week trial. PGx-guided participants with depression had greater than double the likelihood of response and remission. Mean percent improvement in depressive symptoms was higher for the PGx-guided group over Treatment as Usual (TAU). PGx-guided treatment doubled the likelihood of response in patients with treatment resistant depression.[25] A more recent double-blind randomized controlled trial of 316 adults with MDD failed to report a difference in sustained response within a 12-week period. However, the PGx-guided treatment group had a higher responder rate compared to treatment as usual.[26]

While the above clinical trials suggest improved depression outcomes with use of pharmacogenomic-guided management of psychotropic medications when treating major depression in outpatient psychiatric practices, the majority of the trials were small and sponsored by industry. Importantly, a large, definitive trial with non-proprietary drug selection algorithms has not yet been published. Three of the six most commonly used SSRIs (sertraline, citalopram, and escitalopram) require functional CYP2C19 enzyme activity for their hepatic inactivation in vivo, and CPIC recommends dose reduction in the setting of a CYP2C19 poor metabolizer phenotype (to reduce probability of side effects) and alternative drug in the setting of a CYP2C19 ultra-rapid or rapid phenotype (to reduce the probability of pharmacotherapy failure).[27] Two additional common antidepressants (fluvoxamine, paroxetine) are oxidized by CYP2D6, one of the most polymorphic of all human enzymes. For the present investigation,

PGx-guided antidepressant selection will follow CPIC guidelines for *CYP2D6* and *CYP2C19* phenotypes with regard to the selection or dosing of antidepressants.

2.2 Aims and Objectives of the Study

Our rationale for examining a genotype-guided approach to acute and chronic pain management is based on the importance of CYP2D6 for the bioactivation of tramadol, codeine, and hydrocodone and data from a pilot study supporting improved pain control in IM/PMs in the genotype-guided arm who are taking these drugs at baseline. Similarly, the rationale for examining a genotype-guided approach to depression medication therapy is based on the demonstrated role of CYP2D6 in the bio inactivation and CYP2C19 oxidation of select, commonly used SSRIs. Secondly, data from industry sponsored trials support the hypothesis of improved symptom control in a genotype-guided arm.

Acute Pain: Determine if a genotype-guided approach to acute post-surgical pain therapy leads to improved pain control compared to usual care, as defined by a decrease in the SIA score. Secondarily, we will evaluate whether this approach leads to reduced use of DEA Schedule II opioids and reduced pain intensity.

Chronic Pain: Determine if a genotype-guided approach to pain therapy in participants with at least 3 months of chronic pain leads to improved pain control compared to usual care.

Depression: Determine if genotype-guided dosing or selection of antidepressants among participants with at least 3 months of depressive symptoms who require new or revised antidepressant therapy leads to improved control of depression, compared to usual care.

3. ENDPOINTS

3.1 Primary Endpoint(s)

Acute pain: The primary endpoint for the acute pain study is the SIA score, a composite of pain and opioid usage, at 10 days post-surgical procedure. See Appendix A for the SIA score rationale.

Chronic pain: The primary endpoint for the chronic pain study is change in composite pain intensity score, assessed using the PROMIS pain intensity survey, from baseline to 3 months post return of genetic testing results to the provider.

Depression: The primary endpoint for the depression study is change in depression score, assessed using the PROMIS Emotional Distress - Depression 8b survey (adults) or PROMIS pediatric depressive symptoms (pediatric), from baseline to 3 months post return of genetic testing results to provider.

3.2 Secondary Endpoints

Secondary endpoints include:

All Trials

1. Overall well-being at 6-month follow-up
2. Sub-domains of overall well-being: pain interference, physical function, sleep disturbance, social role and activities functioning, fatigue, anxiety, and depression at 6-month follow-up
3. Concordance between metabolizer phenotype and prescribed medication

Acute pain

1. Pain intensity at 10 days post-surgery
2. Opioid usage at 10 days post-surgery
3. Prescription pain medication misuse score at 3-months post-surgery
4. Mobility at 1-month post-surgery
5. Opioid persistence 6 months post-surgery

Chronic pain

1. Pain reduction magnitude at 3-month post return of genetic testing results to provider, relative to baseline
2. Proportion of participants achieving clinically significant pain reduction (30% reduction from baseline) at 3-months post return of genetic testing results to provider
3. Prescription pain medication misuse at 3-months post return of genetic testing results to provider

Depression

1. Change in PHQ-8 score between baseline and 3 months
2. Achieve 50% reduction in PHQ-8 scores at 3 months, relative to baseline
3. Medication side effects severity burden at 3 months
4. Participant medication adherence at 3 months
5. Achieve remission at 6 months, defined as PROMIS depression score ≤ 16
6. Achieve remission at 6 months, defined as PHQ-8 score ≤ 4

4. Study Arms & Design

This is a prospective, multicenter, subset analysis of 1:1 randomized Intervention (immediate PGx testing and genotype-guided opioid or SSRI therapy with clinical decision support) vs. Control (usual care with delayed PGx testing) pragmatic, open label clinical trial (Figure 1). The three trials are 1) genotype-guided opioid therapy among post-surgical acute pain participants (Acute Pain, Figure 1A), 2) genotype-guided opioid therapy among chronic pain participants (Chronic Pain, Figure 1B), and 3) genotype-guided SSRI therapy in participants with depression (Depression, Figure 1C). Trial-specific outcomes will be compared between participants in the intervention arm and control arms who have an actionable phenotype. Actionable phenotypes are defined as CYP2D6 IM and PMs (i.e. CYP2D6 activity score ≤ 0.75) for the acute pain and chronic pain trials and CYP2C19 UM, RM and PMs or CYP2D6 UM and PMs for the depression trial.

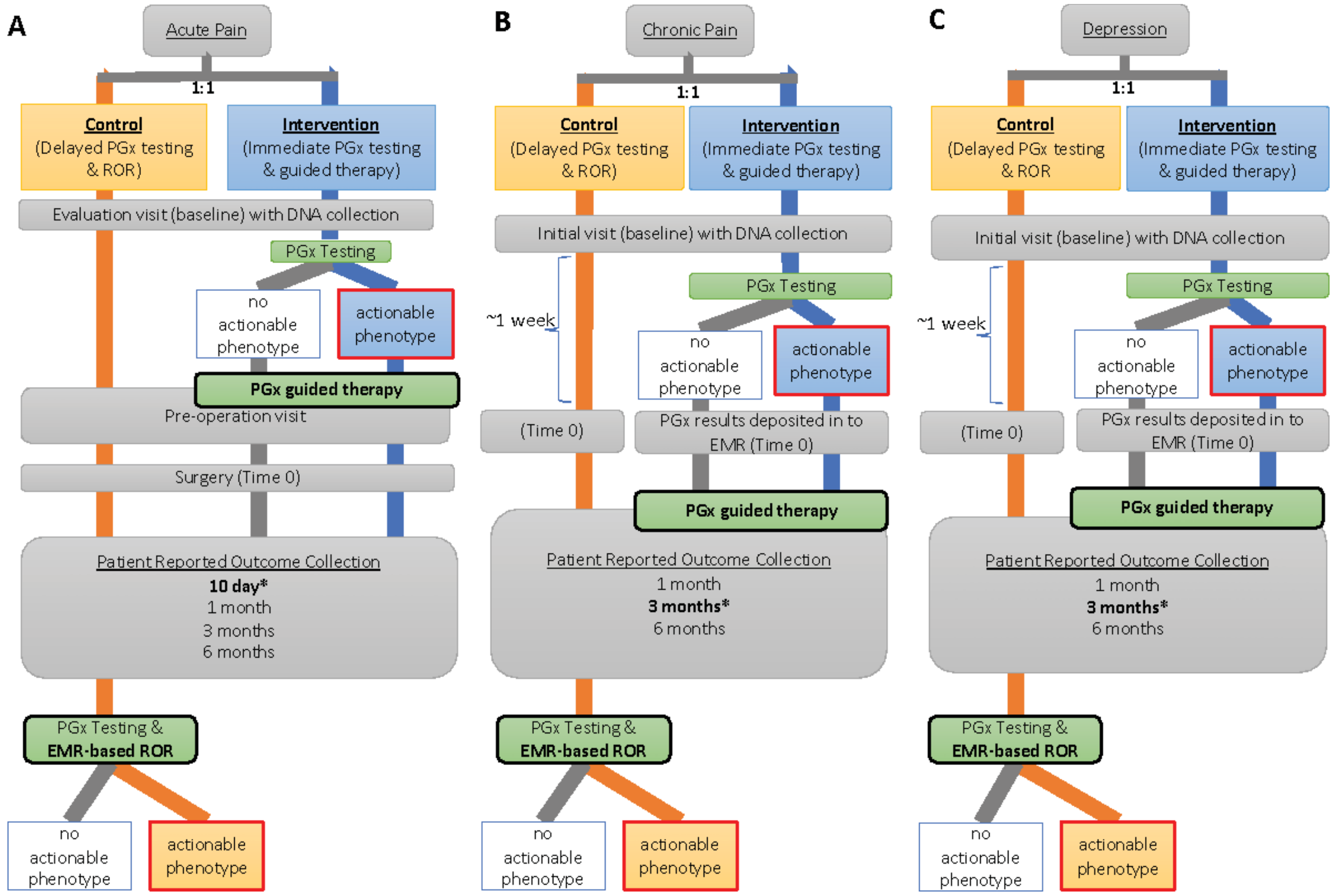

Figure 1. ADOPT PGx Trial Design

Intervention participants are denoted by blue boxes and blue lines, control participants are denoted by orange boxes and orange lines. Return of results are denoted with black outlines, endpoint comparison groups (actionable phenotypes in the control and intervention arms) are denoted with red outlines, and * denote timing of the primary endpoint collection. Time 0 denotes the time from which the follow-up assessment timing begins.

4.1 Randomization

After participants provide informed consent/assent, participants will be randomized in a 1:1 allocation to the Intervention (i.e. immediate PGx testing and genotype-guided opioid or SSRI therapy with clinical decision support) and Control (i.e. usual care with delayed PGx testing) arms. Randomization will be stratified by trial and site or clinic with a random block size within each site or clinic. We anticipate variability in patient populations between recruiting sites or clinics and aim to balance the intervention assignments within the site or clinic unit. Additionally, the randomization for the chronic pain trial will be stratified by presence/absence of depression, and the randomization for the acute pain and depression trials will be stratified by pediatric/adult.

The randomization scheme will be generated by an unblinded statistician. Randomization assignments will be generated in real time in REDCap at the Coordinating Center.

4.2 Blinding

ADOPT PGx randomization assignments will not be blinded to the participants or their providers but will be masked to the UF call center personnel who may be administering some of the participants follow-up surveys. Due to the nature of the intervention, it is impossible to completely mask participants and providers from the pharmacogenetic testing and return of results, hence participants, providers, and local study personnel will not be blinded. While the UF call center personnel administering follow-up surveys will be masked from the results and randomization, study-arm specific questions in the final (6 month) survey may reveal study arm randomization. Additionally, participants may volunteer to reveal their randomization or PGx testing results. If the randomization or phenotype is revealed to the call center, prior to administering the 6 month surveys, it will be documented in the database.

5. STUDY POPULATION

5.1 Inclusion Criteria

Acute Pain

- Age ≥ 8 years
- English speaking or Spanish speaking
- Elective/planned surgery types with planned or anticipated to be treated with tramadol, hydrocodone, or codeine pain management at an enrolling site, which may include orthopedic surgeries(e.g. arthroplasty, spine, etc.), open abdominal surgery, or cardiothoracic surgery and others

Chronic Pain

- Age ≥ 18 years
- English speaking or Spanish speaking
- Seen at primary care clinics at an enrolling site (such as, but not limited to, Internal Medicine, Family Medicine,Pediatrics) or seen in pain-relevant specialty clinics
- History of pain for at least the last 3 months
- Currently treated or being considered for treatment with tramadol, hydrocodone, or codeine to improve pain management

Depression

Age ≥ 8 years
- English speaking or Spanish speaking
- Patients followed at psychiatry clinics or primary care clinics at an enrolling site (such as, but not limited to, Internal Medicine, Family Medicine, orPediatrics)
- Documentation of depression and/or provider report of depression
- Evidence of depressive symptoms for at least 3 months based on patient interview or documentation in electronic health records
- Recent initiation of SSRI therapy, recent revised SSRI therapy, or anticipate need for revised or new SSRI therapy per health care provider

5.2 Exclusion criteria

Trial-wide:

- Life expectancy less than 12 months
- Are too cognitively impaired to provide informed consent/assent and/or complete study protocol
- Are institutionalized or too ill to participate (i.e. mental or nursing home facility or incarcerated)
- Have a history of allogeneic stem cell transplant or liver transplant
- People with prior clinical pharmacogenetic test results for genes relevant for the study in which they will enroll (*CYP2D6* for the pain studies and *CYP2D6* or *CYP2C19* for depression) or already enrolled in an ADOPT PGx trial
- Any other medical, behavioral, or developmental condition that in the opinion of the investigator may confound study data/assessments

Acute Pain

- Receiving chronic opioid therapy, defined as use of opioids on most days for >3 months

Chronic Pain

- Plan to move out of the area within 6 months of enrollment
- Undergoing treatment for an active cancer diagnosis
- Currently taking daily opioids other than tramadol, codeine or hydrocodone for treatment of pain
- Using a pain pump

Depression

- Plan to move out of the area within 6 months of enrollment
- Have active psychosis or diagnosis of a psychotic disorder (schizophrenia, schizoaffectivedisorder, delusional disorder, psychotic depression, substance induced psychosis, schizophreniform disorder) Have dementia or other neurocognitive disorders due to any cause, such as Alzheimer'sdisease, vascular/subcortical, lewy body, frontotemporal lobar degeneration
- Have cognitive developmental delay and/or cognitive disability, including autism spectrum disorders (Note: ADHD is not an exclusion criteria)
- Has a seizure disorder
- Have bipolar disorder

6. RECRUITMENT AND ENROLLMENT PROCEDURES

Outlined below are **suggested strategies** for recruitment and enrollment. It is anticipated that each site will need to optimize the strategies that work best for the clinic and patient population and are in accordance with local regulations and procedures. Strategies utilized will be documented for each recruiting site (see MOP for details). Sites shall maintain local recruitment logs per local policies and share aggregated data with the CC.

6.1 Provider Recruitment and Assent

Acute Pain: Providers with a predominant use of codeine, tramadol, and hydrocodone for pain control before and during the 10-day primary endpoint may be approached by qualified study personnel. Providers will be notified per site institutional guidelines that their patients may be contacted by study site recruiters to participate. Surgical procedures where there is persistent pain at the 10-day primary endpoint should be prioritized for inclusion in the study.

Chronic Pain: Primary care providers, pain clinic providers, and/or anesthesiologists specializing in pain control who treat patients meeting eligibility criteria will be identified, and may be approached by qualified study personnel. Providers will be notified per site institutional guidelines that their patients may be contacted by study site recruiters to participate.

Depression: Primary care providers and psychiatric providers who manage the care of eligible patients will be identified, and may be approached by qualified study personnel, and given the opportunity to participate as a study provider. Providers will be notified per site institutional guidelines that their patients may be contacted by study site recruiters to participate.

6.2 Participant Consent Process

Pre-screened participants that meet the inclusion criteria will be asked to provide an informed consent/assent.

Original informed consent documents will be maintained at the site. Copies of the signed informed consent will be given to the participant. The consenting process can be remote, via a phone or electronically if approved by the reviewing IRB.

6.3 Participant Discontinuation/Withdrawal from the Study

Participants may stop participating and withdraw from the study at any time. All information and data collected up until that point will be used in the study.

If a participant wishes to withdraw consent, they should contact study staff. A participant may also revoke HIPAA authorization and must provide the revocation in writing. Study staff may attempt to obtain a reason for withdrawal from the participant and record it in the study database.

In the acute pain trial, the principal investigators or clinical site investigators may withdraw a participant from the study for any of these reasons:

- Participant does not have the surgery within 12 months of study enrollment
- Participant has the surgery at a healthcare system that is different from where the participant enrolled
- Participant's surgery is scheduled for a date after the close of theenrollment period for that clinical site
- Participant is unable to have the surgery for other medical reasons

6.4 Lost to Follow-Up

Participant status of lost to follow-up will be minimized and retention maximized through various mechanisms to collect study survey responses. The following options will be available: in person, via phone, or a web-based link, text or email. Study staff should confirm the best contact information for the participant at each study encounter.

Additionally, data collected from the EHR and Medicare and Medicaid claims data (see Section 9 for details) will be robust to missing data due to participants that are lost to follow up.

6.5 Risk

The potential risks described below are minimal and reasonable in relation to the potential benefit for genotype-guided therapy to improve the management of pain and depression and reduce the pain and depression burden to society.

Blood Draw
The risks of a blood draw include pain, bruising, and the slight possibility of infection at the place of needle insertion. Some people feel dizzy or may faint during or after a blood draw.

Off FDA label use of antidepressants
In the depression trial, specifically in the pediatric population, the standard practice of medicine may include use of antidepressants that are currently not FDA approved to treat depression in pediatrics. Use of drugs not labeled for use in pediatrics, but prescribed by their treating physician, is not a risk due to study participation.

Prescription Changes
It is possible that the PGx-PGx-recommended drug therapy change may lead to worse pain control or worse control of depression symptoms, though the likelihood is that is no different than with the usual trial and error approach typically used for defining pain management or antidepressant therapy. Pharmacogenetic information is expected to lead to safer and more effective drug therapy, and the ultimate prescribing decision in this study will be left to the physicians. Thus, there are no anticipated risks with basing therapy on pharmacogenetic test results and our preliminary data support a clinical benefit, not risk.

Genetic Information Privacy
The risks of study participation are primarily those related to genetic studies, including risks related to confidentiality surrounding the genetic information and the chance that the genetic information could in some way expose the participant to increased risk regarding employment or that future life, health, disability or long-term care insurance providers could potentially use this genetic information to deny, limit or raise rates for insurance coverage. The Genetic Information Nondiscrimination Act (GINA) makes it illegal for health insurance companies, group health plans, and most employers to discriminate based on genetic information, but other insurers may still use genetic information to discriminate. The pharmacogenetics examples included in our study are only known to be associated with drug response, which is unlikely to lead to insurance discrimination as long as effective, alternative therapies are available, as they are for all gene-drug pairs included in this study.

6.6 Benefit

Study participation may not directly benefit participants of the study especially those who do not have an actionable phenotype. It is possible however that those individuals who have an actionable phenotype may benefit from this study.

Improved drug therapy

The genotype information could lead to improved drug therapy management for select participants. For example, *CYP2D6* genotype may help to identify participants unlikely to respond to codeine, tramadol, or hydrocodone or who are at high risk for toxicity with these opiates plus oxycodone. This could lead to prescribing of alternative opiates or other non-opioid therapies more likely to reduce pain without compromising participant safety. In addition, through improved pain management and use of lower potency opioids in individuals expected to respond well to these drugs based on genotype, the study may indirectly have positive impacts on the opioid crisis by helping to reduce the individual participant opioid burden.

Similar to the above, potential benefits exist for the SSRIs based on more appropriate dosing or selection of an alternative antidepressant. Finally, participants may benefit from optimized therapy for other drugs that may have recommendations based on the panel-based pharmacogenetic testing.

Though the study participants that are randomized to the usual care/control arm have no potential to derive benefit during their participation in the clinical trial, when they complete the 6-month follow-up, they will have their genotype recorded in the EHR, whereby it could be used to guide any future relevant therapies.

6.7 Costs to the Participants

The cost of clinical genotyping and collection of PRO outcomes will be covered by the clinical trial. The cost of their drug therapy will not be covered by the trial since they would be prescribed a medication regardless of participation in the trial. However, taking part in this study may lead to added costs to the participant, specifically the costs of their care, including the physician-prescribed drug therapy, which will generally be covered by the participant's insurance (if insured), and will not be covered by the study.

6.8 Compensation to Participants

Participants will be reimbursed for their time and effort, prorated by study completion. Sites will follow local policies and procedures for amount, timing of, and mechanism for issuing compensation to participants. Participants may be reimbursed for travel or parking expenses per institutional specific policies.

7. STUDY PROCEDURES

An overview of the study procedures to be followed is presented in this section. Recruiting sites will receive training on the protocol and MOP before site activation for enrollment.

7.1 Interventions/Treatments

The intervention, genotype-guided pain or depression therapy, is intended to reflect the practices and procedures that are likely to be implemented if PGx testing were to be integrated into standard clinical practice. The intervention has two technical components: the PGx panel testing and the clinical decisions support/clinical recommendations for providers, described below. The participant and provider facing components of the intervention include the following:

Patient Participants
- The return of the PGx testing results to the participant's medical record that is analogous to typical lab results. Participants may discuss their results with their provider.

Providers
- The return of PGx testing results to the participant's provider via standard site-specific laboratory return of results methods
- Clinical decision support will be provided to all providers, how that is provided may vary. At least one of the two options below that includes drug-drug interactions, is required.
    a. A static report/consult note with interpretation of the genetic testing results and drug-drug interactions, and treatment recommendations
    b. Where possible, a provider prescribing alert for actionable phenotypes, triggered when a relevant opioid or SSRI medication is ordered that indicates the participant's genetic results and/or metabolizer phenotype, the predicted phenotype (i.e. efficacy of various opioid or SSRI medications), other considerations, and treatment recommendations

PGx Panel Testing

For all trials, the PGx testing panel includes two required genes: *CYP2C19* and *CYP2D6*. The list of drugs with responses affected by these genes that will be the focus of recommendations in the trial are found in **Table 1**. Reflecting the pragmatic nature of the trial, there may be site to site variability in the testing of specific variants due to site-specific institutional approvals. The minimal required set of *CYP2C19* and *CYP2D6* variants are listed in Appendix B.

Table 1. Genes to be tested and drugs with CPIC recommendations

Genes	Drugs
CYP2C19	citalopram, escitalopram, sertraline
CYP2D6	codeine, oxycodone, tramadol, hydrocodone, fluvoxamine, paroxetine

CYP2D6 phenotypes will be inferred based on the activity scoring system as shown in **Table 2 and Table 3**. The activity score value of each allele (**Table 2**) is added together to determine the total activity score for the diplotype. The activity scores are converted to phenotypes per **Table 3**. The IM phenotype will be defined as an activity score of >0 to 0.75, not >0.75 to 1.0. As there is debate about

how to define the IM phenotype, we will have an *a priori* analysis plan to determine whether participants in the usual care arm with an activity score of >0.75 to 1 (who we will define as NM) have worse pain control or depressive symptoms than NMs with an activity score > 1 to 2. If an allele is duplicated, and it is unknown which allele is duplicated, then the AS may be a ranged number, resulting in a ranged phenotype. If the ranged phenotype is NM-UM, the individual will be treated clinically as is if they were a UM.

Table 2. CYP2D6 allele to activity score

Alleles	Activity value+
*1, *2	1
*9, *14, *17, *29, *41	0.5
*10	0.25
*3, *4, *5, *6, *7, *8	0

+ Lab reported results may use older versions of CPIC guidelines for activity value definitions

Table 3. CYP2D6 phenotype by Activity Score

Inferred CYP2D6 phenotype	*CYP2D6* activity score (AS)
UM	> 2.0
NM	>0.75 to 2.0
IM*	>0 to 0.75
PM	0

*This phenotype definition differences from current CPIC guidelines and may be different from what appears in the lab reported phenotypes

CYP2C19 phenotypes will be inferred from the genotypes, as shown in **Table 4**.

Table 4. CYP2C19 phenotype by genotype

Inferred CYP2C19 phenotype	Example *CYP2C19* genotypes
UM	*17/*17
RM	*1/*17
NM	*1/*1
IM	*1/*2,*1/*3, *2/*17, *3/*17
PM	*2/*2, *2/*3, *3/*3

The CYP2C19 metabolizer phenotype and the CYP2D6 metabolizer phenotypes and/or activity scores will be included in the PGx testing report generated by the laboratory, which is then returned to the provider and deposited in the EMR, where available.

Clinical Decisions Support / Clinical Recommendations

Acute and Chronic Pain
Standardized clinical consult notes with or without pharmacogenetics expert consultations will be generated based on the genotype-inferred phenotype, and in the case of drugs including *CYP2D6* guidance, will include consideration of drug interactions.

For *CYP2D6*, those taking concomitantly a strong CYP2D6 inhibitor (as defined by FDA guidance on drug interactions[28]) will be considered to have been pheno-converted to a PM and recommendations will be consistent with that for a PM. Examples of strong inhibitors include, but are not limited to: bupropion, fluoxetine and paroxetine. Moderate inhibitors include, but are not limited to: duloxetine and mirabegron and reduce *CYP2D6* activity scores by 50%, and thus the inferred phenotype will be based on the genotype activity score x 0.5. Activity scores that align with a given phenotype, and which will ultimately will be based on genotype and drug interaction data, are shown in Tables 2 and 3. A full list of strong and moderate inhibitors can be found in Appendix C.

While we will not be including oxycodone as a drug on which we will make recommendations in IMs and PMs, based on UF data and other data in the literature that make the importance of *CYP2D6* for the pain response unclear, there are data that suggest that CYP2D6 UMs can have significant toxicities (especially respiratory depression).[1, 29-31] Thus, oxycodone will be a drug for which we will make strong recommendations about avoiding use in UMs.
Using a standardized consult note/CDS or pharmacogenetics expert consultation, recommendations will be made to avoid tramadol, hydrocodone, or codeine in PMs, IMs, and UMs and to use an alternative opioid (e.g. morphine, hydromorphone) or non-opioid (e.g. NSAID), as noted in Table 5. Consideration of tramadol as the first line opioid will be recommended for NMs. While not part of the primary hypothesis, for safety reasons, avoidance of oxycodone will also be recommended in UMs.

Additionally, where possible, the CDS will include a provider alert for actionable phenotypes, triggered when a relevant opioid medication is ordered that indicates the participant's genetic results, the predicted phenotype, other considerations, and treatment recommendations.

Table 5. CDS summary for acute and chronic pain treatments

Treatment	CYP2D6 Phenotypes				
	Ultra-rapid	Normal to Ultra-Rapid	Normal*	Intermediate**	Poor
Tramadol	Avoid	Avoid	Preferred opioid	Avoid	Avoid
Codeine	Avoid	Avoid	Acceptable	Avoid	Avoid
Hydrocodone	Avoid	Avoid	Acceptable	Avoid	Avoid
Oxycodone	Avoid	Avoid	Acceptable	Acceptable	Acceptable

*Defined as activity score >0.75 and ≤ 2.0
**Defined as activity score >0 and ≤ 0.75

Depression
Clinical decision support in the form of computerized alerts, standardized clinical consult notes, and/or pharmacist consultations will be generated to guide prescribers on dosing or selection of SSRIs or selection of alternate antidepressants based on predicted phenotype. Recommendations on drug choice and starting dose will be made in accordance with CPIC guidelines and/or FDA label information for paroxetine, fluvoxamine, citalopram, escitalopram, and sertraline, as outlined in Table 6. Drug-drug interactions leading to pheno-conversion will be incorporated into recommendations. For CYP2D6, concomitant use of a strong inhibitor (listed above) will result in a predicted phenotype of poor metabolizer, and the effect of concomitant moderate inhibitor (listed above) will be estimated by multiplying the CYP2D6 activity score by 0.5.

Table 6. CDS summary for antidepressant medications

	***CYP2D6* Phenotypes**				
	**Ultra-rapid**	**Normal to Ultra-rapid**	**Normal**	**Intermediate**	**Poor**
Paroxetine	Avoid	Avoid	Acceptable	Acceptable	Avoid or 50% Dose Reduction*
Fluvoxamine	No recommendation	No recommendation	Acceptable	Acceptable	Avoid or 25-50% Dose Reduction*
	***CYP2C19* Phenotypes**				
	**Ultra-rapid and Rapid**		**Normal**	**Intermediate**	**Poor**
Citalopram	Avoid		Acceptable	Acceptable	Avoid or 50% Reduction**
Escitalopram	Avoid		Acceptable	Acceptable	Avoid or 50% Reduction*
Sertraline	Monitor for Non-response		Acceptable	Acceptable	Avoid or 50% Reduction*

* Dose reductions refer to starting dose of medication; Avoid refers to recommendation to switch to a drug not predominantly metabolized by the listed drug metabolizing enzyme. No recommendation refers to a scenario where CDS is not triggered but the drug is also not offered as an alternative given the phenotype.
** Per the FDA warning, citalopram 20 mg/day is the maximum recommended dose in *CYP2C19* poor metabolizers due to the risk of QT prolongation

Context of the intervention implementations

Reflecting the pragmatic nature of the clinical trial, the intervention may be implemented in environments in which there are existing PGx testing and CDS. The PGx testing may be completed as part of larger, CLIA validated, PGx panel that include optional gene-drug pairs (Table 7). Additionally, clinical decision support may be in place for the study gene-drug pairs as well as other CPIC drug-gene pairs and/or other drugs affected by the study genes.

Table 7. PGx panel genes and drugs with CPIC recommendations

Genes	Drugs
CYP2C19 ***(Required)***	amitriptyline, clopidogrel, clomipramine, doxepin, imipramine, trimipramine, voriconazole, omeprazole, esomeprazole, pantoprazole, lansoprazole, dexlansoprazole
CYP2D6 ***(Required)***	amitriptyline, clomipramine, desipramine, doxepin, imipramine, nortriptyline, ondansetron, trimipramine, tropisetron
CYP2C9	warfarin, phenytoin
CYP3A5	Tacrolimus
SLCO1B1	Simvastatin
TPMT	azathioprine, mercaptopurine, thioguanine
NUDT15	azathioprine, mercaptopurine, thioguanine
VKORC1	Warfarin
CYP4F2	Warfarin

7.2 Baseline Participant Assessments

All study participants will complete baseline surveys for demographics and participant reported outcomes (PROs) (see Table 8 for details). These data will be collected by the study coordinator in person or alternatively by using telephone, email, or text.

Baseline data will be collected for the Chronic Pain and Depression trials following consent, for the Acute pain trial, survey data will be collected before surgery. The type of surgery information will be collected after the completed procedure.

Participant demographics – including but not limited to age, sex, gender, race, Hispanic/Latino ethnicity (Yes or no), and smoking status will be administered at baseline as a survey.

Past medical history – A snapshot of the participant's medical history. See MOP for details.

Baseline medications - Participant medications will be collected through the EHR and/or participant self-report. See MOP for details.

7.3 Follow-up Participant Assessments

The event that starts the post-intervention data collection timing are as follows.

Acute Pain:

- Date of surgery (intervention and control arms)

Chronic Pain and Depression:

- Intervention arm: When the pharmacogenetics results are returned to the provider and deposited in the EMR
- Control arm: At date of DNA sample collection plus one week, where one week reflects the average time from sample collection to returning the results to the EHR
- Note: There will be variability in time the results are returned to provider and when genotype-guided therapy is delivered to the participant, time from results being available to the next provider-participant interaction (e.g. email, clinic visit, new prescription order, phone visit, etc.) will be recorded by study personnel.

Post intervention assessment collection may be done by the University of Florida College of PharmacyCall Center. The center agents will be trained survey collectors*, using an IRB approved telephone script. The collected survey responses will be entered into the study database. If preferred, the participant will have the option to complete the study follow-up survey by a URL link sent by text (to mobile phone) or email and captured in REDCap database

In the event that the call center is unable to reach a participant, local study coordinators may contact participants to facilitate completion of the follow-up surveys.

Data will be collected in the following timeframes*:

- Acute pain: 10 days, 1 month, 3 months, and 6 months post-surgery
- Chronic pain and depression: 1-month, 3 months, and 6 months post return of results for the intervention arm, and post baseline assessments for the control arm.

**See MOP for call center agents and survey collection interval windows*

All Trials

PROMIS 43 – Adults (≥ 18 years of age) will complete a 43-question survey assessing well-being and sub domains: pain interference, physical function, sleep disturbance, social role and activities functioning, fatigue, anxiety, and depression.[32] This survey will be administered to adult participants at baseline, 1-month, 3-months, and 6-months post-surgery (acute pain), or baseline, 1-month, 3-months, and 6-months post return of results (chronic pain, depression).

PROMIS pediatrics 37 – Pediatric study participants (ages 8-17) will complete a 37-question survey assessing pediatric well-being and sub domains: pain interference, physical function, sleep disturbance, social role and activities functioning, fatigue, anxiety, and depression. This survey will be administered to pediatric participants at baseline, 1-month, 3-months, and 6-months post-surgery (acute pain), or baseline, 1 month, 3 months, and 6 months post return of results (depression).

Health care utilization – Survey of the number of significant cost driver health care encounters (e.g. hospitalizations, clinic visits, etc.). This survey will be administered to participants at 10-day, 1-month, 3-months, and 6-months post-surgery (acute pain), or baseline, 1-month, 3-months, and 6-months post return of results (chronic pain, depression).

Medications - Participant medications will be collected using patient surveys at baseline and the primary endpoint time points: 10-days for Acute Pain and 3-months for Depression and Chronic pain.

Productivity loss – Survey of loss of work the time and pay lost due to the participant's depression or pain. This survey will be administered to participants at 1-month, 3-months, and 6-months post-surgery (acute pain), or 1-month, 3-months, and 6-months post return of results (chronic pain, depression).

Acute Pain:
PROMIS Numeric Rating Scale - Pain Intensity - An 11-point numeric scale ranging from 0 (no pain) to 10 (worst possible pain) for average pain. This survey will be administered to all participants at baseline, 10 days, 1-month, 3-months, and 6-months post-surgery.

PROMIS pain intensity scale – A participant completed 3-question survey of average pain over the last 7 days, worst pain over the last 7 days, and current pain. Each question is on a 1-5 integer scale, the higher the value, the more intense the pain. This survey will be administered to all participants at baseline, 10 days, 1-month, 3-months, and 6-months post-surgery.

Opioid consumption questionnaire – A participant completed survey of pain prescription medication consumption, including the type of opioid pain medication, numberof pills dispensed, if a refill has been obtained, and the number of pills left for each opioid pain medication selected. This survey will be administered to all participants at 10 days and 1-month post- surgery.

PROMIS prescription medication mis-use scale - A participant completed survey of prescription medication misuse over the past 3 months. The survey is comprised of 7 questions on a 5-point ordinal scale of never/rarely/sometimes/frequently/almost always or not at all/a little bit/somewhat/quite a bit/very much[33]. This survey will be administered at baseline, 3-months, and 6-months post-surgery.

PROMIS Item Bank v2.0 Mobility – A participant completed survey of the participant's level of difficulty in completing different physical activities such as standing unassisted, walking, and sprinting or activities that their health currently limits. The survey is comprised of 15 questions, each answered on a 5-point scale ranging from without difficulty to unable to do, or not at all through cannot do[34]. This survey will be administered at baseline, 10 days, 1-month, 3-months, and 6-months post-surgery to the adult participants.

PROMIS Pediatric Mobility – Short Form 8a – A participant completed 8 item survey of difficulty in completing different physical activities such as getting up from the floor, standing on tiptoes, and ability to do sports and exercises that their peers can complete [35]. The survey will be administered at baseline, 10 days, 1-month, 3-months, and 6-months post-surgery to the pediatric participants.

Acute pain specific health care utilization – A participant completed survey of hospital length of stay for the surgical admission, administered at 10 days post-surgery.

Opioid side effects – A participant completed survey of common opioid side effects experienced and the extent to which those side effects bothered the participant. This survey will be administered at baseline, 10 days, 1-month, 3-months, and 6-months post-surgery.

Opioid persistence - Whether or not the participant had an opioid prescription refill 90 – 180 days post-surgery. This survey will be administered to participants at 6 months post-surgery.

Chronic Pain:
PROMIS pain intensity scale – A participant completed 3-question survey of average pain over the last 7 days, worst pain over the last 7 days, and current pain. Each question is on a 1-5 integer scale, the

higher the value, the more intense the pain. This survey will be administered at baseline, 1 month, 3 months, and 6 months post return of results.

PROMIS prescription pain medication mis-use scale - A participant completed survey of prescription medication misuse over the past 30 days. The survey is comprised of 7 questions on a 5-point ordinal scale of never/rarely/sometimes/frequently/almost always or not at all/a little bit/somewhat/quite a bit/very much[33]. This survey will be administered at baseline, 3 months, and 6 months post return of results.

PROMIS emotional distress depression 8b survey - A participant completed eight question survey assessing frequency of depression symptoms over the past 7 days[36]. This survey will be administered at baseline, 1 month, 3 months, and 6 months post return of results to the adult participants.

Opioid side effects – A participant completed survey of common opioid side effects experienced and the extent to which those side effects bothered the participant. This survey will be administered at baseline, 3 months, and 6 months post return of results to the adult participants.

Depression:
PROMIS emotional distress depression 8b survey - A participant completed eight question survey assessing frequency of depression symptoms over the past 7 days[36]. This survey will be administered at baseline,1 month, 3 months, and 6 months post return of results to the adult participants.

PROMIS Pediatric Depressive Symptoms Short Form 8a- A pediatric (ages 8-17 years) participant completed eight question survey assessing frequency of depression symptoms over the past 7 days[37]. This survey will be administered at baseline, 3 months, and 6 months post return of results to the pediatric study participants .

Patient Health Questionnaire-8 – An 8 item survey completed by the participant assessing depression symptoms over the last two weeks. This survey will be administered at baseline, 3 months, and 6 months post return of results[38].

Antidepressant side effects – A participant completed survey of relevant antidepressant side effects. This survey will be administered at baseline, 1 month, 3 months, and 6 months post return of results.

Voils Medication Adherence - A participant completed three question survey, 5-point scale survey assessing participant medication adherence[39]. This survey will be administered to depression trial participants at baseline, 1 month, 3-months and 6 months post return of results.

All of the PROMIS® surveys can be found online[40].

7.4 Specimen Collection

DNA sample collection can be whole blood by venipuncture, saliva, buccal swab, or mouthwash, whichever is most appropriate for the site or study participant preference. The sample collection can occur in person or by mailed kits **after consenting**.

Once sample collection is completed, participants are randomized to a trial Control or Intervention arm. All DNA sample types and collection processes are detailed in the MOP.

7.5 Specimen Transfer and Genetic Testing Procedures

All collected specimens will be clearly marked with two patient identifiers (CLIA requirement). Samples from the Intervention arm participants will be processed, using analytically validated PGx testing procedures (see Table 1 for gene and variant list). For Control arm samples, designated laboratories will either extract DNA after sample receipt, and store the DNA for later analysis or store the sample and extract DNA after the Control participant has completed the 6-month follow-up assessment. After the Control participant has completed the 6-month follow-up assessment, Control Arm samples will be processed using analytically validated procedures. Details of sample storage and transport will be presented in the MOP.

7.6 Return of Results

The designated laboratory will transfer participant genetic testing results to the EHR. Genetic results will be imported into the study database. Providers will be notified of participant results via standard site-specific laboratory return of results methods, a static report/consult note with interpretation and treatment recommendations, and, where possible, a CDS within the EHR as described in Sections 7.1 and 7.7.

After their participation in the trial is complete, the PGx testing results will be activelyreturned to the participants. The PGx testing results that are returned to the participant will follow current FDA guidance.

7.7 Clinical Decision Support Systems

Providers will be notified of participant results via standard site-specific laboratory return of results methods, a static report/consult note with both interpretation and treatment recommendations, and, where possible, a just in time alert within the electronic health system. Sites will work with their institutional information technology departments to set up provider alerts for actionable PGx phenotypes when a relevant opioid or SSRI medication is ordered. Provider alerts will indicate the participant's genetic results, the predicted phenotype (e.g. efficacy of various opioid or SSRIs), and prescribing recommendations.

Continuing on the health IT collaborative efforts established in IGNITE I through CDSKB (http://cdskb.org), all participating sites will seek to harmonize their CDS logic, recommendations, and provider alerts using a framework of required and optional elements for both the static report and the just in time alert. However, due to the pragmatic nature of the trial, the many involved research sites, and variations in local CDS policies, it is anticipated that there will be differences in CDS implementation details, but all sites will ensure that providers are alerted to the PGx test results and associated significance. Details of the alerts will be provided in the MOP.

7.8 Data Collection from the Electronic Health Record

Data collected from the local EHR will include prescription information and encounters including clinic visits, hospitalizations, and emergency department visits and associated diagnoses for the study interval time-zero through six-month follow up. Sites will extract data using prespecified programmatic algorithms, site-developed algorithms, and/or manual chart abstractions. EHR query results will be sent to the IGNITE PTN CC via secure data transfer and formatted for consistency across sites. Details for the EHR elements and transfer process will be provided in the MOP.

7.9 Data Collection from CMS and State Medicaid Agencies

Medicare and Medicaid claims data from CMS and state Medicaid agencies will be used to assess health care utilization, costs, and medications among participants covered by either Medicare or Medicaid. Claims made in the period of 12 months prior to surgery (acute pain) or return of results (chronic pain, depression) through 6 months following those events will be collected. See Section 9 and the MOP for additional details. Schedule of Activities and Timeframe for Collection of Endpoints

Table 8. Data collection schedule

Data collection schedule, method of data collection, and timing

Outcome	Data source/instrument		Baseline	10 d ± 3 d	1 mo. ± 7 d	3 mo. ± 14 d	6 mo. ± 14 d
**Pain Assessments**							
Pain intensity	PROMIS Pain intensity scales		AP, CP	AP	AP, CP	AP, CP*	AP, CP
Pain intensity	PROMIS Numeric Rating Scale - Pain Intensity (NPRS)		AP	AP*	AP	AP	AP
Daily opioid dose & type of opioid use	Average daily mg morphine equivalents (MED) since discharge use of opioids: tramadol/ codeine vs others			AP*	AP		
Opioid use disorder	PROMIS - Prescription pain medication misuse subscale		AP, CP			AP, CP	AP, CP
Mobility	PROMIS mobility		AP	AP	AP	AP	AP
**Depression Assessments**							
Depressive state	PROMIS Emotional Distress - Depression 8b survey		CP, D		CP, D	CP, D*	CP, D
Depressive state	Patient Health Questionnaire-8 depression scale		D			D	D
**Participant Medications**							
Assessment of prescriptions	Custom survey/EHR		AP, CP, D	AP		CP, D	
Assessment of filled prescriptions	Medicaid and Medicare billing records for 12 months prior to time 0 through 6 months after time 0		AP, CP, D				

Adherence	Voils Medication Adherence		D		D	D	D
SSRI AE Survey	Custom survey		D		D	D	D
Opioid side effect survey	Adapted medication side effect survey (SPACE)		AP, CP	AP	AP, CP	AP, CP	AP, CP
Opioid persistence	Custom survey						AP
**Participant Actions**							
Interaction with provider	Participant visits			CP, D			
Interaction with test result	Custom survey						AP, CP, D
**Quality of life and well being**							
Past Medical History	Participant visits/custom survey		AP, CP, D				
Well-being	PROMIS43/PROMIS Peds37		AP, CP, D		AP, CP, D	AP, CP, D	AP, CP, D
**Healthcare Utilization and Costs**							
Medicare/Medicaid billing records	Medicaid and Medicare billing records for 12 months prior to time 0 through 6 months after time 0		AP, CP, D				
LoS	LoS for index admission			AP			
ED/urgent care & inpatient visits	Participant reported visits				AP, CP, D	AP, CP, D	AP, CP, D
Outpatient visits	Participant reported primary care & pain clinic visits				AP, CP, D	AP, CP, D	AP, CP, D
Outpatient visits	Long-term care or in-patient rehab days, home healthcare days				AP	AP	AP
Productively Loss	Survey of lost work time and income				AP, CP, D	AP, CP, D	AP, CP, D

Abbreviations: CP – chronic pain, AP – acute post-surgical pain, D-depression, ED – emergency department, EHR – electronic health record; LoS – length of stay; PT – physical therapy
*Denote primary endpoints

8. SAFETY ASSESSMENT AND MONITORING

ADOPT PGx is a prospective, multicenter, subset analysis of 1:1 randomized Intervention (immediate PGx testing and genotype-guided opioid or SSRI therapy with clinical decision support) vs. Control (usual care with delayed PGx testing) pragmatic, open label clinical trial. It is regulated under an Abbreviated Investigational Device Exemption (IDE). The genotyping-guided therapy is the device and classified as a minimal risk to the welfare of the enrolled participants. Only Adverse Device Effect (ADE) events suspected to be related to the specimen collection, laboratory assay genotyping results, and phenoconversion recommendations from the Best Practice Alerts (BPAs)/Consult notes will be reported to the IRB. Reportable ADEs or unanticipated Adverse Device Effect (UADEs) events including unanticipated study related deaths will be collected in the study database per IRB reporting policies. See the MOP for more details.

The IRB reporting timeline requirements are:

- Immediately (within 24 hours) upon learning of an unanticipated study-related death. Study personnel will notify the IRB via phone or email by providing a brief summary of the event; then within 1 week (5 business days), study personnel should submit report to IRB
- Within 5 business days for unanticipated events
- Within 10 business days for any other problem or event

8.1 Medication Side Effects

Participant reported medication side effects will be collected in the baseline and follow-up surveys. The opioid medication side effects that will be surveyed include: problems with sleep; nausea, gas or indigestion; constipation or diarrhea; and dizziness or balance problems. The SSRI medication side effects that will be surveyed include: fatigue, change in weight, GI upset, sedation/somnolence, anxiety, insomnia, irritability/hostility, and sexual dysfunction.

8.2 Events of Interest

Participant reported emergency department visits and hospitalizations will be collected in the 1, 3, and 6-month follow-up participant surveys. EHR may be used as an additional source for emergency department visits and hospitalizations, up to approximately 12 months after the last patient is randomized, i.e. through completion of the 6 month-follow up activities. For the details of collecting EHR data, see Protocol section 7.8 and MOP. EHR results will be sent to the CC via secure data transfer and formatted for data analyses.

8.3 Data Safety Monitoring Board

The DSMB, appointed by the NHGRI, will be responsible for providing recommendations regarding the conduct of the study and guidance to ensure the safety and well-being of participants. The DSMB will meet semi-annually. A DSMB Charter will be developed detailing the procedures to be followed. A Data and Safety Monitoring Plan and a separate DSMB statistical analysis plan (SAP) will be developed in collaboration with the IGNITE PTN, IGNITE PTN CC, and NHGRI, and enacted by the DSMB.

8.4 Early Termination and Participant Discontinuation

Early termination considerations will generally apply only to emerging issues of major concern, or problems with trial conduct that suggest the trial could not be completed successfully with a reliable conclusion in a feasible time frame.

The site investigator, sponsor or institution may stop involvement of any participant in this research

study at any time without their consent. This may be because the research study is being stopped, the instructions of the study team have not been followed, the investigator believes it is in the participant's best interest, or for any other reason. If specimens or data have been stored as part of the research study, they too may be destroyed without participant consent.

9. MEDICARE AND MEDICAID CLAIMS DATA COLLECTION AND ANALYSIS

9.1 Rationale

The economic analyses will be conducted from the perspective of the payer with the primary goal to provide cost-effectiveness data that can inform reimbursement decisions for genotyping. Reporting metrics will include:

1. Average costs for each study arm, considering overall cost to the payer and select services directly relevant to the acute pain, chronic pain, and depression groups
2. Differences in cost utility between the intervention arm, considering overall cost to the payer and select services directly relevant to the acute pain, chronic pain, and depression groups
3. Incremental cost-effectiveness ratio (ICER), i.e., the incremental change in cost per unit improvement of effectiveness.

All metrics will be ascertained over a 6-month follow-up period with the assumption that beneficial effects of genotype-guided therapy are fully realized within this time period and that the control arm participants have not yet developed any cross-over effects from the 6-month delayed testing.

In a population of mixed payer types, collecting actual costs for all participants is not feasible. Therefore, costs will be obtained from Medicare and Medicaid claims data and imputed or cross-walked to the other payer types. Based on data in the National Inpatient Sample, 53% and 4% of knee and 59% and 4% of hip arthroplasties were reimbursed by Medicare and Medicaid, respectively. For chronic pain participants, data from the University of Florida suggest 40% and 20% are covered by Medicare and Medicaid, respectively. Thus, considering enrollment gaps, at least 60% of the study population is anticipated to have claims data, from which comprehensive cost analyses can be performed and extrapolated to the entire cohort. Major cost drivers associated with pain and depression will be ascertained from the participant using validated resource questionnaires, and verified through secondary claims data whenever possible.

9.2 Data Collection

Data for this specialized analysis will come from these sources:

1. Participant report of healthcare utilization (e.g. emergency department, urgent care, office) for all participants, these will focus on high-cost items and/or items that are expected to be sensitive to the intervention
2. Medicare claims data for Medicare enrollees
3. Medicaid claims data for Medicaid enrollees

Participant reported visits: Participant reported assessment for health care utilization is previously described in section 7.3.
Medicare claims data: For Medicare enrollees in fee-for-service (FFS) plans or Medicare Advantage, clinical groups will ascertain Medicare claims data (Part A-D) directly from the Centers for Medicare and Medicaid Services (CMS).
Medicaid claims data: For Medicaid enrollees, clinical groups will ascertain claims data from their states' Medicaid agencies directly.

To ensure full adjudication, requests to Medicare/Medicaid for claims data will be timed to occur between 6-12 months after the end of the 6-months follow-up of the last enrolled patient. Claims data will also be requested for one year before trial enrollment up to 6 months thereafter.

The procedure for obtaining claims data will require a database linkage step – mapping the participant study ID to the Medicare/Medicaid identifiers. The data linkage will rely on social security number (SSN) or Medicare ID and date of birth (DoB), which can be collected in two possible ways:

1. Both SSN and/or Medicare ID and DoB are collected as part of the trial by the recruiting site
2. One or both variables are extracted from the medical record of the participating health center by a healthcare data security officer or the local equivalent, on behalf of the study team

For **2020-2024 Medicare data**, we will have three groups:(1) Clinical Trial Participants: To achieve our goals to understand the effects of a genotype-guided approach, we will have all Medicare beneficiaries who participated in the ADOPT PGx clinical trial and enrolled in the trial. (2) Controls- a standardized national cohort based on the 5% sample of beneficiaries who did not participate the clinical trial but had similar characteristics with beneficiaries who participate the clinical trial (received opioids or antidepressants). We will further require beneficiaries to have at least 2 months of Part D and at least 2 months of FFS in the year that they received the opioid or antidepressant. (3) Controls – a cohort of beneficiaries from Florida, New York, North Carolina, Tennessee, South Dakota, and Indiana (the states in which the clinical sites are located), pulled from the 100% sample, in which the beneficiaries did not participate the clinical trial but have similar characteristics with beneficiaries who participate the clinical trial received opioids or antidepressants). We will further require beneficiaries to have at least 2 months of Part D and at least 2 months of FFS in the year that they received the opioid or antidepressant. The clinical trial participants will be used to evaluate the effects of immediate vs delayed pharmacogenetic testing and genotype-guided pain or depression therapy on health care utilization, healthcare costs, and cost-effectiveness. We will further compare the Medicare beneficiaries among the trial enrollees to large random sample of beneficiaries who take pain medications to compare demographics and clinical characteristics to explore representativeness of the trial sample, making inferences about generalizability of the results.

For **2020-2024 Medicaid data**, we will have two groups: (1) Clinical Trial Participants: we will have all Medicaid beneficiaries who participated in the ADOPT PGx clinical trial and enrolled in the trial. (2) Controls- a cohort of beneficiaries from Florida, New York, North Carolina, Tennessee, South Dakota, and Indiana (the states in which the clinical sites are located), in which the beneficiaries did not participate the clinical trial but have similar characteristics with beneficiaries who participate the clinical trial received opioids or antidepressants). We will further require beneficiaries to have at least 2 months of FFS in the year that they received the opioid or antidepressant.

For the Controls for Medicare and Medicaid beneficiaries, waiver of consent forms and HIPPA waiver of authorization will be obtained as the analyses of health care utilization and costs are retrospective in nature. It will be impracticable to obtain authorization from this population sample as patient contact information is unavailable.

<u>Costs imputation</u>
The Medicare/Medicaid (M/M) claims will be used to impute healthcare utilization and costs for participants who are enrolled in other plans or not insured as follows. First, the subset of the study participants with both the participant reported set of medical encounters and M/M claims data will be identified. In this subgroup, claims data and self-report data will be used to derive extrapolation factors for total healthcare utilization and cost across all study participants. Additionally, for participants in other plans or uninsured, we will use M/M data to assign average cost to each of the self-reported items.

10. STATISTICAL ANALYSIS PLAN AND SAMPLE SIZE

10.1 Sample Size Determination

All trials power analyses are based on a modified intent to treat analysis, comparing the subset of intervention participants with have an actionable phenotype to the subset of control participants with an actionable phenotype. Effect sizes used to estimate power are based on prior studies of pain and depression pharmacogenetic testing and described below for each trial and shown in Table 8. Several steps were taken to generate a conservative sample size estimate. First, the power requirement was set to 90%. Second, sample sizes are adjusted for a 10% drop-out (for which UF has data from several studies that this is a good estimate). Third, slightly smaller effect sizes than what was observed in prior studies was used. Finally, sample size calculations are based on an alpha of 0.049, to allow one interim analysis with an alpha of 0.001 in each study.

Due to the study design and the delay in the control arm to obtaining the genotyping results required determine the actionable phenotype subgroup assignment, it is not feasible to directly monitor the number of randomized participants in the actionable subgroup for the enrollment stopping rule. Alternatively, the observed trial specific actionable phenotype percentages and the corresponding trial specific total enrollment targets will be used to identify when enrollment can conclude for a fully powered mITT study.

Acute Pain

In the University of Florida's pilot acute pain study, the difference between study arms in SIA score was –38.55 with a SD of 93.5, for a standardized effect size of 0.412. Assuming a reduced standardized effect size of 0.375, 304 participants with an actionable phenotype per group, 152 from the intervention arm and 152 from control arm, are required to achieve 90% power for a two-sided two-sample t-test. Preliminary data from the University of Florida's acute pain study indicates the proportion of participants with an actionable phenotype, genotypic or pheno-converted *CYP2D6* IM or PM, will be approximately 18%. After adjusting for the subset anticipated to have an actionable phenotype, 845 participants completing all assessments, per group, are required to achieve 90% power in the sub-group analysis (1678 total). Assuming 10% drop out or lost to follow-up and 7% of participants not going on to surgery, a total of 2020 randomized participants is required to sufficiently power this study. Factoring in variability in the proportion that are IMs or PMs (15% to 24%), 1516 to 2424 randomized subjects would be need to achieve 90% power in the mITT analysis.

Chronic Pain: In the University of Florida's prior chronic pain study, the difference between study arms in composite pain score was 0.6 with a SD of 1.4, for a standardized effect size of 0.43. The same effect size was observed when assessing 3-month changes in pain scores. Assuming a standardized effect size of 0.40, 268 participants within the specified subgroup, 134 from the intervention arm and 134 from control arm, are required to achieve 90% power for a two-sided two-sample t-test. Preliminary data indicate the proportion of participants with an actionable phenotype will be 30-35%; 10-15% of participants with an actionable genotype-based phenotype (*CYP2D6* IM or PM) and an additional 20% with a pheno-converted PM/IM based on drug-drug-gene interactions. After adjusting for the subset anticipated to have an actionable phenotype, 447 participants completing all assessments, per group, are required to achieve 90% power in the sub-group analysis (894 total). Assuming 10% drop out or lost to follow-up, a total of 994 randomized participants is required to sufficiently power this study. Factoring in variability of the proportion of randomized population that are IMs or PMs (24-33%), 906 to 1244 randomized subjects would be need to achieve 90% power in the mITT analysis.

Depression:

In prior studies of depression, the difference between study arms in PROMIS 8a T-scores was –6.7 with a SD of 10, for a standardized effect size of -.67. For the purposes of power analysis, a 4-point change is considered the minimally important difference representing a response to therapy that is congruent with the predicted CYP enzyme phenotype compared to the response to therapy which

conflicts with the phenotype.

Preliminary data from published studies indicate the proportion of participants with an actionable phenotype (CYP2C19 PM, RM, UM or CYP2D6 PM or UM, or pheno-converted *CYP2D6* PM or UM) will be 40%. However, due to the distribution of study-related antidepressants in primary care and behavioral health settings, an estimated 67% of the participants with an actionable phenotype are anticipated to also have a study-related dosing or drug selection intervention. In other words, the analytic group of CYP2DC UM, PM, or CYP2C19 UM, RM, or PM phenotypes will represent a mixture of participants that may have a clinically meaningful change in depression due to being prescribed a study-relevant antidepressant and participants that may have changes in depression typical of the standard of care arm since they are not being prescribed a study-relevant antidepressant. In accounting for this population mixture, we incorporated a dilution factor of 67% into the clinically meaningful effect size projection and assume the effective difference of 0.28, representing a 2.8-point change to the T-score. We will need 542 modified intent to treat participants, 271 in each arm, to detect a standardized effect size of 0.28 with 90% power. After adjusting for the subset anticipated to have an actionable phenotype (i.e. 40%), 678 participants completing all assessments, per group, are required to achieve 90% power in the mITT analysis (1356 total). After accounting for 90% retention, 754 participants from the intervention arm and 754 participants from control arm (1508 total) are required to achieve 90% power for a two-sided two-sample t-test. Factoring in variability for the on the mITT proportion of the randomized population (40-60%), 1006-1508 randomized subjects would be needed to achieve 90% power in the mITT analysis.

Table 9. Sample size calculations

Sample size calculations for each trial, assuming an alpha of 0.049 to allow for one interim analysis

**Study**	**Assumed Actionable Phenotype**	**Total N**	**Completed N (90% assumed)**	**Intervention**	**Usual Care**	**Intervention: Actionable Phenotype**	**Usual Care: Actionable Phenotype**	**Effect Size**	**Power**
Acute Pain (*CYP2D6* IM/PM)	18% (15%-24%)	2,020 (2424-1516)	1,690* (2028 - 1268)	845 (1014 - 634)	845 (1014 - 634)	152	152	0.375	90.00%
Chronic Pain (*CYP2D6* IM/PM)	30% (24% - 33%)	994 (906 - 1244)	894 (814-1118)	447 (407-559)	447 (407-559)	134	134	0.4	90.00%
Depression (*CYP2D6* or *CYP2C19* PM/UM)	40% (up to 60%)	1,508 (1006)	1,356 (904)	678 (452)	678 (452)	271	271	0.28	90.00%
**Total**		4,522	3,940	1,970	1,970	557	557		

*Accounts for an additional 7% of participants not completing surgery

10.2 General Statistical Methods

A detailed statistical analysis plan will be developed for each trial (acute pain, chronic pain, and

depression) and contained in a separate document. Prior to analysis, each trial study population details, including the number randomized, in each treatment arm, and lost to follow-up will be described. Baseline participant characteristics will be summarized as means, standard deviations, medians, and 25th, 75th percentiles for continuous variables, and as counts and percentages for categorical variables. Model assumptions will be examined prior to analysis and transformations implemented, if necessary, to more adequately meet the assumptions. Unless otherwise stated, hypotheses will be tested as two-sided with a significance level of 0.05.

10.3 Population for Analyses

Acute pain
Participants randomized who have an actionable phenotype, specifically participants who are genotypically or pheno-converted CYP2D6 IM or PMs (i.e. CYP2D6 activity score ≤ 0.75).

Chronic pain
Participants randomized who have an actionable phenotype, specifically participants who are genotypically or pheno-converted CYP2D6 IM or PMs (i.e. CYP2D6 activity score ≤ 0.75).

Depression
Participants randomized who have an actionable phenotype, specifically participants who are genotypically or pheno-converted CYP2D6 PM or UMs or CYP2C19 PM, RM, or UMs.

10.4 Analysis of the Primary Endpoint

Acute Pain
To determine the effect of genotype-guided drug therapy on pain control in acute pain participants, we will conduct a subset analysis comparing SIA scores at 10-days post-surgery in the Intervention group subset with CYP2D6 IM or PM phenotypes to the Control group subset with CYP2D6 IM or PM phenotypes. Participants that have other CYP2D6 phenotypes will not be included. The 10-day post-surgery SIA scores of the Intervention CYP2D6 IM/PM group will be compared to the 10-day post-surgery SIA scores of the Control CYP2D6 IM/PM group using a two-sided two-sample t-test or Mann Whitney test, as appropriate, with a type 1 error of 0.049. This primary analysis will be conducted as a modified intent-to-treat analysis, with participants analyzed and endpoints attributed according to the treatment arm to which the participants were randomized, regardless of subsequent crossover or post-randomization medical care.

Chronic Pain

To determine the effect of genotype-guided drug therapy on pain control in chronic pain participants, we will conduct a subset analysis comparing change in pain intensity score from baseline to 3-month follow-up in the Intervention group subset with CYP2D6 IM or PM phenotypes to the Control group subset with CYP2D6 IM or PM phenotypes. Participants that have other CYP2D6 phenotypes will not be included. The baseline to 3-month change in pain intensity of the Intervention CYP2D6 IM/PM group will be compared to the baseline to 3-month change in pain intensity of the Control CYP2D6 IM/PM group using a two-sided two-sample t-test or Mann Whitney test, as appropriate, with a type 1 error of 0.049. This primary analysis will be conducted according to modified intent-to-treat design, with participants analyzed and endpoints attributed according to the treatment arm to which the participants were randomized, regardless of subsequent crossover or post-randomization medical care.

Depression
To determine the effect of genotype-guided drug therapy on depression symptoms in depression

participants, we will conduct a subset analysis comparing change in depression T-scores from baseline to 3-month follow-up in the Intervention group subset with either CYP2D6 PM or UM or CYP2C19 PM, RM or UM phenotypes to the Control group subset with CYP2D6 PM or UM or CYP2C19 PM, RM, or UM phenotypes. Participants that have other CYP2D6 or CYP2C19 phenotypes will not be included. Pediatric depression T-scores will be transformed to the adult scale using a published crosswalk for a combined analysis. The baseline to 3-month change in depression T-scores of the Intervention CYP2D6 PM/UM or CYP2C19 PM/RM/UM group will be compared to the baseline to 3-month change in depression T-scores of the Control CYP2D6 PM/UM or CYP2C19 PM/RM/UM subgroup using a two-sided two-sample t-test with a type 1 error of 0.049. This primary analysis will be conducted according to a modified intent-to-treat design, with participants analyzed and endpoints attributed according to the treatment arm to which the participants were randomized, regardless of subsequent crossover or post-randomization medical care.

10.5 Analysis of the Secondary Endpoints

All Trials

To determine the effect of genotype-guided drug therapy on overall well-being and the well-being sub-domains (pain interference, physical function, sleep disturbance, social role and activities functioning, fatigue, anxiety, and depression) in acute pain, chronic pain, and depression participants, we will conduct an intent to treat analysis, comparing the well-being (or sub-domain) T-scores from the 6-month follow-up assessments in the Intervention group compared to the control group using a two-sided two-sample t-test or Mann Whitney test, as appropriate, stratified by participant population (acute pain, chronic pain, depression), with a type 1 error of 0.05.

Acute Pain

The effect of genotype-guided opioid therapy on secondary endpoints pain intensity, opioid usage, opioid misuse score, and mobility score will be assessed by comparing the Intervention group subset with CYP2D6 IM or PM phenotypes to the Control group subset with CYP2D6 IM or PM phenotypes using a two-sided two-sample t-test or Mann Whitney test, as appropriate, with a type 1 error of 0.05. The effect of genotype guided opioid therapy on the secondary endpoint opioid persistence will be assessed by comparing the Intervention group subset with CYP2D6 IM or PM phenotypes the Control group subset with CYP2D6 IM or PM phenotypes using a test of two-proportions with an alpha of 0.05. Participants with other CYP2D6 phenotypes will not be included in these analyses.

Chronic Pain

The effect of genotype-guided opioid therapy on secondary endpoints pain intensity reduction and opioid misuse score will be assessed by comparing the Intervention group subset with CYP2D6 IM or PM phenotypes to the Control group subset with CYP2D6 IM or PM phenotypes using a two-sided two-sample t-test or Mann Whitney test, as appropriate, with a type 1 error of 0.05. The effect of genotype guided opioid therapy on the secondary endpoint clinically significant pain reduction, defined as a 30% decrease, will be assessed by comparing the Intervention group subset with CYP2D6 IM or PM phenotypes the Control group subset with CYP2D6 IM or PM phenotypes using a test of two-proportions with an alpha of 0.05. Participants that have other CYP2D6 phenotypes will not be included in these analyses.

Depression

The effect of genotype guided antidepressants therapy on the secondary endpoint depression remission and achieving 5-% reduction in PHQ-8 scores will be assessed by comparing the Intervention group subset with CYP2D6 UM or PM or CYP2C19 UM, RM, or PM phenotypes the

Control group subset with CYP2D6 UM or PM or CYP2C19UM, RM, or PM phenotypes using a test of two-proportions with an alpha of 0.05. The effect of genotype guided antidepressants therapy on the secondary endpoints, medication adherence, PHQ-8 scores, and medication side effect severity burden, will be assessed by comparing the Intervention group subset with CYP2D6 UM or PM or CYP2C19 UM, RM, or PM phenotypes the Control group subset with CYP2D6 UM or PM or CYP2C19 UM, RM, or PM phenotypes using a two-sided Mann Whitney test with an alpha of 0.05. Participants that do not have metabolizer phenotypes will not be included in these analyses.

10.6 Other Planned Analyses

Exploratory endpoints or comparisons of endpoints that will be considered:

- Medication side effects
- Comparisons of measures of depression and/or pain
- Provider actions taken after CDS alerts

Sub-groups that will be considered:

- All randomized participants
- Metabolizer phenotypes: UMs, RMs, NMs, IMs, and PMs.
- CYP2D6 or CYP2C19 activity scores
- Pediatric participants
- Adult participants
- “Per-protocol” - participants with both an actionable phenotype and concordance between the recommended medication/dosage and the prescribed medication and/or dosage
- Participants with an actionable phenotype, a discordant medication at baseline, and a concordant medication at the end of the study
- Stratified by medication, medication class and/or medications combinations
- Stratified by demographic characteristics (e.g. age, sex, gender and/or race)
- Stratified by type and/or design of CDS implemented
- Stratified by institution, enrolling site, and/or practice setting specialty (e.g. psychiatry, primary care, etc.)
- And other relative combinations of the above sub-groups

In addition to the primary research exploratory analyses, we will also analyze the uptake and utilization of the clinical decision support (CDS) tools that were created and its role in any medication changes made by providers. We will examine the impact of the CDS tools on various primary and secondary outcomes. We may also describe the development of the CDS tools, with a specific focus on the automated phenoconversion calculations implemented by some sites. As part of these analyses, we may collect and/or include the following data points: socio-demographics, health care characteristics (i.e. comorbidities), site ID, clinical group ID, CDS related data points (i.e. alert ID, CDS name, CDS alert type, date triggered, provider action), prescriber specialty, medication details (i.e. triggering medications, medication or dose changes, medication concordance/discordance), genotype and phenoconversion results.

We will also analyze reach, adoption, and implementation of the trial and PGx testing. We will examine enrollment and screening information, missing data rates, visit completion information. We will also analyze patient preferences for contact and survey administration, in addition to survey completion information

Specified analyses:

<u>All Trials</u>

Medication side effects. The frequency and severity of medication side effects within each trial and trial arm will be summarized using standard descriptive statistics for discrete data (counts and percentages).

<u>Acute Pain</u>
Trends in endpoints. In addition to the primary and secondary endpoints described above, we will compare the time trends in the endpoints: pain scores, opioid usage, opioid misuse score, and mobility score at baseline, 1, 3, and 6 months among the CYP2D6 IM or PM subgroup, using a repeated-measures mixed effect model or generalized repeated-measures mixed effect model, as appropriate. Since participants are randomized to Intervention and Control groups and not to the CYP2D6 IM or PM subgroup within Intervention and Control groups, there could be important differences in baseline characteristics between the two groups. For these reasons, we will conduct covariate-adjusted repeated measures mixed models that will account for differences in baseline characteristics that differ between the Intervention – CYP2D6 IM or PM subgroup and the Control – CYP2D6 IM or PM subgroup. Potential baseline characteristics may include age, sex, income, education, insurance, surgical procedure type. We will select the appropriate covariance matrix (e.g. compound symmetry, autoregressive, unstructured, or other covariance structure) based on the data.

Difference within the NM group. In the control arm participants, we will compare pain scores in participants with a CYP2D6 activity score >0.75 – 1 to participants with an activity scores >1-2 using a two-sided two-sample t-test or Mann Whitney test, as appropriate, with a type 1 error of 0.05. Additionally, we will use linear regression to adjust for differences in baseline characteristics.

<u>Chronic Pain</u>
Trends in endpoints. In addition to the primary and secondary endpoints described above, we will compare the time trends in the endpoints: pain control, pain reduction, clinically significant pain reduction, and opioid misuse score at baseline, 1, 3, and 6 months among the CYP2D6 IM or PM subgroup, using a repeated-measures mixed effect model or generalized repeated-measures mixed effect model, as appropriate. Since participants are randomized to Intervention and Control groups and not to the CYP2D6 IM or PM subgroup within Intervention and Control groups, there could be important differences in baseline characteristics between the two groups. For these reasons, we will conduct covariate-adjusted repeated measures mixed models that will account for differences in baseline characteristics that differ between the Intervention – CYP2D6 IM or PM subgroup and the Control – CYP2D6 IM or PM subgroup. Potential baseline characteristics may include age, sex, income, education, insurance, and medical conditions. We will select the appropriate covariance matrix (e.g. compound symmetry, autoregressive, unstructured, or other covariance structure) based on the data.

Difference within the NM group. In the control arm participants, we will compare pain scores and pain control in participants with a CYP2D6 activity score >0.75 – 1 to participants with an activity scores >1-2 using a two-sided two-sample t-test or Mann Whitney test, as appropriate, with a type 1 error of 0.05. Additionally, we will use linear regression to adjust for differences in baseline characteristics.

<u>Depression</u>
Trends in endpoints. In addition to the primary and secondary endpoints described above, we will compare the time trends in the endpoint depression symptom scores at baseline, 1, 3, and 6 months among the CYP2D6 PM or UM or CYP2C19 UM, RM or PM subgroup, using a repeated-measures mixed effect model. Since participants are randomized to Intervention and Control groups and not to the CYP2D6 PM or UM or CYP2C19 UM, RM or PM subgroup within Intervention and Control groups, there could be important differences in baseline characteristics between the two groups. For these

reasons, we will conduct covariate-adjusted repeated measures mixed models that will account for differences in baseline characteristics that differ between the Intervention – CYP2D6 PM or UM or CYP2C19 UM, RM or PM subgroup and the Control – CYP2D6 PM or UM or CYP2C19 UM, RM or PM subgroup. Potential baseline characteristics may include age, sex, income, education, insurance, medical conditions. We will select the appropriate covariance matrix (e.g. compound symmetry, autoregressive, unstructured, or other covariance structure) based on the data.

Difference within the NM group. In the control arm participants, we will compare depression scores and depression control in participants with a CYP2D6 activity score >0.75 – 1 to participants with an activity scores >1-2 using a two-sided two-sample t-test or Mann Whitney test, as appropriate, with a type 1 error of 0.05. Additionally, we will use linear regression to adjust for differences in baseline characteristics

<u>Cost Effectiveness</u>
Healthcare costs: In the Medicare fee-for-service or Medicaid enrollees, log-transformed overall expenditures (between index visit and 6 months follow-up) will be compared using a 2-sample t-test between the study arms (after ensuring that the variances in the log-scale are equal). Alternatively, cost comparisons will be made using appropriate regression models such as generalized linear models. For all participants, regardless of insurance type, we will estimate and aggregate participant-reported healthcare utilization during follow-up and assign cost for each item based on unit weighted mean cost estimates from the Medicare/ Medicaid data.

Within the Medicare/Medicaid population, we will compare estimated cost differences between genotype-guided and usual care based on billing records versus participant report to further validate the cost comparisons for the entire population. Cost for the index hospitalization for surgery participants will not be included in the cost estimate, but we will compare length of stay between groups as reference for hospitals regarding potential cost savings in capitation-based reimbursement schemes. All costs will be converted to 2021 $US using the chain-weighted Consumer Price Index (CPI).

Finally, we will compare the Medicare/Medicaid beneficiaries among our trial enrollees to a larger random sample of beneficiaries in the participating states and compare demographic and key clinical characteristics to explore representativeness of our study sample, evaluate the impact of attrition and loss to follow-up and make inferences about the generalizability of our results.

Cost utility: For the cost-utility analysis, the PROMIS-43 responses will be converted into utilities using previously validated crosswalks.[41] Cost and quality adjusted life year (QALY) estimates, adjusted for clustering within site and baseline utility for QALYs, will be obtained using appropriate regression models such as generalized linear models. The analysis of maximum likelihood parameter estimates will allow us to capture differences in cost and QALYs between the intervention arm and control arm of the trial. The Incremental cost-effectiveness ratio (ICER) will be calculated from costs and QALY. A bootstrapping approach will be used to characterize sampling uncertainty and calculate confidence intervals (CI) around the ICER estimate ($2.5^{th}$and 97.5^{th}percentiles corresponding to the lower and upper bounds of the CI, respectively). This sampling uncertainty will be summarized using cost-effectiveness acceptability curves.

Details of these statistical analyses, along with other exploratory analyses will be described in the study SAP.

10.7 Interim Analyses

A single specified interim analysis is planned for each trial. To account for repeated significance testing of the accumulating data, the group sequential method of Lan and DeMets will be used as a guide for

interpreting this interim analysis. Monitoring boundaries for the primary endpoint will be based on a two-sided symmetric O'Brien-Fleming type spending function with an overall two-sided significance level of α=0.05. The O'Brien-Fleming approach requires large critical values early in the study but relaxes (i.e., decreases) the critical value as the trial progresses.

Acute Pain
The interim analysis of the acute pain primary endpoint will compare SIA scores at 10 days post-surgery among participants with *CYP2D6* IM or PM phenotypes in the Control arm to participants with *CYP2D6* IM or PM phenotypes in the intervention arm.

Chronic Pain

The interim analysis of the chronic pain primary endpoint, pain control at 3 months post return of PGx testing result to providers, will be compared between the participants with *CYP2D6* IM or PM phenotypes in the Control arm and the participants with *CYP2D6* IM or PM phenotypes in the intervention arm.

Depression
The interim analysis of the depression primary endpoint, depression control at 3 months post return of PGx testing result to providers, will compare the participants with *CYP2D6* PM/UM or *CYP2C19* PM, RM and UM phenotypes in the Control arm to the participants with *CYP2D6* PM/UM or *CYP2C19* PM/RM/UM phenotypes in the intervention arm.

The primary analysis is a modified intent to treat design and the analytical subgroup is defined by the results of the PGx testing. Because the drug metabolizer phenotypes for participants in the Control arm is determined around the time of the 6-month follow up assessments for all trials (Acute Pain, Chronic Pain, and Depression), only participants that have completed their 6-month assessments will be included in the interim analysis. To account for the delays in receiving genetic testing results, the interim analysis will be targeted to occur when approximately 50% of the participants have completed their 6-month assessments. If the interim analysis does not propose stopping for efficacy (p-value <0.0003 and the intervention has the larger reduction in the primary endpoints), then the conditional power to detect a significant result at the end of the trial will be estimated. The conditional power will be presented to the DSMB to facilitate discussion of whether the trial should be stopped for futility.

10.8 Handling of Missing Data

For all primary and secondary statistical analyses described above, multiple imputation will be used for all missing values except those due to participant death. To ensure that the missing at random assumption for multiple imputation is valid, we will compare baseline pain or depression scores and other patient characteristics in those lost to follow up to participants retained on their randomization assignment. We will also do sensitivity analyses where we compare results obtained with multiple imputation to those obtained without imputation. Missing values due to death will not be imputed and will therefore not be included in the analyses. For exploratory repeated measures analyses, missing data are easily handled as long as the missing at random assumption is valid. However, the missing at random assumption cannot be tested. Accordingly, for exploratory analyses using repeated measures mixed models, we will also conduct a sensitivity analysis where missing values are imputed using multiple imputation. The results with and without multiple imputation will be compared. More complete details of the handling of missing values under different circumstances will be described in the SAP.

10.9 Multiplicity

With the primary and various secondary endpoints that have been outlined, there is a multiplicity of analyses to be performed, which leads to an increased probability that at least one of the comparisons could be "significant" by chance. Adjusting for the effects of the repeated significance testing for the

multiplicity of secondary endpoints would require that very small significance levels be used for every comparison. Therefore, rather than adjusting for multiple comparisons, we will be conservative in the interpretation of the analyses, considering the degree of significance, and looking for consistency across endpoints. The nominal (unadjusted) p-value for each comparison will be reported to aid in the overall interpretation. We have also prespecified the primary and secondary outcome variables to avoid over-interpretation of strictly exploratory comparisons.

11. DATA MANAGEMENT

11.1 Data Entry and Record Keeping

ADOPT PGx data sources include: data collected from participant surveys, genetic testing results, data from the EHR, and CMS billing records. Data will be entered into a REDCap database maintained by the CC by trained and qualified personnel at each clinical site for baseline and follow-up assessments and by the UF call center for follow-up assessments. Site staff will receive training on the use of the REDCap database. After staff are trained, they will receive a unique user identification and password to access data entry forms for their site. Access codes should not be shared and are non-transferable.

Laboratories will transfer genetic testing results to the IGNITE PTN CC and follow standard site-specific return of results procedures for providers. The genotype and result (i.e. metabolizer phenotype) for each participant will then be imported to the participant's study database record from laboratory produced output files. The accuracy of the import of laboratory results will be verified by comparing the results recorded in the study database to an external record of the result for a subset of the study participants. See MOP for details.

Sites will extract the relevant data from their EHR (see Section 7.7) and transfer of those data to the IGNITE PTN CC following procedures that will be specified in the MOP. Briefly, sites will be given a list of trial-specific information to extract for their ADOPT PGx participants, a common format that data should be converted to, and procedures for secure file transfer to the CC.

11.2 Data Element Definitions

Clinically significant pain reduction: Whether or not the pain reduction score is ≤ 0.7. If the pain reduction score is ≤0.7, clinically significant pain reduction is achieved, if pain reduction scores is > 0.7, clinically significant pain reduction is not achieved.

Depression symptom control: The change in the PROMIS Emotional Distress - Depression 8b or PROIMIS pediatric Depression 8a survey depression score from baseline to time t.

Depression remission: A. Whether or not the summed raw PROMIS emotional distress depression 8bsurvey responses are <= 16, which is equivalent to the participant responding "rarely" or "never" tomost or all of the PROMIS emotional distress - depression 8b survey questions. B. Whether or not the PHQ-8 scores are ≤ 4, which is equivalent to depression severity being none-mild.

Depression (PHQ-8) score: The sum of the responses to the PHQ-8 survey, range from 0 to 24.

Depression (T) score: The T-score converted summed responses to the PROMIS emotional distress depression 8b survey. The T-score conversion is centered around 50 with standard deviation of 10.

Depression severity: The depression score converted to depression severity (none, mild, moderate, severe) based on the scoring guide for the PROMIS emotional distress depression 8b survey.

Medication adherence: The scored responses to the Voils Medication Adherence survey.

Medication concordance: Whether or not the medication the participant is prescribed after the intervention is concordant with the participants CYP2D6 and, for the depression trial, CYP2C19 phenotype.

Medication side effects severity burden: (Depression only) The sum of the scored severity (none = 0, mild = 1, moderate = 2, severe = 3) for common SSRI side effects.

Opioid misuse score: The T-score converted summed responses to the PROMIS prescription medication misuse 7a survey. The T-score conversion is centered around 50 with standard deviation of10.

Opioid persistence: Defined as ≥1 opioid prescription refill between 90 days and 180 days after the surgical procedure.

Opioid usage: The average daily morphine equivalents in mg (MED) in past 7 days. MEDs will be calculated using data from the opioid usage survey.

Overall well-being: Defined as the T-score converted summed responses to the PROMIS 43/PROMIS 37 pediatrics survey. The T-score conversion is centered around 50 with standard deviation of 10.

Pain control: The difference in pain intensity score at follow-up time t and the baseline pain intensity score.

Pain intensity score: The mean of average pain in the last 7 days, current pain, and worst pain in the last 7 days. Average pain, current pain, and worst pain will be collected using the 3-question PROMIS pain intensity scale.

Pain reduction score: The ratio of the pain intensity score (derived from the PROMIS pain intensity survey) at follow-up time t to the baseline pain intensity score.

Silverman integrative analgesic assessment (SIA) score: This is an integrated measure of pain and opioid usage, calculated as follows: pain and opioid usage are ranked, the ranks are converted to percentiles and linearly transformed such that the scores are centered at 0 and range -100 to 100. The pain scores and opioid usage transformed percentiles are summed to generate the SIA score that ranges -200 to 200. Negative values indicate low pain with minimal opioid usage, while positive values indicate higher pain with higher opioid usage. See Appendix A for additional details and supporting rationale.

11.3 Database Management and Quality Control of Data

The IGNITE PTN CC will develop and manage the ADOPT PGx study database and perform internal database quality-control checks. The CC will conduct data audits throughout the course of the trial. These audits are intended to identify data errors, protocol deviations, failure of standardization, missing data, or inconsistencies. Any out-of-range values and missing or inconsistent key variables are flagged and addressed/answered at the site in real time during the data entry process.

The CC will periodically perform additional data quality checks in SAS. Clinical sites may also be given regular feedback directly to discuss issues identified by QC assessments.

11.4 Database Lock and Study Close Out

The end of the study is defined as the completion of the final participant follow-up. The designated central clinical research monitor with oversight by the CC will coordinate participating site close-out process according to the Clinical Monitoring Plan (See MOP). The CC will follow the database lock process in the study Data Management Plan.

11.5 Data Sharing

In accordance with the NIH Genomic Data Sharing policy, https://grants.nih.gov/grants/guide/notice-files/NOT-HG-19-024.html deidentified genotypes, linked phenotype, and clinical outcome data, excluding Medicare or Medicaid claims data, will be deposited in an NIH-designated data repository.

12. ETHICAL AND HUMAN SUBJECTS CONSIDERATIONS

12.1 Institutional Review Board/Ethics Committee Review

This study will be initiated after all required documentation has been reviewed and approved by the central IRB according to national and international regulations. All participating sites will be required to have central IRB approval prior to activation. The CC will be responsible for the coordination of all IRB activities.

12.2 Use and Disclosure of Protected Health Information (HIPAA)

For clinical trial sites, an authorization for the use and disclosure of protected health information (PHI) under the HIPAA Privacy Rule [45 CFR § 164.102 *et seq*] will be obtained from every trial participant prior to, or at the time of, enrollment. HIPAA Authorization may either be a separate form or included in the study ICF, dependent upon local requirements. It will be presented to, and signed by, the subject at the same time as the Informed Consent Form (ICF). See Section 6.4 Participant Consent Process .

12.3 Confidentiality and Privacy

Participant confidentiality will be maintained throughout the clinical study in a way that ensures the information can always be tracked back to the source data. For this purpose, a unique subject identification (ID number) will be used that allows identification of all data reported for each subject. Subject information collected in this study and all records will be kept confidential and the subject's name will not be released by study staff at any time.

Clinical data will be entered into a data entry system provided by the CC. The data system includes password protection and internal quality checks, such as automatic range checks to identify data that appear inconsistent, incomplete, or inaccurate. All data collection and storage devices will be password protected with a strong password and all sensitive research information on portable devices will be encrypted. Access to identifiable data will be limited to members of the study team. If it is necessary to use portable devices for initial collection of identifiers, the data files will be encrypted and the identifiers moved to a secure system as soon as possible. The portable device(s) will be locked up in a secure location when it is not in use.

12.4 Publication and Data Sharing Policies

The IGNITE PTN will have a Publications and Presentations Committee (PPC) with the primary responsibility for coordinating, monitoring, and reviewing all publications and presentations resulting from IGNITE PTN studies. In addition, the PPC will oversee the review, approval, and supervision of the secondary analyses and ancillary studies that are conducted within the Network. The goal of the PPC is to facilitate dissemination of the maximum amount of information from these studies in a scientifically sound and ethically responsible fashion in accordance with the unique nature of the IGNITE PTN mission. The IGNITE PTN Coordinating Center will draft a PPC charter in collaboration with NHGRI, PPC, and the IGNITE PTN which specifies the publication policies and procedures. The primary outcomes from each trial, Acute Pain, Chronic Pain, Depression, will be published as separate publications.

The sharing of datasets will be performed per DCRI SOPs and requirements for NIH policy for data

sharing, and guidelines for NIH Data Set Preparation. The de-identified or anonymized data, excluding Medicare and Medicaid claims data, and documentation in standardized formats will be madeavailable in an NIH-designated data repository for sharing to the larger scientific community. Requested unrestricted data may be made available after database lock to parties who sign a data sharing agreement, which stipulates that data must be: 1) used solely for research purposes, 2) properly acknowledged in resulting publications, 3) kept confidential and inaccessible to third parties, and 4) destroyed or returned after analyses are completed. Additionally, users must agree not to use data toidentify individual participants.

13. PROTOCOL DEVIATIONS AND VIOLATIONS

A protocol deviation is defined as an event where the Investigator or site personnel did not conduct the study according to the protocol or the Investigator Agreement. A protocol violation is an intentional act in which the protocol is not followed. (See Protocol Section 8 for IRB reporting timelines)

Protocol deviations and violations will be reported to the IRB if it affects

- subject rights and welfare
- affects subject safety
- affects the integrity of study data
- affects the subject's willingness to continue in the study
- is specifically requested by a government agency, internal/external auditor, medical monitor, or the IRB.

REFERENCES

1. Smith, D.M., et al., *CYP2D6-guided opioid therapy improves pain control in CYP2D6 intermediate and poor metabolizers: a pragmatic clinical trial.* Genet Med, 2019.
2. Gaskin, D.J. and P. Richard, *The economic costs of pain in the United States.* J Pain, 2012. **13**(8): p. 715-24.
3. *Relieving Pain in America: A Blueprint for Transforming Prevention, Care, Education, and Research.* Mil Med, 2016. **181**(5): p. 397-9.
4. Aubrun, F., et al., *Relationships between measurement of pain using visual analog score and morphine requirements during postoperative intravenous morphine titration.* Anesthesiology, 2003. **98**(6): p. 1415-21.
5. Bruehl, S., et al., *Personalized medicine and opioid analgesic prescribing for chronic pain: opportunities and challenges.* J Pain, 2013. **14**(2): p. 103-13.
6. Guy, G.P., Jr., et al., *Vital Signs: Changes in Opioid Prescribing in the United States, 2006-2015.* MMWR Morb Mortal Wkly Rep, 2017. **66**(26): p. 697-704.
7. *U.S. Prescribing Rate Maps*. 2017 2017-10-5]; Available from: https://www.cdc.gov/drugoverdose/maps/rxrate-maps.html.
8. Levy, B., et al., *Trends in Opioid Analgesic-Prescribing Rates by Specialty, U.S., 2007-2012.* Am J Prev Med, 2015. **49**(3): p. 409-13.
9. Informatics, M.I.f.H. *Medication use and athe shifting cost of healthcare - a review of use of medicine in teh United States in 2013.* [cited 2017; Available from: https://democrats-oversight.house.gov/sites/democrats.oversight.house.gov/files/documents/IMS-Medicine%20use%20and%20shifting%20cost%20of%20healthcare.pdf.
10. Crews, K.R., et al., *Clinical Pharmacogenetics Implementation Consortium guidelines for cytochrome P450 2D6 genotype and codeine therapy: 2014 update.* Clin Pharmacol Ther, 2014. **95**(4): p. 376-82.
11. Ciszkowski, C., et al., *Codeine, ultrarapid-metabolism genotype, and postoperative death.* N Engl J Med, 2009. **361**(8): p. 827-8.
12. Gasche, Y., et al., *Codeine intoxication associated with ultrarapid CYP2D6 metabolism.* N Engl J Med, 2004. **351**(27): p. 2827-31.
13. Kelly, L.E., et al., *More codeine fatalities after tonsillectomy in North American children.* Pediatrics, 2012. **129**(5): p. e1343-7.
14. Koren, G., et al., *Pharmacogenetics of morphine poisoning in a breastfed neonate of a codeine-prescribed mother.* Lancet, 2006. **368**(9536): p. 704.
15. Orliaguet, G., et al., *A case of respiratory depression in a child with ultrarapid CYP2D6 metabolism after tramadol.* Pediatrics, 2015. **135**(3): p. e753-5.
16. Adams, E.H., et al., *A comparison of the abuse liability of tramadol, NSAIDs, and hydrocodone in patients with chronic pain.* J Pain Symptom Manage, 2006. **31**(5): p. 465-76.
17. Preston, K.L., D.R. Jasinski, and M. Testa, *Abuse potential and pharmacological comparison of tramadol and morphine.* Drug Alcohol Depend, 1991. **27**(1): p. 7-17.
18. Taylor, W.D., *Clinical practice. Depression in the elderly.* N Engl J Med, 2014. **371**(13): p. 1228-36.
19. Noordam, R., et al., *Identifying genetic loci associated with antidepressant drug response with drug-gene interaction models in a population-based study.* J Psychiatr Res, 2015. **62**: p. 31-7.

20. Thase, M.E., et al., *Remission rates following antidepressant therapy with bupropion or selective serotonin reuptake inhibitors: a meta-analysis of original data from 7 randomized controlled trials.* J Clin Psychiatry, 2005. **66**(8): p. 974-81.
21. Singh, A.B., *Improved Antidepressant Remission in Major Depression via a Pharmacokinetic Pathway Polygene Pharmacogenetic Report.* Clin Psychopharmacol Neurosci, 2015. **13**(2): p. 150-6.
22. Hall-Flavin, D.K., et al., *Utility of integrated pharmacogenomic testing to support the treatment of major depressive disorder in a psychiatric outpatient setting.* Pharmacogenet Genomics, 2013. **23**(10): p. 535-48.
23. Bradley, P., et al., *Improved efficacy with targeted pharmacogenetic-guided treatment of patients with depression and anxiety: A randomized clinical trial demonstrating clinical utility.* J Psychiatr Res, 2018. **96**: p. 100-107.
24. Bousman, C.A., et al., *Pharmacogenetic tests and depressive symptom remission: a meta-analysis of randomized controlled trials.* Pharmacogenomics, 2019. **20**(1): p. 37-47.
25. Winner, J.G., et al., *A prospective, randomized, double-blind study assessing the clinical impact of integrated pharmacogenomic testing for major depressive disorder.* Discov Med, 2013. **16**(89): p. 219-27.
26. Perez, V., et al., *Efficacy of prospective pharmacogenetic testing in the treatment of major depressive disorder: results of a randomized, double-blind clinical trial.* BMC Psychiatry, 2017. **17**(1): p. 250.
27. Hicks, J.K., et al., *Clinical Pharmacogenetics Implementation Consortium (CPIC) Guideline for CYP2D6 and CYP2C19 Genotypes and Dosing of Selective Serotonin Reuptake Inhibitors.* Clin Pharmacol Ther, 2015. **98**(2): p. 127-34.
28. Administration, U.S.F.a.D. *Drug Development and Drug Interactions: Table of Substrates, Inhibitors and Inducers*. 2017 11/14/2017 [cited 2019; Available from: https://www.fda.gov/drugs/drug-interactions-labeling/drug-development-and-drug-interactions-table-substrates-inhibitors-and-inducers.
29. Zwisler, S.T., et al., *Impact of the CYP2D6 genotype on post-operative intravenous oxycodone analgesia.* Acta Anaesthesiol Scand, 2010. **54**(2): p. 232-40.
30. Andreassen, T.N., et al., *Do CYP2D6 genotypes reflect oxycodone requirements for cancer patients treated for cancer pain? A cross-sectional multicentre study.* Eur J Clin Pharmacol, 2012. **68**(1): p. 55-64.
31. Lemberg, K.K., et al., *Does co-administration of paroxetine change oxycodone analgesia: An interaction study in chronic pain patients.* Scand J Pain, 2010. **1**(1): p. 24-33.
32. Cella, D., et al., *PROMIS((R)) Adult Health Profiles: Efficient Short-Form Measures of Seven Health Domains.* Value Health, 2019. **22**(5): p. 537-544.
33. Pilkonis, P.A., et al., *An Item Bank for Abuse of Prescription Pain Medication from the Patient-Reported Outcomes Measurement Information System (PROMIS(R)).* Pain Med, 2017. **18**(8): p. 1516-1527.
34. Hays, R.D., et al., *Upper-extremity and mobility subdomains from the Patient-Reported Outcomes Measurement Information System (PROMIS) adult physical functioning item bank.* Arch Phys Med Rehabil, 2013. **94**(11): p. 2291-6.
35. Quinn, H., et al., *Using item response theory to enrich and expand the PROMIS pediatric self report banks.* Health and quality of life outcomes, 2014. **12**: p. 160.

36. Pilkonis, P.A., et al., *Item banks for measuring emotional distress from the Patient-Reported Outcomes Measurement Information System (PROMIS(R)): depression, anxiety, and anger*. Assessment, 2011. **18**(3): p. 263-83.
37. Forrest, C.B., et al., *Development and Evaluation of the PROMIS((R)) Pediatric Positive Affect Item Bank, Child-Report and Parent-Proxy Editions.* J Happiness Stud, 2018. **19**(3): p. 699-718.
38. Patient Health Questionnaire-8, 2006; Available from https://www.phqscreeners.com/select-screener
39. Voils, C.I., et al., *Content Validity and Reliability of a Self-Report Measure of Medication Nonadherence in Hepatitis C Treatment*. Dig Dis Sci, 2019. **64**(10): p. 2784-2797.
40. *PROMIS (Patient-Reported Outcomes Measurement Information System)*. 2020; Available from: http://www.healthmeasures.net/explore-measurement-systems/promis.
41. Hartman, J.D. and B.M. Craig, *Comparing and transforming PROMIS utility values to the EQ-5D*. Qual Life Res, 2018. **27**(3): p. 725-733.

14. APPENDICES

14.1 Appendix A. SIA Score Background and Rationale

For the primary endpoint, we propose to use a composite score that captures both opioid consumption (MED) and pain intensity. This is based on preliminary data from a University of Florida (UF) pilot study in which CYP2D6 genotype-guided opioid prescribing after arthroplasty surgery was compared to a usual care approach (ClinicalTrials.gov Identifier: NCT03534063). While not powered to detect differences in clinical endpoints, preliminary results from 174 patients showed similar pain intensity at 2-week post-surgery between genotype-guided and usual care groups, but significantly lower MMEs in the genotype-guided group. The combination of lower opioid consumption with similar pain intensity indicates better pain control in the genotype-guided arm. However, assessing opioid consumption and pain intensity as two separate variables fails to characterize the inter-individual differences in opioid use as pain intensity changes over time, which is why we believe a composite endpoint that integrates the two is justifiable.

The Silverman Integrating approach (SIA) score has been shown to provide superior statistical power with appropriate control of type 1 error compared to methods that integrate pain score and post-operative opioid consumption.[1, 2] When comparing the SIA score between genotype-guided and usual care arms in the UF study described above, the score indicated lesser pain despite fewer opioids consumed in the genotype-guided arm compared with greater pain despite more opioids consumed in the usual care group (p=0.07). To date, at least 11 clinical trials have used the SIA score in the post-operative setting.[3-13] While the majority utilized patient-controlled analgesia pumps, a pain expert at UF proposes the SIA score applicability warrants consideration in an acute post-surgical pain population. The SIA score is referenced in recommendations by the Initiative on Methods, Measurement, and Pain Assessment in Clinical Trials (IMMPACT), which aims to develop consensus recommendations for design and execution of clinical trials of pain management [14], as well as in a systematic review of predictive experimental pain studies.[15] However, both acknowledge that the SIA score is rarely used in pain research and that additional research is needed on its utility. While this approach is not part of current consensus recommendations for pain trials, consensus bodies have expressed interest in its utility. Importantly, it allows us to jointly assess two important, interlinked parameters in the setting of acute post-surgical pain – both opioid use and pain control. While we could focus on MMEs as the primary endpoint based on the pilot data described above, we believe that MME alone, without knowing the pain control, is difficult to interpret clinically, and thus suboptimal.

References

1. Silverman, D.G., T.Z. O'Connor, and S.J. Brull, Integrated assessment of pain scores and rescue morphine use during studies of analgesic efficacy. Anesth Analg, 1993. 77(1): p. 168-70.
2. Dai, F., et al., Integration of pain score and morphine consumption in analgesic clinical studies. J Pain, 2013. 14(8): p. 767-77 e8.
3. Ahdieh, H., et al., Efficacy of oxymorphone extended release in postsurgical pain: a randomized clinical trial in knee arthroplasty. J Clin Pharmacol, 2004. 44(7): p. 767-76.
4. Gambling, D., et al., A comparison of Depodur, a novel, single-dose extended-release epidural morphine, with standard epidural morphine for pain relief after lower abdominal surgery. Anesth Analg, 2005. 100(4): p. 1065-74.
5. Gilron, I., et al., A placebo-controlled randomized clinical trial of perioperative administration of gabapentin, rofecoxib and their combination for spontaneous and movement-evoked pain after abdominal hysterectomy. Pain, 2005. 113(1-2): p. 191-200.
6. Katz, J., et al., High dose alfentanil pre-empts pain after abdominal hysterectomy. Pain, 1996. 68(1): p. 109-18.

7. Liu, S., et al., Effects of epidural bupivacaine after thoracotomy. Reg Anesth, 1995. 20(4): p. 303-10.
8. Liu, S., et al., Intravenous versus epidural administration of hydromorphone. Effects on analgesia and recovery after radical retropubic prostatectomy. Anesthesiology, 1995. 82(3): p. 682-8.
9. Reinhart, D.J., et al., Postoperative analgesia after peripheral nerve block for podiatric surgery: clinical efficacy and chemical stability of lidocaine alone versus lidocaine plus clonidine. Anesth Analg, 1996. 83(4): p. 760-5.
10. Reinhart, D.J., et al., Postoperative analgesia after peripheral nerve block for podiatric surgery: clinical efficacy and chemical stability of lidocaine alone versus lidocaine plus ketorolac. Reg Anesth Pain Med, 2000. 25(5): p. 506-13.
11. Romundstad, L., et al., Methylprednisolone reduces pain, emesis, and fatigue after breast augmentation surgery: a single-dose, randomized, parallel-group study with methylprednisolone 125 mg, parecoxib 40 mg, and placebo. Anesth Analg, 2006. 102(2): p. 418-25.
12. Therasse, E., et al., Percutaneous biliary drainage: clinical trial of analgesia with interpleural block. Radiology, 1997. 205(3): p. 663-8.
13. Wininger, S.J., et al., A randomized, double-blind, placebo-controlled, multicenter, repeat-dose study of two intravenous acetaminophen dosing regimens for the treatment of pain after abdominal laparoscopic surgery. Clin Ther, 2010. 32(14): p. 2348-69.
14. Cooper, S.A., et al., Research design considerations for single-dose analgesic clinical trials in acute pain: IMMPACT recommendations. Pain, 2016. 157(2): p. 288-301.
15. Werner, M.U., et al., Prediction of postoperative pain: a systematic review of predictive experimental pain studies. Anesthesiology, 2010. 112(6): p. 1494-502.

14.2 Appendix B. Table of Minimum Required Variants

Gene	Allele	Variant	dbSNP
CYP2C19	*2	681G>A	rs4244285
	*3	636G>A	rs4986893
	*4	1A>G	rs28399504
	*6	395G>A	rs72552267
	*8	358T>C	rs41291556
	*17 (also *4 haplotype [*4B])	-806C>T	rs12248560
CYP2D6	*2	2850C>T	rs16947
	*2	4180G>C	rs1135840
	*3	2549delA	rs35742686
	*4	1846G>A	rs3892097
	*5	CYP2D6 deleted	
	*6	1707delT	rs5030655
	*8	1758G>T	rs5030865(A)
	*9	2615_2617delAAG	rs5030656
	*10 (also *36 gene conversion)	100C>T	rs1065852
	*17	1023C>T	rs28371706
	*29 (also *70)	3183G>A	rs59421388
	*41	2988G>A	rs28371725
	1XN	copy number	
	2XN	copy number	
	4XN	copy number	
	9XN	copy number	
	17XN	copy number	
	29XN	copy number	

	41XN	copy number	

14.3 APPENDIX C. CYP2D6 INHIBITORS AS DEFINED BY THE FDA GUIDENCE ON DRUG INTERACTIONS

As of 3/6/20:

Strong Inhibitors	bupropion, fluoxetine, paroxetine, quinidine, terbinafine
Moderate Inhibitors	abiraterone, cinacalcet, duloxetine, lorcaserin, mirabegron

Acute Pain Statistical Analysis Plan

A Depression and Opioid Pragmatic Trial in Pharmacogenetics

(ADOPT PGx)

(For Primary Manuscript)

Study Funding:	National Human Genome Research Institute
Protocol Version:	7.0
Protocol Date:	September 27, 2023
Analysis Plan Version:	1.0
Analysis Plan Date:	February 21, 2024

Revision History

Version Date	File Name	Summary of Changes
1.0	ADOPT PGx_AP_PrimaryMS_SAP_v1.0.docx	Original

Table of Contents

1 Introduction 7

2 Objectives of the Study 7

2.1 Primary 7

2.2 Secondary 7

3 Data Sources 8

4 Analysis Population 8

4.1 Intention-to-treat (ITT) Population 8

4.2 Modified Intention-to-treat (mITT) Population 8

4.3 Concordant Population 8

4.4 The Non-oxycodone Population 9

4.5 Non-actionable Population 9

5 General Analysis Conventions 9

5.1 Population Stratification 9

5.2 Statistical Conventions 9

5.3 Multiplicity 10

5.4 Derivation of Rank-Based Endpoints 10

5.5 CYP2D6 Inhibitor Phenoconversion 10

6 Patient Characteristics 10

6.1 Disposition of Participants 10

6.2 Baseline Characteristics 11

7 Efficacy Evaluation 11

7.1 Primary Endpoint and Analysis 11

7.2 Sensitivity Analyses 12

7.2.1 Covariate Adjusted Analysis 12

7.2.2 Concordant Population 12

7.2.3 Non-oxycodone Population 12

7.2.4 Non-actionable population 13

7.2.5 Alterative SIA Derivations 13

7.3 Secondary Endpoints and Analyses 13
7.3.1 Secondary Endpoints 13
7.4 Exploratory Analyses 17
7.4.1 Interaction Between Prescribed CYP2D6 Opioid Type and Intervention 17
7.4.2 SIA Score at 1 Month 17
7.4.3 Prescribed Opioid Usage at 1 Month 17
7.4.4 Time Trends in Pain Intensity 18
7.4.5 Time Trends in Composite Pain Intensity Scores 18
7.4.6 Differences by Surgery Type 18
7.4.7 Participants' Interaction with PGx Test Results 18
7.5 Handling of Missing Data 18
8 Safety Evaluation 19

List of Abbreviations

Abbreviation	Definition
ADOPT PGx	A Depression and Opioid Pragmatic Trial in Pharmacogenetics
CPIC	Clinical Pharmacogenetics Implementation Consortium
DEA	Drug Enforcement Administration
IGNITE PTN	Implementing Genomics in Practice Pragmatic Trials Network
IM	Intermediate metabolizer
ITT	Intention-to-treat
mITT	Modified intention-to-treat
MME	Milligrams of morphine equivalent
NHGRI	The National Human Genome Research Institute
NM	Normal metabolizer
NPRS	Numeric pain rating scale
PGx	Pharmacogenetics
PM	Poor metabolizer
PROMIS	Patient Reported Outcome Measurement System
RxNorm	Medical prescription normalized system
RxCUI	RxNorm Concept Unique Identifier
SIA	Silverman integrated analgesic
UM	Ultra-rapid metabolizer

1 Introduction

A Depression and Opioid Pragmatic Trial in Pharmacogenetics (ADOPT PGx) is a prospective, multicenter, subset analysis of 1:1 randomized Intervention (immediate PGx testing and genotype-guided opioid or selective serotonin reuptake inhibitor (SSRI) therapy with clinical decision support) vs. Control (usual care with delayed PGx testing) pragmatic, open label clinical trial. The three trials are 1) genotype-guided opioid therapy among participants with post-surgical acute pain (Acute Pain), 2) genotype-guided opioid therapy among participants with chronic pain (Chronic Pain), and 3) genotype-guided SSRI therapy in participants with depression (Depression).

The objective of the Acute Pain trial is to determine if a genotype-guided approach to acute post-surgical pain therapy leads to improved pain control compared to usual care, as defined by a decrease in the Silverman Integrated Analgesic (SIA) score. Secondarily, we will evaluate whether this approach leads to reduced use of Drug Enforcement Administration (DEA) Schedule II opioids and reduced pain intensity. The primary endpoint for the Acute Pain study is the SIA score, a composite of pain and opioid usage, at 10 days post-surgical procedure. Patients with planned/elective surgery who are anticipated to start post-surgery pain control using a study drug of interest (i.e., tramadol, hydrocodone, or codeine) will be enrolled. The total enrollment required for this trial is dependent upon the proportion of the randomized population that have an **actionable phenotype**, defined as CYP2D6 Intermediate Metabolizer (IM) or Poor Metabolizer (PM) genotype (i.e., CYP2D6 activity score ≤ 0.75) or through the use of concomitant medications that are CYP2D6 strong or moderate inhibitors ("phenoconversion"). Assuming that 24%, 21%, or 18% of the population are actionable, 1518, 1732, or 2020 randomized participants will be needed, respectively.

2 Objectives of the Study

The rationale for examining a genotype-guided approach to post-surgical pain management is based on the importance of CYP2D6 for the bioactivation of tramadol, codeine, and hydrocodone and data from a pilot study supporting improved pain control in IM/PMs with CYP2D6-guided therapy.

2.1 Primary

The objective of the ADOPT-PGx Acute Pain study is to determine if a genotype-guided approach to acute post-surgical pain therapy leads to improved pain control compared to usual care, as defined by a decrease in the SIA score.

2.2 Secondary

- Determine the effect of genotype-guided pain therapy on pain intensity at 10 days post-surgery
- Determine the effect of genotype-guided pain therapy on opioid usage at 10 days post-surgery
- Determine the effect of genotype-guided pain therapy on prescription pain medication misuse score at 3-months post-surgery
- Determine the effect of genotype-guided pain therapy on mobility at 1-month post-surgery

- Determine the effect of genotype-guided pain therapy on opioid persistence 6 months post-surgery
- Determine the effect of genotype-guided pain therapy on concordance between PGx phenotype and medication prescribed

3 Data Sources

The primary data source for the ADOPT PGx Depression study will be the participant survey data, the consent, randomization, medications, surgery information, safety events, and site-reported protocol deviations/violations; these will be captured in a REDCap database by trained and qualified personnel at each clinical site for baseline assessments and primarily by the University of Florida Call Center or site personnel for follow-up assessments. Genetic testing results will be imported to the study REDCap database by laboratories or study-staff.

Supporting data for the ADOPT PGx Acute Pain study will include PROMIS survey instrument T-score scoring maps or scoring of the PROMIS survey instruments using the Health Centers scoring service (https://www.assessmentcenter.net/ac_scoringservice), and expert-reviewed list of study-related medications and the associated RxNorm identifiers (RxCUI).

Database will be locked prior to statistical analysis finalization.

4 Analysis Population

4.1 Intention-to-treat (ITT) Population

The intent-to-treat population (ITT) includes all participants who have been randomized to one of the study arms.

4.2 Modified Intention-to-treat (mITT) Population

The modified intent to treat (mITT) population is the subset of the ITT population who completed their surgery, were discharged from their surgery admission, and have an actionable PGx phenotype for medication efficacy. Actionable PGx phenotype is defined as a PGx test result that is a CYP2D6 IM or PM (i.e., CYP2D6 activity score ≤ 0.75), after accounting for CYP2D6 phenoconversion. This population will hence forth be referred to as the "**actionable subgroup**". Participants that have other CYP2D6 phenotypes will not be included in the mITT population. This excludes PGx phenotypes that are actionable for medication safety (*i.e.* CYP2D6 ultrarapid metabolizer or normal – ultrarapid metabolizer). *Unless otherwise specified, the mITT population will be used in the ADOPT PGx Acute Pain primary endpoint, secondary endpoints, and exploratory analyses.*

4.3 Concordant Population

The concordant population will consist of the subset of the **actionable subgroup** whose PGx test results were returned as expected relative to the surgery date and have pain medications concordant with the intervention status. Specifically:

- For the immediate PGx arm, this is limited to participants who had their PGx test result deposited in the medical record prior to the surgery date and did not have a conflict between their PGx metabolizer phenotype and the post-surgery pain medication prescriptions (i.e. a drug gene conflict)
    - The drug-gene combinations that conflict with the intervention are as follows:
        - CYP2D6 IM or PM, after accounting for pheno conversion, prescribed tramadol, hydrocodone, or codeine
- For the delayed PGx arm, this is limited to participants who had their PGx test result returned after the surgery

4.4 The Non-oxycodone Population

The non-oxycodone population is similar to the concordant per protocol population (section 4.3), with the exclusion of immediate PGx arm participants prescribed oxycodone for post-surgery pain management. Specifically:

- For the immediate PGx arm, this is the subset of the mITT population who did not have a conflict between their PGx metabolizer phenotype and the pain medication(s) prescribed for post-surgery pain and were not prescribed oxycodone for post-surgery pain management
- For the delayed PGx arm, this is the subset of the mITT population who had their PGx test result returned after the primary endpoint timepoint

4.5 Non-actionable Population

The **non-actionable population** is defined as the subset of the ITT population who completed their surgery and who have a non-actionable PGx phenotype for medication efficacy. Non-actionable phenotype is defined as a CYP2D6 activity score ≥ 1.0, after accounting for CYP2D6 phenoconversion. Note that this includes PGx phenotypes that are actionable for medication safety.

5 General Analysis Conventions

5.1 Population Stratification

Participants from all study sites will be combined and included in the primary, secondary, and exploratory analyses. Any other population stratification will be described in subsequent sections.

5.2 Statistical Conventions

Unless otherwise specified, descriptive statistics will be presented as mean, standard deviation, median percentiles (25th, 75th), and range as appropriate for the continuous variables, and as frequencies and percentages for categorial variables. Unless otherwise specified, summaries will be reported on non-missing observations and percentages will be based on the non-missing values.

Statistical comparisons by treatment groups will be performed using two-sided significance tests. Additional details for specific analyses are presented in subsequent sections.

For modeling purposes, rare or uncommon levels of categorical variables will be merged into other levels.

5.3 Multiplicity

With the primary and various secondary endpoints and sensitivity analyses outlined below, there is a multiplicity of analyses to be performed, which leads to an increased probability that at least one of the comparisons could be “significant” by chance. Adjusting for the effects of the repeated significance testing for the multiplicity of secondary endpoints would require that very small significance levels be used for every comparison. Therefore, rather than adjusting for multiple comparisons, we will be conservative in the interpretation of the analyses, considering the degree of significance, and looking for consistency across endpoints. The nominal (unadjusted) p-value for each comparison will be reported to aid in the overall interpretation.

5.4 Derivation of Rank-Based Endpoints

Primary and secondary endpoints that are based on rank transformations of quantitative data (e.g. the Silverman Integrative Analgesic [SIA] Score) will be derived separately in the mITT population and the ITT population with completed surgeries. Analyses will be conducted using the mITT population derivation of the rank-based measures unless otherwise noted.

5.5 CYP2D6 Inhibitor Phenoconversion

CYP2D6 phenoconversion due to concomitant CYP2D6 inhibitor medications:

- If a participant reports taking a strong CYP2D6 inhibitor at baseline, the CYP2D6 activity score is multiplied by 0 and the phenotype converts to a poor metabolizer
- If a participant reports taking a moderate CYP2D6 inhibitor at baseline, the CYP2D6 activity score is multiple by 0.5 and the phenotype is assigned based on the converted activity score
- If a participant reports neither a strong nor a moderate CYP2D6 inhibitor at baseline, the CYP2D6 activity score and phenotype is unchanged

6 Patient Characteristics

6.1 Disposition of Participants

Disposition data for all participants (number of participants who were screened, randomized, received PGx testing, withdrew consent, lost to follow-up, died prior the 10-day visits, etc.) will be summarized by randomized treatment groups.   The summary will include:

- Number of participants who were screened, randomized, completed surgery, and/or received PGx testing
- Inclusion in ITT, actionable subgroup (mITT), and concordant populations
- Participants completing 10 day, 1, 3, and/or 6 month visits
- Lost to follow-up
- Withdrew consent for follow-up

- Died prior to visits at 10-days, 1, 3, or 6 months

6.2 Baseline Characteristics

The demographic and baseline characteristics collected in the study will be tabulated and summarized as descriptive statistics for both ITT and actionable subgroup (mITT) populations by randomized treatment group.

The data will be presented and compared by randomized treatment groups. Statistical tests will be performed using Wilcoxon rank-sum test for continuous variables and Chi-square test or Fisher's Exact test (if any of the expected cell count is less than 5) for categorical variables. The corresponding p-values will be presented for the comparison between treatment groups.

7 Efficacy Evaluation

7.1 Primary Endpoint and Analysis

The primary efficacy endpoint is pain control, defined the Silverman Integrated Analgesic (SIA) score, at 10-days post-surgery. The SIA score is a composite measure of pain and opioid usage, calculated as follows: pain and *prescribed* opioid usage are ranked, the ranks are converted to percentiles and linearly transformed such that the scores are centered at 0 and range -100 to 100. The pain scores and opioid usage transformed percentiles are summed to generate the SIA score that ranges -200 to 200. Additional details can be found in Dai, F., et al J. Pain (2023) 14(8):767-77. Negative values indicate low pain with minimal opioid usage, while positive values correspond with higher pain levels and/or higher opioid usage. The SIA score is quantified for participants who completed their surgery, were not prescribed liquid forms of opioids for pain control, and were able to report their medication usage 10-days post-surgery. If either the pain rating or the opioid usage data are incomplete or missing, then the SIA score will be missing.

The primary hypothesis is:

H_0 (null hypothesis): Providing the provider with PGx-guided therapy recommendations has no effect on the SIA scores at 10-days post-surgery.

H_a (alternative hypothesis): Providing the provider with PGx-guided therapy recommendations does affect the SIA score 10-days post-surgery.

Special note:

In the study protocol, a single interim analysis was planned and the final analysis significance level was specified as 0.049 to account for the alpha spending in a single interim analysis. The planned interim analysis was not conducted due to limited utility. Since the interim analysis was not conducted, there

was no associated alpha spending that needs to be accounted for in the primary endpoint significance level. As such, the primary endpoint analysis will be considered significant if the p-value is < 0.05.

Primary analysis:

In the actionable subgroup (mITT) population, the mean SIA score in the immediate PGx arm 10-days post-surgery will be compared to the mean SIA score in the delayed arm at 10-days post-surgery using a two-sided two-sample t-test and will be considered significant if the p-value is < 0.05. The mean difference in SIA (standard error) and p-value will be presented and summary statistics will be provided for the SIA score at 10 days post-surgery for each intervention arm.

7.2 Sensitivity Analyses

7.2.1 Covariate Adjusted Analysis

For the primary endpoint (*i.e.* 10-day post-surgery SIA score), we will conduct covariate-adjusted linear regression to account for differences in baseline characteristics between the immediate PGx arm and the delayed PGx arm. This analysis will be conducted in the actionable subgroup population with complete data.

Scientifically relevant baseline characteristics such as: *age, gender, race, surgery type, and baseline depression score* will be considered as potential covariates. All potential covariates will be assessed for missing data, if the overall proportion (i.e. across all potential covariates) of observations with missing data is >5%, the adjustment variable(s) contributing to this missing data may be excluded, while jointly considering levels of missing data and scientific relevance. Additionally, surgery type and baseline depression score will be assessed for imbalance between treatment arms in the actionable subgroup population and if an imbalance exists at significance level of p-value < 0.05, will be included as potential covariates.

Pairwise correlation will used to identify collinearity among the potential covariates. Covariates with < 0.6 correlation coefficient will be included in the model. Additionally, if the model fails to converge or encounters difficulties, we will attempt to simplify the model to eliminate the modeling problems.

The treatment effect will be estimated as the difference in the least squares mean (lsmean) estimates for the by treatment mean SIA scores. The lsmean estimates, standard error, and the difference in lsmeans, standard error, 95% confidence interval, and p-value will be reported.

7.2.2 Concordant Population

A sensitivity analysis for the primary endpoint will be applied to the concordant population defined in section 4.3.

7.2.3 Non-oxycodone Population

A sensitivity analysis for the primary endpoint will be applied to the subset of the concordant population not prescribed oxycodone defined in section 4.4

7.2.4 Non-actionable population

A sensitivity analysis for the primary endpoint will be applied to the non-actionable population, defined in section 4.5, using the SIA score derived in the ITT population with completed surgeries.

7.2.5 Alterative SIA Derivations

A sensitivity analysis using an alternative SIA score derivation will be conducted in the actionable phenotype subgroup.

7.2.5.1 SIA Using Alternative Pain Measure

The SIA score will be calculated using *prescribed* opioid usage and the composite pain intensity score, instead of the numeric pain rating scale.

Analysis: By-treatment differences in this alternative SIA score calculation at 10 days post-surgery within the actionable subgroup will be assessed by comparing alternative SIA score using a two-sided two-sample t-test. The mean difference in the alternative SIA score (standard error) and p-value will be reported.

Using the covariates established in section 7.2.1, multivariate linear regression will be used to determine the treatment differences in the alternative SIA score calculation after adjusting for baseline characteristics. The by treatment lsmeans estimate, standard error, and the difference in treatment ls means estimate, standard error, 95% confidence interval, and p-value will be reported.

7.3 Secondary Endpoints and Analyses

7.3.1 Secondary Endpoints

The secondary endpoints for the ADOPT-PGx Acute Pain trial are as follows:

- Opioid usage at 10 days post-surgery
- Pain intensity at 10 days post-surgery
- Composite pain intensity score at 10 days post-surgery
- Prescription pain medication misuse score at 3-months post-surgery
- Mobility at 1-month post-surgery
- Opioid persistence 6 months post-surgery
- Concordance between PGx phenotype and prescribed pain medication

7.3.1.1 Opioid Usage

7.3.1.1.1 Prescribed Opioid Usage

Definition: Opioid usage is defined as the cumulative morphine milligram equivalents (MMEs) reported among the *prescribed* opioids between surgery discharge and the 10-day survey dates and adjusted for the number of days between discharge and the 10-day survey date, i.e. average daily MME. Usage of each prescribed opioid are derived from the recorded number of pills used and the pill strength, yielding total mg consumed. Each opioid total mg consumed is converted to an MME and the per opioid MME

are summed across all prescribed opioids and adjusted for the number of days between discharge and the 10-day survey date.

The hypothesis is:

H_0 (null hypothesis): Providing the provider with PGx-guided therapy recommendations has no effect on opioid usage at 10 days post-surgery.

H_a (alternative hypothesis): Providing the provider with PGx-guided therapy recommendations does affect the change on opioid usage at 10 days post-surgery.

Analysis: By-treatment differences in opioid usage at 10 days post-surgery within the actionable subgroup will be assessed using a Wilcoxon rank sum test. The mean difference in the alternative SIA score (standard error) and p-value will be reported.

Using the covariates established in section 7.2.1, multivariate linear regression will be used to determine the treatment differences in opioid usage after adjusting for baseline characteristics. The by treatment lsmeans estimate, standard error, and the difference in treatment ls means estimate, standard error, 95% confidence interval, and p-value will be reported.

7.3.1.2 Pain Intensity

Definition: The participant response to the numeric pain rating scale, a numeric scale ranging from 0 (no pain) to 10 (worst possible pain) for average pain, at 10-days post-surgery.

The hypothesis is:

H_0 (null hypothesis): Providing the provider with PGx-guided therapy recommendations has no effect on pain intensity at 10 days post-surgery.

H_a (alternative hypothesis): Providing the provider with PGx-guided therapy recommendations does affect the pain intensity at 10 days post-surgery.

Analysis: By-treatment differences in pain intensity at 10 days post-surgery within the actionable subgroup will be assessed by comparing mean pain intensity using a two-sided two-sample t-test. The mean difference in pain intensity (standard error) and p-value will be reported.

Using the covariates established in section 7.2.1, multivariate linear regression will be used to determine the treatment differences in pain intensity after adjusting for baseline characteristics. The by treatment lsmeans estimate, standard error, and the difference in treatment ls means estimate, standard error, 95% confidence interval, and p-value will be reported.

7.3.1.3 Composite Pain Intensity Score

Definition: The composite pain intensity score is the sum of the average and worst pain in the past 7 days and current pain, each rated on a 5-point scale.

The hypothesis is:

H_0 (null hypothesis): Providing the provider with PGx-guided therapy recommendations has no effect on pain intensity at 10 days post-surgery.

H_a (alternative hypothesis): Providing the provider with PGx-guided therapy recommendations does affect the pain intensity at 10 days post-surgery.

Analysis: By-treatment differences in composite pain intensity score within the actionable subgroup will be assessed by comparing mean composite pain intensity score using a two-sided two-sample t-test. The mean difference in pain intensity (standard error) and p-value will be reported.

Using the covariates established in section 7.2.1, multivariate linear regression will be used to determine the treatment differences in the composite pain intensity score, after adjusting for baseline characteristics. The by treatment lsmeans estimate, standard error, and the difference in treatment lsmeans estimate, standard error, 95% confidence interval, and p-value will be reported.

7.3.1.4 Concordance between PGx Phenotype and Prescribed Pain Medications

Definition: The absence of a conflict between the CYP2D6 phenotype and CYP2D6 opioid medication prescription recorded for post-surgery pain management. The list of incompatible CYP2D6 phenotypes and mediations are as follow:

- CYP2D6 IM or PM, after accounting for phenoconversion, prescribed tramadol, hydrocodone, or codeine
- CYP2D6 UM or NM-UM, after accounting for phenoconversion, prescribed tramadol, hydrocodone, codeine, or oxycodone

The hypothesis is:

H_0 (null hypothesis): Providing the provider with PGx-guided therapy recommendations has no effect on the concordance between CYP2D6 phenotypes and prescribed pain medications prescribed for post-surgery pain management.

H_a (alternative hypothesis): Providing the provider with PGx-guided therapy recommendations does affect the concordance between CYP2D6 phenotypes and prescribed pain medications prescribed for post-surgery pain management.

Analysis: By treatment differences in the proportion of concordant participants in the actionable subgroup will be assessed using a test of two-proportions.

Using the covariates established in section 7.2.1, multivariate logistic regression will be used to determine the treatment differences in the proportion of concordant participants after adjusting for baseline characteristic. The treatment effect parameter (B_1), standard error, odds ratio, 95% confidence interval of the odds ratio, and p-value will be reported.

7.3.1.5 Prescription Pain Mediation Misuse

Definition: The participant responses to the *PROMIS prescription medication mis-use scale* will be scored per the published scoring guidelines. The scores reported at 3-months post-surgery will be evaluated.

The hypothesis is:

H_0 (null hypothesis): Providing the provider with PGx-guided therapy recommendations has no effect on prescription pain medication misuse scores at 3-months post-surgery.

H_a (alternative hypothesis): Providing the provider with PGx-guided therapy recommendations does affect the pain medication misuse scores at 3-months post-surgery.

Analysis: By-treatment differences in prescription pain medication misuse T-score within the actionable subgroup will be assessed by comparing mean T-scores using a two-sided two-sample t-Test. The mean difference in prescription pain medication misuse T-score reduction (standard error) and p-value will be reported.

7.3.1.6 Mobility

Definition: Summed responses to the PROMIS item Bank v2.0 mobility questions at 1-month post-surgery (adults-only).

The hypothesis is:

H_0 (null hypothesis): Providing the provider with PGx-guided therapy recommendations has no effect on mobility scores at 1-month post-surgery.

H_a (alternative hypothesis): Providing the provider with PGx-guided therapy recommendations does affect the mobility score at 1-month post-surgery.

Analysis: The by treatment differences in the 1-month adult mobility scores within the adult participants in the actionable subgroup will be assessed by comparing mean T-scores using a two-sided two-sample t-test. The mean difference in mobility score (standard error) and p-value will be reported.

7.3.1.7 Opioid Persistence

Definition: Having an opioid prescription filled 90-180 days post-surgery, among the participants who reported one or more filled opioid prescriptions at 10-days.

The hypothesis is:

H_0 (null hypothesis): Providing the provider with PGx-guided therapy recommendations has no effect on opioid persistence at 90-180 days post-surgery.

H_a (alternative hypothesis): Providing the provider with PGx-guided therapy recommendations does affect the change on opioid persistence at 90-180 days post-surgery.

Analysis: By treatment differences in the proportion of participants with opioid persistence in the actionable subgroup will be assessed using a test of two proportions. Difference in proportion and the 95% confidence interval will be reported.

7.4 Exploratory Analyses

For analyses using covariate-adjusted models, potential baseline covariates included will be the same as those used for the covariate-adjusted primary endpoint analysis.

For all analyses using mixed model for repeated measures, appropriate covariance matrix (*e.g.*, compound symmetry, autoregressive, variance components, or other covariance structure) will be selected based on the data to account for the correlated endpoints from individual participants. We will select the covariance structure that has the smallest Akaike's Information Criteria (AICC) value. When two or more covariance structures yield a similarly small AICC value, the most parsimonious model with the fewest number of parameters (*i.e.*, distinct covariance values) will be selected.

7.4.1 Interaction Between Prescribed CYP2D6 Opioid Type and Intervention

In the actionable subgroup participants randomized to the immediate PGx arm, we will assess if the treatment effect on SIA scores differs between participants who were and were not prescribed oxycodone for post-surgery pain management.

H_0 (the null hypothesis): The treatment effect on pain control is the same for participants not prescribed oxycodone, compared to participants prescribed oxycodone.

H_a (the alternative hypothesis): The treatment effect on pain control is different for participants not prescribed oxycodone, compared to participants prescribed oxycodone.

This analysis will involve testing for the interaction between intervention arm and oxycodone prescriptions using a covariate-adjusted linear regression model. If evidence supports an interaction, we will test the intervention effect on the 10-day post-surgery SIA scores in the subset of participants who were prescribed oxycodone and those who were not prescribed oxycodone.

7.4.2 SIA Score at 1 Month

We will compare the SIA scores at 1 month between the two randomized treatment groups (*i.e.* Immediate PGx testing with genotype guided therapy and delayed PGx testing), using the same t-test described previously.

7.4.3 Prescribed Opioid Usage at 1 Month

We will compare the opioid usage at 1 month between the two randomized treatment groups (*i.e.* Immediate PGx testing with genotype guided therapy and delayed PGx testing), using the same t-test described previously.

7.4.4 Time Trends in Pain Intensity

We will compare the time trends in the pain intensity at 10 days, 1, 3, and 6 month between the two randomized treatment groups (*i.e.* Immediate PGx testing with genotype guided therapy and delayed PGx testing), using a repeated-measures mixed effect model with a baseline-value and baseline-covariates adjustments.

7.4.5 Time Trends in Composite Pain Intensity Scores

We will compare the time trends in in the composite pain intensity scores at 10 days, 1, 3, and 6 months between the two randomized treatment groups (*i.e.* Immediate PGx testing with genotype guided therapy and delayed PGx testing), using a repeated-measures mixed effect model with a baseline-value and baseline-covariates adjustments.

7.4.6 Differences by Surgery Type

Variability in the treatment effect on the 10-day SIA score by surgery type, total knee vs total hip replacement, will be assessed through linear regression, including a treatment by surgery type interaction term, in the subset of participants who had a total knee or total hip replacement. The interaction term p-value and the surgery type stratified treatment effects (standard error) will be reported.

7.4.7 Participants' Interaction with PGx Test Results

Among participants randomized to the immediate PGx arm, we will assess if participant's interaction with their PGx test results impacts the SIA score at 10-days, using the SIA score derived in the ITT population with completed surgeries. Participants who indicated that they interacted with their PGx by looking up their result in their medical record, requested a copy of the result, or discussed with their provider will be referred to as "unmasked" while participants who indicated that they did not interact with their PGx test result will be referred to as "masked". We will compare the SIA score at 10-days in the masked and unmasked groups, using a bivariate t-test and multivariable modeling.

7.5 Handling of Missing Data

If the SIA score is missing in 10% or more of the observations in the actionable subgroup population, sensitivity analysis will be performed using data obtained from multiple imputation. The sensitivity analysis will be performed for the primary analysis and the covariate-adjusted analyses for the primary endpoint. Multiple imputation will be used to impute all missing pain intensity scores and opioids usage values except for those due to participant death or due to study-discontinuation due to not having the surgery completed at the participating healthcare institution as expected.

Under the missing at random (MAR) assumption, subjects who discontinue early **after their surgery** and are alive are assumed to have the same data pattern as subjects who remain in the study in the same treatment arm. Multiple imputation will be carried out to impute missing data for at baseline and 10-days.

Step 1 - First, the intermittent pain intensity and opioid usage will be imputed for each treatment relying on non-missing data from all subjects within each treatment group using the Markov Chain Monte Carlo (MCMC) imputation model with baseline pain intensity, covariates (as identified in section 7.2.1), and the available non-missing opioid usage and pain intensity data to generate a monotone missing data pattern. Then, missing data (i.e. 10-day opioid usage and 10-day pain intensity) will be imputed to derive 20 imputed datasets with non-missing data. It is anticipated that 20 imputations will be sufficient to achieve stable parameter estimates. 20 imputations are expected to be sufficient to achieve <5% change in the standard error estimates when the fraction of missing information is < 31%.

Step 2 - The 20 multiple-imputation datasets with imputed and observed pain intensity and opioid usage at 10-days will be used to derive the SIA score endpoint. Each imputation dataset will be analyzed separately, the results of which will be averaged to obtain the final treatment effect estimates.

The imputed values will be used for sensitivity analyses only and will not be included in the primary analysis.

8 Safety Evaluation

ADOPT-PGx is a study regulated under an Abbreviated Investigational Device Exemption (IDE). The genotyping-guided therapy is the device and classified as a minimal risk to the welfare of the enrolled participants. The following safety data will be presented by randomized treatment for safety evaluation:

- Early termination and discontinuation
- Self-reported emergency department visits and hospitalizations
- Adverse effects occurring during treatment with pain medications
- Unanticipated study-related deaths
- Reportable adverse device effects or unanticipated adverse device effects

Safety evaluation will be performed among all participants in the actionable subgroup (mITT) and ITT populations.

Acute Pain Statistical Analysis Plan

A Depression and Opioid Pragmatic Trial in Pharmacogenetics

(ADOPT PGx)

(For Primary Manuscript)

Study Funding:	National Human Genome Research Institute
Protocol Version:	8.0
Protocol Date:	May 23, 2024
Analysis Plan Version:	2.0
Analysis Plan Date:	July 11, 2024

Revision History

Version Date	File Name	Summary of Changes
February 21, 2024	ADOPT PGx_AP_PrimaryMS_SAP_v1.0.docx	Original
July 11, 2024	ADOPT PGx_AP_PrimaryMS_SAP_v2.0.docx	• Minor grammatical corrections, wording adjustments, and updates to section references across the SAP<br>• Clarification of factors that define the mITT population (section 4.2)<br>• Revised subgroup label from Concordant Population to Per-Protocol Subgroup (section 4.3)<br>• Revised population subgroup label from the Non-oxycodone Concordant Population to Non-oxycodone Per-Protocol Subgroup (section 4.4)<br>• Inclusion of surgery characteristics in the Baseline Characteristics (section 6.2)<br>• Addition of Post-Baseline characteristics summary (section 6.3)<br>• Addition of PGx guided therapy implementation characteristics data descriptions (section 6.4)<br>• Renamed sensitivity analysis section (formerly section 7.2) to Exploratory Analyses of the Primary Endpoint (Section 7.4)<br>• Moved the alternative SIA score analysis to section 7.4.1 (formerly section 7.5.2.1)<br>• Update description the Per-Protocol Subgroup of Special Interest Analysis (formerly the Concordant Population, Section 7.4.2), modified the statistical approach from two-sample t-test to a linear regression with and without baseline-covariate adjustments<br>• Update description the Non-oxycodone Per-protocol Subgroup of Special Interest Analysis (formerly the Non-oxycodone Concordant Population, Section 7.4.3), added the scientific rational for this subgroup of special interest, modified the statistical approach from two-sample t-test to a linear regression with and without baseline-covariate adjustments<br>• Consolidated unadjusted and adjusted analyses of other subgroup analyses of the primary endpoint, update methods to linear regression modeling with interaction terms (Sections 7.4.4, 7.4.5, and 7.4.6)

		• Updated statistical approach to include both unadjusted and baseline-covariate analyses for exploratory analysis sections 7.5.1, 7.5.2, 7.5.3, and 7.5.4.<br>• Addition of assessments for systematic disruptions in missing primary endpoint data or PGx results data (section 7.5.5)<br>• Clarification that medication side effects are only captured for participants reporting taking a prescription for pain medication (section 8)

Table of Contents

1 *Introduction* 8

2 *Objectives of the Study* 8

2.1 Primary 8

2.2 Secondary 8

3 *Data Sources* 9

4 *Analysis Population* 9

4.1 Intention-to-treat (ITT) Population 9

4.2 Modified Intention-to-treat (mITT) Population 9

4.3 Per-Protocol Subgroup 10

4.4 The Non-oxycodone Per-Protocol Subgroup 10

4.5 Non-actionable Population 10

5 *General Analysis Conventions* 10

5.1 Population Stratification 10

5.2 Statistical Conventions 11

5.3 Multiplicity 11

5.4 Derivation of Rank-Based Endpoints 11

5.5 CYP2D6 Inhibitor Phenoconversion 11

6 *Patient and Implementation Characteristics* 12

6.1 Disposition of Participants 12

6.2 Baseline Characteristics 12

6.3 Post-baseline Characteristics 12

6.4 Implementation Characteristics 12

7 *Efficacy Evaluation* 13

7.1 Primary Endpoint and Analysis 13

7.2 Covariate Adjusted Analysis 14

7.3 Secondary Endpoints and Analyses 14

7.3.1 Secondary Endpoints 14

7.4 Exploratory Analyses of the Primary Endpoint 18
7.4.1 SIA Using Alternative Pain Measure 18
7.4.2 Per-Protocol Subgroup of Special Interest 18
7.4.3 Non-oxycodone Per-Protocol Subgroup of Special Interest 19
7.4.4 Non-actionable Complementary Subgroup 19
7.4.5 Differences by Surgery Type 20
7.4.6 Participants' Interaction with PGx Test Results 20

7.5 Exploratory Analyses 21
7.5.1 SIA Score at 1 Month 21
7.5.2 Prescribed Opioid Usage at 1 Month 21
7.5.3 Time Trends in Pain Intensity 21
7.5.4 Time Trends in Composite Pain Intensity Scores 21
7.5.5 Potential Post-Hoc Analyses 22

7.6 Handling of Missing Data 22

8 Safety Evaluation 23

List of Abbreviations

Abbreviation	Definition
ADOPT PGx	A Depression and Opioid Pragmatic Trial in Pharmacogenetics
CPIC	Clinical Pharmacogenetics Implementation Consortium
DEA	Drug Enforcement Administration
IGNITE PTN	Implementing Genomics in Practice Pragmatic Trials Network
IM	Intermediate metabolizer
ITT	Intention-to-treat
mITT	Modified intention-to-treat
MME	Milligrams of morphine equivalent
NHGRI	The National Human Genome Research Institute
NM	Normal metabolizer
NPRS	Numeric pain rating scale
PGx	Pharmacogenetics
PM	Poor metabolizer
PROMIS	Patient Reported Outcome Measurement System
RxNorm	Medical prescription normalized system
RxCUI	RxNorm Concept Unique Identifier
SIA	Silverman integrated analgesic
UM	Ultra-rapid metabolizer

1 Introduction

A Depression and Opioid Pragmatic Trial in Pharmacogenetics (ADOPT PGx) is a prospective, multicenter, subset analysis of 1:1 randomized Intervention (immediate PGx testing and genotype-guided opioid or selective serotonin reuptake inhibitor (SSRI) therapy with clinical decision support) vs. Control (usual care with delayed PGx testing) pragmatic, open label clinical trial. The three trials are 1) genotype-guided opioid therapy among participants with post-surgical acute pain (Acute Pain), 2) genotype-guided opioid therapy among participants with chronic pain (Chronic Pain), and 3) genotype-guided SSRI therapy in participants with depression (Depression).

The objective of the Acute Pain trial is to determine if a genotype-guided approach to acute post-surgical pain therapy leads to improved pain control compared to usual care, as defined by a decrease in the Silverman Integrated Analgesic (SIA) score. Secondarily, we will evaluate whether this approach leads to reduced use of Drug Enforcement Administration (DEA) Schedule II opioids and reduced pain intensity. The primary endpoint for the Acute Pain study is the SIA score, a composite of pain and opioid usage, at 10 days post-surgical procedure. Patients with planned/elective surgery who are anticipated to start post-surgery pain control using a study drug of interest (i.e., tramadol, hydrocodone, or codeine) will be enrolled. The total enrollment required for this trial is dependent upon the proportion of the randomized population that have an **actionable phenotype**, defined as CYP2D6 Intermediate Metabolizer (IM) or Poor Metabolizer (PM) genotype (i.e., CYP2D6 activity score ≤ 0.75) or by concomitant medications that are CYP2D6 strong or moderate inhibitors ("phenoconversion"). Assuming that 24%, 21%, or 18% of the population are actionable, 1518, 1732, or 2020 randomized participants will be needed, respectively.

2 Objectives of the Study

The rationale for examining a genotype-guided approach to post-surgical pain management is based on the importance of CYP2D6 for the bioactivation of tramadol, codeine, and hydrocodone and data from a pilot study supporting improved pain control in IM/PMs with CYP2D6-guided therapy.

2.1 Primary

The objective of the ADOPT-PGx Acute Pain study is to determine if a genotype-guided approach to acute post-surgical pain therapy leads to improved pain control compared to usual care, as defined by a decrease in the SIA score.

2.2 Secondary

- Determine the effect of genotype-guided pain therapy on pain intensity at 10 days post-surgery
- Determine the effect of genotype-guided pain therapy on opioid usage at 10 days post-surgery
- Determine the effect of genotype-guided pain therapy on prescription pain medication misuse score at 3-months post-surgery
- Determine the effect of genotype-guided pain therapy on mobility at 1-month post-surgery

- Determine the effect of genotype-guided pain therapy on opioid persistence 6 months post-surgery
- Determine the effect of genotype-guided pain therapy on concordance between PGx phenotype and medication prescribed

3 Data Sources

The primary data source for the ADOPT PGx Depression study will be the participant survey data, the consent, randomization, medications, surgery information, safety events, and site-reported protocol deviations/violations; these will be captured in a REDCap database by trained and qualified personnel at each clinical site for baseline assessments and primarily by the University of Florida Call Center or site personnel for follow-up assessments. Genetic testing results will be imported to the study REDCap database by laboratories or study-staff.

Supporting data for the ADOPT PGx Acute Pain study will include PROMIS survey instrument T-score scoring maps or scoring of the PROMIS survey instruments using the Health Measures scoring services, and expert-reviewed list of study-related medications and the associated RxNorm identifiers (RxCUI).

Database will be locked prior to statistical analysis finalization.

4 Analysis Population

4.1 Intention-to-treat (ITT) Population

The intent-to-treat population (ITT) includes all participants who have been randomized to one of the study arms.

4.2 Modified Intention-to-treat (mITT) Population

The modified intent to treat (mITT) population is the subset of the ITT population who completed their surgery at the participating healthcare institution, have non-missing CYP2D6 PGx test results, and have an actionable PGx phenotype for medication efficacy.

Actionable PGx phenotype is defined as a PGx test result that is a CYP2D6 IM or PM (i.e., CYP2D6 activity score ≤ 0.75), after accounting for CYP2D6 phenoconversion due to the presence of CYP2D6 inhibitors in the baseline medications. This population will hence forth be referred to as the "**actionable subgroup**". Participants that have other CYP2D6 phenotypes (*i.e*. CYP2D6 activity score > 0.75), including phenotypes actionable for medication safety (*i.e.* CYP2D6 ultrarapid metabolizer or normal – ultrarapid metabolizer), or did not complete their surgeries at the participating healthcare institution will not be included in the mITT population. *Unless otherwise specified, the mITT population will be used in the ADOPT PGx Acute Pain primary endpoint, secondary endpoints, and exploratory analyses.*

4.3 Per-Protocol Subgroup

The per-protocol subgroup will consist of the subset of the mITT population (actionable subgroup) described in Section 4.2 whose PGx test results were returned as expected relative to the surgery date and have pain medications concordant with the PGx recommendations. Specifically:

- For the immediate PGx arm, this is limited to participants who had their PGx test result deposited in the medical record prior to the surgery date and did not have a conflict between their PGx metabolizer phenotype and the post-surgery pain medication prescriptions (i.e. a drug gene conflict)
  - The drug-gene combinations that conflict with the intervention are as follows:
    - CYP2D6 IM or PM, after accounting for pheno conversion, prescribed tramadol, hydrocodone, or codeine
- For the delayed PGx arm, this is limited to participants who had their PGx test result returned after the surgery

4.4 The Non-oxycodone Per-Protocol Subgroup

The non-oxycodone population is similar to the per-protocol subgroup of the mITT described in section 4.3, with the exclusion of immediate PGx arm participants prescribed oxycodone for post-surgery pain management. Specifically:

- For the immediate PGx arm, this is the subset of the mITT population who had their PGx test result returned prior to surgery, did not have a conflict between their PGx metabolizer phenotype, and the pain medication(s) prescribed for post-surgery pain and were not prescribed oxycodone for post-surgery pain management
- For the delayed PGx arm, this is the subset of the mITT population who had their PGx test result returned after surgery

4.5 Non-actionable Population

The **non-actionable population** is defined as the subset of the ITT population who completed their surgery and who have a non-actionable PGx phenotype for medication efficacy. Non-actionable phenotype is defined as a CYP2D6 activity score ≥ 1.0, after accounting for CYP2D6 phenoconversion. Note that this includes PGx phenotypes that are actionable for medication safety.

5 General Analysis Conventions

5.1 Population Stratification

Participants from all study sites will be combined and included in the primary, secondary, and exploratory analyses. Any other population stratification will be described in subsequent sections.

5.2 Statistical Conventions

Unless otherwise specified, descriptive statistics will be presented as mean, standard deviation, median percentiles (25th, 75th), and range as appropriate for the continuous variables, and as frequencies and percentages for categorial variables. Unless otherwise specified, summaries will be reported on non-missing observations and percentages will be based on the non-missing values.

Statistical comparisons by treatment groups will be performed using two-sided significance tests. Additional details for specific analyses are presented in subsequent sections.

For modeling purposes, rare or uncommon levels of categorical variables will be merged into other levels.

5.3 Multiplicity

With the primary and various secondary endpoints and sensitivity analyses outlined below, there is a multiplicity of analyses to be performed, which leads to an increased probability that at least one of the comparisons could be "significant" by chance. Adjusting for the effects of the repeated significance testing for the multiplicity of secondary endpoints would require that very small significance levels be used for every comparison. Therefore, rather than adjusting for multiple comparisons, we will be conservative in the interpretation of the analyses, considering the degree of significance, and looking for consistency across endpoints. The nominal (unadjusted) p-value for each comparison will be reported to aid in the overall interpretation.

5.4 Derivation of Rank-Based Endpoints

Primary and secondary endpoints that are based on rank transformations of quantitative data (e.g. the Silverman Integrative Analgesic [SIA] Score) will be derived separately in the mITT population and the ITT population with completed surgeries. Analyses will be conducted using the mITT population derivation of the rank-based measures unless otherwise noted.

5.5 CYP2D6 Inhibitor Phenoconversion

CYP2D6 phenoconversion due to concomitant CYP2D6 inhibitor medications:

- If a participant reports taking a strong CYP2D6 inhibitor at baseline, the CYP2D6 activity score is multiplied by 0 and the phenotype converts to a poor metabolizer
- If a participant reports taking a moderate CYP2D6 inhibitor at baseline, the CYP2D6 activity score is multiple by 0.5 and the phenotype is assigned based on the converted activity score
- If a participant reports neither a strong nor a moderate CYP2D6 inhibitor at baseline, the CYP2D6 activity score and phenotype is unchanged

6 Patient and Implementation Characteristics

6.1 Disposition of Participants

Disposition data for all participants (number of participants who were screened, randomized, received PGx testing, withdrew consent, lost to follow-up, died prior the 10-day visits, etc.) will be summarized by randomized treatment groups. The summary will include:

- Number of participants who were screened, randomized, completed surgery, and/or received PGx testing
- Inclusion in ITT, actionable subgroup (mITT), and concordant populations
- Participants completing 10 days, 1, 3, and/or 6-month visits
- Lost to follow-up
- Withdrew consent for follow-up
- Died prior to visits at 10-days, 1, 3, or 6 months

6.2 Baseline Characteristics

The demographic and baseline characteristics, including surgery characteristics, collected in the study will be tabulated and summarized as descriptive statistics for both ITT and actionable subgroup (mITT) populations by randomized treatment group.

The data will be presented and compared by randomized treatment groups. Statistical tests will be performed using Wilcoxon rank-sum test for continuous variables and Chi-square test or Fisher's Exact test (if any of the expected cell count is less than 5) for categorical variables. The corresponding p-values will be presented for the comparison between treatment groups.

6.3 Post-baseline Characteristics

The participant medications prescribed for post-surgery pain management, and medications reported at 10-days will be tabulated and summarized as descriptive statistics for the actionable subgroup (mITT) population by randomized treatment group.

The data will be presented and compared by randomized treatment groups. Statistical tests will be performed using Wilcoxon rank-sum test for continuous variables and Chi-square test or Fisher's Exact test (if any of the expected cell count is less than 5) for categorical variables. The corresponding p-values will be presented for the comparison between treatment groups.

6.4 Implementation Characteristics

Data associated with the implementation of PGx testing will be summarized using descriptive statistics for the ITT population and by randomized treatment group, where appropriate. The summary will include:

- Number of participants with a PGx test result reported in the medical record
- Number of PGx test results returned appropriately relative to the surgery date

  - i.e. before surgery for participants randomized to immediate PGx guided therapy and after surgery for participants randomized to delayed PGx testing
- Number participants with concordance between the PGx guided therapy recommendations and the pain medications prescribed for post-surgery pain management
- Number of participants who had changes in CYP2D6 inhibitor medications between baseline and 10-days
  - Number of participants without a CYP2D6 inhibitor medication at baseline and with one reported at 10-days
  - Number of participants who reported a baseline CYP2D6 inhibitor medication that was discontinued at 10-days

7 Efficacy Evaluation

7.1 Primary Endpoint and Analysis

The primary efficacy endpoint is pain control, defined the Silverman Integrated Analgesic (SIA) score, at 10-days post-surgery. The SIA score is a composite measure of pain and opioid usage, calculated as follows: pain and *prescribed* opioid usage are ranked, the ranks are converted to percentiles and linearly transformed such that the scores are centered at 0 and range -100 to 100. The pain scores and opioid usage transformed percentiles are summed to generate the SIA score that ranges -200 to 200. Additional details can be found in Dai, F., et al J. Pain (2023) 14(8):767-77. Negative values indicate low pain with minimal opioid usage, while positive values correspond with higher pain levels and/or higher opioid usage. The SIA score is quantified for participants who completed their surgery, were not prescribed liquid forms of opioids for pain control, were discharged prior to the 10-day survey, and were able to report their medication usage 10-days post-surgery. If either the pain rating or the opioid usage data are incomplete or missing, then the SIA score will be missing.

The primary hypothesis is:

H_0 (null hypothesis): Providing the provider with PGx-guided therapy recommendations has no effect on the SIA scores at 10-days post-surgery.

H_a (alternative hypothesis): Providing the provider with PGx-guided therapy recommendations does affect the SIA score 10-days post-surgery.

Special note:

In the study protocol, a single interim analysis was planned, and the final analysis significance level was specified as 0.049 to account for the alpha spending in a single interim analysis. The planned interim analysis was not conducted due to limited utility. Since the interim analysis was not conducted, there was no associated alpha spending that needs to be accounted for in the primary endpoint significance level. As such, the primary endpoint analysis will be considered significant if the p-value is < 0.05.

Primary analysis:

In the actionable subgroup (mITT) population, the mean SIA score in the immediate PGx arm 10-days post-surgery will be compared to the mean SIA score in the delayed arm at 10-days post-surgery using a two-sided two-sample t-test and will be considered significant if the p-value is < 0.05. The mean difference in SIA (standard error) and p-value will be presented and summary statistics will be provided for the SIA score at 10 days post-surgery for each intervention arm.

7.2 Covariate Adjusted Analysis

For the primary endpoint (*i.e.* 10-day post-surgery SIA score), we will conduct covariate-adjusted linear regression to account for differences in baseline characteristics between the immediate PGx arm and the delayed PGx arm. This analysis will be conducted in the actionable subgroup population with complete data.

Scientifically relevant baseline characteristics such as: *age, gender, race, surgery type, and baseline depression score* will be considered as potential covariates. All potential covariates will be assessed for missing data, if the overall proportion (i.e. across all potential covariates) of observations with missing data is >5%, the adjustment variable(s) contributing to this missing data may be excluded, while jointly considering levels of missing data and scientific relevance. Additionally, surgery type and baseline depression score will be assessed for imbalance between treatment arms in the actionable subgroup population and if an imbalance exists at significance level of p-value < 0.05, will be included as potential covariates.

Pairwise correlation will used to identify collinearity among the potential covariates. Covariates with < 0.6 correlation coefficient will be included in the model. Additionally, if the model fails to converge or encounters difficulties, we will attempt to simplify the model to eliminate the modeling problems.

The treatment effect will be estimated as the difference in the least squares mean (lsmean) estimates for the by treatment mean SIA scores. The lsmean estimates, standard error, and the difference in lsmeans, standard error, 95% confidence interval, and p-value will be reported.

7.3 Secondary Endpoints and Analyses

7.3.1 Secondary Endpoints

The secondary endpoints for the ADOPT-PGx Acute Pain trial are as follows:

- Opioid usage at 10 days post-surgery
- Pain intensity at 10 days post-surgery
- Composite pain intensity score at 10 days post-surgery
- Prescription pain medication misuse score at 3-months post-surgery
- Mobility at 1-month post-surgery
- Opioid persistence 6 months post-surgery

- Concordance between PGx phenotype and prescribed pain medication

7.3.1.1 Opioid Usage

7.3.1.1.1 Prescribed Opioid Usage

Definition: Opioid usage is defined as the cumulative morphine milligram equivalents (MMEs) reported among the *prescribed* opioids between surgery discharge and the 10-day survey dates and adjusted for the number of days between discharge and the 10-day survey date, i.e. average daily MME. Usage of each prescribed opioid are derived from the recorded number of pills used and the pill strength, yielding total mg consumed. Each opioid total mg consumed is converted to an MME and the per opioid MME are summed across all prescribed opioids and adjusted for the number of days between discharge and the 10-day survey date.

The hypothesis is:

H_0 (null hypothesis): Providing the provider with PGx-guided therapy recommendations has no effect on opioid usage at 10 days post-surgery.

H_a (alternative hypothesis): Providing the provider with PGx-guided therapy recommendations does affect the change on opioid usage at 10 days post-surgery.

Analysis: By-treatment differences in opioid usage at 10 days post-surgery within the actionable subgroup will be assessed using a Wilcoxon rank sum test. The mean difference in the opioid usage (standard error) and p-value will be reported.

Using the covariates established in section 7.2, multivariate linear regression will be used to determine the treatment differences in the ranked opioid usage after adjusting for baseline characteristics. The by treatment lsmeans estimate, standard error, and the difference in treatment lsmeans estimate, standard error, 95% confidence interval, and p-value will be reported.

7.3.1.2 Pain Intensity

Definition: The participant response to the numeric pain rating scale, a numeric scale ranging from 0 (no pain) to 10 (worst possible pain) for average pain, at 10-days post-surgery.

The hypothesis is:

H_0 (null hypothesis): Providing the provider with PGx-guided therapy recommendations has no effect on pain intensity at 10 days post-surgery.

H_a (alternative hypothesis): Providing the provider with PGx-guided therapy recommendations does affect the pain intensity at 10 days post-surgery.

Analysis: By-treatment differences in pain intensity at 10 days post-surgery within the actionable subgroup will be assessed by comparing mean pain intensity using a two-sided two-sample t-test. The mean difference in pain intensity (standard error) and p-value will be reported.

Using the covariates established in section 7.2, multivariate linear regression will be used to determine the treatment differences in pain intensity after adjusting for baseline characteristics. The by treatment lsmeans estimate, standard error, and the difference in treatment lsmeans estimate, standard error, 95% confidence interval, and p-value will be reported.

7.3.1.3 Composite Pain Intensity Score

Definition: The composite pain intensity score is the sum of the average and worst pain in the past 7 days and current pain, each rated on a 5-point scale.

The hypothesis is:

H_0 (null hypothesis): Providing the provider with PGx-guided therapy recommendations has no effect on pain intensity at 10 days post-surgery.

H_a (alternative hypothesis): Providing the provider with PGx-guided therapy recommendations does affect the pain intensity at 10 days post-surgery.

Analysis: By-treatment differences in composite pain intensity score within the actionable subgroup will be assessed by comparing mean composite pain intensity score using a two-sided two-sample t-test. The mean difference in pain intensity (standard error) and p-value will be reported.

Using the covariates established in section 7.2, multivariate linear regression will be used to determine the treatment differences in the composite pain intensity score, after adjusting for baseline characteristics. The by treatment lsmeans estimate, standard error, and the difference in treatment lsmeans estimate, standard error, 95% confidence interval, and p-value will be reported.

7.3.1.4 Concordance between PGx Phenotype and Prescribed Pain Medications

Definition: The absence of a conflict between the CYP2D6 phenotype and CYP2D6 opioid medication prescription recorded for post-surgery pain management. The list of incompatible CYP2D6 phenotypes and mediations are as follow:

- CYP2D6 IM or PM, after accounting for phenoconversion, prescribed tramadol, hydrocodone, or codeine
- CYP2D6 UM or NM-UM, after accounting for phenoconversion, prescribed tramadol, hydrocodone, codeine, or oxycodone

The hypothesis is:

H_0 (null hypothesis): Providing the provider with PGx-guided therapy recommendations has no effect on the concordance between CYP2D6 phenotypes and prescribed pain medications prescribed for post-surgery pain management.

H_a (alternative hypothesis): Providing the provider with PGx-guided therapy recommendations does affect the concordance between CYP2D6 phenotypes and prescribed pain medications prescribed for post-surgery pain management.

Analysis: By treatment differences in the proportion of concordant participants in the actionable subgroup will be assessed using a test of two-proportions.

Using the covariates established in section 7.2, multivariate logistic regression will be used to determine the treatment differences in the proportion of concordant participants after adjusting for baseline characteristics. The treatment effect parameter (B_1), standard error, odds ratio, 95% confidence interval of the odds ratio, and p-value will be reported.

7.3.1.5 Prescription Pain Mediation Misuse

Definition: The participant responses to the *PROMIS prescription medication mis-use scale* will be scored per the published scoring guidelines. The scores reported at 3-months post-surgery will be evaluated.

The hypothesis is:

H_0 (null hypothesis): Providing the provider with PGx-guided therapy recommendations has no effect on prescription pain medication misuse scores at 3-months post-surgery.

H_a (alternative hypothesis): Providing the provider with PGx-guided therapy recommendations does affect the pain medication misuse scores at 3-months post-surgery.

Analysis: By-treatment differences in prescription pain medication misuse T-score within the actionable subgroup will be assessed by comparing mean T-scores using a two-sided two-sample t-Test. The mean difference in prescription pain medication misuse T-score reduction (standard error) and p-value will be reported.

7.3.1.6 Mobility

Definition: Summed responses to the PROMIS item Bank v2.0 mobility questions at 1-month post-surgery (adults-only).

The hypothesis is:

H_0 (null hypothesis): Providing the provider with PGx-guided therapy recommendations has no effect on mobility scores at 1-month post-surgery.

H_a (alternative hypothesis): Providing the provider with PGx-guided therapy recommendations does affect the mobility score at 1-month post-surgery.

Analysis: The by treatment differences in the 1-month adult mobility scores within the adult participants in the actionable subgroup will be assessed by comparing mean T-scores using a two-sided two-sample t-test. The mean difference in mobility score (standard error) and p-value will be reported.

7.3.1.7 Opioid Persistence

Definition: Having an opioid prescription filled 90-180 days post-surgery, among the participants who reported one or more filled opioid prescriptions at 10-days.

The hypothesis is:

H_0 (null hypothesis): Providing the provider with PGx-guided therapy recommendations has no effect on opioid persistence at 90-180 days post-surgery.

H_a (alternative hypothesis): Providing the provider with PGx-guided therapy recommendations does affect the change on opioid persistence at 90-180 days post-surgery.

Analysis: By treatment differences in the proportion of participants with opioid persistence in the actionable subgroup will be assessed using a test of two proportions. Difference in proportion and the 95% confidence interval will be reported.

7.4 Exploratory Analyses of the Primary Endpoint

7.4.1 SIA Using Alternative Pain Measure

An exploratory analysis using an alternative SIA score derivation will be conducted in the actionable phenotype subgroup. The SIA score will be re-calculated using *prescribed* opioid usage and the composite pain intensity score, instead of the numeric pain rating scale.

Analysis: By-treatment differences in this alternative SIA score calculation at 10 days post-surgery within the actionable subgroup will be assessed by comparing alternative SIA score using a two-sided two-sample t-test. The mean difference in the alternative SIA score (standard error) and p-value will be reported.

Using the covariates established in section 7.2, multivariate linear regression will be used to determine the treatment differences in the alternative SIA score calculation after adjusting for baseline characteristics. The by treatment lsmeans estimate, standard error, and the difference in treatment lsmeans estimate, standard error, 95% confidence interval, and p-value will be reported.

7.4.2 Per-Protocol Subgroup of Special Interest

An exploratory analysis of the primary endpoint will be conducted in the per-protocol population defined in section 4.3. This protocol-specified sub-population analysis uses post-baseline data to test the following hypothesis:

H_0 (null hypothesis): Provider adherence with PGx-guided therapy recommendations has no effect on the SIA scores at 10-days post-surgery.

H_a (alternative hypothesis): Provider adherence with PGx-guided therapy recommendations does affect the SIA score 10-days post-surgery.

A linear regression will be used to estimate the treatment effect on SIA scores within the subgroup. The treatment effect will be estimated as the difference in the least squares mean (lsmean) estimates for the by treatment mean SIA scores. The lsmean estimates, standard error, and the difference in lsmeans, standard error, 95% confidence interval, and p-value will be reported. If any baseline features are imbalanced between treatment arms within this subgroup, those features will be included as covariates in the linear model. This model will be estimated first without, then with the baseline covariates defined in section 7.2.

7.4.3 Non-oxycodone Per-Protocol Subgroup of Special Interest

An exploratory analysis of the primary endpoint will be conducted in the subset of the per-protocol population not prescribed oxycodone defined in section 4.4. The body of literature surrounding the effect of CYP2D6 phenotypes on analgesic response to oxycodone is unclear. At the outset of the ADOPT PGx trial, data showed an effect of CYP2D6 phenotypes on oxycodone metabolism, but this did not appear to translate into effects on analgesic response. Thus, PGx efficacy recommendations for oxycodone were not included in the intervention. Recently published data suggested otherwise, showing that patients prescribed oxycodone with a CYP2D6 inhibitor had more emergency department visits compare to those prescribed oxycodone without an inhibitor, providing evidence that CYP2D6 phenotype maybe be an important factor influencing oxycodone response. This subgroup analysis will be used to test the following hypothesis:

H_0 (null hypothesis): Provider adherence with PGx-guided therapy recommendations and excluding oxycodone has no effect on the SIA scores at 10-days post-surgery.

H_a (alternative hypothesis): Provider adherence with PGx-guided therapy recommendations and excluding oxycodone does affect the SIA score 10-days post-surgery.

A linear regression will be used to estimate the treatment effect on SIA scores within the subgroup. The treatment effect will be estimated as the difference in the least squares mean (lsmean) estimates for the by treatment mean SIA scores. The lsmean estimates, standard error, and the difference in lsmeans, standard error, 95% confidence interval, and p-value will be reported. If any baseline features are imbalanced between treatment arms within this subgroup, those features will be included as covariates in the linear model. This model will be estimated first without, then with the baseline covariates defined in section 7.2.

7.4.4 Non-actionable Complementary Subgroup

An exploratory analysis of the primary endpoint will be used to assess the intervention effect in the non-actionable population, defined in section 4.5, using the SIA score derived in the ITT population with completed surgeries, not prescribed liquid forms of opioids for pain control, discharged prior to the 10-day survey, and able to report their medication usage 10-days post-surgery. In this subgroup, we will

assess if the treatment effect on SIA scores differs between participants who have an actionable CYP2D6 PGx test result and those who do not have an actionable CYP2D6 PGx test result.

H_0 (the null hypothesis): The treatment effect on pain control is the same for participants with an actionable PGx test result, compared to participants without an actionable PGx test result

H_a (the alternative hypothesis): The treatment effect on pain control is different for participants with an actionable PGx test result, compared to participants without an actionable PGx test result.

Linear regression will be used to estimate the treatment effect, PGx actionability effect, and the interaction between treatment and PGx actionability on SIA scores. The interaction term p-value will be reported. The treatment effect will be estimated as the difference in the least squares mean (lsmean) estimates for the by treatment mean SIA scores, within each PGx actionability group (i.e. actionable and non-actionable). The lsmean estimates, standard error, and the difference in lsmeans, standard error, 95% confidence interval, and p-value for the by group treatment effects will be reported. If any baseline features are imbalanced between treatment arms within this subgroup, those features will be included as covariates in the linear model. This model will be estimated first without, then with the baseline covariates defined in section 7.2.

7.4.5 Differences by Surgery Type

An exploratory analysis of the primary endpoint will be conducted to assess the intervention effect by surgery type: total knee arthroplasty vs total hip arthroplasty among participants who had either total knee or total hip arthroplasty, using the SIA score derived in the mITT population.

H_0 (the null hypothesis): The treatment effect on pain control is consistent across surgery types.

H_a (the alternative hypothesis): The treatment effect on pain control is variable by surgery type.

A linear regression will be used to estimate the treatment effect, surgery-type effect, and the interaction between treatment and surgery type on SIA scores within the mITT population. The interaction p-value will be reported and the within surgery type treatment effect will be estimated as the difference in the least squares mean (lsmean) estimates for the by treatment mean SIA scores. The within treatment arm difference between surgery types will be similarly estimated. The lsmean estimates, standard error, and the difference in lsmeans, standard error, 95% confidence interval, and p-value will be reported. This model will be estimated first without, then with the baseline covariates defined in section 7.2.

7.4.6 Participants' Interaction with PGx Test Results

An exploratory analysis of the primary endpoint will be conducted to assess if participant's interaction with their PGx test result impacts the SIA scores at 10-days. Participants randomized to the PGx guided therapy arm who indicated that they interacted with their PGx test result through looking up their result in their medical record, requesting a copy of the result, or discussing with their provider will be referred to as having reviewed their PGx test result.

H_0 (the null hypothesis): Participant review of their PGx test result has no effect on pain control.

H_a (the alternative hypothesis): Participant review of their PGx test result affects their pain control.

Within the subset of the ITT population randomized to immediate PGx guided therapy and who had completed surgeries using the SIA score derived in the ITT population, a linear regression will be used to estimate the participant review of PGx results effect, PGx actionability, and the interaction between participant review of PGx results and PGx actionability on SIA scores. The interaction p-value will be reported. Within actionability group, the effect of participant review their PGx test result on SIA scores will be estimated as the difference in the within group least squares mean (lsmean) estimates mean SIA scores. The lsmean estimates, standard error, and the difference in lsmeans, standard error, 95% confidence interval, and p-value will be reported. This model will be estimated first without, then with the baseline covariates defined in section 7.2.

7.5 Exploratory Analyses

For analyses using covariate-adjusted models, potential baseline covariates included will be the same as those used for the covariate-adjusted primary endpoint analysis.

For all analyses using mixed model for repeated measures, appropriate covariance matrix (*e.g.*, compound symmetry, autoregressive, variance components, or other covariance structure) will be selected based on the data to account for the correlated endpoints from individual participants. We will select the covariance structure that has the smallest Akaike's Information Criteria (AICC) value. When two or more covariance structures yield a similarly small AICC value, the most parsimonious model with the fewest number of parameters (*i.e.*, distinct covariance values) will be selected.

7.5.1 SIA Score at 1 Month

We will compare the SIA scores at 1 month between the two randomized treatment groups (*i.e.* Immediate PGx testing with genotype guided therapy and delayed PGx testing), using the same t-test and covariate-adjusted linear regression described previously in sections 7.1 and 7.2, respectively.

7.5.2 Prescribed Opioid Usage at 1 Month

We will compare the opioid usage at 1 month between the two randomized treatment groups (*i.e.* Immediate PGx testing with genotype guided therapy and delayed PGx testing), using the same t-test and covariate-adjusted linear regression described previously.

7.5.3 Time Trends in Pain Intensity

We will compare the time trends in the pain intensity at 10 days, 1, 3, and 6 months between the two randomized treatment groups (*i.e.* Immediate PGx testing with genotype guided therapy and delayed PGx testing), using a repeated-measures mixed effect model with a baseline-value, with and without the baseline-covariates adjustments defined in section 7.2.

7.5.4 Time Trends in Composite Pain Intensity Scores

We will compare the time trends in in the composite pain intensity scores at 10 days, 1, 3, and 6 months between the two randomized treatment groups (*i.e.* Immediate PGx testing with genotype guided

therapy and delayed PGx testing), using a repeated-measures mixed effect model with a baseline-value, with and without the baseline-covariates adjustments defined in section 7.2.

7.5.5 Potential Post-Hoc Analyses

While there are no known systematic disruptions in the trial conduct, patterns underlying missing data in the primary endpoints and the genotype results will be assessed for systematic differences that persist across the entire trial period after unblinding. If evidence of such systematic differences arises, post-hoc analysis with adjustments may be considered.

7.6 Handling of Missing Data

If the SIA score is missing in 10% or more of the observations in the actionable subgroup population, sensitivity analysis will be performed using data obtained from multiple imputation. The sensitivity analysis will be performed for the primary analysis and the covariate-adjusted analyses for the primary endpoint. Multiple imputation will be used to impute all missing pain intensity scores and opioids usage values except for those due to participant death or due to study-discontinuation due to not having the surgery completed at the participating healthcare institution as expected.

Under the missing at random (MAR) assumption, subjects who discontinue early **after their surgery** and are alive are assumed to have the same data pattern as subjects who remain in the study in the same treatment arm. Multiple imputation will be carried out to impute missing data for at baseline and 10-days.

Step 1 - First, the intermittent pain intensity and opioid usage will be imputed for each treatment relying on non-missing data from all subjects within each treatment group using the Markov Chain Monte Carlo (MCMC) imputation model with baseline pain intensity, covariates (as identified in section 7.2), and the available non-missing opioid usage and pain intensity data to generate a monotone missing data pattern. Then, missing data (i.e. 10-day opioid usage and 10-day pain intensity) will be imputed to derive 20 imputed datasets with non-missing data. It is anticipated that 20 imputations will be sufficient to achieve stable parameter estimates. 20 imputations are expected to be sufficient to achieve <5% change in the standard error estimates when the fraction of missing information is < 31%.

Step 2 - The 20 multiple-imputation datasets with imputed and observed pain intensity and opioid usage at 10-days will be used to derive the SIA score endpoint. Each imputation dataset will be analyzed separately, the results of which will be averaged to obtain the final treatment effect estimates.

The imputed values will be used for sensitivity analyses only and will not be included in the primary analysis.

8 Safety Evaluation

ADOPT-PGx is a study regulated under an Abbreviated Investigational Device Exemption (IDE). The genotyping-guided therapy is the device and classified as a minimal risk to the welfare of the enrolled participants. The following safety data will be presented by randomized treatment for safety evaluation:

- Early termination and discontinuation
- Self-reported emergency department visits and hospitalizations
- Adverse effects occurring during treatment with pain medications*
- Unanticipated study-related deaths
- Reportable adverse device effects or unanticipated adverse device effects

*Medication side effects captured only for participants reporting that they have taken a prescription for pain medicine in the past 7 days at the time of the survey.

Safety evaluation will be performed among all participants in the actionable subgroup (mITT) and ITT populations.
