# Supplementary material for: CYP2D6-Guided Opioid Management and Postoperative Pain Control: A Randomized Clinical Trial
Source: JAMA Netw Open. 2026 Feb 20;9(2):e2558299. doi: 10.1001/jamanetworkopen.2025.58299 (PMC12924106; doi:10.1001/jamanetworkopen.2025.58299)
Supplement: Supplement 3. — Group Information [file jamanetwopen-e2558299-s003.pdf]

Supplemental Online Content: Nonauthor Collaborators
*First name, last name, and suffix (if applicable) are required and will appear in PubMed.

*Group Name(s): IGNITE Pragmatic Trials Network							
***First Name and Middle Initial(s)**	***Last Name**	***Suffix (eg, Jr, III)**	Academic Degrees	Institution	Location (city, state/province, country)	Role or Contribution, eg, chair, principal investigator	Group (if more than 1 Group listed in the byline) and/or Subgroup (eg, Steering Committee)
Neil	Calman						
Kerri	Cavanaugh						
Geoff	Ginsburg						
Kady-Ann	Steen-Burrell						
Bhargav	Adagarla						
Sarah	George						
Phyllis	Kennel						
Kristen	Linney						
Rania	Metry						
Wanda	Parker						
Gayle	Passmore						
Carol	Pereira						
Teji	Rakhra-Burris						
Jaclyn	Holland						
Yashika	Johnson						
Ryanne	Wu						
Ruth	Lehan						
Tyffany	Locklear						
Sabrina	Clermont						
Bart	Ferket						
Kenneth	Fifer						
Diane	Hauser						
Joseph	Kannry						
James	Murrough						
Janet	Seo						
Tatiana	Sabin						
Nandini	Shroff						
Saskia	Shuman						
Abi	Colwell						
Amy M.	Breman						

Supplemental Online Content: Nonauthor Collaborators
*First name, last name, and suffix (if applicable) are required and will appear in PubMed.

***First Name and Middle Initial(s)**	***Last Name**	***Suffix (eg, Jr, III)**	Academic Degrees	Institution	Location (city, state/province, country)	Role or Contribution, eg, chair, principal investigator	Group (if more than 1 Group listed in the byline) and/or Subgroup (eg, Steering Committee)
Zeru	Desta						
Cathy	Fulton						
Jennelle C.	Hodge						
Sheryl	Lynch						
Jonathan	Oliver						
Victoria M.	Pratt						
Ross	Robinson						
Elizabeth	Rowe						
Jennifer	Stuart						
Ashely	Vetor						
Kelsey	Cook						
Karam	Diaby						
Julio	Duarte						
Ben	Duong						
Karla	Giron						
Elizabeth	Eddy						
Julia	Krutov						
Carol	Mathews						
Haesuk	Park						
Robyn	Nelson						
Ryan	Rhoden						
Caroline	Schlierle						
Taylor	Sullivan						
Almut	Winterstein						
Kristen	Wiisanen						
Sara	Van Driest						
Sara	Block						
Michelle	Benck						
Chantel	Bender						
Sylvia	Eluhu						
Colette	Free						
Shane	Gonnelly						

Supplemental Online Content: Nonauthor Collaborators
*First name, last name, and suffix (if applicable) are required and will appear in PubMed.

***First Name and Middle Initial(s)**	***Last Name**	***Suffix (eg, Jr, III)**	Academic Degrees	Institution	Location (city, state/province, country)	Role or Contribution, eg, chair, principal investigator	Group (if more than 1 Group listed in the byline) and/or Subgroup (eg, Steering Committee)
Sarah	Hedeen						
Steven	Houtschilt						
Michelle	Liu						
Salisha	Marryshow						
Nicole	Neville						
Natasha	Petry						
Sidd	Pratap						
Vernon	Sherden						
Kimberly	Snell						
Ana	Tomescu						
Megan	Trietsch						
James	Walker						
Deanna	Web						
Erin	Whiting						
Russell A.	Wilke						
Sarah	Hutchison						
Natalie	Kucher						
Ebony	Madden						
Jessica	Reinach						
Ismail	Safi						
Ella	Samer						
Lani	Banez						
Lisa	Bendz						
Jackie	Jordan						
Ann	McGee						
Christina	Nix						
Lorraine	Vergara						
Tyler	Colon						
Aaishah	Francis						
Najiba	Khan						
Camila	Tan Lam						
Michelle	Sciarrino						

Supplemental Online Content: Nonauthor Collaborators
*First name, last name, and suffix (if applicable) are required and will appear in PubMed.

*First Name and Middle Initial(s)	*Last Name	*Suffix (eg, Jr, III)	Academic Degrees	Institution	Location (city, state/province, country)	Role or Contribution, eg, chair, principal investigator	Group (if more than 1 Group listed in the byline) and/or Subgroup (eg, Steering Committee)
Emma	Maiman-Stadtmauer						
Sofia	Medina-Pardo						
Ololade (Lola)	Williams						
Sheng	Luo						
Kevin	Anstrom						
Alicia	Ellis						
Stephen	Ellis						
Hwasoon	Kim						
Peter	Merrill						
Rachel	Myers						
Lilin	She						
Jennifer	Shepherd						
Saira	Siddiqui						
Jun	Wen						
John	Howard						
Alia	Jamison						
Lydia	Joyner						
Ellie	Kiihne						
Libbie	Silverman						
Jordan	Sonneville						
Laurel	Tokar						
Daviana	Buck						
Nicole	Canchucaja						
Porshia	Cook						
Ariel	Jacobs						
Danielle	John						
Samantha	Guagliardo						
Mirta (Mimi)	Milanes						
Wambui	Ngari						
Megan	Triestch						
Abraham	Garcia						

Supplemental Online Content: Nonauthor Collaborators
*First name, last name, and suffix (if applicable) are required and will appear in PubMed.

***First Name and Middle Initial(s)**	***Last Name**	***Suffix (eg, Jr, III)**	Academic Degrees	Institution	Location (city, state/province, country)	Role or Contribution, eg, chair, principal investigator	Group (if more than 1 Group listed in the byline) and/or Subgroup (eg, Steering Committee)
Carol	Gutierrez						
Maria	Lopez						
Alan	Mejia						
Anaite	Montes Bu						
Deanna	Webb						
Sonja	White						
Ali	Brewer						
Brittni	Deadrick						
Alexis	Meyer						
Blair	Svennes						
Hana	Al Alshaykh						
Tala	Basha						
Emily	Cicali						
Asia	Cobb						
Brendon	Cooper						
Amanda	Elchynski						
Amanda	Elsey						
Anuksha	Gotmare						
Lauren	Lemke						
Arielle	Nelson						
Michael	Read						
Genevieve	Rosier						
Isabella	Schultz						
Chiara	Chermont Spina						
Joshua	Terrell						
Alexa	Valko						
Precious	Williams						
Tracy	Leonard						
David	Smith						
Marian	Davis						
Vincent	Guerra						
Jennifer	Salo						

Supplemental Online Content: Nonauthor Collaborators
*First name, last name, and suffix (if applicable) are required and will appear in PubMed.

***First Name and Middle Initial(s)**	***Last Name**	***Suffix (eg, Jr, III)**	Academic Degrees	Institution	Location (city, state/province, country)	Role or Contribution, eg, chair, principal investigator	Group (if more than 1 Group listed in the byline) and/or Subgroup (eg, Steering Committee)
Claudia	Cartaya Torres						
Jorge	Valedon						
Paola	Nieves Viana						
Alexander	Parker						
Jose	Alonso						
Jeffery	Gainer						
Morgan	Howard						
Fatoumata	Kaba						
Brianna	Rivers						
Liliana	Serrano						
Kimberly	Vigal						
ADeia	Williams						
Angelo	Lazaro						
Salisha	Merryshow						
Henry	Ong						
Asiyanbola	Bolanle						
John Thomas	Callaghan						
Justin	Deen						
Michael T.	Eadon						
Daniel	Getu						
Blake	Goff						
Joseph	King						
Eric A.	Larson						
Brandi	Plunkett						
Luis	Pulido						
Marc B.	Rosenman						
Robert	Van Demark, Jr						
Walter W.	Virkus						
Thomas	Wright						
Jonathan	Wright						
