# Supplementary material for: CYP2D6-Guided Opioid Management and Postoperative Pain Control: A Randomized Clinical Trial
Source: JAMA Netw Open. 2026 Feb 20;9(2):e2558299. doi: 10.1001/jamanetworkopen.2025.58299 (PMC12924106; doi:10.1001/jamanetworkopen.2025.58299)
Supplement: Supplement 4. — Data Sharing Statement [file jamanetwopen-e2558299-s004.pdf]

Data Sharing Statement

Cavallari. CYP2D6-Guided Opioid Management and Postoperative Pain Control. *JAMA Netw Open*. Published February 20, 2026. doi:10.1001/jamanetworkopen.2025.58299

Data
Additional Information: ClinicalTrials.gov; Identifier NCT04445792 (https://clinicaltrials.gov/search?term=NCT04445792).
Data available: Yes
Data types: Deidentified participant data, Data dictionary
How to access data: Deidentified data and data dictionaries are available via the National Human Genome Research Institute (NHGRI) Genomic Analysis, Visualization and Informatics Lab-space (AnVIL) (https://anvilproject.org/), PHS#: ADOPT PGx Acute Pain Trial: phs004058.v1.p1. Datasets will be designated as controlled access, and researchers will be able to apply to National Institutes of Health (NIH) data access committees (DACs) for use of these datasets.
When available: With publication

Supporting Documents
Document types: Other (please specify)
Additional Information: Protocol, data dictionary, case report forms, informed consent forms, descriptions of derived variables.
How to access documents: NHGRI Genomic Analysis, Visualization and Informatics Lab-space (AnVIL): https://anvilproject.org/
When available: With publication

Additional Information
Who can access the data: Individuals whose proposed use of the data receives approval from NHGRI AnVIL independent data-access committee established for review of such requests.
Types of analyses: Dataset will be available for health/medical/biomedical use with a subset of the data also available for general research use.
Mechanisms of data availability: Download after approval
